# Mechanism of Cu-Catalyzed Iododeboronation: A Description of Ligand-Enabled Transmetalation, Disproportionation, and Turnover in Cu-Mediated Oxidative Coupling Reactions

**DOI:** 10.1021/acscatal.3c02839

**Published:** 2023-08-07

**Authors:** Matthew J. Andrews, Ambre Carpentier, Alexandra M. Z. Slawin, David B. Cordes, Stuart A. Macgregor, Allan J. B. Watson

**Affiliations:** † EaStCHEM, School of Chemistry, University of St Andrews, Purdie Building, St Andrews KY16 9ST, U.K.; ‡ Institute of Chemical Sciences, Heriot-Watt University, Edinburgh EH14 4AS, U.K.

**Keywords:** Cu-catalyzed iododeboronation, DFT analyses, Cu-mediated oxidative coupling reactions

## Abstract

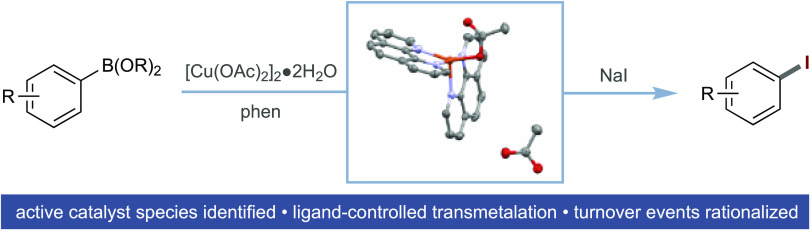

We report a combined experimental and computational study of the mechanism of the Cu-catalyzed arylboronic acid iododeboronation reaction. A combination of structural and density functional theory (DFT) analyses has allowed determination of the identity of the reaction precatalyst with insight into each step of the catalytic cycle. Key findings include a rationale for ligand (phen) stoichiometry related to key turnover events—the ligand facilitates transmetalation via H-bonding to an organoboron boronate generated in situ and phen loss/gain is integral to the key oxidative events. These data provide a framework for understanding ligand effects on these key mechanistic processes, which underpin several classes of Cu-mediated oxidative coupling reactions.

## Introduction

**Scheme 1:**
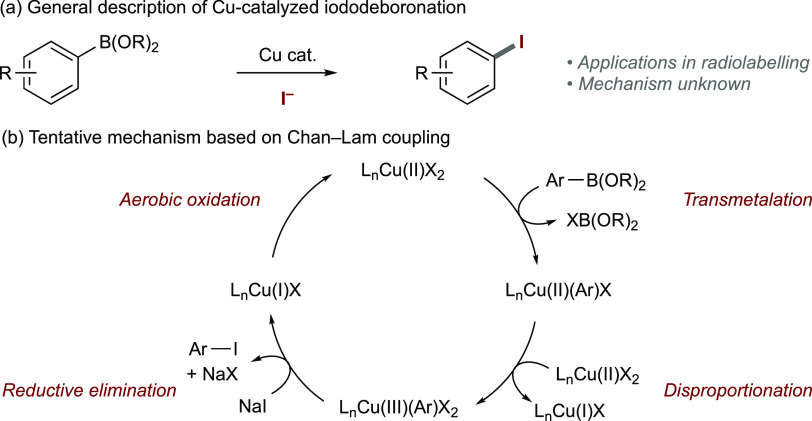
(a) General Representation of Cu-Catalyzed Iododeboronation and (b) Tentative Description of the Iododeboronation Reaction^9^

Cu-catalyzed iododeboronation of arylboronic acids using iodide is a useful reaction for the synthesis of aryl iodides (Scheme 1a).^1−4^ More importantly, this reaction accommodates iodide radioisotopes providing products that have important applications within in vivo imaging and radiotherapy.^5−9^

There are several reported methods for Cu-catalyzed iododeboronation, each with subtle variations in the copper source, ligand, and associated reaction conditions.^1−4^ Related copper-catalyzed or -mediated halodeboronation reactions, such as the equivalent fluorination reaction, also operate with similar reaction conditions.^10−13^ This general reaction class therefore has broad applications across synthetic chemistry and specific applications in bio-facing fields.

Very limited mechanistic information is available on the halodeboronation reaction in general, with a tentative mechanistic description of the iododeboronation largely based on the framework of the Chan–Lam reaction (Scheme 1b).^9,14−18^ This proposed mechanism involves transmetalation of the arylorganoboron compound to Cu(II), giving a Cu(II)(aryl) species, followed by disproportionation^19^ to Cu(III) and anion exchange/reductive elimination to give the aryl iodide product and Cu(I). Aerobic reoxidation of Cu(I) to Cu(II) closes the cycle.

Despite the importance and influence that ligand stereoelectronics exert on redox processes at metal centers, robust identification of intermediate ligand structures and speciation states remains a general problem in Cu-mediated oxidative catalysis. This renders the study of key mechanistic events, such as transmetalation, disproportionation, and oxidative turnover, difficult to understand and, therefore, control through rational catalyst design.

The main knowledge in this area arises from several key studies of catalytic reactions. Hartwig identified a Cu(III) intermediate in solution and proposed a mechanism for transmetalation of arylboronic acid pinacol (ArBpin) esters in a fluorodeboronation reaction using an F^+^ reagent, with ligands proposed for this key intermediate and event.^11^ Stahl,^15,16^ Watson,^17,20^ and Schaper^21,22^ proposed ligand sets for related Chan–Lam etherification and amination reactions, with broadly similar descriptions of rate-limiting transmetalation.^23^ Computational analysis of Chan–Lam amination reactions have aligned closely, although with some differences in the interpretation of the rate-limiting event.^24^ While being integral to many Cu-based oxidative coupling reactions, there is limited information on the disproportionation event or oxidative turnover.

Here, we report a detailed mechanistic description of the Cu-catalyzed iododeboronation reaction using a combination of structural, spectroscopic, and computational analyses. For the first time, this includes an assessment of ligand structures at all stages of the proposed catalytic cycle. More broadly, this analysis has provided insight into the transmetalation, disproportionation, and oxidative turnover events that underpin many Cu-mediated oxidative coupling reactions.

## Results and Discussion

### Synthesis and Characterization of Cu Complexes

**Scheme 2:**
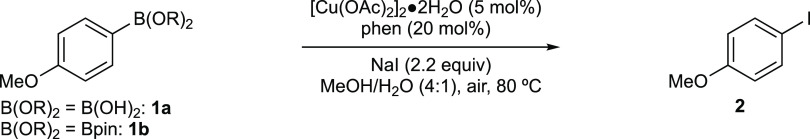
Model Iododeboronation System Used in This Study

For our analysis, we selected a catalytic iododeboronation system reported by Gouverneur using [Cu(OAc)_2_]_2_·2H_2_O and 1,10-phenanthroline (phen) as the catalyst/ligand system and NaI as the requisite source of iodide (Scheme 2).^7^ Based on these conditions, we sought to prepare complexes that may be formed in situ.

**Scheme 3:**
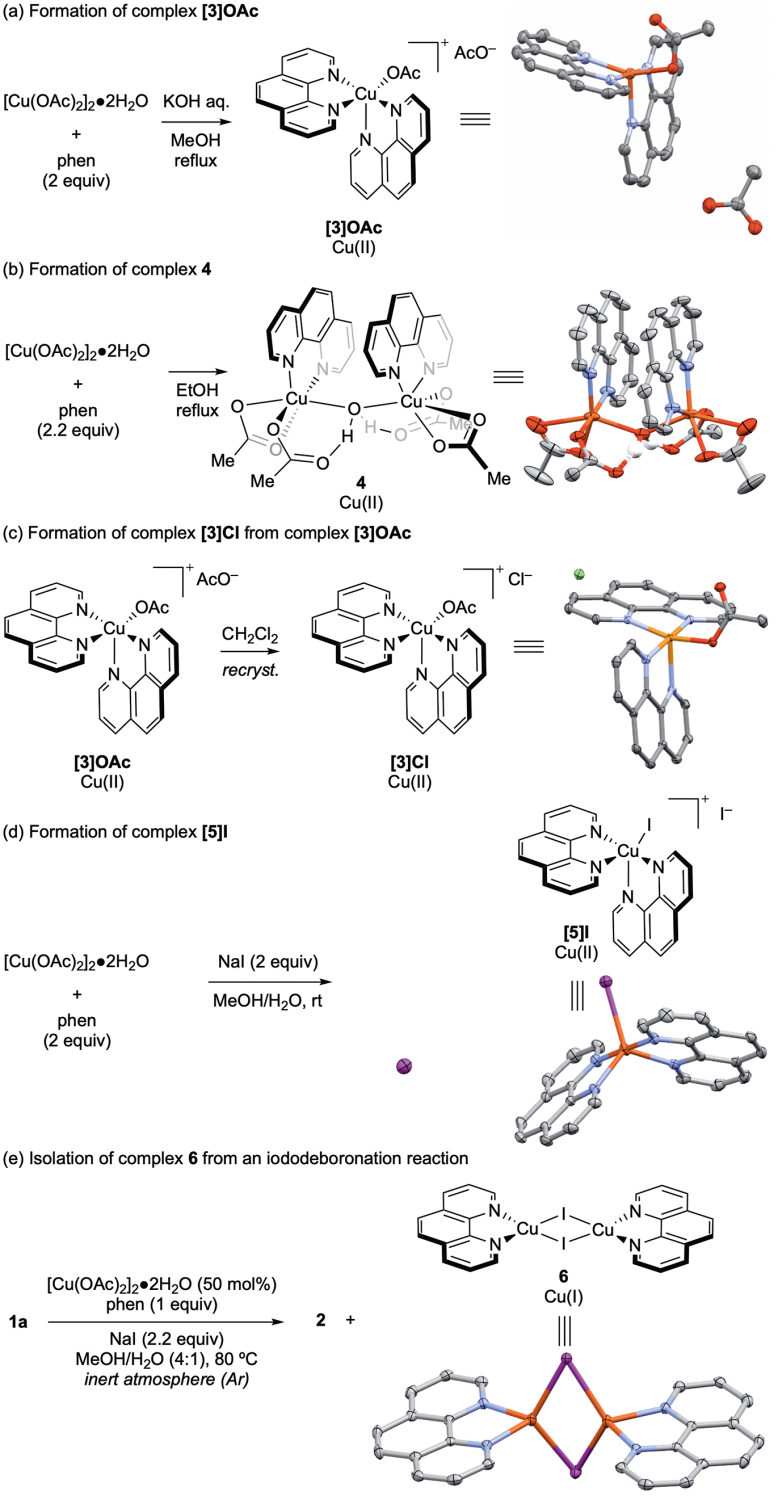
Formation of Cu Complexes **3**–**6**

Treatment of [Cu(OAc)_2_]_2_·2H_2_O with phen delivered the monomeric complex [Cu(OAc)(phen)_2_]OAc (**[3]OAc**)^25^ or the dimeric complex [Cu(OAc)_2_(phen)]_2_·μ-H_2_O (**4**),^26^ following previous literature procedures (Scheme 3a). Recrystallization of **[3]OAc** in CH_2_Cl_2_ fortuitously led to the formation of [Cu(OAc)(phen)_2_]Cl (**[3]Cl**) (Scheme 3c). Treatment of [Cu(OAc)_2_]_2_·2H_2_O with phen under halodeboronation reaction conditions (NaI, MeOH/H_2_O) led to the formation of [Cu(I)(phen)_2_]I (**[5]I**) (Scheme 3d).^27^ A crystalline material from the reaction mixture of a completed iododeboronation reaction of **1a** conducted under an inert atmosphere was isolated and determined to be the dimeric complex [Cu(μ-I)(phen)]_2_ (**6**) (Scheme 3e).^28^

### Identification of Reaction-Relevant Cu Complexes

To elucidate the function of reaction components and identify reaction-relevant [Cu] + phen (2 equiv) Cu complexes, [Cu(OAc)_2_]_2_·2H_2_O was first treated with NaI; however, no reaction or denucleation of the paddlewheel was observed by electron paramagnetic resonance (EPR) spectroscopy (not shown, see Figure S13).

**Figure 1:**
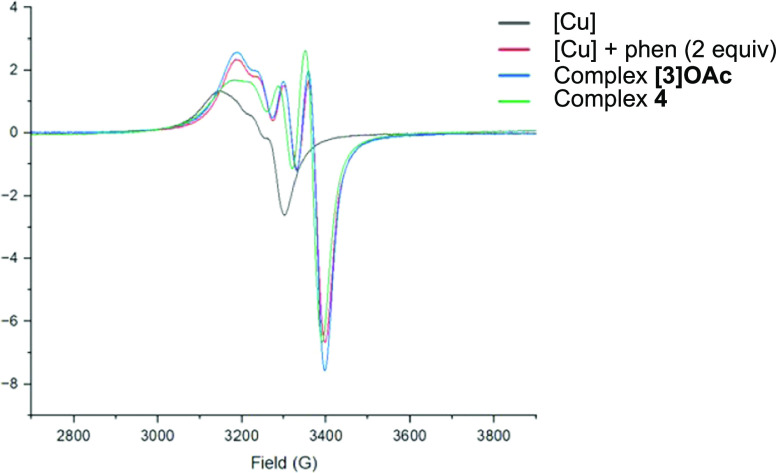
Overlaid EPR spectra for [Cu(OAc)_2_]_2_·2H_2_O (black), complex **[3]OAc** (blue), complex **4** (green), and the proposed in situ formation of a complex through complexation of **[3]OAc** by treatment of [Cu(OAc)_2_]_2_·2H_2_O with phen (2 equiv) (red). Solvent = MeOH/H_2_O (4:1). [Cu] = [Cu(OAc)_2_]_2_·2H_2_O.

We therefore hypothesized that the reaction initiates by the formation of a Cu(II)(phen)*_n_* complex. Treatment of [Cu(OAc)_2_]_2_·2H_2_O with phen (2 equiv) and analysis by EPR spectroscopy showed the formation of a complex with a spectrum consistent with that of **[3]OAc** and less consistent with that of **4** (Figure 1). This suggested that **[3]OAc** was the dominant species arising from reaction-relevant Cu:phen stoichiometry (1:2).

**Scheme 4:**
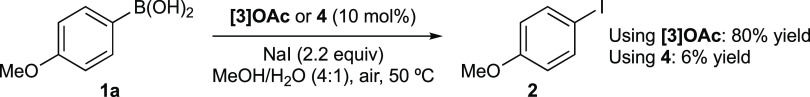
Competency of **[3]OAc** and **4** in the Iododeboronation of **1a** Yields determined by ^1^H NMR using an internal standard.

Optimization of temperature and time was carried out, which allowed a range of complexes to be evaluated for catalytic performance (not shown, see SI Sections S2.2–S2.4). Using preformed **[3]OAc** or **4** in the iododeboronation reaction of **1a** delivered the expected product **2**, indicating reaction relevance and catalytic competency (Scheme 4); however, while **[3]OAc** exhibited catalytic turnover, **4** did not.

Note that to avoid complications in data analysis, **1a** was used to avoid off-cycle inhibitory processes that are associated with release of pinacol from reactions using ArBpin (i.e., **1b**; see SI Section S6).^17^ In addition, while it is possible to prepare Cu(II)(phen)_3_ complexes,^29^ these are not readily accessible under conditions relevant to the iododeboronation process and, as such, were discounted from this study.

**Figure 2:**
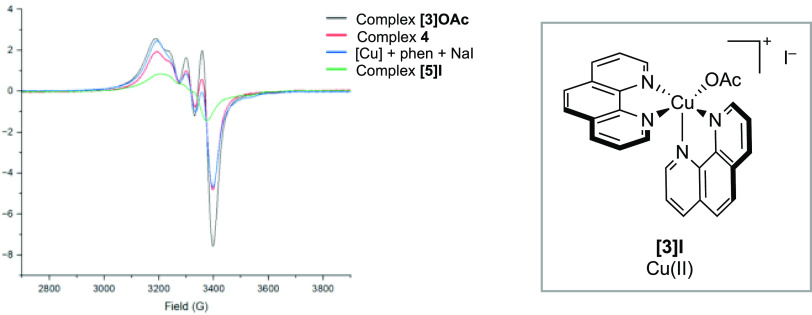
Overlaid EPR spectra for compound **[3]OAc** (black), complex **4** (red), complex **[5]I** (green), and the proposed in situ formation of **[3]I** through reaction of [Cu(OAc)_2_]_2_·2H_2_O with phen (2 equiv), and NaI (2 equiv) (blue).

Treatment of [Cu(OAc)_2_]_2_·2H_2_O with phen and NaI and analysis by EPR spectroscopy revealed a solution structure consistent with complexes **[3]OAc** and **[3]Cl**, with the observation of increased line broadening, which were significantly different to the spectrum of **[5]I** (Figure 2). Based on these data, we propose the formation of **[3]I** in situ, which subsequently proceeds to **[5]I** via further anionic ligand metathesis. Attempts to isolate **[3]I** were unsuccessful—all attempts led to isolation of **[5]I**.

Use of complexes **[3]Cl** and **[5]I** in halodeboronation reactions was informative (Table 1). Stoichiometric **[3]Cl** delivered only 16% of the expected chloroarene product **7** while stoichiometric **[5]I** delivered quantitative conversion to **2** (entries 1 and 2). Similar effects were observed when using catalytic **[3]Cl** and **[5]I** in the presence of NaCl and NaI, respectively (entries 3 and 4): poor conversion was observed with **[3]Cl** but **[5]I** was competent. Interestingly, using NaI as the halide source, **[3]Cl** was catalytically competent and delivered exclusively the iododeboronation product **2** (**7** not observed) despite the presence of 10 mol % chloride (entry 5). The reciprocal reaction using **[5]I** with NaCl delivered iododeboronation commensurate with the presence of 20 mol % iodide (from 10 mol % **5**) as expected, but low levels of chlorodeboronation despite the presence of excess chloride (entry 6). These data suggest that (i) **[3]Cl** and **[5]I** are catalytically competent, (ii) complex **[3]**^**+**^ is likely a key on-cycle species and exists in equilibrium with, or is a precursor to, **[5]**^**+**^, and (iii) iodide transfer is more efficient than chloride. This latter point begins to impact upon understanding of halodeboronation in general, in particular aligning with the more difficult fluorodeboronation process.^10−13^

**Table 1:**
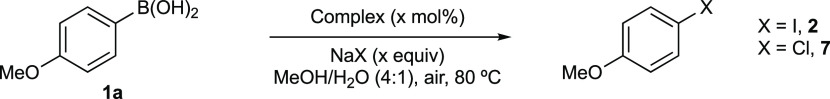
Halodeboronation Competency and Halide Effect of Complexes **[3]Cl** and **[5]I**

entry	complex (loading)	NaX (equiv)	yield (product)^a^
1	**[3]Cl** (1 equiv)		16% (**7**)
2	**[5]I** (1 equiv)		>99% (**2**)
3	**[3]Cl** (10 mol %)	NaCl (2.2)	20% (**7**)
4	**[5]I** (10 mol %)	NaI (2.2)	83% (**2**)
5	**[3]Cl** (10 mol %)	NaI (2.2)	67% (**2**)
6	**[5]I** (10 mol %)	NaCl (2.2)	16% (**2**)
			17% (**7**)

a Yields determined by ^1^H NMR using an internal standard.

In situ reaction monitoring and sequential addition of reagents showed productive reactivity was only observed with the addition of NaI prior to **1a** (not shown, see Figures S25 and S26). In addition, in the absence of NaI, low levels of side reactions were observed (ca. 18% in total, see Figure S27). Attempts to identify Cu(II)(aryl) complexes were unsuccessful. These observations were consistent with transmetalation being rate-limiting, as indicated through computational studies (vide infra), and for other reactions in this class, e.g., Chan–Lam processes.^14^ This suggested that the [Cu(II)(aryl)]^+^ complex can be intercepted by H_2_O or MeOH, with formation of minor side products. The effect of added I^−^ could in principle arise from **[3]I** being able to undergo faster transmetalation than **[3]OAc** although this seems unlikely. Instead we propose that I^–^ can competitively intercept the resulting [Cu(II)(aryl)]^+^ complex with the Cu(II)(aryl)(I) complex being more favorable for the subsequent mechanistic event. This last possibility is borne out by our computational studies.

**Figure 3:**
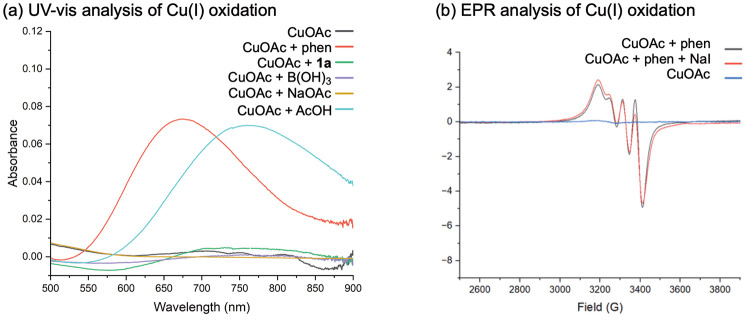
(a) Oxidation of CuOAc in air and in the absence/presence of reaction components. (b) EPR spectra of oxidation of CuOAc + phen/NaI under air.

We hypothesized that Cu(I) complex **6** was produced following reductive elimination of the product from a Cu(III) intermediate. This complex was notable due to the 1:1 Cu:phen ratio, which implied loss of one phen ligand from the proposed intermediate **[3]**^**+**^. To enable catalysis, **6** would require oxidation to Cu(II) in the presence of air (the terminal oxidant used in the iododeboronation process). Oxidation of CuOAc under air was therefore monitored by ultraviolet–visible (UV–vis spectroscopy) (Figure 3a) and in the presence of other reaction-relevant additives. Oxidation was not observed in air or in the presence of NaOAc or substrate **1a**. B(OH)_3_, which has been effective in promoting oxidation in Chan–Lam amination reactions, also had no effect;^17^ however, addition of phen (Cu:phen = 1:2) and AcOH was effective at promoting the oxidation. The effect of AcOH is consistent with previous observations in Chan–Lam reactions.^15−17^ The dependence on a Cu:phen ratio of 1:2 for catalyst turnover was shown by the direct use of 10 mol % **4**, when a yield of 6% was obtained. Further investigation using stoichiometric **4** under an inert atmosphere resulted in a 51% yield, confirming reactivity, with catalytic turnover limited by an inability to undergo reoxidation. When an additional equivalent of phen was included (with respect to Cu loading), this enabled catalytic turnover, resulting in a 70% yield (Table S5). The effect of phen stoichiometry on oxidation was notable: oxidation of [Cu(phen)I] required an 18 h reflux, compared to [Cu(phen)_2_I], where oxidation was achieved within 20 mins at 70 °C.^30^ Heating to a minimum of 30 °C was also required for oxidation to occur (Figure S10), with a reaction yield of 9% (i.e., one catalytic cycle at 10 mol % catalyst) at room temperature and 70% at 30 °C (Table S2). Moreover, the EPR spectrum of the oxidation in the presence of phen was consistent with that of the proposed **[3]I** (Figure 3b).

**Figure 4:**
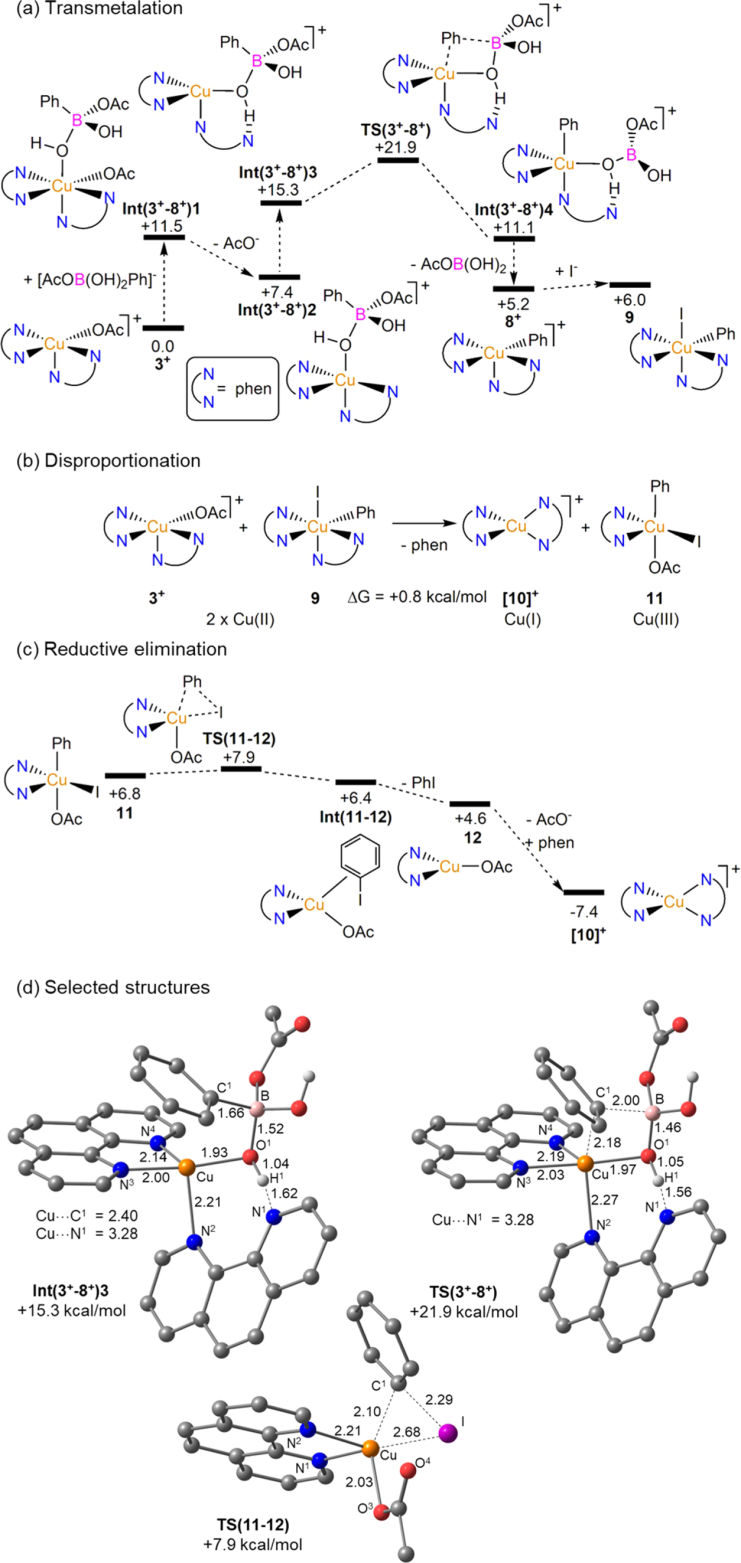
Computed reaction profiles (free energies, kcal/mol) for (a) the transmetalation step linking **[3]**^**+**^ to **[8]**^**+**^ and **9** with AcO^–^ as a base, (b) disproportionation of Cu(II) intermediates **[3]**^**+**^ and **9**, and (c) reductive elimination from Cu(III) intermediate **11**. (d) Selected computed geometries with key distances in Å and nonhydroxy H atoms are omitted for clarity. See SI Section 8 for alternative pathways considered.

The above data indicated that ligand speciation, specifically relating to phen stoichiometry, varied during the reaction and Cu required one or two phen for specific mechanistic events. To shed more light on this issue and to clarify the details of the iododeboronation reaction mechanism, we turned to DFT calculations (Figure 4).^31^

The geometry computed in solution for the [Cu(phen)_2_OAc]^+^ cation, **[3]**^**+**^, displays a distorted square-pyramidal geometry (τ = 0.28^32^) with an axial N-donor and a κ^1^-OAc ligand (Cu–O distances = 2.01 Å/2.70 Å), similar to the cation in the solid-state structure of **[3]OAc** (τ = 0.42, Cu–O = 2.00/2.64 Å^25^). A variety of alternative precursor species were also assessed: 6-coordinate Cu(phen)_2_(OAc)*_x_*(X)_2–*x*_ (X = I, OAc; *x* = 0–2), cationic [Cu(phen)_2_(X)(S)]^+^ (S = MeOH, H_2_O), and mono-phen 4-coordinate Cu(phen)(OAc)*_x_*(I)_2–*x*_ (see Tables S6 and S7). Of these, the most accessible was Cu(phen)(OAc)_2_ (Δ*G* = +3.1 kcal/mol), while OAc/I exchange in **[3]**^**+**^ to give 5-coordinate [Cu(phen)_2_I]^+^ (**[5]**^**+**^) was endergonic by 5.4 kcal/mol. The computed geometry of **[5]**^**+**^ is trigonal bipyramidal (τ = 0.90) in good agreement with the solid-state structure of **[5]I** (τ = 0.85). Similar speciation studies identified [Cu(phen)_2_Ph]^+^, **[8]**^**+**^, as the most stable Cu-aryl intermediate formed upon transmetalation (where Ph was used as the prototypical aryl group). **[8]**^**+**^ exhibits a distorted square-pyramidal geometry with an axial N-donor (τ = 0.37). Cu(phen)_2_(I)Ph, **9**, lies only 0.8 kcal/mol above **[8]**^**+**^, suggesting that it would be readily accessible in solution. Loss of a phen ligand from **9** to form Cu(phen)(I)(Ph) is disfavored (ΔG = +7.5 kcal/mol).

Having identified **[3]**^**+**^ and **[8]**^**+**^ as the most likely precursor and intermediate formed in the transmetalation step, we turned to the details of that process. The most accessible computed profile is shown in Figure 4a, where the acetate present in solution engages **1a** to deliver the cognate boronate.^33^ This first binds to **[3]**^**+**^ via a hydroxyl substituent to give **Int(3**^**+**^**-8**^**+**^**)1** at +11.5 kcal/mol. Dissociation of the Cu-bound acetate ligand then forms **Int(3**^**+**^**-8**^**+**^**)2** (+7.4 kcal/mol) from which the Cu–N^1^ phen arm decoordinates to form **Int(3**^**+**^**-8**^**+**^**)3** (+15.3 kcal/mol), which features a strong H-bond with the Cu-bound OH of the boronate (N^1^···H^1^ = 1.62 Å, see also Figure 4d for the computed structure and atom labeling). This places the Ph group adjacent to a vacant site at Cu (Cu···C^1^ = 2.40 Å) from which Ph group transfer can readily occur via **TS(3**^**+**^**-8**^**+**^**)** with an additional barrier of only 6.6 kcal/mol. All Cu–N bonds lengthen slightly in this transition state to accommodate the transferring phenyl group, while the N^1^···H^1^ shortens further to 1.56 Å. The initial Cu–phenyl intermediate, **Int(3**^**+**^**-8**^**+**^**)4** (+11.1 kcal/mol), retains the B(OH)_2_(OAc) side-product via Cu–O and OH···N interactions. Dissociation of this species with re-coordination of the free phen arm then forms **[8]**^**+**^ at +5.2 kcal/mol. Transmetalation therefore occurs with an accessible overall barrier of 21.9 kcal/mol but is endergonic by 5.2 kcal/mol. This is consistent with the nonobservation of any Cu-aryl species and low conversion to undesired products, when the reaction is performed in the absence of a halide source.^34^

Retention of the weakly bound κ^1^-*N*-phen ligand in **TS(3**^**+**^**-8**^**+**^**)** is important as in its absence, the overall barrier for an equivalent mono-phen pathway increases to 34.4 kcal/mol. Alternative transition states were also located in which MeOH or H_2_O solvent molecules act as H-bond acceptors to the boronate with similar overall computed barriers of 22.0 and 22.8 kcal/mol, respectively. Elongation of the Cu–N^1^ distance was far less marked in these cases (ca. 2.53 Å) and intrinsic reaction coordinate (IRC) calculations indicated the direct formation of **[8]**^**+**^ with expulsion of AcOB(OH)_2_ (i.e., no intermediate equivalent to **Int(3**^**+**^**-8**^**+**^**)4** was located). Other pathways, including those in which HO^–^ was considered as a base, proved higher in energy; in addition, transmetalation with PhBpin gave a higher overall barrier of 26.9 kcal/mol (see SI Section S8.3).

Once **[8]**^**+**^ is formed, iodide can readily add to give Cu(phen)_2_(Ph)(I), **9**, from which reductive elimination of PhI could in principle occur. However, this process would form a Cu(0) species and was shown to be thermodynamically inaccessible (Δ*G* = +18.1 kcal/mol). Instead, we propose that **9** undergoes disproportionation with a further equivalent of **[3]**^**+**^ to form Cu(I) species [Cu(phen)_2_]^+^, [**10]**^**+**^, and a Cu(III) species, Cu(phen)(I)(OAc)Ph, **11** (Figure 4b).

**11** is the lowest of four low-spin square-pyramidal Cu(III) isomers that all lie with 4 kcal/mol; all of these structures are at least 17 kcal/mol less stable when computed as a high-spin triplet. Ph–I bond forming reductive elimination from **11** then proceeds with a minimal barrier of 1.1 kcal/mol to form an initial η^2^-PhI complex at +6.4 kcal/mol (see Figure 4c).^35^ Dissociation of the PhI product forms Cu(phen)(OAc), **12**, at which AcO^–^/phen substitution forms [**10]**^**+**^ at −7.4 kcal/mol.

The DFT modeling studies indicate that bis-phen species are implicated both prior to (**[3]**^**+**^) and after (**[8]**^**+**^) the transmetalation step and that it is the disproportionation step that forms a mono-phen Cu(III) intermediate, **11**. Both the transmetalation and the disproportionation steps are slightly endergonic, but the kinetically facile Ph–I reductive elimination from **11** drives the reaction to completion once coupled with the thermodynamically favorable formation of [Cu(phen)_2_]^+^, **[10]**^**+**^. The AcOB(OH)_2_ side-product formed in the computed mechanism would be readily hydrolyzed under the reaction conditions releasing AcOH. AcOH facilitates the Cu(I) oxidative turnover step with O_2_ to re-form **[3]**^**+**^ (see Figure 3)^15−17^ releasing AcO^–^, which is then available for the boronate formation that enables the transmetalation step under catalytic turnover.

**Scheme 5:**
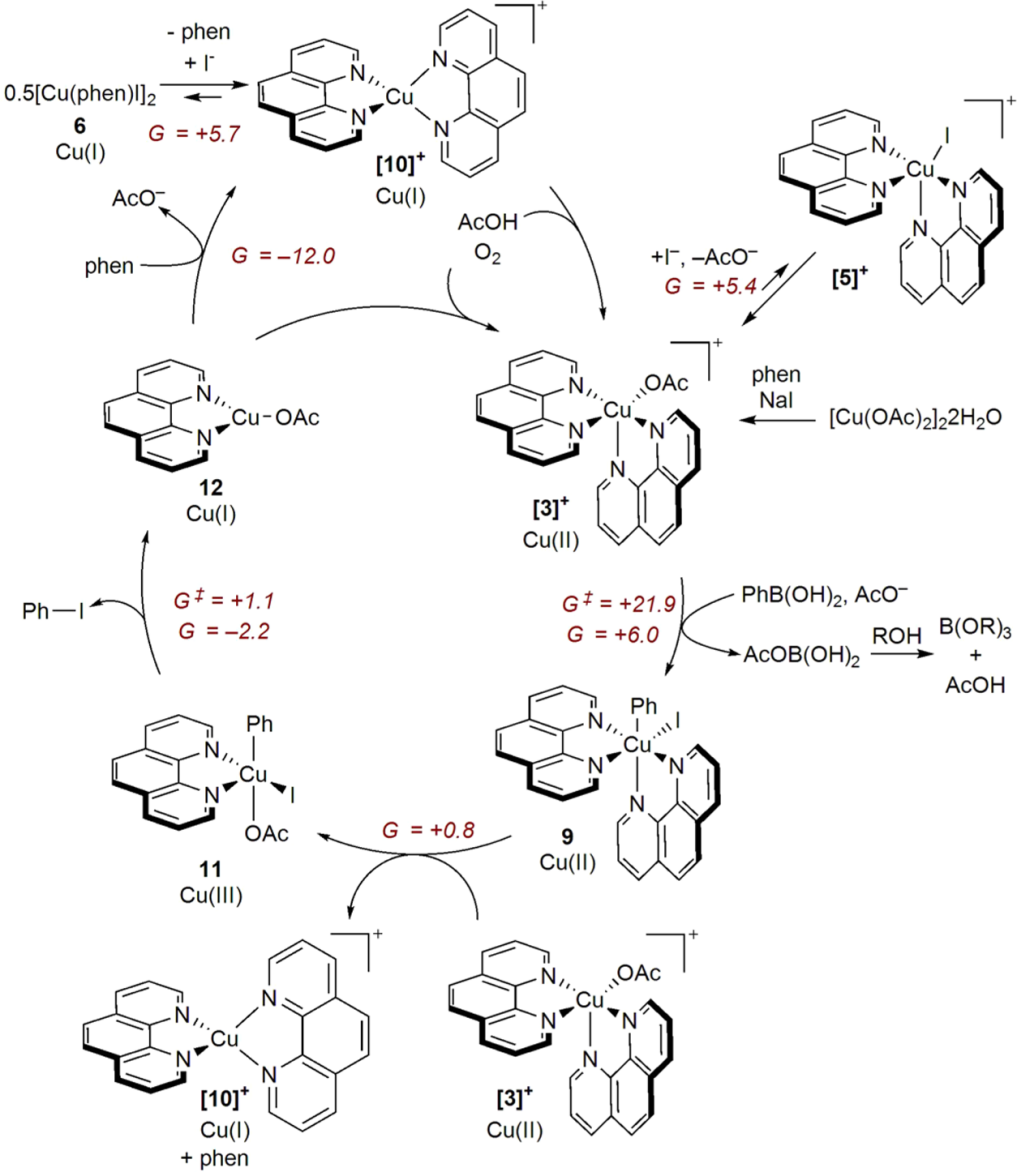
Proposed Catalytic Cycle with Computed Free Energy Changes and Activation Barriers (kcal/mol) Shown in Italics

Based on the totality of the dataset, an illustrative description of the proposed key events of the catalytic cycle is provided in Scheme 5.

[Cu(OAc)_2_]_2_·2H_2_O undergoes denucleation in the presence of phen and NaI to give **[3]**^**+**^. **[5]**^**+**^ can, in principle, be accessed from **[3]**^**+**^, but the equilibrium is negligible. AcO^–^ induces boronate formation from the arylboronic acid, facilitating transmetalation to give initially **[8]**^**+**^ that then adds I^–^ to form **9**. Disproportionation involving **9** and **[3]**^**+**^ gives **11** as the most stable Cu(III) species along with Cu(I) species **[10]**^**+**^ and so proceeds with loss of one phen ligand. **11** then undergoes reductive elimination, delivering the aryl iodide product and Cu(I) species **12**. AcO^–^/phen exchange at **12** reforms **[10]**^**+**^ which undergoes oxidative turnover by action of AcOH and O_2_ to close the catalytic cycle. The Cu(I) dimer **6** can also be formed via anion metathesis at **12**, although the formation of this species at 5.7 kcal/mol above **[10]**^**+**^ suggests that it is not directly implicated in catalysis.

In summary, a combination of experimental and computational investigations has provided the first complete description of the Cu-catalyzed iododeboronation reaction. This analysis has revealed the key role of the ligand in three critical steps—facilitating transmetalation via H-bonding to the boronate and promoting the key oxidative events (disproportionation and turnover). More specifically, ligand speciation is a key operation—ligand loss is favored at disproportionation, but gain is required at oxidative turnover, which provides a basis for understanding of ligand stoichiometry. From these data, future catalyst design principles can be constructed, with ligand stoichiometry crucial for catalytic turnover. This will be increasingly relevant in processes where turnover is a critical issue, such as the related fluorodeboronation reaction. This work therefore adds to the wider knowledge base of Cu-mediated oxidative coupling reactions and may support understanding, design, and development of related processes.

## Data Availability

The research data supporting this publication can be accessed at https://doi.org/10.17630/8603890e-da81-4225-9343-a3f5baba470c. Crystallographic data for compounds **[3]OAc**, **[3]Cl**, **4**, **5**, and **6** are available from the Cambridge Crystallographic Data Centre (CCDC) under Deposition Numbers 2258899–2258903.

## Supporting Information Available

Experimental procedures; characterization data; computational data; and X-ray data (PDF)
Combined cif (CIF)
Combined checkcif (PDF)
ESI geometries (XYZ)

## Supplementary Material

cs3c02839_si_001.pdf

cs3c02839_si_002.cif

cs3c02839_si_003.pdf

cs3c02839_si_004.xyz

## Abbreviations

Ac: acyl
Ar: aryl
IRC: intrinsic reaction coordinate
phen: 1,10-phenanthroline
Ph: phenyl
pin: pinacolato
