# Supplementary material for: Mechanism of Cu-Catalyzed Iododeboronation: A Description of Ligand-Enabled Transmetalation, Disproportionation, and Turnover in Cu-Mediated Oxidative Coupling Reactions
Source: ACS Catal. 2023 Aug 7;13(16):11117–26. doi: 10.1021/acscatal.3c02839 (PMC10442916; doi:10.1021/acscatal.3c02839)
Supplement: Supplementary file 1 — cs3c02839_si_001.pdf [file cs3c02839_si_001.pdf]

Mechanism of Cu-catalyzed iododeboronation: A description of ligand-enabled transmetalation, disproportionation, and turnover in Cu-mediated oxidative coupling reactions

Matthew J. Andrews,[a‡] Ambre Carpentier,[b‡] Alexandra M. Z. Slawin,[a] David B. Cordes,[a] Stuart A. Macgregor,[b]* and Allan J. B. Watson[a]*

[a]EaStCHEM, School of Chemistry, University of St Andrews, Purdie Building, St Andrews, KY16 9ST, U.K.

[b]Institute of Chemical Sciences, Heriot-Watt University, Edinburgh, EH14 4AS, U.K.

*Corresponding authors: s.a.macgregor@hw.ac.uk; aw260@st-andrews.ac.uk

Contents

1 General Information 2
2 Experimental Procedures 3
2.1 Synthesis of Complexes 3
2.2 Optimisation Reactions 6
2.3 General Procedure for Iodination 8
2.4 Catalyst Screen 9
3 UV-Vis Experiments 10
4 EPR Experiments 14
5 SCXRD Data 18
6 Control Reactions 23
7 NMR 25
7.1 Sequential Addition of Reagents 25
7.2 Reaction Omitting NaI 26
7.3 Stoichiometric Addition of Reagents Under Ar 27
8 Computational 29
8.1 Computational Details 29
8.2 Speciation of Cu(II) Active Species and Cu(II)(aryl) Complex 30
8.3 Alternative Transmetalation Mechanisms 33
8.4 Alternative Disproportionation Pathways 43
8.5 Cu(III) Isomers and Reductive Elimination 45
8.6 Functional Testing 49
8.7 Computed Geometries 50
References 127

1 General Information

All reagents and solvents were obtained from commercial suppliers and were used without further purification unless otherwise stated. Purification was carried out according to standard laboratory methods. Reactions were typically done in standard borosilicate glassware unless otherwise stated. Room temperature (RT) was generally *ca.* 20 °C. Reactions at elevated temperatures were obtained using a temperature-regulated hotplate/stirrer. Thin layer chromatography was carried out using Merck aluminium-backed silica plates coated with F_{254} fluorescent indicator, analysed under 254 nm UV light. Column chromatography was performed using silica gel (40 – 62 µm, Fluorochem).

^{1}H NMR spectroscopy was carried out using a Bruker AV 300 fitted with a BBFO probe, a Bruker AVIII-HD 500 equipped with BBFO+ probe, or a Bruker AVII400 equipped with BBFO probe. All ^{1}H spectra were recorded at RT with the deuterated solvents used as a lock for spectra and internal reference. ^{11}B spectra were externally referenced to $F_3B{\bullet}OEt_2$ in $CDCl_3$. Chemical shifts are reported in parts per million (ppm) to the nearest 0.01 ppm (^{1}H) or 0.1 ppm (^{11}B). NMR spectra are reported as follows: chemical shift/ppm (multiplicity, coupling constant(s), number of nuclei). Multiplicity given as br (broad), s (singlet), d (doublet), t (triplet), q (quartet), p (pentet), h (hextet), m (multiplet), and combinations thereof. Signals which overlap with one another are described as multiplets. UV-Vis spectra were recorded using a Jasco V650 UV-Vis double-beam spectrophotometer fitted with a USE-753 accessory and a D2/WI lightsource and the optical range used was 190 – 900 nm. The cell length was 1 mm and data was typically collected between 400 – 900 nm at a scan speed of 200 nm/min and a collection interval of 0.5 nm. Typical results storage was 1001 single measured values as a linear data array. EPR experiments were conducted on a Bruker EMX plus spectrometer controlled by a Bruker ER 083 CS microwave bridge operating at X-Band, microwave frequency of ≈9.9 GHz at microwave power of 20.70 mW, modulation amplitude of 10.0 G and a time constant of 40.96 s. The sweep centre was at 3425 G and a sweep width of 2000 G with 2000 points on X-axis. The samples were prepared in volumetric flasks with [Cu] (5 mM) dissolved in reaction solvent (MeOH:H_2O 4:1) and an aliquot drawn into a 20 µL micropipette. All spectra corrected against solvent baseline. Spectra were recorded at either RT or 100 K in the glassy state as specified.

Diffraction quality crystals were obtained and coated with paratone oil and mounted on the end of a nylon loop attached to the end of a goniometer. X-ray diffraction data for compound **3[OAc]** was collected at 173 K using a Rigaku SCXmini CCD diffractometer with a SHINE monochromator [Mo Kα radiation (λ = 0.71073 Å)]. Diffraction data for compounds **[3]Cl**, **4**, **5**, and **6** were collected at either 93 K (**[3]Cl**, **4**, and **6**) or 173 K (**5**) using a Rigaku FR-X Ultrahigh Brilliance Microfocus RA generator/confocal optics with XtaLAB P200 diffractometer [Mo Kα radiation (λ = 0.71073 Å)]. Intensity data for all compounds were collected using ω steps accumulating area detector images spanning at least a hemisphere of reciprocal space. Data for all compounds analysed were collected using CrystalClear[1] and processed (including correction for Lorentz, polarization, and absorption) using

either CrystalClear or CrysAlisPro.[2] Structures were solved by dual-space methods (SHELXT[3]) and refined by full-matrix least-squares against F^2 (SHELXL-2018/3[4]). Non-hydrogen atoms were refined anisotropically, and carbon-bound hydrogen atoms were refined using a riding model. Hydrogen atoms bound to oxygen were identified from the difference Fourier map and refined isotropically subject to a distance restraint, except for those in **6**, which were placed in calculated positions and refined using a riding model. All calculations were performed using either the CrystalStructure[5] or the Olex2[6] interface. Selected crystallographic data are presented in Section 4. CCDC 2258899-2258903 contains the supplementary crystallographic data for this paper. These data can be obtained free of charge from The Cambridge Crystallographic Data Centre via www.ccdc.cam.ac.uk/structures.

2 Experimental Procedures

2.1 Synthesis of Complexes

Complexes were synthesised according to literature procedures and confirmed through SCXRD and analysed via EPR spectroscopy.

Complex [3]OAc

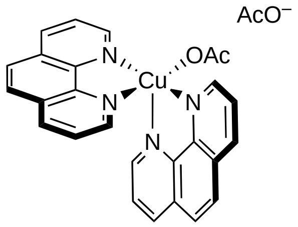

$[Cu(OAc)_2]_2 \cdot 2H_2O$ (1.0 equiv, 1.50 mmol, 0.299 g) was dissolved in MeOH (10 mL) and aq. KOH 1 M (2.0 equiv, 3.0 mmol, 3 mL) was added followed by 1,10-phenanthroline (2 equiv, 3.0 mmol, 0.541 g). The mixture was stirred overnight forming a dark green solution. This was evaporated to dryness under reduced pressure before acetone (10 mL) was added. This was concentrated under reduced pressure, before crystallization was induced through warming, resulting in the precipitation of dark blue crystals. These were isolated by filtration, washed with acetone, and dried in air to give the desired complex (0.391 g, 48% yield). Data were consistent with the literature.[7]

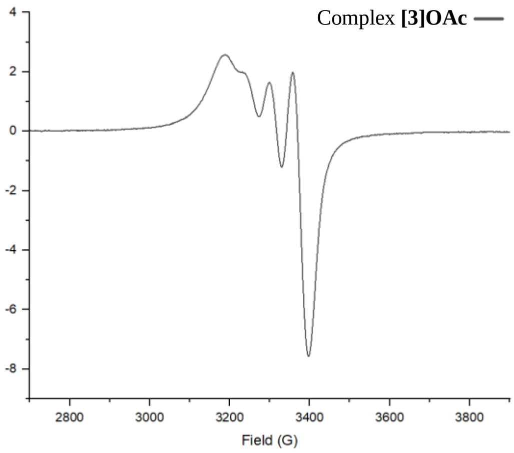

Figure S1. EPR spectrum of **[3]OAc** (5 mM) at RT.

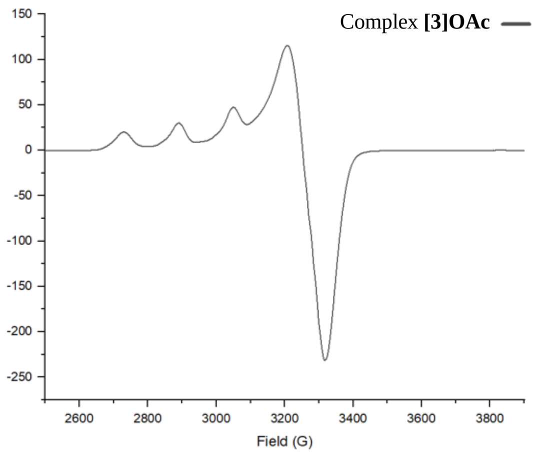

Figure S2. EPR spectrum of **[3]OAc** (5 mM) in glassy state at 100 K.

Complex 4

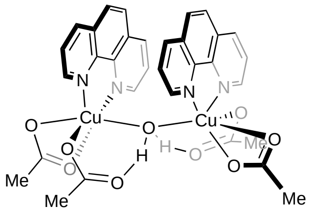

To a solution of 1,10-phenanthroline (2.2 equiv, 2.75 mmol, 0.496 g) in EtOH (25 mL) was added $[Cu(OAc)_2]_2 \cdot 2H_2O$ (1.0 equiv, 1.25 mmol, 0.250 g) and the resultant blue solution was refluxed for one hour under air. The volume of the mixture was then reduced under reduced pressure to *ca.* 2.5 mL before acetone (10 mL) was added. The flask was then gently warmed at 40 °C, causing formation of a pale blue precipitate, which was isolated by filtration, washed with acetone, and dried in air to give the desired complex (0.279 g, 60% yield). Data were consistent with the literature.[8]

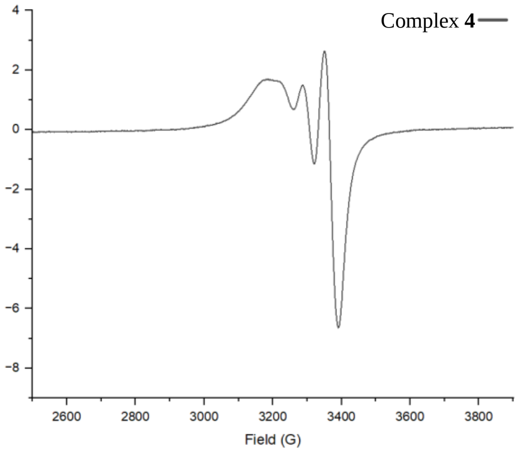

Figure S3. EPR spectrum of **4** (5 mM) at RT.

Complex [3]Cl

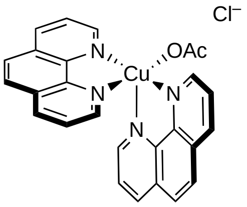

A crude reaction mixture from the synthesis of complex **[3]OAc** (see above) was dissolved in CH_2Cl_2 and left overnight to slowly evaporate, resulting in the formation of dark green crystals, which were isolated by filtration.

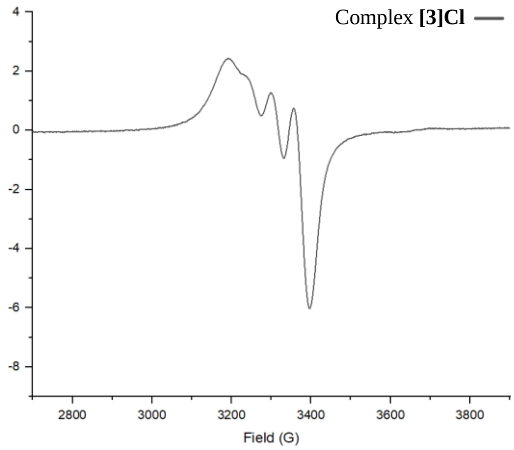

Figure S4. EPR spectrum of **[3]Cl** (5 mM) at RT.

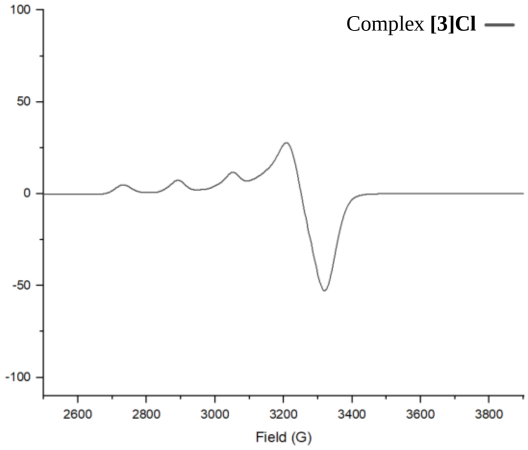

Figure S5. EPR spectrum of **[3]Cl** (5 mM) in glassy state at 100 K.

Complex [5]I

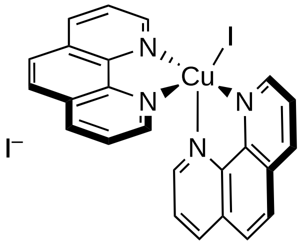

$[Cu(OAc)_2]_2 \bullet 2H_2O$ (1.0 equiv, 0.5 mmol, 0.100 g) was dissolved in MeOH (5 mL). 1,10-Phenanthroline (2.0 equiv, 1.0 mmol, 0.180 g) and NaI (2.0 equiv, 1.0 mmol, 0.150 g) were added and the resultant mixture was heated to 60 °C for 20 mins. Concentration under reduced pressure delivered a dark red/black precipitate, which was isolated by filtration and washed with acetone to afford the desired complex (0.210 g, 62% yield). Crystals suitable for SCXRD were obtained through the slow evaporation of the reaction mixture in a 2 mL vial. Data were consistent with the literature.[9]

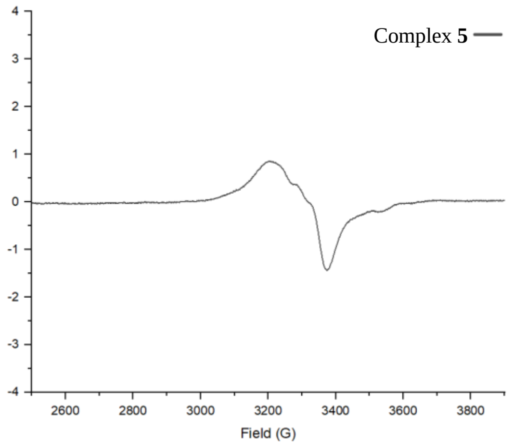

Figure S6. EPR spectrum of **5[I]** (5 mM) at RT.

Complex 6

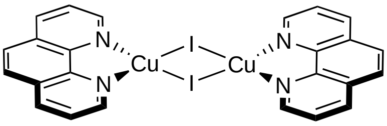

A solution of 1,10-phenanthroline (1.0 equiv, 1.0 mmol, 0.180 g,) in MeCN (5 mL) was added to a suspension of CuI (1.0 mmol, 0.190 g) in MeCN (5 mL), resulting in the precipitation of a brick red precipitate (0.267 g, 72% yield).[10] Crystals suitable for SCXRD were obtained from a J. Young's NMR tube following reaction conducted under Ar atmosphere, outlined in **Section 7.2**.

2.2 Optimisation Reactions

4-Methoxybenzeneboronic acid (1.0 equiv, 200 µmol, 30.4 mg), $[Cu(OAc)_2]_2$•$2H_2O$ (10 mol%, 20.0 µmol, 4.00 mg), 1,10-phenanthroline (20 mol%, 40 µmol, 10.8 mg,) and NaI (2.2 equiv, 440 µmol, 66.0 mg) were dissolved in MeOH:H_2O (4:1; 7.5 mL). This was heated to 70 °C for 2 hours exposed to air, before sodium thiosulfate aq. soln. (0.8 mL) was added. H_2O (5 mL) was then added, and the product extracted with EtOAc (10 mL followed by 2×5 mL), washed with brine (5 mL), dried over Na_2SO_4, and solvent removed under rotatory evaporation. Methyl *tert*-butyl ether (MTBE) (1.0 equiv, 200 µmol, 24 µL) was then added as an internal standard and a crude NMR was taken to determine NMR yield.

Note: Temperature was monitored using an internal probe within a control flask filled with deionised water on the same hotplate.

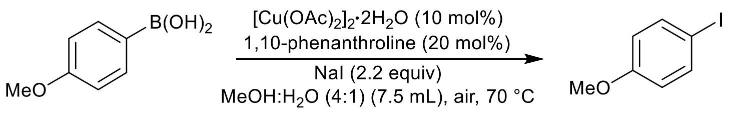

(0.2 mmol)
1a **2**

Table S1. Time study at 70 °C.

Time (h)	NMR Yield (%)
2	61
3	78
18	63

Table S2. Temperature study at 2 h reaction time.

Temperature (°C)	NMR Yield (%)
RT (20)	9
30	70
40	95
50	97
60	63
70[a]	54
80	10
90[b]	7

[a]Trichloroethene (24 µL, 1.3 equiv) used as internal standard. [b]Reaction at 90 °C carried out using 20 mol% [Cu], 40 mol% 1,10-phenanthroline.

Table S3. Temperature study at 1 h reaction time.

Temperature (°C)	NMR Yield (%)
30	73
40	72
50	87
60	82

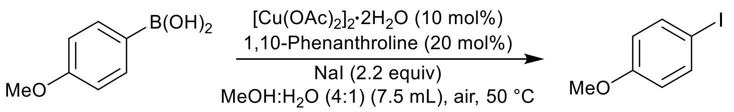

(0.2 mmol)

1a **2**

Table S4. Time study at 50 °C.

Time (min)	NMR Yield (%)
60	87
40	82
20	60

2.3 General Procedure for Iodination

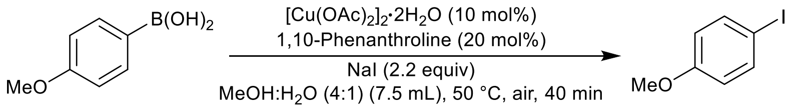

(0.2 mmol)

1a **2**

A mixture of 4-methoxybenzeneboronic acid (1.0 equiv, 200 µmol, 30.4 mg), $[Cu(OAc)_2]_2$ •$2H_2O$ (10 mol%, 20.0 µmol, 4.00 mg, 1,10-phenanthroline (20 mol%, 40.0 µmol, 10.8 mg), and NaI (2.2 equiv, 440 µmol, 66.0 mg) in MeOH:H_2O (4:1; 7.5 mL) was heated to 50 °C for 40 mins in an open flask. The mixture was allowed to cool to RT and sodium thiosulfate aq. soln. (0.8 mL) was added followed by H_2O (5 mL). The mixture was extracted with EtOAc (10 mL followed by 2×5 mL), and the combined organic extracts were washed with brine (5 mL), dried over Na_2SO_4, and concentrated under reduced pressure. MTBE (1.0 equiv, 200 µmol, 24 µL) was added as an internal standard before analysis by ^{1}H NMR. The spectroscopic data matches a commercial sample.

^{1}H NMR (400 MHz, $CDCl_3$) δ 7.56 (d, *J* = 9.0 Hz, 2H), 6.68 (d, *J* = 9.0 Hz, 2H), 3.78 (s, 3H).

2.4 Catalyst Screen

Table S5. Catalyst screen using the general procedure in **Section 2.3.**

**Complex**	**NMR Yield (%)**
$[Cu(OAc)_2]_2 \cdot 2H_2O$ (10 mol%) + 1,10-phenanthroline (10 mol%)	9
$[Cu(OAc)_2]_2 \cdot 2H_2O$ (10 mol%) + 1,10-phenanthroline (20 mol%)	97
$[Cu(OAc)_2]_2 \cdot 2H_2O$ (10 mol%) + 1,10-phenanthroline (30 mol%)	Quant.
Compound **[3]OAc**	80
Compound **4**	6
Compound **4** (1 equiv) under Ar	51
Compound **4** + 1,10-phenanthroline (10 mol%)	70
Compound **5**	83
Compound **[3]Cl**	67
Compound **6**	8
Compound **6** (1 equiv) under Ar	Trace
Compound **6** + 1,10-phenanthroline (10 mol%)	76
Compound **6** + NaOAc (10 mol%)	5
Compound **6** + 1,10-phenanthroline (10 mol%)+ NaOAc (10 mol%)	70

3 UV-Vis Experiments

$[Cu(OAc)_2]_2 \cdot 2H_2O$, compounds **[3]Cl**, **[3]OAc**, and **4** were analysed by UV-vis spectroscopy for characterisation of the d-d transition band using the following procedure:

[Cu] (10 µmol) was dissolved in $MeOH:H_2O$ (4:1; 2 mL) and the absorbance spectra measured from a 1 mL cuvette between 500 – 900 nm alongside a baseline solvent control.

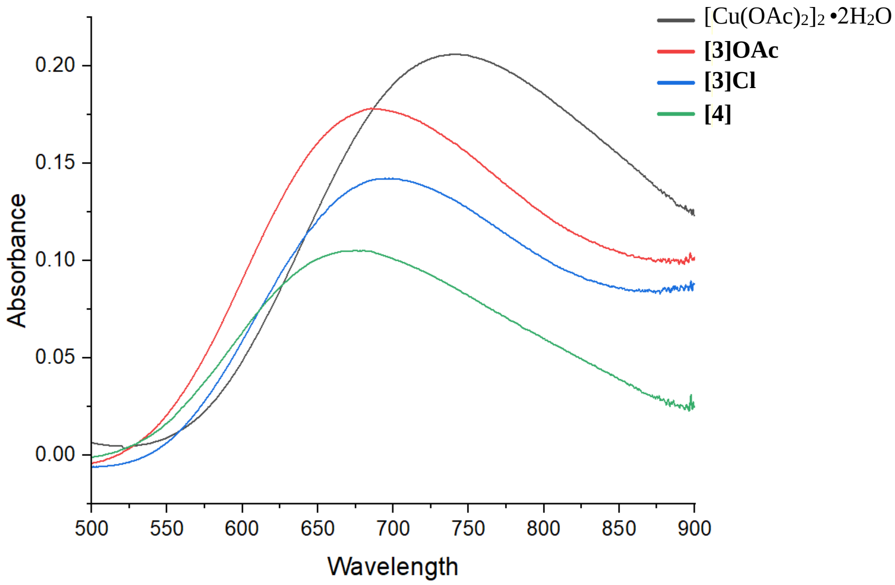

Figure S7. Overlaid UV-Vis spectra (500 – 900 nm region) of $[Cu(OAc)_2]_2 \cdot 2H_2O$, **[3]OAc**, **[3]Cl**, and **[4]** using the procedure detailed above.

The oxidation of CuOAc was studied through UV-Vis spectroscopy for the appearance of the characteristic d-d transition band at *ca.* 680 nm using the following procedure:

A 10 mL glass vial was charged with CuOAc (1.0 equiv, 10 µmol, 1.2 mg), additive (1.0 equiv), and $MeOH:H_2O$ (4:1; 2 mL). The mixture was then stirred at 60 °C for 20 minutes before being filtered through cotton wool. The UV-Vis absorbance spectra were then recorded from a 1 mL cuvette between 500 – 900 nm alongside a baseline solvent control.

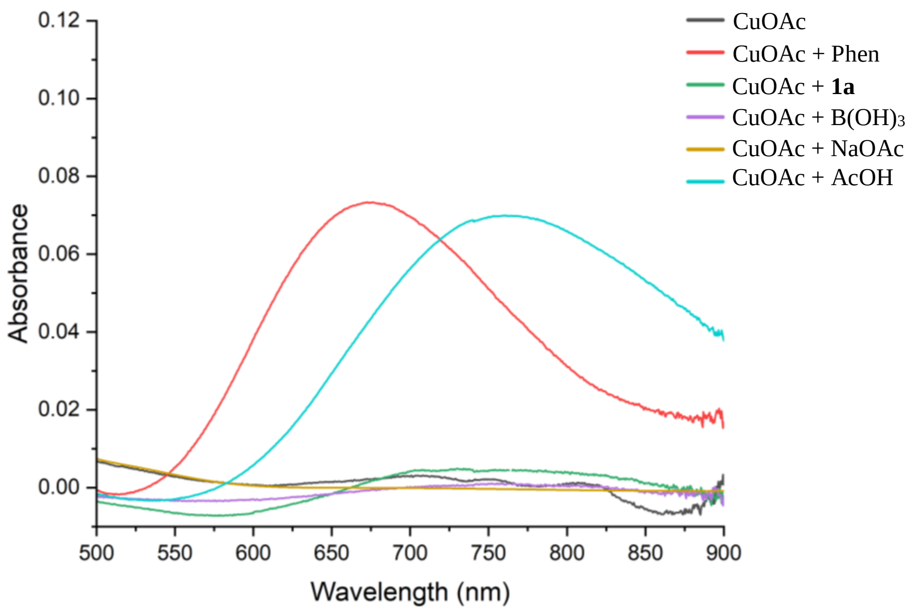

Figure S8. Overlaid UV-Vis spectra (500 – 900 nm region) studying the oxidation of CuOAc with various reaction components using the procedure detailed above.

The effect of combinations of additives was assessed using the following procedure:

A 10 mL glass vial was charged with CuOAc (1.0 equiv, 10 µmol, 1.2 mg), 1,10-phenanthroline (1.0 equiv, 10 µmol, 1.8 mg), additive (1.0 equiv), and MeOH:H_2O (4:1; 2 mL). The mixture was stirred at 60 °C under air for 20 minutes before being filtered through cotton wool. The UV-Vis absorbance spectra were then recorded from a 1 mL cuvette between 500 – 900 nm alongside a baseline solvent control.

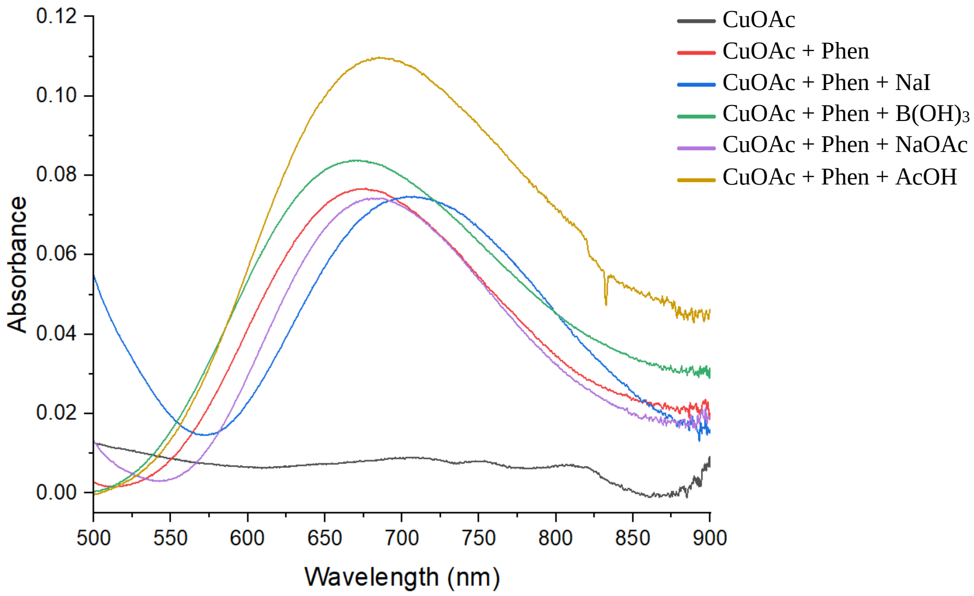

Figure S9. Overlaid UV-Vis spectra (500 – 900 nm region) studying the oxidation of CuOAc with various reaction components using the procedure detailed above.

The effect of temperature was assessed using the following procedure:

A 10 mL glass vial was charged with CuOAc (1.0 equiv, 10 µmol, 1.2 mg), 1,10-phenanthroline (1.0 equiv, 10 µmol, 1.8 mg) and MeOH:H_2O (4:1; 2 mL). The mixture was stirred at different temperatures under air for 20 minutes before being filtered through cotton wool. The UV-Vis absorbance spectra were then recorded from a 1 mL cuvette between 500 – 900 nm alongside a baseline solvent control.

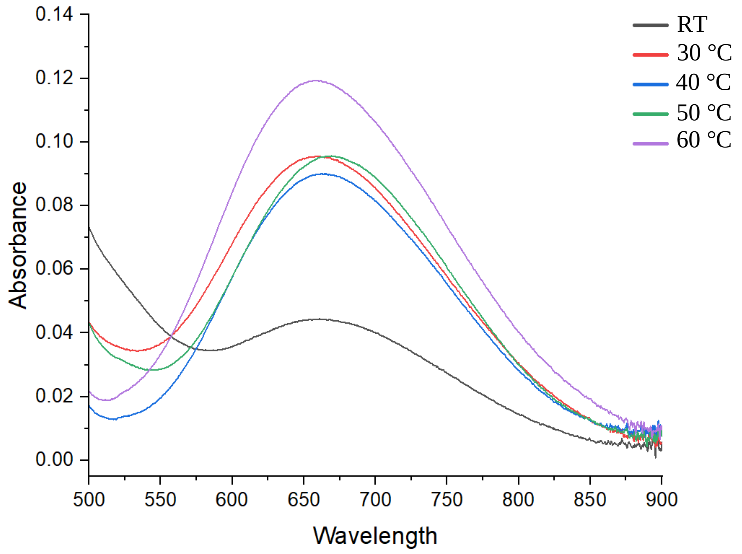

Figure S10. Overlaid UV-Vis spectra (500 – 900 nm region) studying the oxidation of CuOAc in the presence of phenanthroline at different temperatures using the procedure detailed above.

Control experiments were conducted at RT to deconvolute the binding of 1,10-phenanthroline with the oxidation, with spectra obtained at 200 – 400 nm showing similar shifts to that of compound **[4],** indicating that a Cu(I)/phen complex forms at RT prior to oxidation.

[Cu] (1.0 equiv, 10 µmol) and/or 1,10-phenanthroline (1.0 equiv, 10 µmol, 1.8 mg) was dissolved in MeOH:H_2O (4:1; 2 mL) and stirred at RT before the absorbance spectra measured from a 1 mL cuvette between 200 – 400 nm alongside a baseline solvent control, requiring dilution with MeOH:H_2O (4:1) until the spectra would not max out the detector.

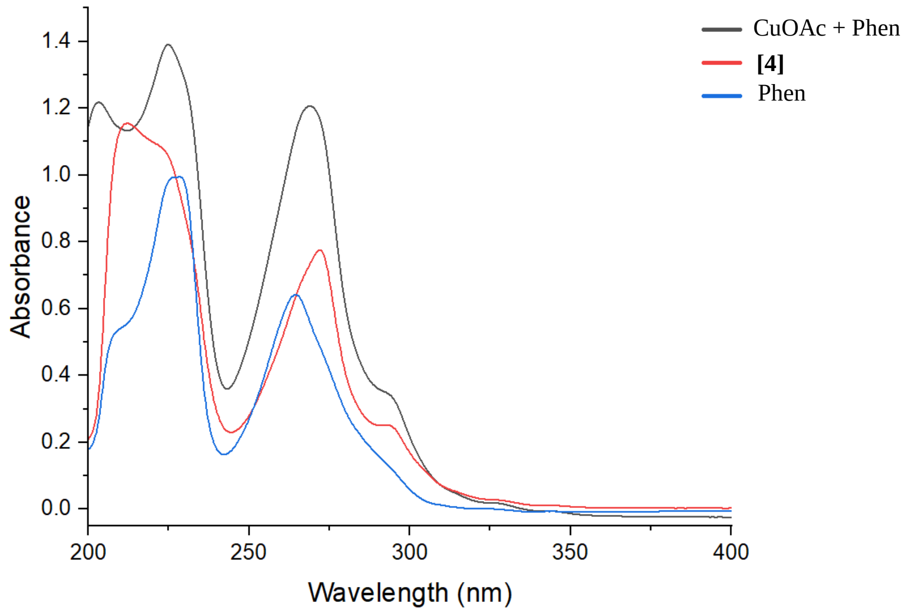

Figure S11. Overlaid UV-Vis spectra (200 – 400 nm region) studying the binding of 1,10-phenanthroline at RT using the procedure detailed above.

A comparison of the effect of ligand on the oxidation of CuOAc was investigated through comparing 1,10-phenanthroline to pyridine (a poor ligand for the catalytic process) which showed the importance of phen to the Cu(I) oxidation.

A 10 mL glass vial was charged with CuOAc (1.0 equiv, 10 µmol, 1.2 mg), either 1,10-phenanthroline (1.0 equiv, 10 µmol, 1.8 mg) or pyridine (1.0 equiv, 10 µmol, 0.8 µL), and MeOH:H_2O (4:1; 2 mL). The mixture was stirred under air at 60 °C for 20 minutes before being filtered through cotton wool. The UV-Vis absorbance spectra were then recorded from a 1 mL cuvette between 500 – 900 nm alongside a baseline solvent control.

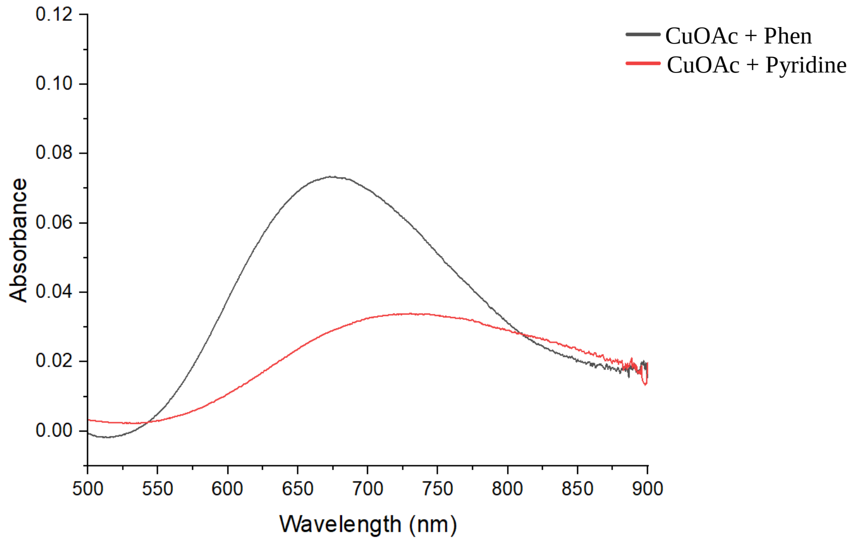

Figure S12. Overlaid UV-Vis spectra (500 – 900 nm region) comparing oxidation of CuOAc in the presence of 1,10-phenanthroline or pyridine using the procedure detailed above.

4 EPR Experiments

NaI (2.0 equiv, 10 µmol, 1.4 mg) and $[Cu(OAc)_2]_2 \bullet 2H_2O$ (1.0 equiv, 5 µmol, 0.9 mg, 5 mM) were dissolved in MeOH:H_2O (4:1; 1 mL) and stirred under air for five minutes at RT before being measured as outlined in the general information.

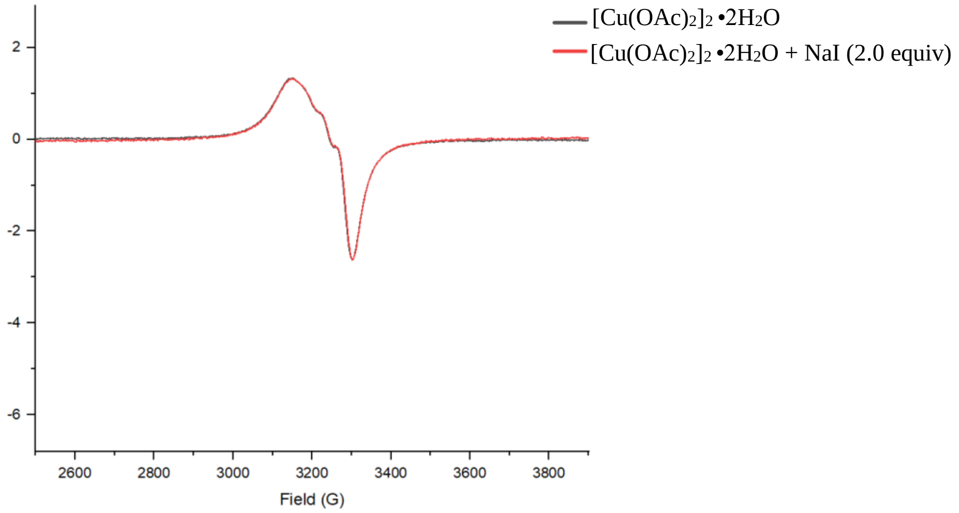

Figure S13. Overlaid EPR spectra showing no change to $[Cu(OAc)_2]_2 \bullet 2H_2O$ (black) by the addition of NaI (red) using the procedure detailed above.

1,10-Phenanthroline (2.0 equiv, 10 µmol, 1.8 mg) and $[Cu(OAc)_2]_2 \bullet 2H_2O$ (1.0 equiv, 5 µmol, 0.9 mg, 5 mM) were dissolved in MeOH:H_2O (4:1; 1 mL) and stirred at RT for five minutes before being measured as outlined in the general information.

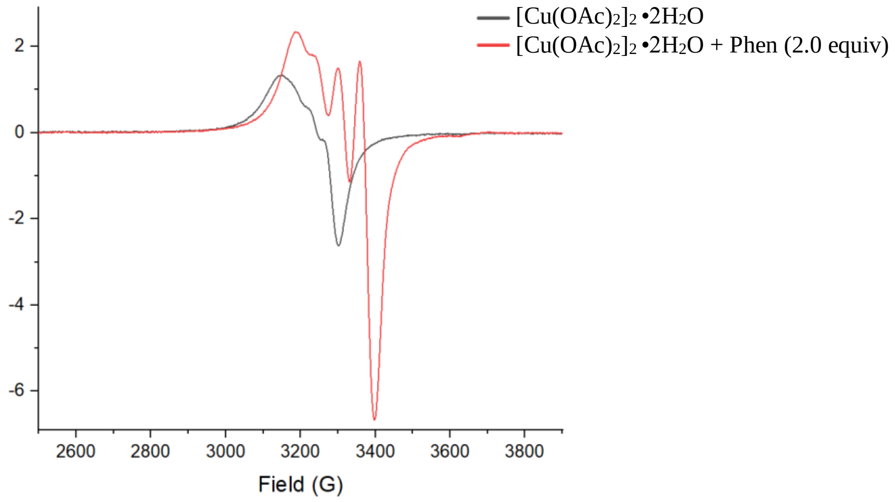

Figure S14. Overlaid EPR spectra showing the binding of 1,10-phenanthroline to $[Cu(OAc)_2]_2 \bullet 2H_2O$ using the procedure detailed above.

[$Cu(OAc)_2]_2$ •$2H_2O$ (1.0 equiv, 5 µmol, 0.9 mg, 5 mM) and 1,10-phenanthroline (2.0 equiv, 10 µmol, 1.8 mg) were dissolved in $MeOH:H_2O$ (4:1; 1 mL) and stirred under air at RT for five minutes before being measured as outlined in the general information (red) or with further addition prior to mixing of NaI (2.0 equiv, 10 µmol, 1.4 mg) (blue) or compound **1a** (1.0 equiv, 10 µmol, 0.8 mg) (black).

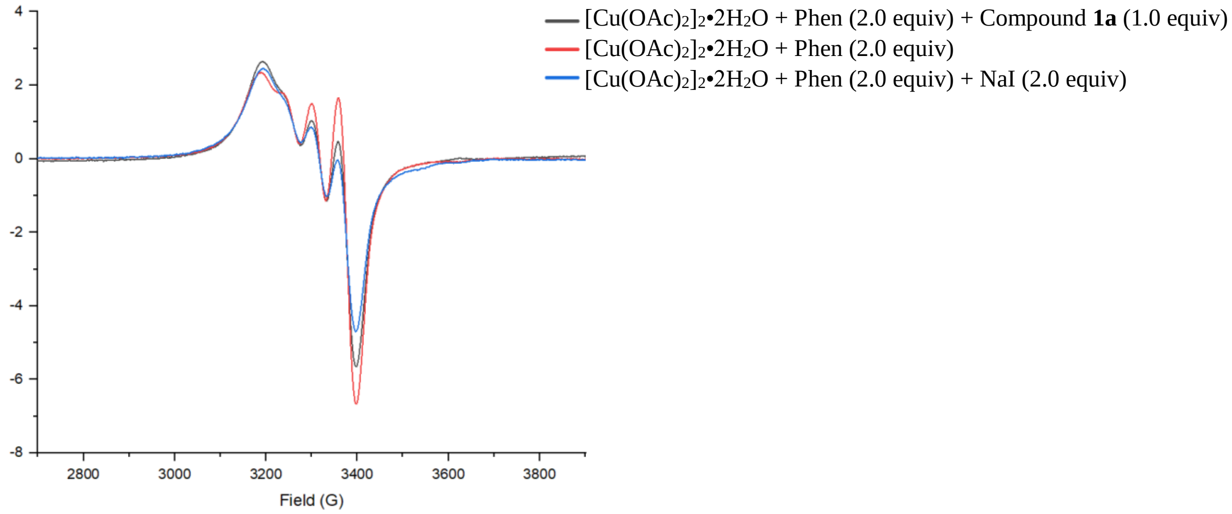

Figure S15. Overlaid EPR spectra of [$Cu(OAc)_2]_2$ •$2H_2O$ and Phen complex (red) formed in situ with NaI (blue) or compound **1a** (black) added accordingly using the procedure detailed above.

Compound **3[OAc]** (1.0 equiv, 5 µmol, 2.7 mg, 5 mM) and NaI (2.0 equiv, 10 µmol, 1.4 mg) were dissolved in $MeOH:H_2O$ (4:1; 1 mL) and stirred at 60 °C for 20 minutes before being measured as outlined in the general information.

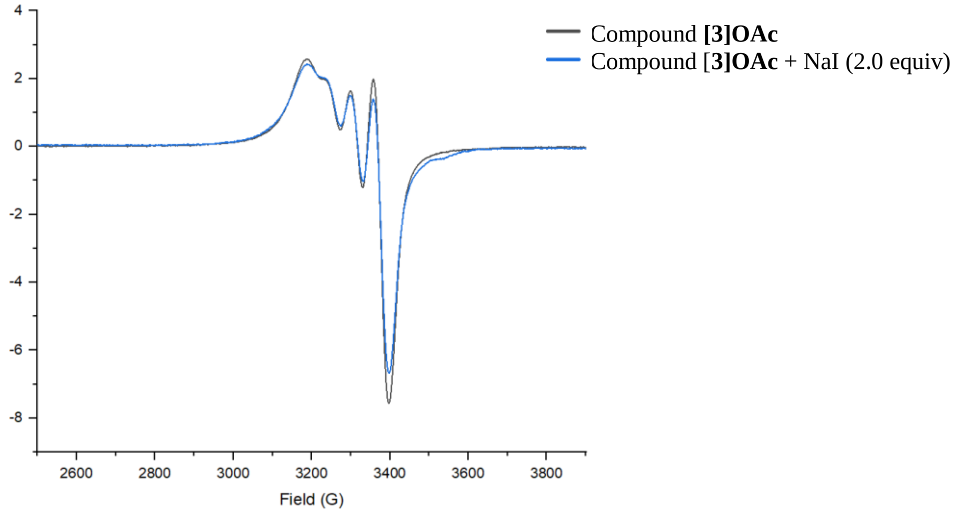

Figure S16. Overlaid EPR spectra of compound **[3]OAc** (black) and after NaI addition (blue) using the procedure detailed above.

Compound **4** (1.0 equiv, 5 µmol, 1.8 mg, 5 mM) and NaI (2.0 equiv, 10 µmol, 1.4 mg) were dissolved in MeOH:H_2O (4:1; 1 mL) and stirred under air at 60 °C for 20 minutes before being measured as outlined in the general information.

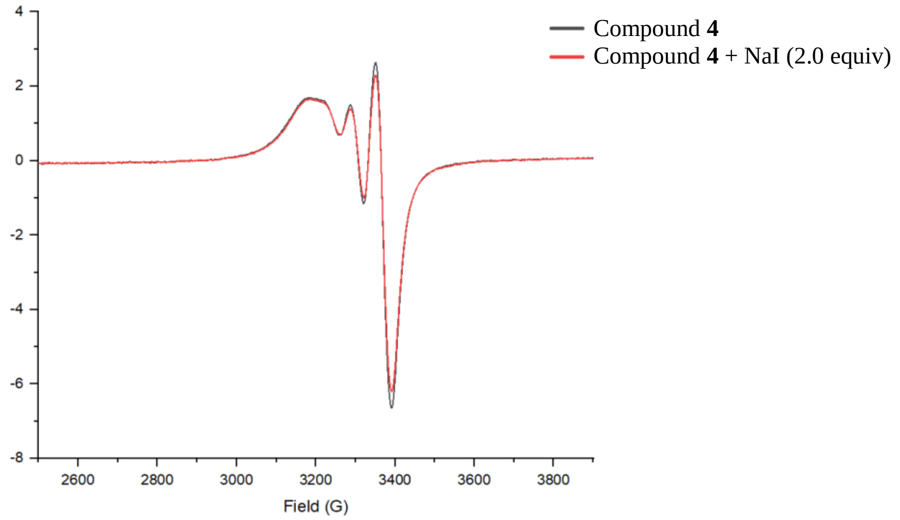

Figure S17. Overlaid EPR spectra of compound **4** (black) and after NaI addition (red) using the procedure detailed above.

Compound **3[Cl]** (1.0 equiv, 5 µmol, 2.6 mg, 5 mM) and NaI (2.0 equiv, 10 µmol, 1.4 mg) were dissolved in MeOH:H_2O (4:1; 1 mL) and stirred at 60 °C for 20 minutes before being measured as outlined in the general information.

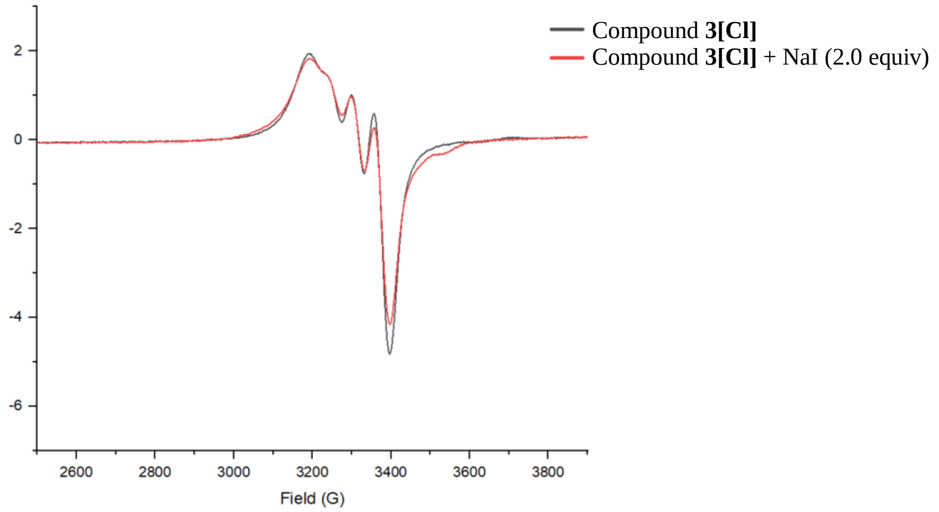

Figure S18. Overlaid EPR spectra of compound **3[Cl]** (black) and after NaI addition (red) using the procedure detailed above.

Overlaid EPR spectra showing the oxidation of CuOAc (1.0 equiv, 5 µmol, 0.6 mg, 5 mM) (blue) to Cu(II) through the addition of 1,10-phenanthroline (2.0 equiv, 10 µmol, 1.8 mg) (black), and oxidation with subsequent complexation of NaI (2.0 equiv, 10 µmol, 1.4 mg) (red), dissolved in MeOH:H_2O

(4:1; 1 mL) and stirred under air at 60 °C for 20 minutes before being measured as outlined in the general information.

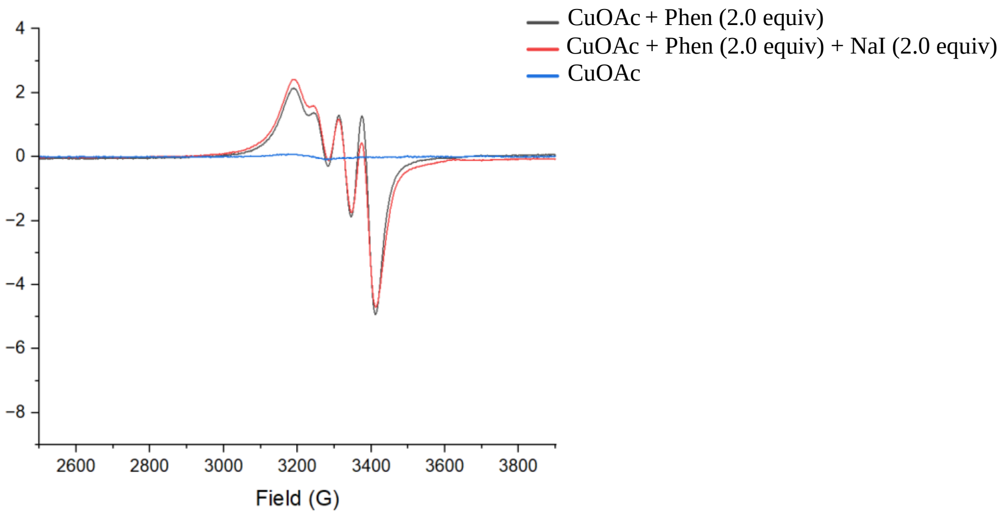

Figure S19. Overlaid EPR spectra showing the oxidation of CuOAc to Cu^{II} through the addition of 1,10-phenanthroline (black) and oxidation and subsequent complexation with NaI (red) using the procedure detailed above.

5 SCXRD Data

Complex [3]OAc

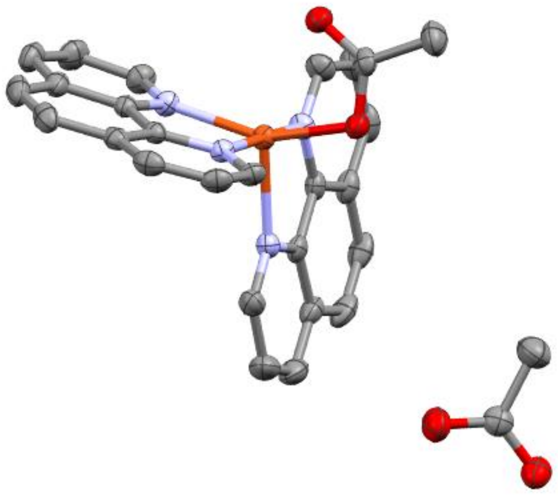

Figure S20. Asymmetric unit cell of complex **[3]OAc** with 50% ellipsoids. Hydrogen atoms and solvent have been omitted for clarity. Cu; Orange.

Identification code	$[Cu(Phen)_2(OAc)](OAc)$
CCDC number	2258899
Empirical formula	$C_{28}H_{36}CuN_4O_{11}$
Formula weight	668.16
Temperature/K	173
Crystal system	triclinic
Space group	P-1
a/Å	8.7325(6)
b/Å	12.2149(8)
c/Å	15.6708(11)
α/°	103.318(5)
β/°	102.145(5)
γ/°	97.555(5)
Volume/Å^3	1561.49(19)
Z	2
ρ_{calc}g/cm^3	1.421
μ/mm^{-1}	0.763
F(000)	698.0
Crystal size/mm^3	0.22 × 0.20 × 0.17
Radiation	Mo Kα (λ = 0.71073)
2Θ range for data collection/°	3.828 to 55.046
Index ranges	$-11 \leq h \leq 11$, $-15 \leq k \leq 15$, $-20 \leq l \leq 20$
Reflections collected	16178
Independent reflections	7138 [R_{int} = 0.0471, R_{sigma} = 0.0603]
Data/restraints/parameters	7138/14/441
Goodness-of-fit on F^2	0.989
Final R indexes [I>=2σ (I)]	R_1 = 0.0390
Final R indexes [all data]	wR_2 = 0.1024
Largest diff. peak/hole / e Å^{-3}	0.38/-0.63

Complex 4

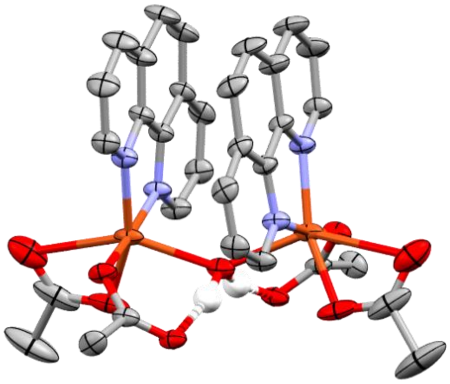

Figure S21. Asymmetric unit cell of complex **4** with 50% ellipsoids. Non-relevant hydrogen atoms and solvent have been omitted for clarity. Cu; Orange.

Identification code	$[Cu(Phen)(OAc)_2$
CCDC number	2258900
Empirical formula	$C_{32}H_{32}Cu_2N_4O_{10}$
Formula weight	741.70
Temperature/K	93.15
Crystal system	monoclinic
Space group	C2/c
a/Å	17.9655(10)
b/Å	9.431(4)
c/Å	18.835(11)
α/°	90.0000
β/°	100.04(2)
γ/°	90.0000
Volume/Å^3	3142(2)
Z	4
ρ_{calc}g/cm^3	1.568
μ/mm^{-1}	1.415
F(000)	1520.0
Crystal size/mm^3	0.12 × 0.12 × 0.12
Radiation	Mo Kα (λ = 0.71073)
2Θ range for data collection/°	4.392 to 50.752
Index ranges	$-20 \leq h \leq 21$, $-11 \leq k \leq 11$, $-22 \leq l \leq 19$
Reflections collected	18289
Independent reflections	2882 [R_{int} = 0.0315, R_{sigma} = 0.0178]
Data/restraints/parameters	2882/1/219
Goodness-of-fit on F^2	1.109
Final R indexes [I>=2σ (I)]	R_1 = 0.0393
Final R indexes [all data]	wR_2 = 0.1192
Largest diff. peak/hole / e Å^{-3}	2.00/-0.49

Complex [3]Cl

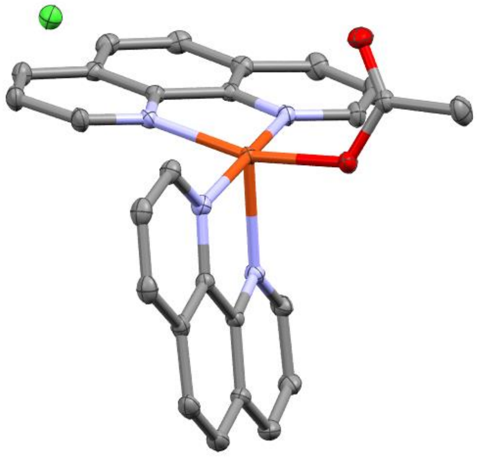

Figure S22. Asymmetric unit cell of complex **[3]Cl** with 50% ellipsoids. Hydrogen atoms and solvent have been omitted for clarity. Cu; Orange.

Identification code	$[Cu(Phen)_2(OAc)](Cl)$
CCDC number	2258901
Empirical formula	$C_{28}H_{27}CuN_4O_4Cl_5$
Formula weight	724.36
Temperature/K	93
Crystal system	monoclinic
Space group	C2/c
a/Å	22.0731(3)
b/Å	12.05400(14)
c/Å	21.9959(2)
α/°	90.0000
β/°	93.9717(10)
γ/°	90.0000
Volume/Å^3	5838.38(12)
Z	8
ρ_{calc}g/cm^3	1.648
μ/mm^{-1}	1.249
F(000)	2952.0
Crystal size/mm^3	0.10 × 0.10 × 0.10
Radiation	Mo Kα (λ = 0.71073)
2Θ range for data collection/°	4.22 to 73.552
Index ranges	-36 ≤ h ≤ 36, -19 ≤ k ≤ 19, -35 ≤ l ≤ 36
Reflections collected	63109
Independent reflections	13953 [R_{int} = 0.0282, R_{sigma} = 0.0163]
Data/restraints/parameters	13953/5/393
Goodness-of-fit on F^2	1.057
Final R indexes [I>=2σ (I)]	R_1 = 0.0321
Final R indexes [all data]	wR_2 = 0.1053
Largest diff. peak/hole / e Å^{-3}	1.01/-1.71

Complex 5

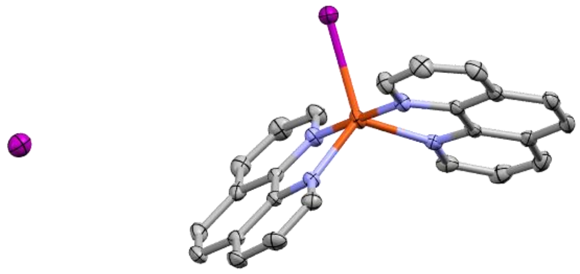

Figure S23. Asymmetric unit cell of complex **5** with 50% ellipsoids. Hydrogen atoms and solvent have been omitted for clarity. Cu; Orange.

Identification code	$[Cu(Phen)_2I]I$
CCDC number	2258902
Empirical formula	$C_{24}H_{18}CuI_2N_4O$
Formula weight	695.76
Temperature/K	173.15
Crystal system	triclinic
Space group	P-1
a/Å	10.1076(3)
b/Å	11.5890(3)
c/Å	12.0092(3)
α/°	66.113(2)
β/°	65.541(3)
γ/°	72.032(2)
Volume/Å^3	1153.92(6)
Z	2
ρ_{calc}g/cm^3	2.0024
μ/mm^{-1}	3.646
F(000)	666
Crystal size/mm^3	0.19 × 0.03 × 0.01
Radiation	Mo Kα (λ = 0.71073)
2Θ range for data collection/°	3.9 to 58.28
Index ranges	$-13 \le h \le 13$, $-15 \le k \le 15$, $-15 \le l \le 15$
Reflections collected	15150
Independent reflections	5085 [R_{int} = 0.0210, R_{sigma} = 0.0350]
Data/restraints/parameters	5085/2/297
Goodness-of-fit on F^2	1.077
Final R indexes [I>=2σ (I)]	R_1 = 0.0254
Final R indexes [all data]	wR_2 = 0.0670
Largest diff. peak/hole / e Å^{-3}	0.90/-0.28

Complex 6

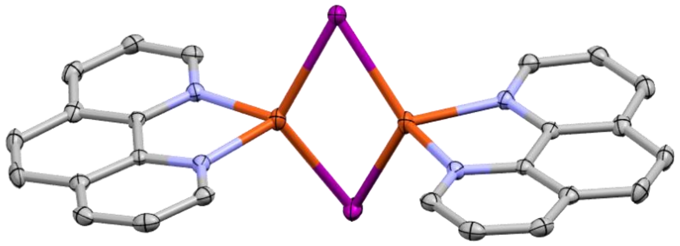

Figure S24. Asymmetric unit cell of complex **6** with 50% ellipsoids. Hydrogen atoms and solvent have been omitted for clarity. Cu; Orange.

Identification code	$[Cu(Phen)I]_2$
CCDC number	2258903
Empirical formula	$C_{25}H_{20}Cu_2I_2N_4O$
Formula weight	773.36
Temperature/K	93
Crystal system	orthorhombic
Space group	P*nma*
a/Å	14.387(3)
b/Å	17.196(3)
c/Å	9.9809(19)
α/°	90.0000
β/°	90.0000
γ/°	90.0000
Volume/Å^3	2469.3(8)
Z	4
ρ_{calc}g/cm^3	2.080
μ/mm^{-1}	4.250
F(000)	1480.0
Crystal size/mm^3	0.03 × 0.03 × 0.02
Radiation	Mo Kα (λ = 0.71073)
2Θ range for data collection/°	6.866 to 50.762
Index ranges	$-16 \leq h \leq 17$, $-20 \leq k \leq 20$, $-12 \leq l \leq 11$
Reflections collected	16479
Independent reflections	2325 [R_{int} = 0.0326, R_{sigma} = 0.0169]
Data/restraints/parameters	2325/0/162
Goodness-of-fit on F^2	1.063
Final R indexes [I>=2σ (I)]	R_1 = 0.0239
Final R indexes [all data]	wR_2 = 0.0621
Largest diff. peak/hole / e Å^{-3}	2.12/-1.10

6 Control Reactions

Assessing potential *ipso*-substitution pathway

To a solution of $[Cu(OAc)_2]_2•2H_2O$ (0.5 equiv, 16.5 µmol, 3.0 mg), 1,10-phenanthroline (1.0 equiv, 32.9 µmol, 5.9 mg) and NaI (2.2 equiv, 72.4 µmol, 10.9 mg) dissolved in MeOD:D_2O (0.5 mL) in an NMR tube was added anisole (1.0 equiv, 32.9 µmol, 3.6 µL). The NMR tube was then heated at 65 °C whilst exposed to air and the reaction monitored over 18 hours, during which time no change was observed.

2-Methoxyphenyl boronic acid (1.0 equiv, 0.2 mmol, 30.4 mg), $[Cu(OAc)_2]_2•2H_2O$ (10 mol%, 20 µmol, 4.0 mg), 1,10-phenanthroline (20 mol%, 40 µmol, 7.2 mg), and NaI (2.2 equiv, 0.44 mmol, 66.0 mg) were dissolved in MeOH:H_2O (4:1; 5 mL). This was heated to 80 °C for 18 hours exposed to air, before sodium thiosulfate aq. soln. (0.8 mL) was added, brine (5 mL) added and the organic components extracted with EtOAc (10 mL followed by 2×5 mL), washed with brine (5 mL), dried over $MgSO_4$, and solvent removed under rotary evaporation. A crude NMR was then carried out to determine the yield based on an added internal standard of MTBE (91% yield of the expected 2-substituted product). The spectroscopic data matches commercial sample.

^{1}H NMR; (400 MHz, $CDCl_3$) δ 7.74 (dd, *J* = 7.8, 1.6 Hz, 1H), 7.33 (td, *J* = 7.4, 1.6 Hz, 1H), 6.95 (dd, *J* = 8.3, 1.3 Hz, 1H), 6.70 (td, *J* = 7.6, 1.3 Hz, 1H) 3.85 (s, 3H)

3-Methoxyphenyl boronic acid (1.0 equiv, 0.2 mmol, 30.4 mg), $[Cu(OAc)_2]_2•2H_2O$ (10 mol%, 20 µmol, 4.0 mg), 1,10-phenanthroline (20 mol%, 40 µmol, 7.2 mg), and NaI (2.2 equiv, 0.44 mmol, 66.0 mg) were dissolved in MeOH:H_2O (4:1; 5 mL). This was heated to 80 °C for 18 hours exposed to air, before sodium thiosulfate aq. soln. (0.8 mL) was added, brine (5 mL) added and the organic components extracted with EtOAc (10 mL followed by 2×5 mL), washed with brine (5 mL), dried over $MgSO_4$, and solvent removed under rotary evaporation. A crude NMR was then carried out to determine the yield based on an added internal standard of MTBE (78% yield of the expected 3-substituted product). The spectroscopic data matches commercial sample.

^{1}H NMR; (400 MHz, $CDCl_3$) δ 7.33 – 7.19 (m, 2H), 7.02 (t, *J* = 8.3 Hz, 1H), 6.96 – 6.87 (m, 1H), 3.77 (s, 3H)

Assessing impact of added pinacol on reaction efficiency

4-Methoxyphenyl boronic acid (1.0 equiv, 0.2 mmol, 30.4 mg), $[Cu(OAc)_2]_2•2H_2O$ (10 mol%, 20 µmol, 4.0 mg), 1,10-phenanthroline (20 mol%, 40 µmol, 7.2 mg), 2,3-dimethylbutane-2,3-diol (1.0 equiv, 0.2 mmol, 23.6 mg), and NaI (2.2 equiv, 0.44 mmol, 66.0 mg) were dissolved in MeOH:H_2O (4:1; 5 mL). This was heated to 50 °C for 40 mins exposed to air, before sodium thiosulfate aq. soln. (0.8 mL) was added, brine (5 mL) added and the organic components extracted with EtOAc (10 mL followed by 2×5 mL), washed with brine (5 mL), dried over $MgSO_4$, and solvent removed under rotary

evaporation. A crude NMR was then carried out to determine the yield based of an added internal standard of MTBE (65% yield, compared to 97% yield without the addition of pinacol).

Assessing the impact of the pinacol boronic ester derivative on reaction efficiency

4-Methoxyphenyl boronic acid pinacol ester (1.0 equiv, 0.1 mmol, 23.4 mg), $[Cu(OAc)_2]_2 \cdot 2H_2O$ (10 mol%, 10 µmol, 2.0 mg), 1,10-phenanthroline (20 mol%, 20 µmol, 3.6 mg), and NaI (2.2 equiv, 0.22 mmol, 33.0 mg) were dissolved in MeOH:H_2O (4:1; 2.5 mL). This was heated to 50 °C for 40 mins exposed to air, before sodium thiosulfate aq. soln. (0.8 mL) was added, brine (5 mL) added and the organic components extracted with EtOAc (10 mL followed by 2×5 mL), washed with brine (5 mL), dried over $MgSO_4$, and solvent removed under rotary evaporation. A crude NMR was then carried out to determine the yield based of an added internal standard of MTBE (83% yield, compared to 97% yield with boronic acid).

7 NMR

7.1 Sequential Addition of Reagents

Addition of NaI prior to boronic acid

$[Cu(OAc)_2]_2$•$2H_2O$ (0.5 equiv, 16.5 µmol, 3.0 mg), 1,10-phenanthroline (1.0 equiv, 32.9 µmol, 5.9 mg) and 1,4-dinitrobenzene (0.2 equiv, 6.6 µmol, 1.1 mg) were dissolved in MeOD:D_2O (4:1; 0.5 mL) (1). To this, components were added sequentially; NaI (2.2 equiv, 72.4 µmol, 10.9 mg) (2), 4-methoxyphenylboronic acid (1.0 equiv, 32.9 µmol, 5.0 mg) (3) and the reaction was heated at 65 °C initially for an hour (4) before being left overnight at 65 °C (5).

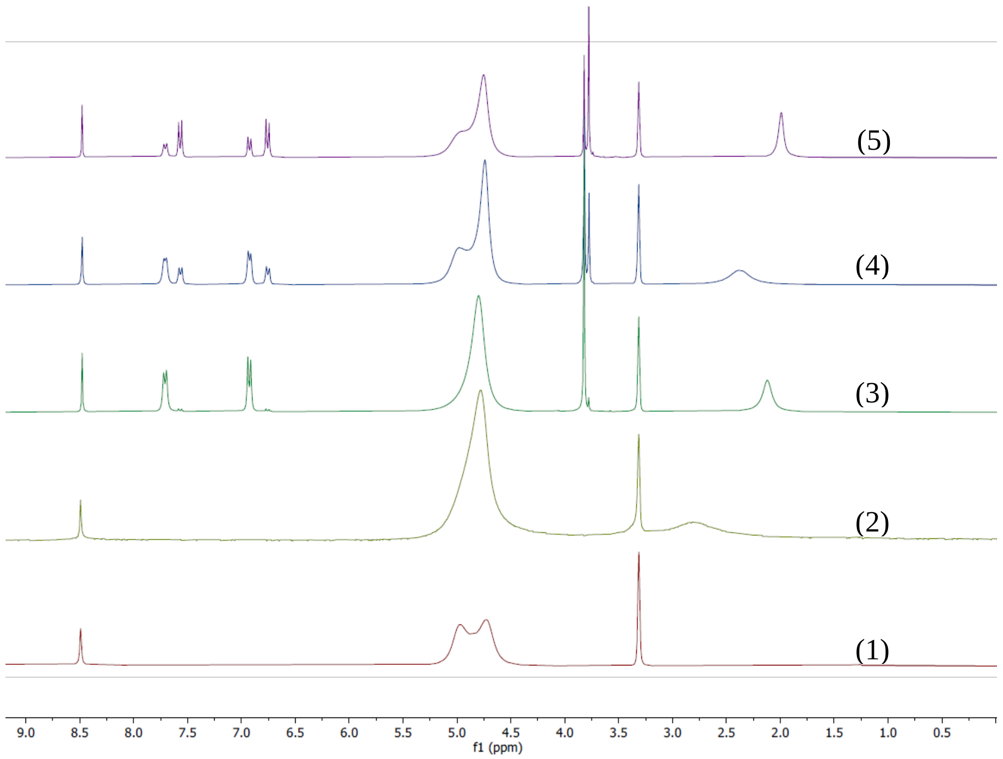

Figure S25. Overlaid NMR spectra of sequential reagent addition with NaI added prior to boronic acid.

Addition of the boronic acid prior to NaI

$[Cu(OAc)_2]_2$•$2H_2O$ (0.5 equiv, 16.5 µmol, 3.0 mg), 1,10-phenanthroline (1.0 equiv, 32.9 µmol, 5.9 mg) and 1,4-dinitrobenzene (0.2 equiv, 6.6 µmol, 1.1 mg) dissolved in MeOD:D_2O (4:1; 0.5 mL) (1). To this, components were added sequentially; 4-methoxyphenylboronic acid (1.0 equiv, 32.9 µmol, 5.0 mg) (2), and the reaction was heated to 65 °C for an hour (3) before NaI (2.2 equiv, 72.4 µmol, 10.9 mg) (4) was added, after which there was no further change upon continued heating.

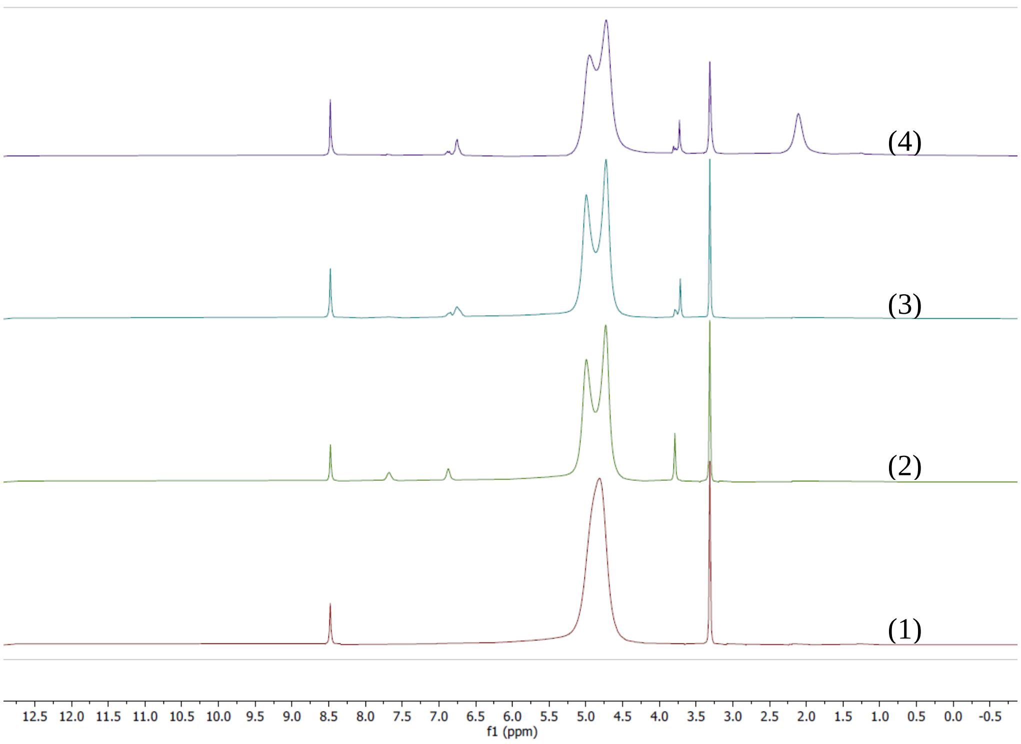

Figure S26. Overlaid NMR spectra of sequential reagent addition with boronic acid added prior to NaI.

7.2 Reaction Omitting NaI

The general procedure was followed except for the omission of NaI. This was then analysed through ^1H NMR to determine side reactions which could occur.

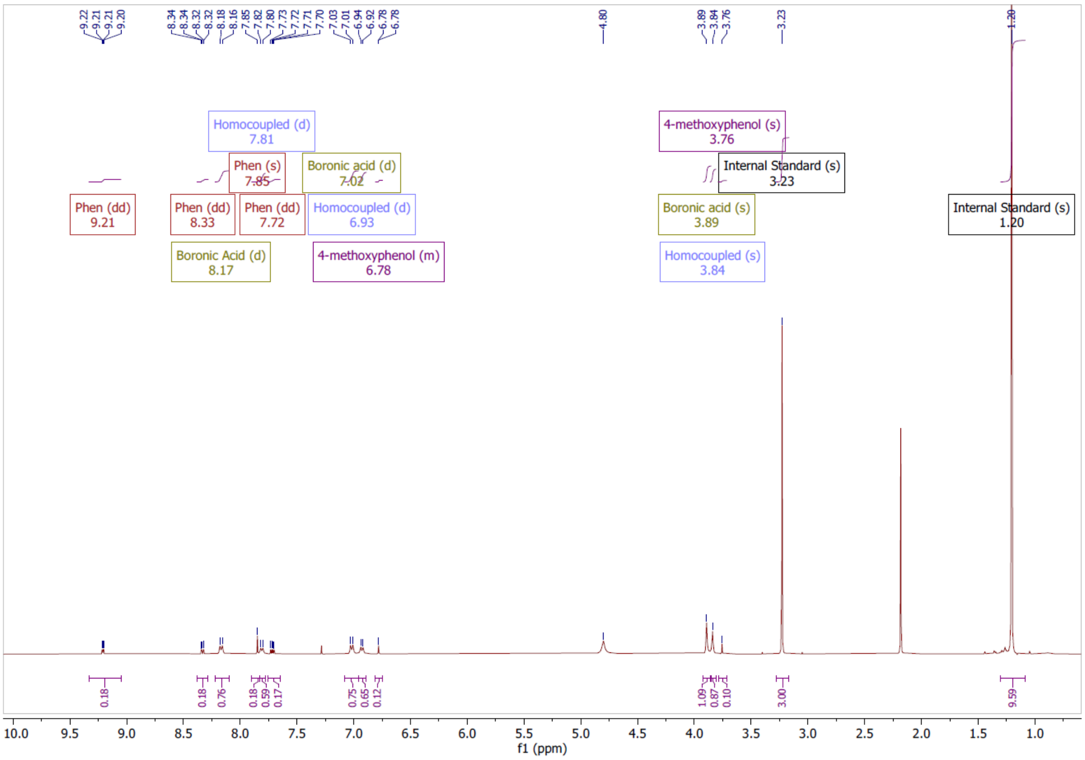

Chemical Shift (ppm)	Integration	Ratio	Inference
9.18	0.18	2 H	Phen (9%)
8.31	0.18	2 H	Phen (9%)
8.13	0.76	2 H	Boronic acid (**1a**) (38%)
7.82	0.18	2 H	Phen (9%)
7.78	0.59	4 H	Homocoupled (15%)
7.69	0.17	2 H	Phen (9%)
7.00	0.73	2 H	Boronic acid (**1a**) (37%)
6.90	0.65	4 H	Homocoupled (16%)
6.76	0.12	4 H	4-methoxyphenol (3%)
3.87	1.09	3 H	Boronic acid (**1a**) (36%)
3.81	0.87	6 H	Homocoupled (15%)
3.73	0.10	3 H	4-methoxyphenol (3%)
3.20	3 H		Internal Standard

Figure S27. ^{1}H NMR spectra from worked-up crude reaction mixture with MTBE (1.0 equiv, 200 µmol, 24 µL) was added as an internal standard for relative abundance. Peak assignment was carried out through a combination of comparison with commercial samples (1,10-phenanthroline, **1a**, 4-methoxyphenol) or through comparison to literature in combination with relative integration between aromatic and OMe regions of unassigned peaks. Homocoupled product refers to 4,4-dimethoxybiphenyl. Internal standard refers to MTBE.

7.3 Stoichiometric Addition of Reagents Under Ar

Under Ar, $[Cu(OAc)_2]_2 \cdot 2H_2O$ (0.5 equiv, 16.5 µmol, 3.0 mg), 1,10-phenanthroline (1.0 equiv, 32.9 µmol, 5.9 mg) and 1,4-dinitrobenzene (0.2 equiv, 6.6 µmol, 1.1 mg) dissolved in degassed MeOD:D_2O (4:1; 0.5 mL) and NaI (2.2 equiv, 72.4 µmol, 10.9 mg) and 4-methoxyphenylboronic acid

(1.0 equiv, 32.9 µmol, 5.0 mg) were added to a J. Young's NMR tube and heated to 65 °C for 15 minutes (1) before the reaction was further heated at 65 °C overnight (2). A second dose of NaI (2.2 equiv, 72.4 µmol, 10.9 mg) and 4-methoxyphenylboronic acid (1.0 equiv, 32.9 µmol, 5.0 mg) were added under Ar (3) and no further catalysis was observed despite heating at 65 °C overnight (4). At this point, the sample was then exposed to air, restarting the catalysis (5).

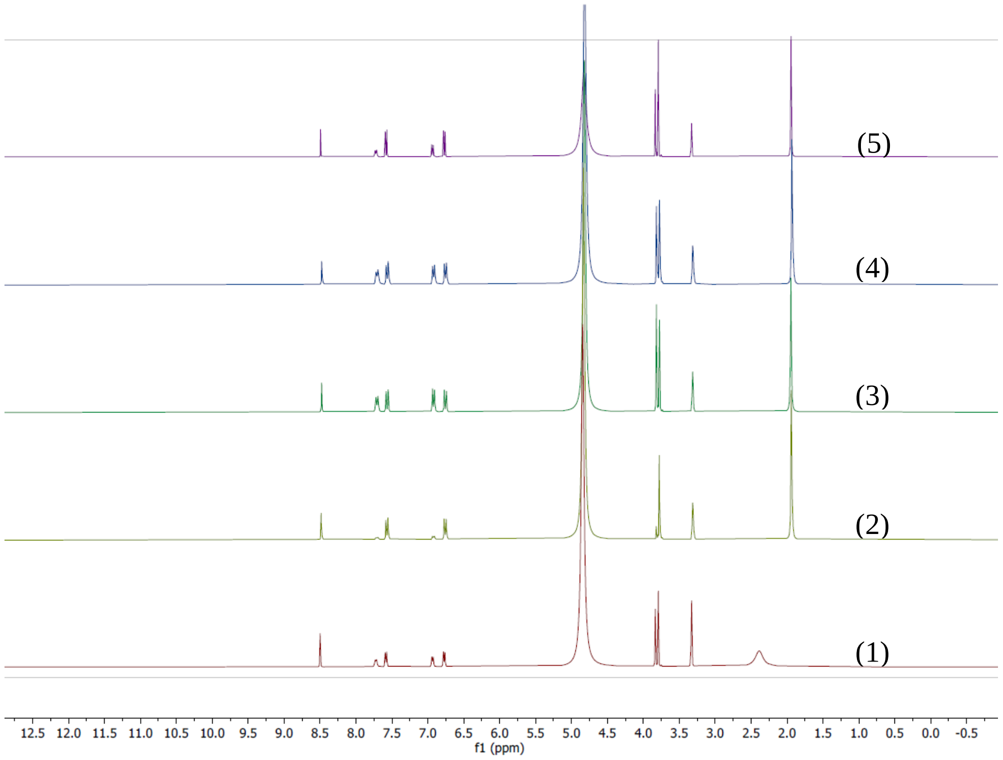

Figure S28. Overlaid NMR spectra of sequential reagent addition carried out under inert conditions.

8 Computational

8.1 Computational Details

All geometry optimizations were computed using Gaussian 16 (Revision A.03)[12] and the BP86 functional.[13,14] Cu, I and Cl centres were described with the Stuttgart RECPs and corresponding basis set.[15] d-orbital polarisation (ζ = 0.289 , ζ = 0.640) were added for I and Cl respectively.[16] 6-31G(d,p) basis sets were used to describe all other centres.[17] Methanol solvent was included in the optimisation protocol modelled by the Polarizable Continuum Model (PCM) and its corresponding dielectric constant (ε = 32.613).[18] Analytical frequency calculations were carried out to confirm minima as displaying no imaginary frequency. Transition states all present one negative frequency and were validated by running IRCs and subsequent optimizations, connecting them to adjacent minima. Final free energies were recovered after corrections including the triple-ζ basis set Def2-TZVP[19] and dispersion using the D3BJ method.[20]

8.2 Speciation of Cu(II) Active Species and Cu(II)(aryl) Complex

Table S6: Computed relative energies for the range of different candidates of possible Cu(II) active species, focussing on a description of the coordination, geometry and stability compared to the active species proposed experimentally **(3^+)**. For species containing hydroxo ligands, energies include OH^- formation via deprotonation of water by acetate. Cu-O interactions were considered as bound up to 2.07 Å, this being 5% over the sum of the published covalent radii.[21] A κ^2 coordination mode was considered for ΔO < 0.4 (where ΔO is the difference in Cu-O distances) and Cu-O_2 < 2.4 Å.

Name	*Compound*	*Coordination Geometry*	*ΔG relative to CuPhen₂OAc (kcal/mol)*
**$[Cu(Phen)_2(OAc)]^+$ (3^+)**	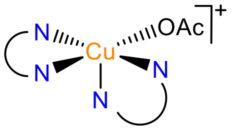	5<br>τ=0.28[1]	0.0
**$[Cu(Phen)_2(OH)]^+$**	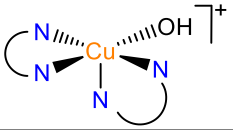	5<br>τ=0.18	+14.9
**$[Cu(Phen)_2(OAc)_2]$**	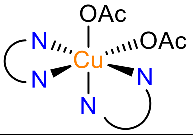	6<br>Jahn-Teller distorted O_h	+6.7
**$[Cu(Phen)_2(OH)_2]$**	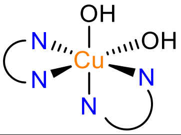	6<br>Jahn-Teller distorted O_h	+33.3
**$[Cu(Phen)_2(OAc)(OH)]$**	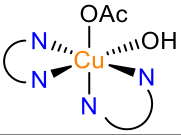	6<br>Jahn-Teller distorted O_h	+19.9
**$[Cu(Phen)(OH)_2]$**	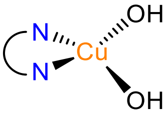	4<br>D_{2d}, θ=1.4° [2]	+28.1
**$[Cu(Phen)(OAc)_2]$**	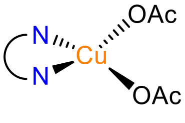	4<br>D_{2d}, q=15.4°<br>ΔO^1=0.56<br>ΔO^2=0.57	+3.1
**$[Cu(Phen)(OAc)(OH)]$**	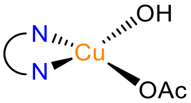	4<br>D_{2d}, θ=22.6°<br>ΔO=0.54	+17.9
**$[Cu(Phen)(OAc)]^+$**	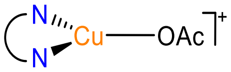	3<br>Trigonal Planar	+30.9
**$[Cu(Phen)(OH)]^+$**	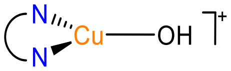	3<br>Trigonal Planar	+36.8
**$[Cu(Phen)_2I]^+$ (5^+)**	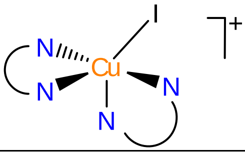	5<br>τ=0.85	+5.4
**$[Cu(Phen)(OAc)I]$**	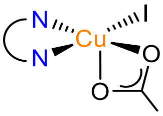	5<br>τ=0.48<br>ΔO=0.28	+9.8

**[Cu(Phen)$_2$(OAc)I]**	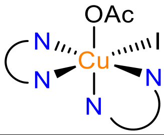	6<br>Jahn-Teller distorted Oh	+5.7
**[Cu(Phen)$_2$I(OH)]**	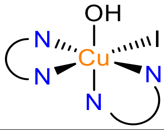	6<br>Jahn-Teller distorted Oh	+14.8
**[Cu(Phen)I$_2$]**	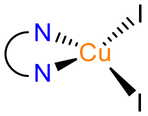	4<br>D$_{2d}$, θ=44.7°	+16.2
**[Cu(Phen)I(OH)]**	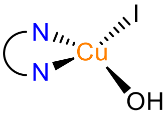	4<br>D$_{2d}$, θ=45.0°	+24.3
**[Cu(Phen)I]$^+$**	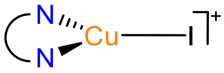	3<br>Trigonal Planar	+35.9
**[Cu(Phen)$_2$Cl]$^+$**	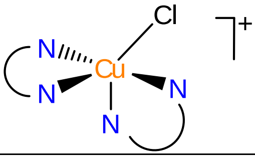	5<br>τ=0.80	+3.2
**[Cu(Phen)(OAc)Cl]**	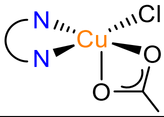	5<br>τ=0.23<br>ΔO=0.39	+6.4
**[Cu(Phen)$_2$(OAc)Cl]**	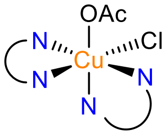	6<br>Jahn Teller distorted Oh	+4.1
**[Cu(Phen)$_2$Cl(OH)]**	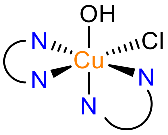	6<br>Jahn-Teller distorted Oh	+13.8
**[Cu(Phen)Cl$_2$]**	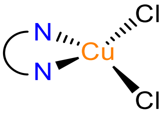	4<br>D$_{2d}$, θ=27.9°	+10.3
**[Cu(Phen)Cl(OH)]**	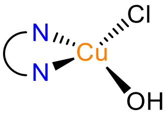	4<br>D$_{2d}$, θ=33.1°	+21.1
**[Cu(Phen)Cl]$^+$**	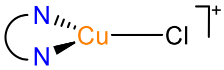	3<br>Trigonal Planar	+33.0

Bis-phen bis-halides species as well as solvent coordination at **3**$^+$ and **5**$^+$ were considered but not located, indicating no formation of these species experimentally.

1 Addison Parameter: $\tau = \frac{\alpha - \beta}{60}$ with α and β the two greatest angles. For ideal trigonal bipyramid τ = 1, for ideal square pyramid τ = 0.[22]

2 Dihedral angle, angle between the N-Cu-N and O-Cu-O planes.

Table S7: Computed relative energies of a range of different candidates of Cu(II)(aryl) complexes, focussing on a description of the coordination, geometry and stability compared to **[Cu(Phen)$_2$Ph]$^+$ (8$^+$)**. For species containing hydroxo ligands, energies include OH^- formation via deprotonation of water by acetate. Cu-O interactions were considered as bound up to 2.07 Å. κ^2 coordination mode was considered for $\Delta O < 0.4$ and $Cu\text{-}O_2 < 2.4$ Å.

Name	*Compound*	*Coordination Geometry*	*ΔG relative to CuPhen$_2$Ph (kcal/mol)*
**[Cu(Phen)$_2$(Ph)]$^+$ (8$^+$)**	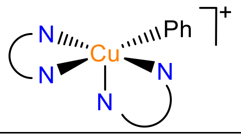	5<br>τ=0.37	0.0
**[Cu(Phen)$_2$(OAc)(Ph)]**	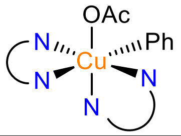	6<br>Jahn-Teller distorted O_h	+4.6
**[Cu(Phen)$_2$(Ph)(OH)]**	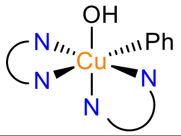	6<br>Jahn-Teller distorted O_h	+20.5
**[Cu(Phen)(Ph)(OH)]**	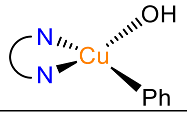	4<br>D_{2d}, θ=22.4°	+18.6
**[Cu(Phen)(OAc)(Ph)]**	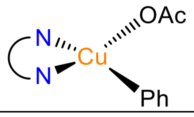	4<br>D_{2d}, θ=20.3°<br>ΔO=0.46	+4.7
**[Cu(Phen)(Ph)]$^+$**	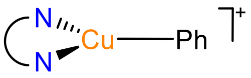	3<br>Trigonal Planar	+23.4
**[Cu(Phen)$_2$I(Ph)]**	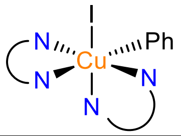	6<br>Jahn-Teller distorted O_h	+0.8
**[Cu(Phen)I(Ph)]**	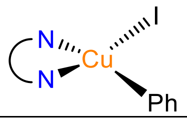	4<br>D_{2d}, θ=41.0°	+8.3
**[Cu(Phen)$_2$Cl(Ph)]**	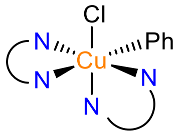	6<br>Jahn-Teller distorted O_h	+0.1
**[Cu(Phen)Cl(Ph)]**	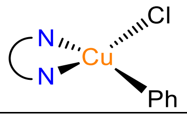	4<br>D_{2d}, θ=35.7°	+5.9

8.3 Alternative Transmetalation Mechanisms

8.3.1 Transmetalation involving Acetate as Base and Boronate Binding via Hydroxyl

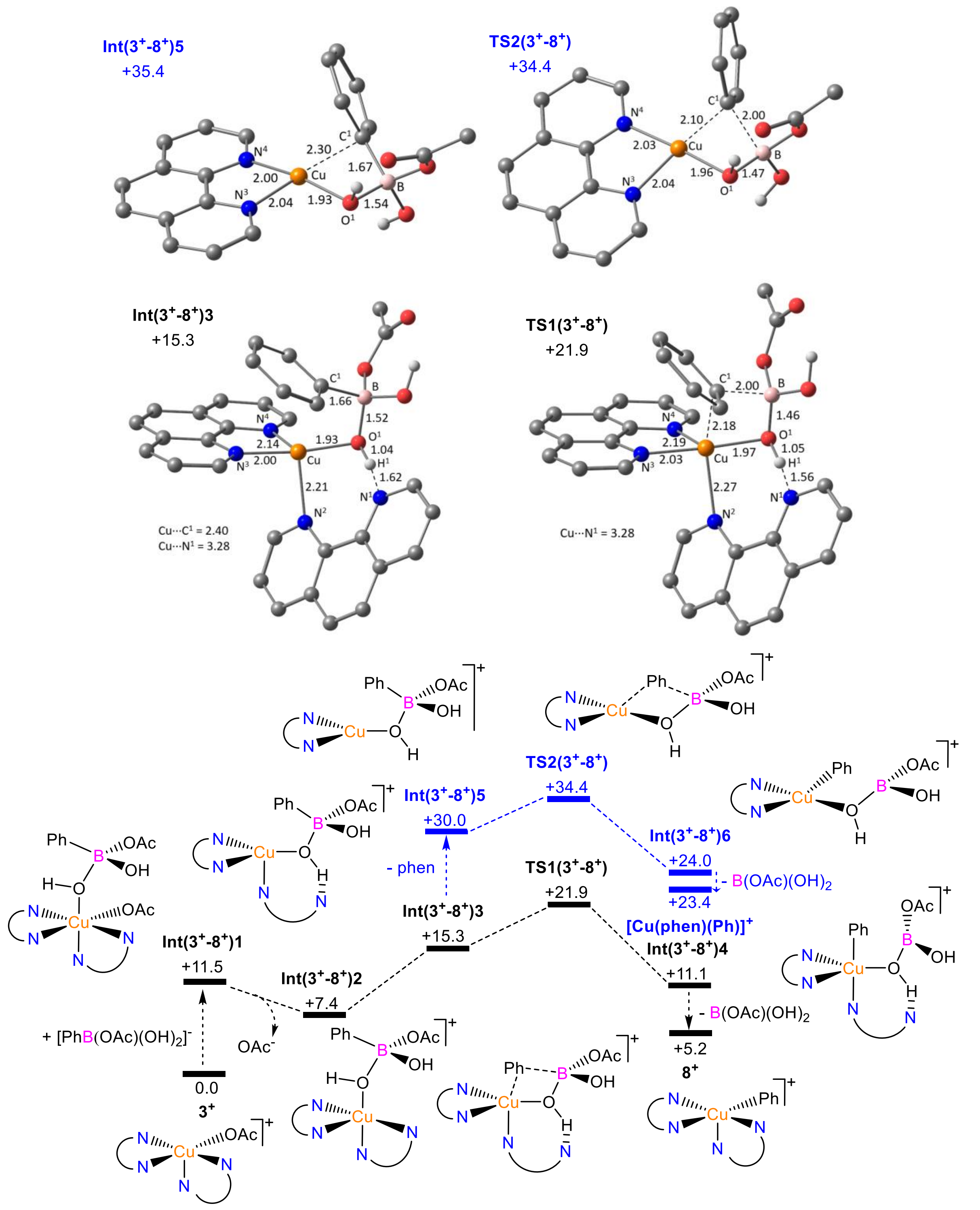

Figure S29: Computed reaction profile (free energies, kcal/mol) for the transmetalation step linking **3**$^{+}$ and **8**$^{+}$ with OAc as base and hydroxyl binding to Cu(II). Black pathway displays retention of both phenanthroline ligands, blue pathway displays loss of one phenanthroline. Details of the geometries of key stationary points are also shown with selected distances in Å (non-hydroxy H atoms omitted for clarity).

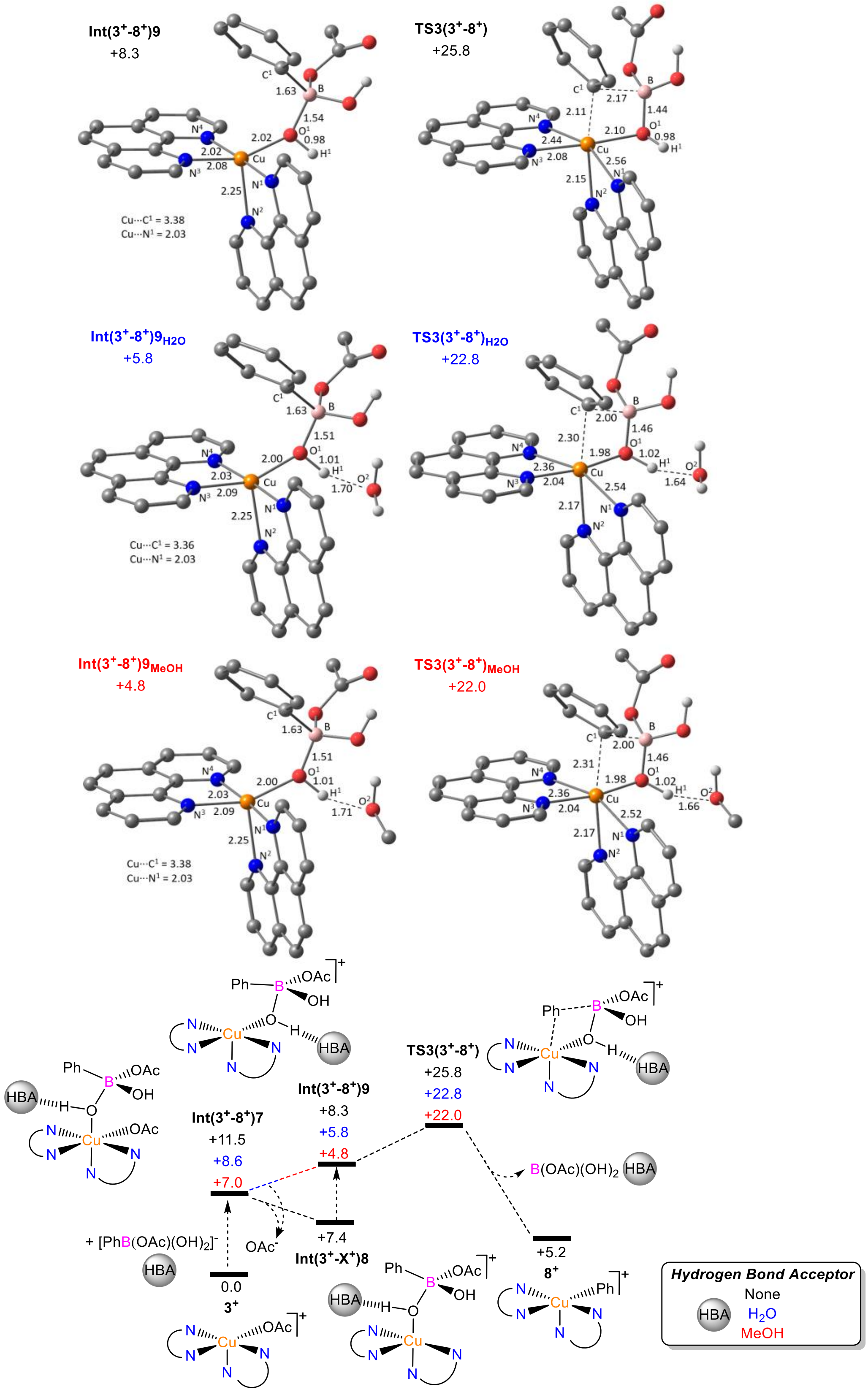

Figure S30: Computed reaction profile (free energies, kcal/mol) for the transmetalation step linking **3**$^+$ and **8**$^+$ with OAc as base and hydroxyl binding to Cu(II). κ^2-N binding of both phenanthrolines is displayed (black pathway) as well as the effect of hydrogen bonding with either water (blue) or MeOH (red) solvent molecules. Details of the geometries of key stationary points are also shown with selected distances in Å (non-hydroxy H atoms omitted for clarity).

8.3.2 Transmetalation involving Acetate as Base and Boronate Binding via Acetate

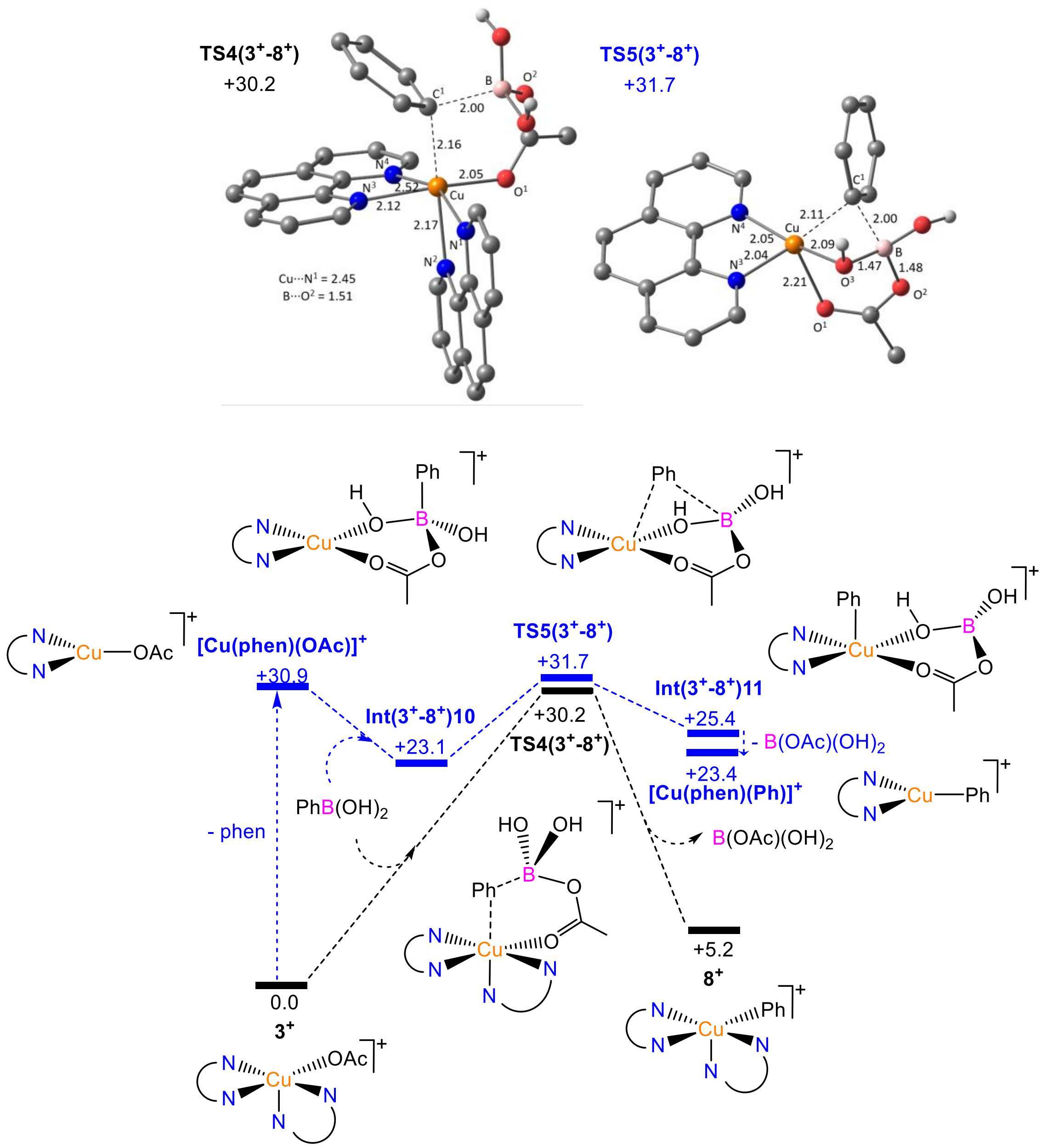

Figure S31: Computed reaction profile (free energies, kcal/mol) for the transmetalation step linking **3**$^{+}$ and **8**$^{+}$ with OAc as base and acetate binding to Cu(II) via the outer-oxygen. Black pathway displays retention of both phenanthrolines, blue pathway displays loss of one phenanthroline. Details of the geometries of key stationary points are also shown with selected distances in Å (non-hydroxy H atoms omitted for clarity).

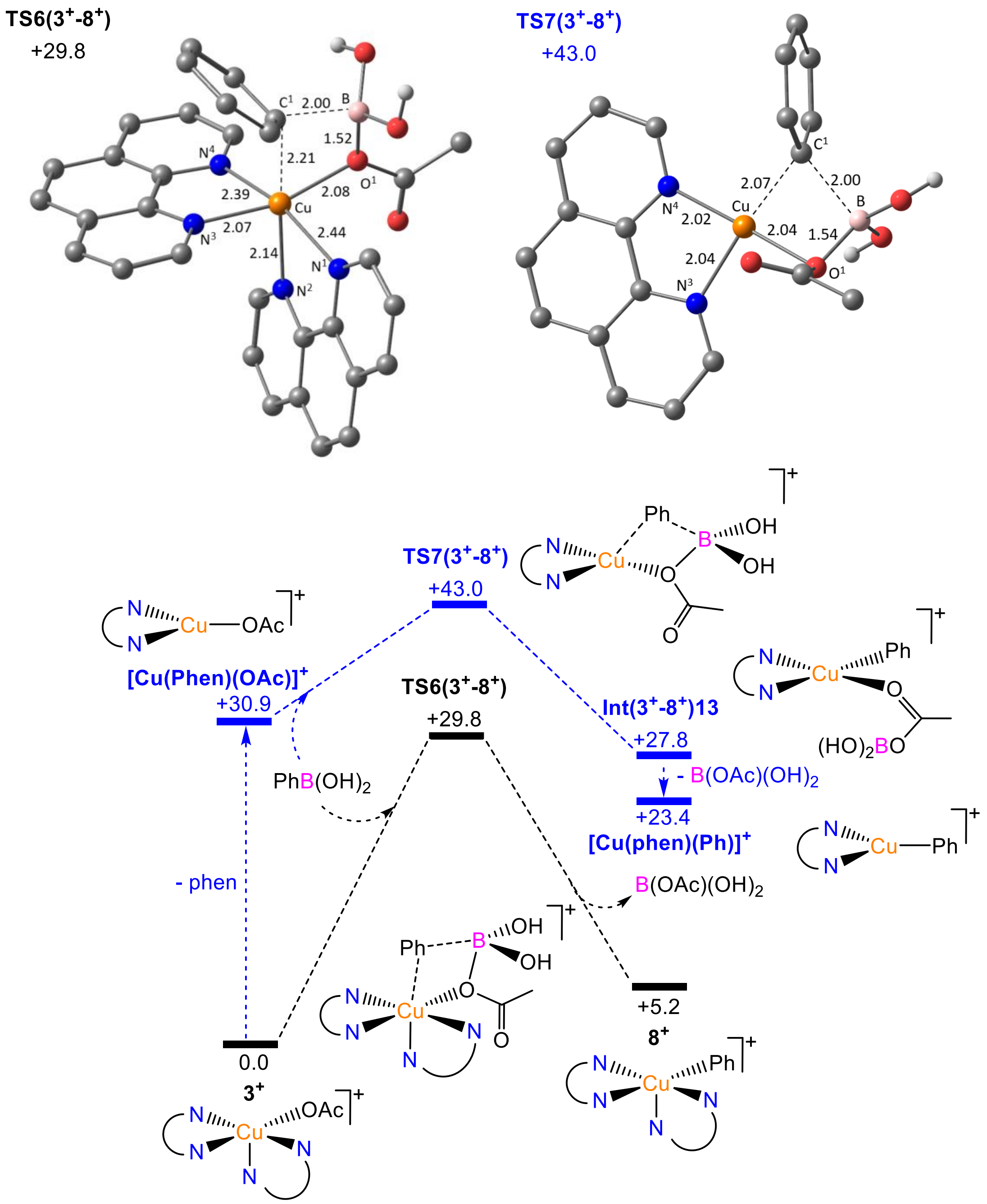

Figure S32: Computed reaction profile (free energies, kcal/mol) for the transmetalation step linking **3**$^+$ and **8**$^+$ with OAc as base and acetate binding to Cu(II) via the inner-oxygen. Black pathway displays retention of both phenanthrolines, blue pathway displays loss of one phenanthroline. Details of the geometries of key stationary points are also shown with selected distances in Å (non-hydroxy H atoms omitted for clarity).

8.3.3 Transmetalation involving Hydroxide as Base and Boronate Binding via Hydroxyl

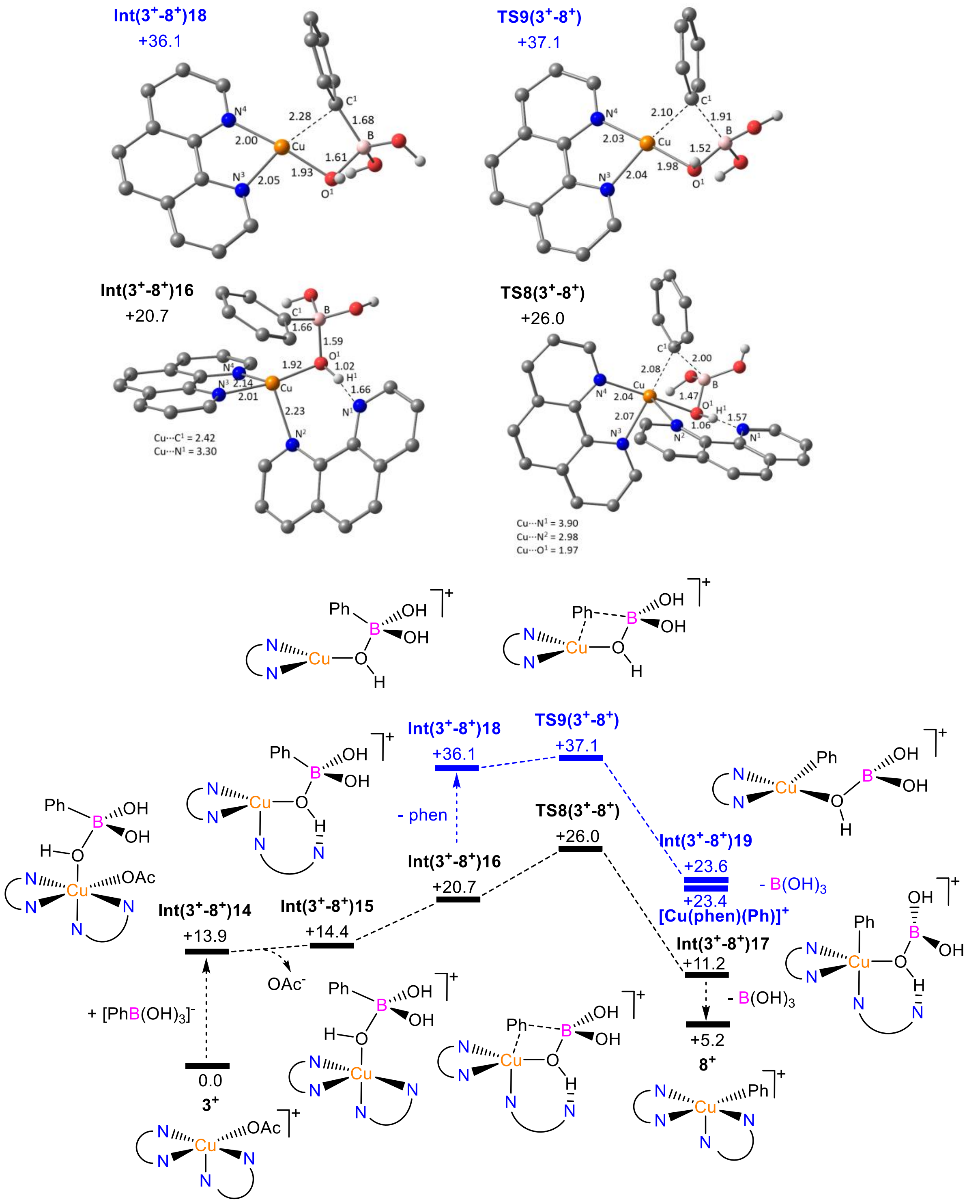

Figure S33: Computed reaction profile (free energies, kcal/mol) for the transmetalation step linking **3**$^+$ and **8**$^+$ with hydroxy as base and hydroxyl binding to Cu(II). Black pathway displays retention of both phenanthrolines, blue pathway displays loss of one phenanthroline. Details of the geometries of key stationary points are also shown with selected distances in Å (non-hydroxy H atoms omitted for clarity).

8.3.4 Transmetalation with PhBpin involving Hydroxide as Base and Pinacol Binding

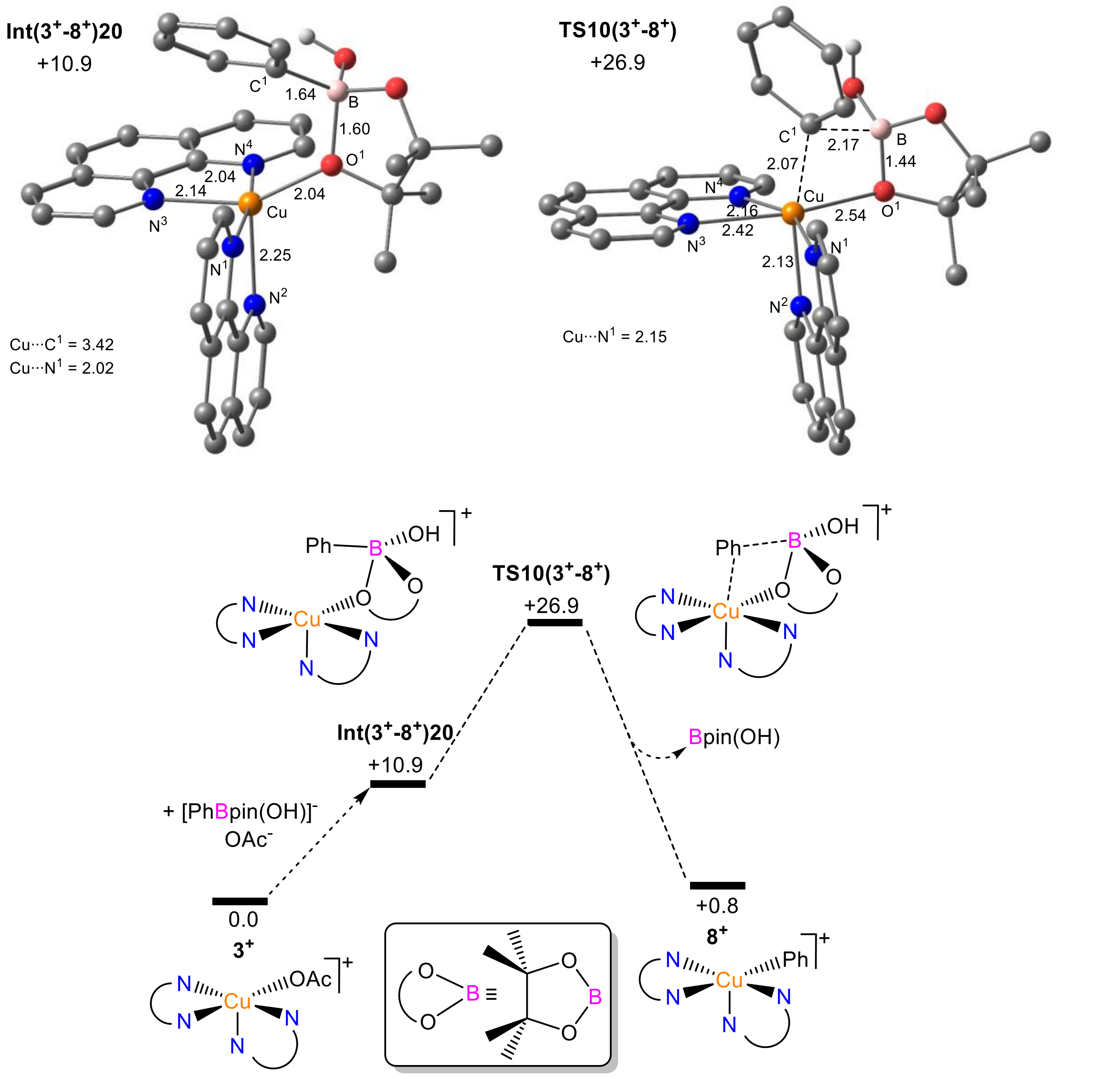

Figure S34: Computed reaction profile (free energies, kcal/mol) for the transmetalation step linking **3**$^+$ and **8**$^+$ with PhBpin as reactant, hydroxide as base and pinacol binding to Cu(II). Details of the geometries of key stationary points are also shown with selected distances in Å (non-hydroxy H atoms omitted for clarity).

8.3.5 Transmetalation with PhBpin involving Hydroxide as Base and Boronate Binding via Hydroxyl

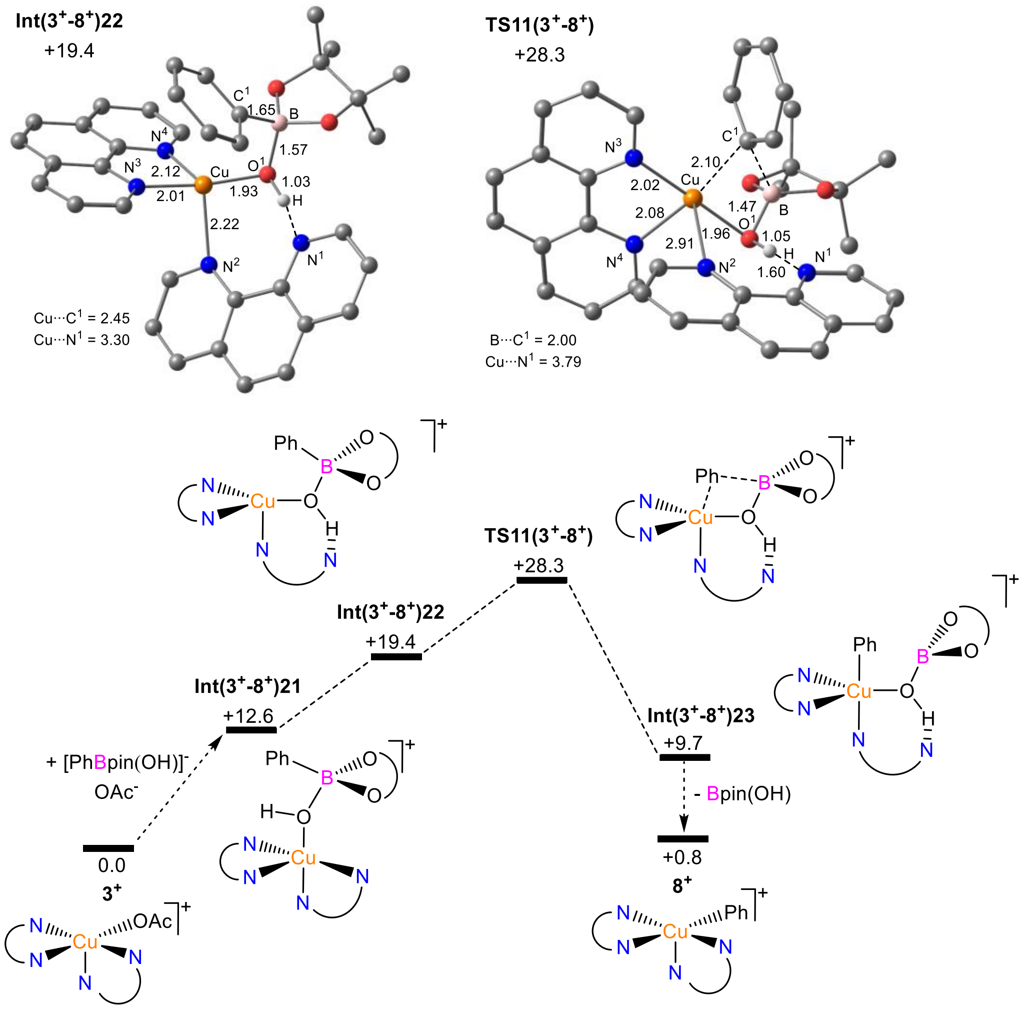

Figure S35: Computed reaction profile (free energies, kcal/mol) for the transmetalation step linking **3**$^+$ and **8**$^+$ with PhBpin as reactant, hydroxy as base and hydroxy binding to Cu(II). Details of the geometries of key stationary points are also shown with selected distances in Å (non-hydroxy H atoms omitted for clarity).

8.3.6 Transmetalation with PhBpin involving Acetate as Base and Boronate Binding via Pinacol

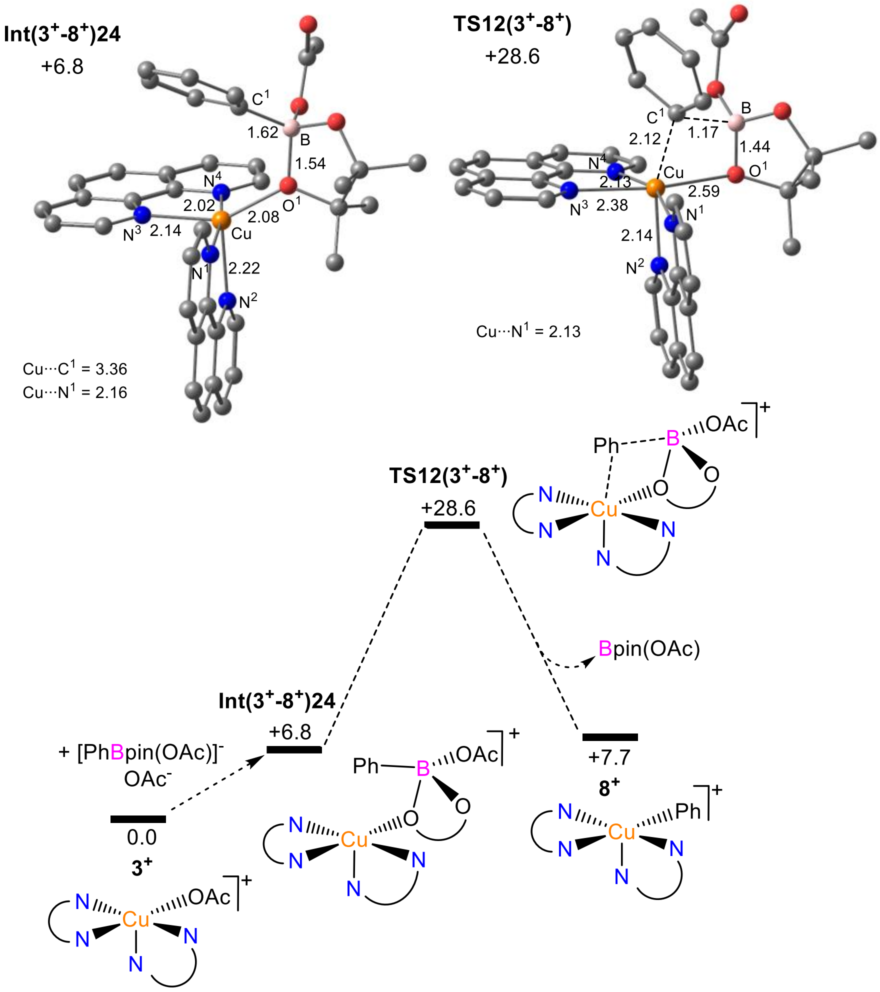

Figure S36: Computed reaction profile (free energies, kcal/mol) for the transmetalation step linking **3**$^+$ and **8**$^+$ with PhBpin as reactant, acetate as base and pinacol binding to Cu(II). Details of the geometries of key stationary points are also shown with selected distances in Å (non-hydroxy H atoms omitted for clarity).

8.3.7 Transmetalation with PhBpin involving Acetate as Base and Acetate Binding

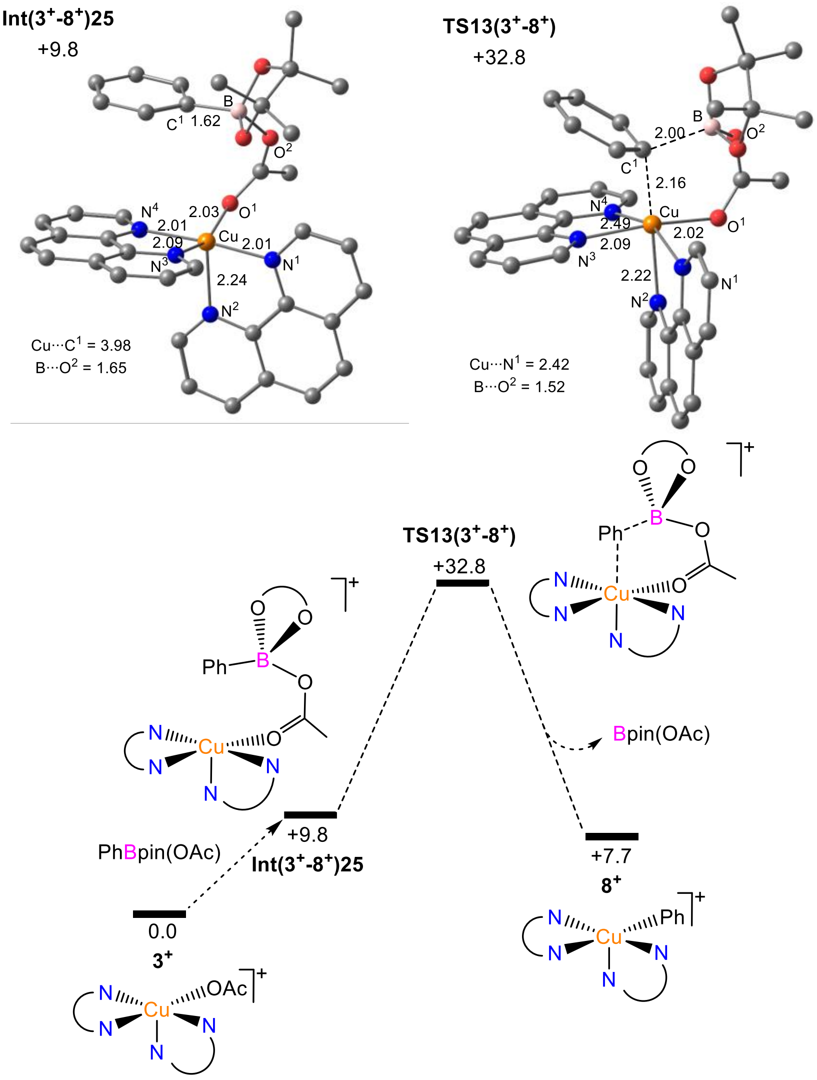

Figure S37: Computed reaction profile (free energies, kcal/mol) for the transmetalation step linking **3**$^+$ and **8**$^+$ with PhBpin as reactant, acetate as base and acetate binding to Cu(II) via the outer-oxygen. Details of the geometries of key stationary points are also shown with selected distances in Å (non-hydroxy H atoms omitted for clarity).

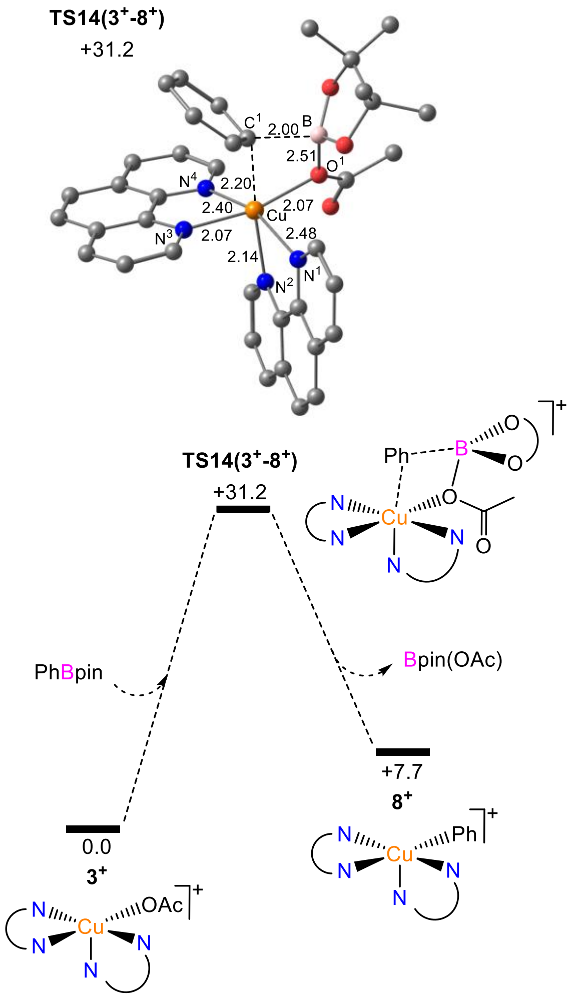

Figure S38: Computed reaction profile (free energies, kcal/mol) for the transmetalation step linking **3**$^{+}$ and **8**$^{+}$ with PhBpin as reactant, acetate as base and acetate binding to Cu(II) via the inner-oxygen. Details of the geometries of key stationary points are also shown with selected distances in Å (non-hydroxy H atoms omitted for clarity).

8.4 Alternative Disproportionation Pathways

Different disproportionation pathways were investigated thermodynamically, aiming to investigate the role of the phen and anionic ligands, including both iodide and chloride. Equation ***(1)*** involves simple electron transfer from **9** to $\mathbf{3}^{+}$ and is endergonic by 8.3 kcal/mol. Loss of phen requires an additional 8.6 kcal/mol. Equation ***(2)*** corresponds to electron transfer accompanied by anionic ligand exchange and is slightly less endergonic (+6.5 kcal/mol). Loss of phen requires an additional 6.5 kcal/mol. Loss of phen being required to form **11**, other pathways consider phen departure during disproportionation.

Equation ***(3)*** shows that disproportionation without acetate is endergonic by 11.1 kcal/mol. Equation ***(4)*** demonstrates that a second acetate is endergonic by ca. 5 kcal/mol. Equation ***(5)*** shows that halide is required prior to disproportionation. Equation ***(6)*** demonstrates that a second one disfavours the reaction by 5 to 10 kcal/mol. Note that ***(5)*** and ***(6)*** gives two combinations of products, endergonic.

Equations ***(7)*** involving replacement of OAc by iodide is disfavoured by 15.4 kcal/mol, whilst replacement of iodide by OAc in equation ***(8)*** is disfavoured by 14.6 kcal/mol. This shows that two inions of same nature, bound to different Cu, involve higher energies. Lowest energies are obtained when both acetate and iodide are bound to Cu(III), opposed to any two anions.

Combining all the above results, equation ***(9)*** is obtained, corresponding to electron and acetate transfer with loss of phen, and is endergonic by only 0.8 kcal/mol.

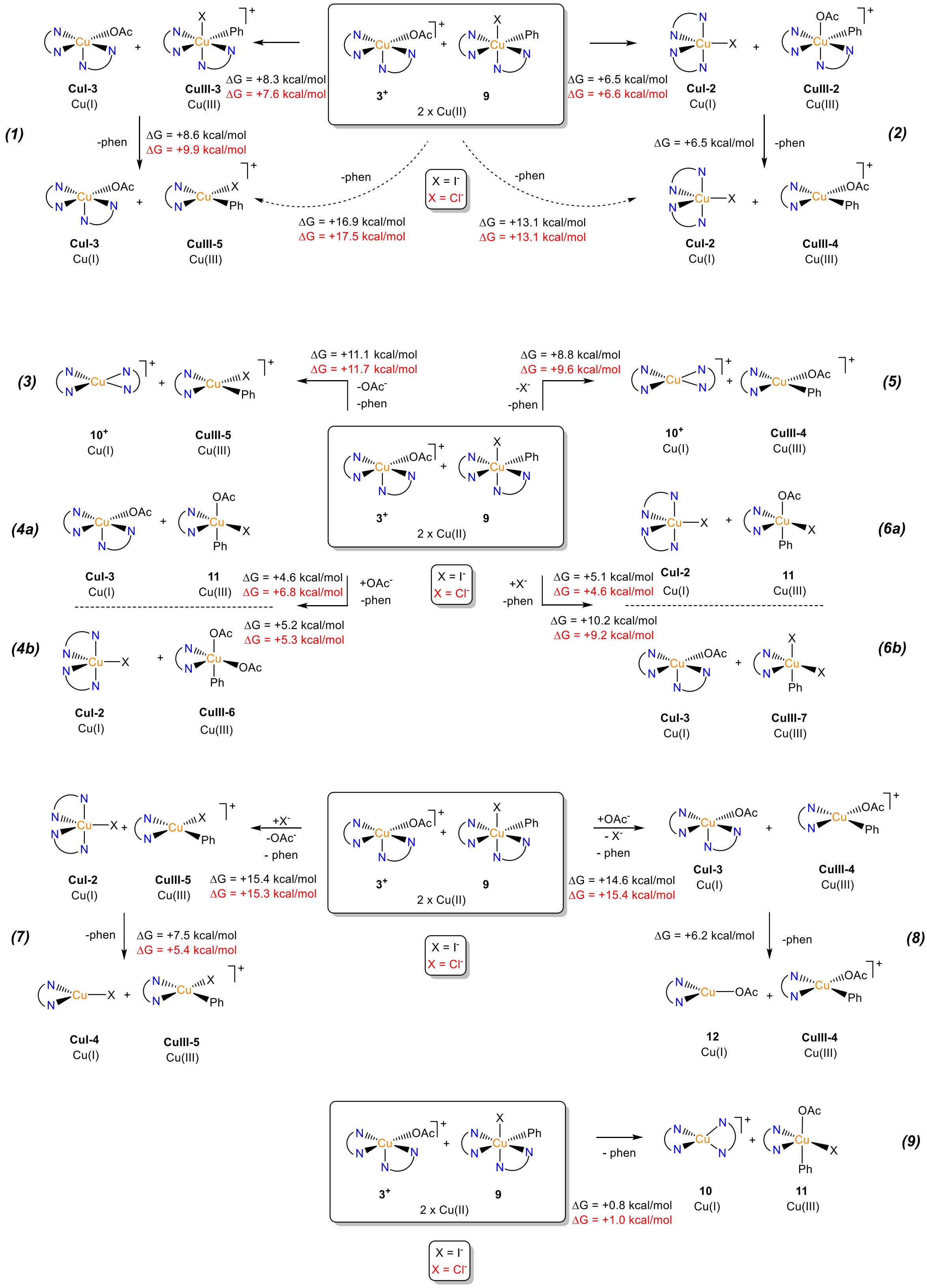

Scheme S1: Alternative disproportionation pathways of Cu(II) intermediates $\mathbf{3^+}$ and **9**, focussing on the role of phenanthroline and anionic ligands. Thermodynamics only are investigated (free energies, kcal/mol), in black for X=I and in red for X=Cl.

8.5 Cu(III) Isomers and Reductive Elimination

Table S8: Computed singlet-triplet energy difference for isomers of $\mathbf{11_{I}}$ and $\mathbf{11_{Cl}}$.

Name	*Compound*	*ΔG Singlet-Triplet (kcal/mol)*
	Iodide Ligand	
$\mathbf{11\text{-}1_{I}}$	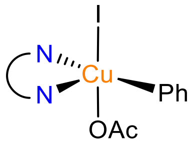	+18.6
$\mathbf{11\text{-}2_{I}}$	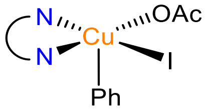	Singlet SCF convergence failure
**11**	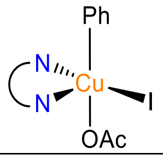	Triplet SCF convergence failure
$\mathbf{11\text{-}4_{I}}$	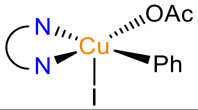	+17.5
$\mathbf{11\text{-}5_{I}}$	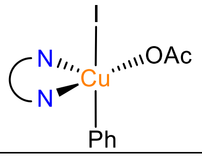	+18.2
	Chloride Ligand	
$\mathbf{11\text{-}1_{Cl}}$	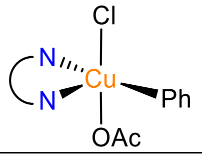	+18.9
$\mathbf{11\text{-}2_{Cl}}$	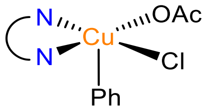	Singlet SCF convergence failure
$\mathbf{11\text{-}3_{Cl}}$	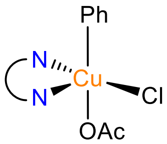	Triplet SCF convergence failure
$\mathbf{11\text{-}4_{Cl}}$	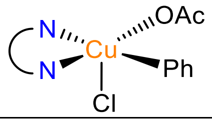	+18.6
$\mathbf{11\text{-}5_{Cl}}$	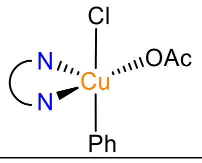	+19.2

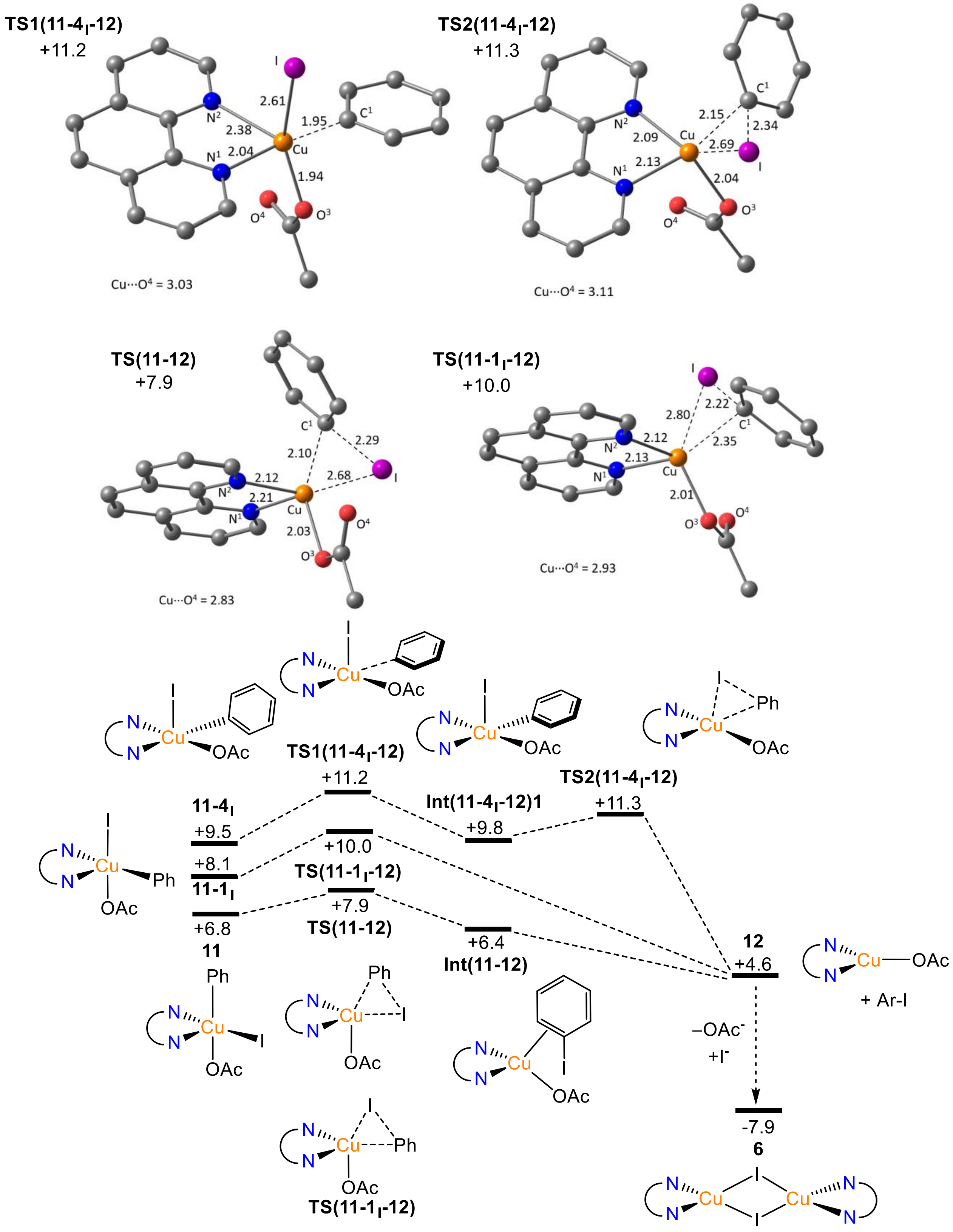

Figure S39: Computed reaction profiles (free energies, kcal/mol) for Ph-I reductive elimination from **11$_I$** isomers followed by dimerization. Details of the transition state geometries are also shown with selected distances in Å (non-hydroxy H atoms omitted for clarity). Note that an η^2-intermediate exists for **Int(11-3$_I$-12)1** and an extra **TS1(11-4$_I$-12)** is required to rotate the phenyl to allow coupling with the iodide.

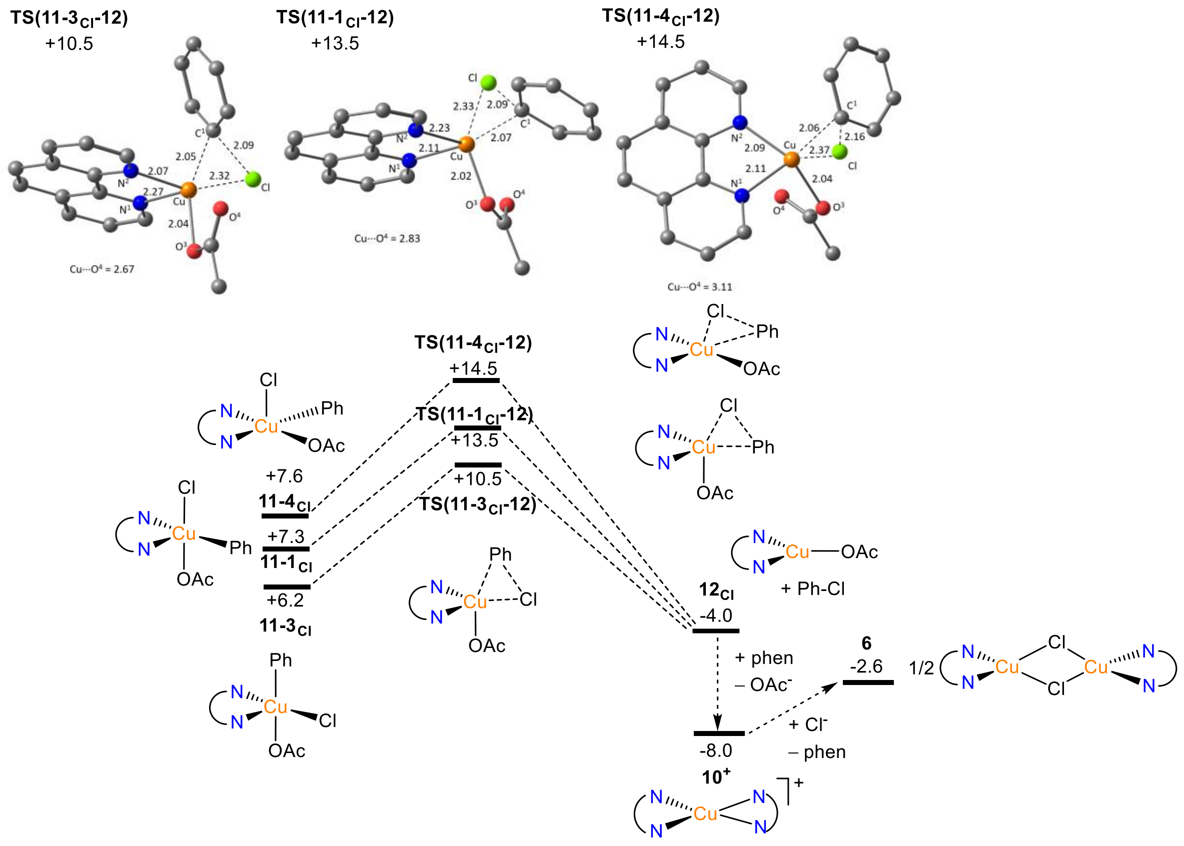

Figure S40: Computed reaction profiles (free energies, kcal/mol) for Ph-Cl reductive elimination from **11$_{Cl}$** isomers followed by dimerization. Details of the transition state geometries of **TS(11$_{Cl}$-12)** are also shown with selected distances in Å (non-hydroxy H atoms omitted for clarity). Note that reductive elimination happens in only one step for all three isomers.

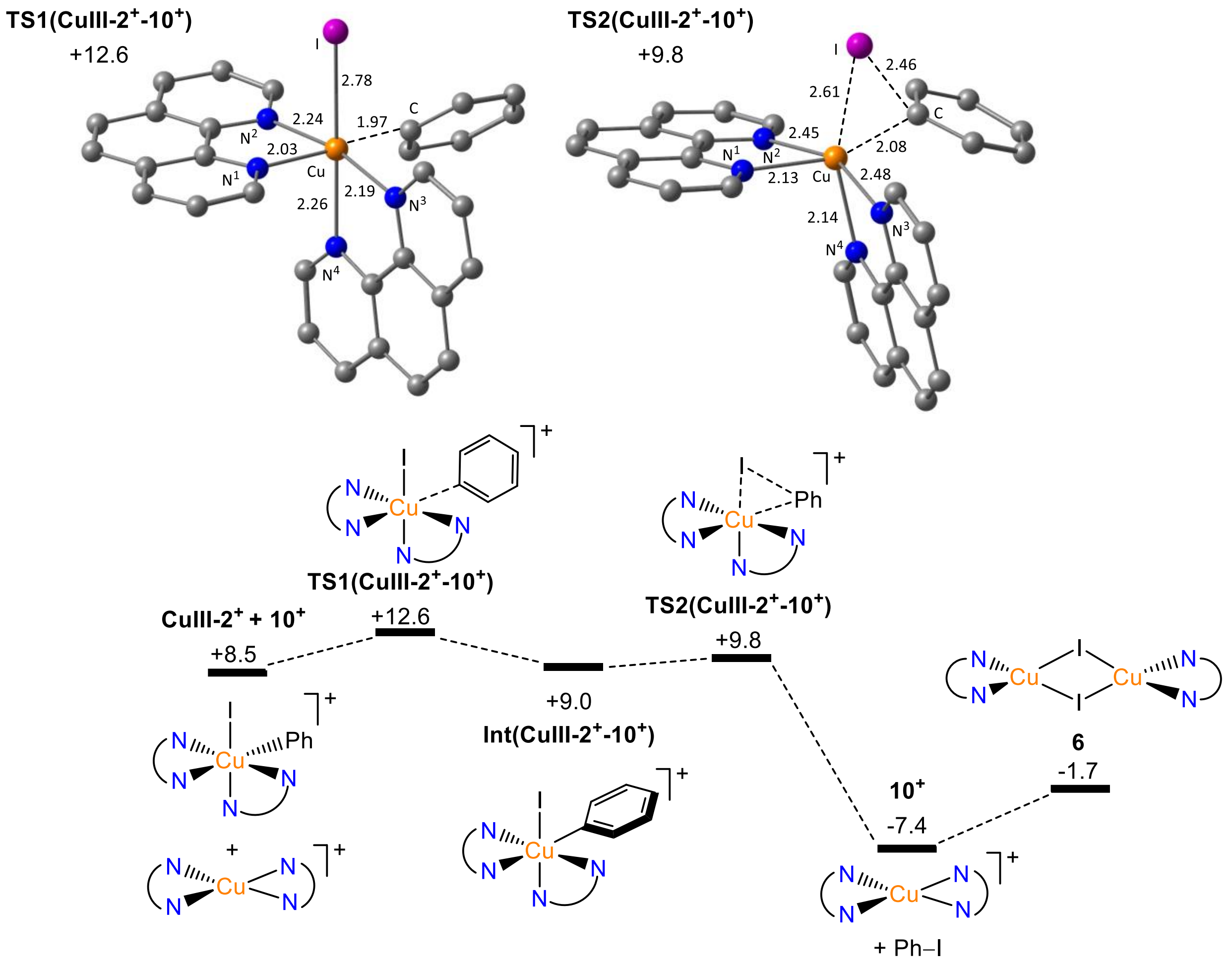

Figure S41: Alternative computed reaction profiles (free energies, kcal/mol) for Ph-I reductive elimination from **Cu(III)-2**$^{+}$ followed by dimerization. Details of the transition state geometries of are also shown with selected distances in Å (non-hydroxy H atoms omitted for clarity). Note that an extra **TS1(CuIII-2-10**$^{+}$**)** is required to rotate the phenyl to allow coupling with the iodide.

8.6 Functional Testing

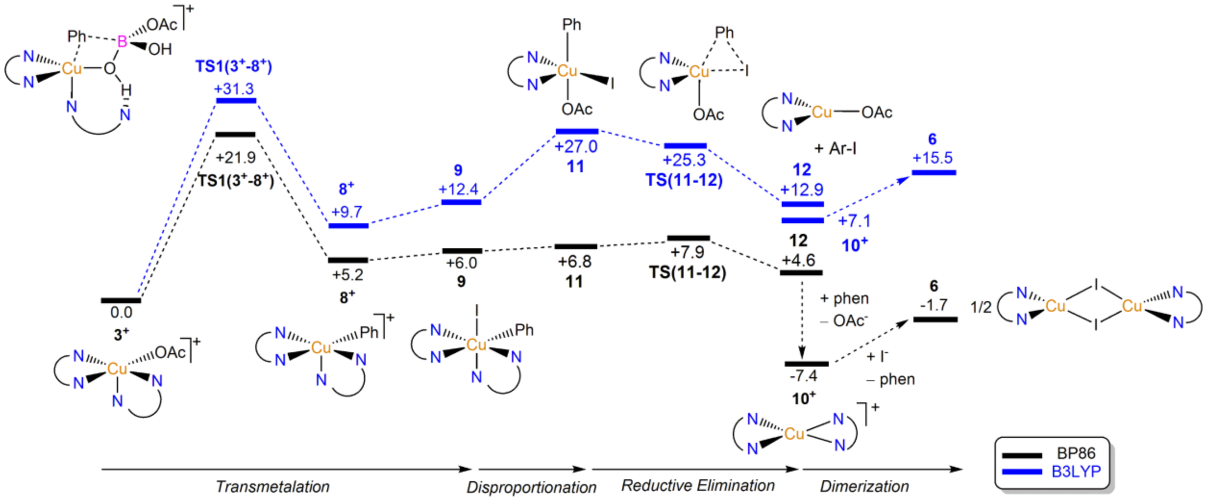

Figure S42: Computed reaction profiles (free energies, kcal/mol) displaying selected stationary points to assess functional dependence of the proposed mechanism. GGA functional BP86 is compared to hybrid B3LYP, showing a significant effect of hybrid functional to the energetics of disproportionation, whilst transmetalation is rate-determining step in both cases, in alignment with experiment.

8.7 Computed Geometries

H_2O
SCF = -76.4245783492
H(0 K)= -76.403825
H(298 K)= -76.400045
G(298 K)= -76.422168
SCF(D3BJ) = -76.4250383180
SCF(BS2) = -76.4701024562
SCF(BS2+D3BJ) = -76.4705615221
Low Freq. = 1628.1057cm-1, 3689.1573cm-1

```
O   0.00000  0.12202  0.00000
H   0.75998 -0.48807  0.00000
H  -0.75998 -0.48808 -0.00000
```

MeOH
SCF = -115.722405949
H(0 K)= -115.672579
H(298 K)= -115.668306
G(298 K)= -115.695343
SCF(D3BJ) = -115.724968824
SCF(BS2) = -115.776496418
SCF(BS2+D3BJ) = -115.779059292
Low Freq. = 330.0995cm-1, 1016.2818cm-1

```
C   0.66604 -0.01869 -0.00000
O  -0.75669  0.12335 -0.00000
H  -1.12763 -0.77845  0.00002
H   1.09125  0.99836 -0.00088
H   1.04685 -0.54655  0.89816
H   1.04679 -0.54808 -0.89728
```

phen
SCF = -571.617311322
H(0 K)= -571.451481
H(298 K)= -571.441052
G(298 K)= -571.486400
SCF(D3BJ) = -571.663654885
SCF(BS2) = -571.817697407
SCF(BS2+D3BJ) = -571.864117130
Low Freq. = 95.8167cm-1, 105.8719cm-1

```
C  -2.84413  0.84980  0.00000
C  -3.50383 -0.37240  0.00000
C  -2.72535 -1.55564  0.00000
N  -1.38818 -1.57850 -0.00000
C  -0.73191 -0.38273 -0.00000
C  -1.42373  0.87691 -0.00000
H  -3.40011  1.79379  0.00000
H  -4.59612 -0.43148  0.00000
H  -3.22412 -2.53431  0.00000
C   0.73206 -0.38333 -0.00000
C  -0.68646  2.11265 -0.00000
C   0.68641  2.11199  0.00000
C   1.42342  0.87642  0.00000
N   1.38874 -1.57976 -0.00000
C   2.84378  0.84986 -0.00000
C   3.50373 -0.37150  0.00000
C   2.72536 -1.55532  0.00000
H  -1.24574  3.05463  0.00000
H   1.24599  3.05396  0.00000
H   3.39890  1.79444  0.00000
H   4.59602 -0.42991  0.00001
H   3.22520 -2.53356  0.00000
```

OAc^-
SCF = -228.602243406
H(0 K)= -228.555256
H(298 K)= -228.549863
G(298 K)= -228.582534
SCF(D3BJ) = -228.607480618
SCF(BS2) = -228.725289594
SCF(BS2+D3BJ) = -228.730614885
Low Freq. = 57.7691cm-1, 416.6639cm-1

```
C   0.20314  0.00046 -0.00000
O   0.71739  1.16551  0.00000
O   0.80949 -1.11821  0.00000
C  -1.36026 -0.04535  0.00000
H  -1.75105 -1.07710  0.00000
H  -1.76066  0.48399  0.88470
H  -1.76066  0.48399 -0.88470
```

HOAc
SCF = -229.094763648
H(0 K)= -229.034903
H(298 K)= -229.029288
G(298 K)= -229.062371
SCF(D3BJ) = -229.100792999
SCF(BS2) = -229.196233710
SCF(BS2+D3BJ) = -229.202266983
Low Freq. = 54.8754cm-1, 412.1238cm-1

```
H  -1.87530  0.37830  0.00000
O  -1.25429 -0.38391  0.00000
O   0.19918  1.36356  0.00000
C   0.00000  0.15359  0.00000
```

C   1.06108 -0.92330 -0.00000
H   0.94614 -1.56694 -0.88745
H   2.05746 -0.46345 -0.00000
H   0.94614 -1.56694 0.88745

HO^-
SCF =   -75.8552419157
H(0 K)=   -75.847281
H(298 K)=   -75.843976
G(298 K)=   -75.863574
SCF(D3BJ) =   -75.8554399354
SCF(BS2) =   -75.9463977340
SCF(BS2+D3BJ) =   -75.9465957537
Low Freq. = 3494.4660cm-1, cm-1

O   0.00000 0.00000 0.10954
H   0.00000 0.00000 -0.87630

$PhB(OH)_2$
SCF =   -408.263645632
H(0 K)=   -408.142139
H(298 K)=   -408.133129
G(298 K)=   -408.175235
SCF(D3BJ) =   -408.288084251
SCF(BS2) =   -408.426855316
SCF(BS2+D3BJ) =   -408.451341885
Low Freq. = 49.0898cm-1,
136.1079cm-1

C -1.95775 -1.21654 -0.00255
C -0.55388 -1.21225 -0.00265
C -2.66248 0.00034 0.00036
H -0.00978 -2.16330 -0.00450
H -3.75798 0.00074 0.00084
C   0.17856 -0.00069 -0.00054
C -1.95697 1.21673 0.00275
H -2.50272 2.16667 0.00522
C -0.55317 1.21132 0.00200
H -0.00847 2.16200 0.00358
H -2.50435 -2.16598 -0.00459
B   1.75284 -0.00030 -0.00026
O   2.39371 1.22375 -0.00471
H   3.36621 1.16163 -0.00586
O   2.39610 -1.22307 0.00498
H   3.36866 -1.15916 0.00826

$PhB(OAc)(OH)_2$
SCF =   -636.892229430
H(0 K)=   -636.721941
H(298 K)=   -636.707764
G(298 K)=   -636.763160
SCF(D3BJ) =   -636.929113860
SCF(BS2) =   -637.164046443
SCF(BS2+D3BJ) =   -637.200946367
Low Freq. = 33.6934cm-1,
38.0488cm-1

C -3.12437 0.55353 1.15743
C -1.78752 0.11779 1.19964
C -3.80095 0.62437 -0.07352
H -1.27077 0.05809 2.16570
H -4.84273 0.96320 -0.11442
C -1.08085 -0.25928 0.03338
C -3.12729 0.25321 -1.25096
H -3.64717 0.30076 -2.21591
C -1.79059 -0.18012 -1.18808
H -1.27585 -0.47556 -2.11090
H -3.64179 0.83675 2.08248
B   0.47099 -0.73885 0.08826
O   0.79985 -1.58562 -1.05564
H   1.76791 -1.71473 -0.98335
O   0.81075 -1.27245 1.40515
H   1.77683 -1.42470 1.35791
O   1.34755 0.64282 -0.09308
O   3.27594 -0.56252 0.04248
C   2.65235 0.52160 -0.08193
C   3.40914 1.84368 -0.19593
H   3.58228 2.25192 0.81582
H   4.39118 1.68284 -0.66556
H   2.83284 2.58914 -0.76453

$B(OAc)(OH)_2$
SCF =   -405.138480887
H(0 K)=   -405.055130
H(298 K)=   -405.046033
G(298 K)=   -405.089177
SCF(D3BJ) =   -405.150440357
SCF(BS2) =   -405.320205336
SCF(BS2+D3BJ) =   -405.332175659
Low Freq. = 26.3419cm-1,
65.8580cm-1

O -0.14236 -0.37125 -0.62312
B   1.19125 -0.09348 -0.19235
O   1.83796 -1.13586 0.41475
O   1.68651 1.13800 -0.52491
C -1.16406 0.07008 0.16301
C -2.51546 -0.24711 -0.43423
O -0.97224 0.64422 1.23109
H   2.61050 1.28378 -0.25393
H   2.76845 -0.95340 0.63714
H -2.62764 -1.33822 -0.54679
H -3.31249 0.14320 0.21152
H -2.59684 0.19332 -1.44133

$PhB(OH)_3$
SCF =   -484.206715672
H(0 K)=   -484.074421

H(298 K)=   -484.063215
G(298 K)=   -484.109730
SCF(D3BJ) =   -484.234814570
SCF(BS2) =   -484.426486451
SCF(BS2+D3BJ) =   -484.454588161
Low Freq. = 54.8127cm-1, 104.3615cm-1

```
C  -0.82669  1.19982 -0.03728
C  -0.07871 -0.00282 -0.06726
C  -0.83707 -1.19992 -0.04553
C  -2.24327 -1.20587  0.00869
C  -2.95112  0.00935  0.04090
C  -2.23246  1.21855  0.01504
B   1.56929  0.00791 -0.03218
O   2.09713  0.06405  1.37805
O   2.06116  1.24625 -0.69601
O   2.01773 -1.25459 -0.69921
H  -0.29992 -2.15687 -0.08166
H  -4.04686  0.01400  0.08157
H  -2.77203  2.17448  0.03287
H  -0.27452  2.14820 -0.06843
H  -2.79125 -2.15704  0.02247
H   2.87754  1.46986 -0.21196
H   2.99277 -1.23665 -0.66130
H   2.07558 -0.85591  1.69741
```

B(OH)$_3$

SCF =   -252.483860367
H(0 K)=   -252.436746
H(298 K)=   -252.431187
G(298 K)=   -252.463365
SCF(D3BJ) =   -252.488406882
SCF(BS2) =   -252.608031429
SCF(BS2+D3BJ) =   -252.612558236
Low Freq. = 309.0730cm-1, 425.7533cm-1

```
O  -0.04276  1.36326  0.00067
B   0.00436 -0.02213 -0.00010
O  -1.08328 -0.87662 -0.00072
O   1.24000 -0.64041  0.00013
H   1.94900  0.02662 -0.00061
H  -1.93828 -0.41420  0.00576
H  -0.94422  1.72841 -0.00537
```

PhB(OAc)(OH)$_{H2O}$

SCF =   -713.338512836
H(0 K)=   -713.143311
H(298 K)=   -713.126814
G(298 K)=   -713.187801
SCF(D3BJ) =   -713.379783962
SCF(BS2) =   -713.646813101
SCF(BS2+D3BJ) =   -713.688084227
Low Freq. = 20.7940cm-1, 40.1150cm-1

```
O   0.80795 -1.55492 -1.37685
B   0.53860 -0.66680 -0.27615
O   1.20305  0.77359 -0.66605
C  -1.05928 -0.37126 -0.12646
C  -1.74942 -0.65811  1.07378
C  -3.12990 -0.42740  1.22080
C  -3.86910  0.10676  0.15142
C  -3.21250  0.40430 -1.05717
C  -1.83239  0.16822 -1.18400
O   1.19004 -1.12452  0.97573
H  -1.17645 -1.07840  1.91017
H  -4.94467  0.29007  0.25703
H  -3.77966  0.82213 -1.89820
H  -1.34016  0.41096 -2.13482
H  -3.63058 -0.66299  2.16818
H   1.73168 -1.87678 -1.24686
H   1.36084 -0.28631  1.48208
C   1.58790  1.65182  0.22906
O   1.61085  1.47064  1.47202
C   2.01083  2.99065 -0.36828
H   2.66074  2.83670 -1.24448
H   1.11673  3.53561 -0.71815
H   2.52897  3.60443  0.38225
O   3.27364 -2.35159 -0.11446
H   2.58336 -1.86871  0.44906
H   3.03502 -3.28820  0.01655
```

PhB(OAc)(OH)$_{2MeOH}$

SCF =   -752.636727685
H(0 K)=   -752.413903
H(298 K)=   -752.395862
G(298 K)=   -752.461369
SCF(D3BJ) =   -752.681570166
SCF(BS2) =   -752.953267729
SCF(BS2+D3BJ) =   -752.998110210
Low Freq. = 17.5996cm-1, 31.9884cm-1

```
C  -1.18140 -0.47776 -0.11520
C  -1.76185 -0.74232  1.14669
C  -3.14689 -0.92882  1.31190
C  -4.00209 -0.85599  0.19891
C  -3.45594 -0.59777 -1.07199
C  -2.07009 -0.41176 -1.21641
O   0.94161 -1.04083 -1.38622
B   0.42552 -0.26228 -0.28931
O   1.20758 -0.47153  0.95477
O   0.60367  1.30538 -0.70905
```

C 0.74284 2.27576 0.16273
C 0.72715 3.66743 -0.46159
O 0.86601 2.13387 1.40508
O 3.52184 -1.10590 -0.11237
H -1.09867 -0.81121 2.01844
H -5.08205 -1.00072 0.31853
H -4.11371 -0.54173 -1.94818
H -1.66359 -0.21427 -2.21667
H -3.56068 -1.13316 2.30718
H 1.92188 -1.04149 -1.28739
H 1.14232 0.39595 1.43816
H 1.37295 3.69899 -1.35376
H -0.29732 3.91111 -0.79280
H 1.05435 4.42269 0.26703
H 2.71721 -0.80985 0.43467
C 3.67483 -2.50324 0.12750
H 3.92939 -2.73017 1.18432
H 2.76329 -3.07976 -0.13395
H 4.50199 -2.87613 -0.50201

PhBpin
SCF = -642.928299826
H(0 K)= -642.663814
H(298 K)= -642.647982
G(298 K)= -642.705240
SCF(D3BJ) = -642.984273699
SCF(BS2) = -643.157115786
SCF(BS2+D3BJ) = -
643.213089662
Low Freq. = 31.1250cm-1,
53.8061cm-1

C -3.81572 1.20782 -0.15374
C -2.41228 1.20427 -0.15211
C -4.51959 0.00001 0.00002
H -1.86756 2.14784 -0.26923
H -5.61498 0.00002 0.00003
C -1.68306 -0.00002 -0.00003
C -3.81574 -1.20782 0.15375
H -4.36276 -2.14926 0.27318
C -2.41230 -1.20429 0.15209
H -1.86759 -2.14788 0.26921
H -4.36273 2.14927 -0.27313
B -0.12144 -0.00002 -0.00006
O 0.63575 -1.13594 0.23068
O 0.63574 1.13587 -0.23089
C 2.03430 -0.78771 -0.07874
C 2.03427 0.78768 0.07874
C 2.29231 -1.25637 -1.52082
H 1.64129 -0.72881 -2.23732
H 3.34190 -1.09332 -1.81558
H 2.07830 -2.33519 -1.59245
C 2.94552 -1.53580 0.89785
H 2.85727 -2.62210 0.73160
H 3.99970 -1.25333 0.73799
H 2.68162 -1.32563 1.94513
C 2.94552 1.53594 -0.89769
H 2.68196 1.32560 -1.94503
H 2.85683 2.62223 -0.73157
H 3.99976 1.25388 -0.73754
C 2.29205 1.25631 1.52087
H 3.34163 1.09340 1.81573
H 2.07790 2.33510 1.59251
H 1.64103 0.72862 2.23727

PhBpin(OAc)
SCF = -871.550166668
H(0 K)= -871.237296
H(298 K)= -871.216446
G(298 K)= -871.284990
SCF(D3BJ) = -871.625279133
SCF(BS2) = -871.884932635
SCF(BS2+D3BJ) = -
871.960045101
Low Freq. = 34.1819cm-1,
38.9029cm-1

C -3.74029 -0.80798 -0.98824
C -2.47120 -0.20911 -1.07752
C -4.05471 -1.63093 0.10857
H -2.25024 0.43903 -1.93555
H -5.04314 -2.09843 0.18535
C -1.47108 -0.40844 -0.09369
C -3.08695 -1.84338 1.10602
H -3.32195 -2.47818 1.96946
C -1.82070 -1.24014 0.99705
H -1.06990 -1.40838 1.77914
H -4.48789 -0.63233 -1.77162
B 0.02923 0.22116 -0.24564
O 0.80999 0.10959 1.00019
O 0.84109 -0.43263 -1.29628
C 2.16777 -0.21352 0.64595
C 1.97771 -1.05562 -0.67909
C 2.80736 -0.98180 1.81311
H 2.20432 -1.85919 2.09496
H 3.82543 -1.32455 1.55656
H 2.88707 -0.32523 2.69729
C 2.95591 1.09448 0.40353
H 2.87811 1.73041 1.30248
H 4.02704 0.90662 0.21130
H 2.53354 1.65218 -0.44704
C 1.63479 -2.53578 -0.38909
H 0.80401 -2.60768 0.33177
H 1.31367 -3.01719 -1.32884
H 2.49745 -3.09997 0.00732
C 3.16758 -0.99482 -1.65173
H 2.94660 -1.59337 -2.55298
H 3.37249 0.03830 -1.97307

H   4.08353 -1.40661 -1.19162
O -0.09705 1.69587 -0.75628
O -0.62721 3.86433 -0.62893
C -0.57115 2.74471 -0.09235
C -1.04039 2.53206 1.34328
H -1.26382 3.50106 1.81225
H -1.94803 1.90410 1.34549
H -0.27687 1.97800 1.91116

Bpin(OAc)
SCF =   -639.801172394
H(0 K)=   -639.574568
H(298 K)=   -639.558894
G(298 K)=   -639.615975
SCF(D3BJ) =   -639.844055343
SCF(BS2) =   -640.048254279
SCF(BS2+D3BJ) =   -
640.091137228
Low Freq. = 30.8427cm-1,
50.8958cm-1

B   0.56361 -0.37961 -0.03493
O -0.37379 -1.23544 -0.56862
O   0.06490 0.76633 0.53720
C -1.68570 -0.71811 -0.12731
C -1.36666 0.80687 0.16813
C -2.06777 -1.51495 1.13040
H -1.34410 -1.35212 1.94620
H -3.07081 -1.23666 1.49251
H -2.07547 -2.58971 0.88693
C -2.69888 -0.96048 -1.24774
H -2.83665 -2.04397 -1.39687
H -3.67803 -0.52770 -0.98272
H -2.36746 -0.52075 -2.20024
C -2.14450 1.41541 1.33633
H -1.96585 0.86965 2.27475
H -1.83660 2.46374 1.48235
H -3.22684 1.40650 1.12494
C -1.47000 1.70243 -1.07666
H -2.51940 1.84382 -1.38235
H -1.04194 2.69111 -0.84533
H -0.91215 1.27680 -1.92704
O   1.90866 -0.77216 -0.03707
O   2.84614 1.29038 -0.37611
C   2.96679 0.11384 -0.07639
C   4.26083 -0.58734 0.26175
H   4.41853 -1.43718 -0.42243
H   5.10009 0.11582 0.18688
H   4.20661 -1.00016 1.28283

PhBpin(OH)
SCF =   -718.875209177
H(0 K)=   -718.599630
H(298 K)=   -718.582093
G(298 K)=   -718.642613
SCF(D3BJ) =   -718.937842538
SCF(BS2) =   -719.154586527
SCF(BS2+D3BJ) =   -
719.217219888
Low Freq. = 21.0510cm-1,
56.6564cm-1

C   1.97656 -0.94871 -0.69388
C   1.56400 0.31606 -0.21115
C   2.55547 1.07642 0.45785
C   3.86842 0.60813 0.64586
C   4.24147 -0.65673 0.15452
C   3.28611 -1.43544 -0.52199
B   0.02374 0.86886 -0.38480
O   0.13131 2.23272 -0.94487
O -0.77214 -0.09702 -1.22994
C -2.03044 -0.34708 -0.60415
C -3.07328 0.67577 -1.12432
O -0.75062 0.92000 0.91262
C -1.70984 -0.13693 0.93226
C -2.91593 0.29827 1.78492
C -2.50784 -1.76316 -0.97370
C -1.07508 -1.39626 1.57194
H   2.29041 2.07238 0.83713
H   5.26354 -1.02785 0.29511
H   3.56497 -2.42110 -0.91643
H   1.23298 -1.55780 -1.22291
H   4.60502 1.22785 1.17312
H -1.74988 -2.52099 -0.71986
H -3.44782 -2.02283 -0.45391
H -2.69801 -1.82562 -2.06018
H -3.07611 0.65142 -2.22781
H -4.09806 0.45723 -0.77324
H -2.81490 1.70026 -0.80691
H -0.23222 -1.75788 0.96099
H -0.68317 -1.12940 2.56917
H -1.80048 -2.21997 1.69892
H -2.60529 0.43074 2.83677
H -3.33184 1.25618 1.43415
H -3.72013 -0.45931 1.76608
H -0.76392 2.61486 -0.87767
~

Bpin(OH)
SCF =   -487.147349912
H(0 K)=   -486.956744
H(298 K)=   -486.944822
G(298 K)=   -486.991557
SCF(D3BJ) =   -487.182374245
SCF(BS2) =   -487.335965126
SCF(BS2+D3BJ) =   -
487.370989460

Low Freq. = 101.1876cm-1, 117.5274cm-1

```
C  -0.81019 -1.45943  1.31240
C  -0.56063 -0.78202 -0.04593
O   0.81781 -1.11117 -0.43902
B   1.59899 -0.04998 -0.00992
O   0.88021  1.05406  0.42818
C  -0.51969  0.80004  0.04746
C  -1.43023  1.40923  1.11623
C  -1.51238 -1.34164 -1.10631
O   2.96956 -0.13163 -0.03101
C  -0.74262  1.49087 -1.30837
H  -0.13603 -1.06057  2.08856
H  -1.85075 -1.32076  1.64878
H  -0.62076 -2.54088  1.21520
H  -1.26028 -0.97660 -2.11331
H  -1.45440 -2.44269 -1.11461
H  -2.55452 -1.05943 -0.88073
H  -1.18859  1.03148  2.12107
H  -1.31469  2.50576  1.12331
H  -2.48776  1.18203  0.90022
H  -0.49406  2.56041 -1.21246
H  -0.09753  1.05628 -2.09000
H  -1.79190  1.40928 -1.63623
H   3.37011  0.71333  0.24171
```

PhI

```
SCF  =      -243.101321766
H(0 K)=     -243.013923
H(298 K)=   -243.006904
G(298 K)=   -243.045834
SCF(D3BJ) =       -243.124615264
SCF(BS2) =        -529.569248614
SCF(BS2+D3BJ) =   -529.592542271
```

Low Freq. = 145.7803cm-1, 211.2001cm-1

```
C   1.26253  1.22670  0.00000
C   0.58128 -0.00016  0.00001
C   1.26264 -1.22676  0.00001
C   2.66922 -1.21515 -0.00000
C   3.37312  0.00008 -0.00000
C   2.66895  1.21532 -0.00000
H   0.71711 -2.17378  0.00001
H   4.46735  0.00014 -0.00001
H   3.20897  2.16769  0.00000
H   0.71666  2.17350  0.00001
H   3.20925 -2.16752 -0.00001
I  -1.57030 -0.00000 -0.00000
```

PhCl

```
SCF  =      -246.673102623
H(0 K)=     -246.584801
H(298 K)=   -246.578160
G(298 K)=   -246.614732
SCF(D3BJ) =       -246.693734568
SCF(BS2) =        -691.987582978
SCF(BS2+D3BJ) =   -692.008214923
```

Low Freq. = 180.8050cm-1, 280.7868cm-1

```
C  -0.17932  1.22679  0.00000
C   0.49851  0.00000  0.00000
C  -0.17932 -1.22679  0.00000
C  -1.58473 -1.21596  0.00001
C  -2.28855  0.00000  0.00001
C  -1.58473  1.21596  0.00000
H   0.37728 -2.16731  0.00000
H  -3.38271  0.00000  0.00001
H  -2.12580  2.16743  0.00001
H   0.37728  2.16731  0.00000
H  -2.12580 -2.16743  0.00001
Cl  2.28168  0.00000 -0.00001
```

[3]$^{+}$

```
SCF  =      -1569.14301603
H(0 K)=     -1568.757018
H(298 K)=   -1568.728092
G(298 K)=   -1568.819405
SCF(D3BJ) =       -1569.28089830
SCF(BS2) =        -3012.95991672
SCF(BS2+D3BJ) =   -3013.09777196
```

Low Freq. = 9.3492cm-1, 16.4678cm-1

```
Cu  0.07659  0.70002 -0.03124
O  -0.31469  2.51794  0.72554
O   0.41586  3.02430 -1.35338
N   1.95287  0.74325  0.74387
N   0.95173 -0.80554 -1.20043
N  -1.02981 -0.64486  1.44348
N  -1.77431  0.58727 -0.88371
C   2.40644  1.54493  1.71696
H   1.68808  2.27328  2.10390
C   3.73377  1.46020  2.19619
H   4.05824  2.13523  2.99159
C   4.60413  0.52381  1.64585
H   5.63653  0.44306  1.99983
C   4.14712 -0.33464  0.61005
C   2.79402 -0.18084  0.19283
C   2.25799 -1.01088 -0.85158
C   0.42233 -1.56174 -2.17203
H  -0.62422 -1.37092 -2.42444
C   1.16494 -2.55744 -2.84761
H   0.68195 -3.14206 -3.63413
C   2.49452 -2.77484 -2.50047
H   3.08962 -3.53997 -3.00851
```

C 3.08422 -1.99131 -1.47289
C 4.44919 -2.13024 -1.03310
H 5.07670 -2.88583 -1.51536
C 4.95993 -1.33528 -0.03279
H 5.99843 -1.45105 0.29184
C -0.65412 -1.24446 2.57944
H 0.37735 -1.06888 2.90318
C -1.52017 -2.06371 3.34206
H -1.15597 -2.52701 4.26249
C -2.82418 -2.26028 2.90225
H -3.52119 -2.88602 3.46866
C -3.25853 -1.63803 1.70112
C -2.30671 -0.83370 1.00213
C -2.69986 -0.17642 -0.22670
C -2.12179 1.22824 -2.01087
H -1.34177 1.85002 -2.45998
C -3.41785 1.12832 -2.56301
H -3.65054 1.66654 -3.48490
C -4.37137 0.34686 -1.92005
H -5.38344 0.24979 -2.32549
C -4.03249 -0.32981 -0.71827
C -4.96338 -1.14947 0.01260
H -5.97910 -1.25864 -0.37973
C -4.59090 -1.78034 1.17533
H -5.30596 -2.40093 1.72446
C -0.00786 3.36899 -0.22287
C -0.19329 4.83769 0.14968
H 0.00223 5.48211 -0.71883
H 0.49905 5.10567 0.96542
H -1.21629 5.01279 0.52059

[Cu(Phen)$_2$(OH)]$^+$

SCF = -1416.45751813
H(0 K)= -1416.108438
H(298 K)= -1416.083117
G(298 K)= -1416.163924
SCF(D3BJ) = -1416.58279425
SCF(BS2) = -2860.21707123
SCF(BS2+D3BJ) = -2860.34233057
Low Freq. = 16.1464cm-1,
18.8443cm-1

Cu -0.11471 -0.25968 -1.03231
N 1.73764 0.65077 -1.18492
N 1.08828 -1.25076 0.68242
N -0.96288 1.22794 0.18652
N -2.04715 -0.93342 -0.95264
C 2.03966 1.57427 -2.11180
H 1.22831 1.87196 -2.78257
C 3.32722 2.14125 -2.22688
H 3.51726 2.89265 -2.99688
C 4.32744 1.72768 -1.35395
H 5.33633 2.14728 -1.41817
C 4.03959 0.74801 -0.36721
C 2.70859 0.22831 -0.31801
C 2.36643 -0.77702 0.66974
C 0.76501 -2.18409 1.58502
H -0.27017 -2.54210 1.56281
C 1.68635 -2.70111 2.52697
H 1.36272 -3.46410 3.23957
C 2.99171 -2.22340 2.52365
H 3.73192 -2.60009 3.23683
C 3.37135 -1.23275 1.57866
C 4.70068 -0.68612 1.50449
H 5.45415 -1.04771 2.21129
C 5.02222 0.26686 0.56831
H 6.03523 0.67770 0.51640
C -0.39325 2.30583 0.74478
H 0.66492 2.46846 0.52211
C -1.10629 3.19797 1.57789
H -0.58851 4.06123 2.00280
C -2.45234 2.96332 1.84119
H -3.02523 3.64016 2.48261
C -3.08671 1.82977 1.26688
C -2.28552 0.98928 0.44092
C -2.86829 -0.17356 -0.17348
C -2.53875 -2.02060 -1.56009
H -1.82150 -2.56920 -2.17846
C -3.89002 -2.41049 -1.41073
H -4.24885 -3.30579 -1.92422
C -4.74069 -1.64957 -0.61294
H -5.78939 -1.93436 -0.48186
C -4.24152 -0.48791 0.03565
C -5.02777 0.37762 0.87709
H -6.08227 0.13328 1.03791
C -4.47262 1.48955 1.46832
H -5.08075 2.13932 2.10509
O 0.19680 -1.40681 -2.51265
H 1.16186 -1.40379 -2.66346

[Cu(Phen)$_2$(OAc)$_2$]

SCF = -1797.76314354
H(0 K)= -1797.329599
H(298 K)= -1797.294283
G(298 K)= -1797.401246
SCF(D3BJ) = -1797.91668654
SCF(BS2) = -3241.68257606
SCF(BS2+D3BJ) = -3241.83623890
Low Freq. = 14.5533cm-1,
17.6487cm-1

Cu 0.31944 1.04273 0.31679
O -0.11366 1.56232 2.15108
O -2.01290 2.82683 1.93157
N 1.78798 -0.26000 1.17483
N 1.14034 0.20454 -1.37992

```
N   -0.93197 -1.66027  0.68006
N   -1.87366  0.64221 -0.58521
C    2.09616 -0.44375  2.46330
H    1.50823  0.14998  3.17064
C    3.10770 -1.34373  2.87821
H    3.32109 -1.45834  3.94409
C    3.81296 -2.06967  1.92193
H    4.59866 -2.77262  2.21662
C    3.50803 -1.89092  0.54558
C    2.47539 -0.95985  0.22977
C    2.12294 -0.71293 -1.14426
C    0.81025  0.48702 -2.64653
H    0.03719  1.24777 -2.78216
C    1.42809 -0.14804 -3.74795
H    1.11709  0.11613 -4.76174
C    2.42324 -1.09489 -3.52270
H    2.91769 -1.59976 -4.35859
C    2.80603 -1.40074 -2.19003
C    3.83668 -2.34618 -1.84233
H    4.35306 -2.87351 -2.65058
C    4.17486 -2.58099 -0.52930
H    4.96385 -3.29759 -0.28012
C   -0.49732 -2.76639  1.29106
H    0.51663 -2.72099  1.70767
C   -1.26076 -3.95133  1.42316
H   -0.84143 -4.82099  1.93685
C   -2.54143 -3.97279  0.88734
H   -3.17238 -4.86495  0.96246
C   -3.04443 -2.81796  0.23103
C   -2.18732 -1.66759  0.15406
C   -2.67602 -0.46236 -0.51093
C   -2.34789  1.73224 -1.20791
H   -1.66027  2.58325 -1.23699
C   -3.63173  1.80521 -1.79531
H   -3.95060  2.72629 -2.29101
C   -4.46161  0.69591 -1.72264
H   -5.46626  0.70691 -2.15884
C   -3.99899 -0.47709 -1.07099
C   -4.82078 -1.65369 -0.97086
H   -5.82382 -1.62518 -1.40964
C   -4.36237 -2.78361 -0.34225
H   -4.99054 -3.67755 -0.26695
C   -1.11375  2.28907  2.60045
C   -1.07916  2.43034  4.13291
H   -1.93468  3.02746  4.48061
H   -0.14085  2.91645  4.44910
H   -1.10702  1.43664  4.61057
O    0.34733  2.87268 -0.59686
O    2.51876  3.10662  0.05561
C    1.48815  3.50516 -0.53230
C    1.48429  4.85758 -1.26368
H    2.47573  5.33071 -1.21251
H    0.73643  5.52963 -0.80965
H    1.19626  4.71974 -2.31932
```

[Cu(Phen)$_2$(OH)$_2$]

```
SCF  =       -1492.39747654
H(0 K)=      -1492.038209
H(298 K)=    -1492.009914
G(298 K)=    -1492.098404
SCF(D3BJ) =        -1492.52716819
SCF(BS2) =         -2936.19802287
SCF(BS2+D3BJ) =    -2936.32767375
Low Freq. = 15.4863cm-1,
26.3844cm-1

Cu -0.02921 -0.04076  1.16630
O  -0.76818 -1.23643  2.47998
N   2.31440 -1.16718  0.69539
N   1.06674  1.08854 -0.32064
N  -1.07139 -1.07633 -0.35410
N  -2.29164  1.17938  0.69773
C   2.93078 -2.22440  1.23584
H   2.29071 -2.92412  1.78720
C   4.32064 -2.46673  1.11917
H   4.76351 -3.35075  1.58617
C   5.09303 -1.56849  0.39272
H   6.16954 -1.72321  0.26468
C   4.47719 -0.43110 -0.19499
C   3.06656 -0.26760  0.00209
C   2.40190  0.90063 -0.55791
C   0.46236  2.15437 -0.86676
H  -0.60405  2.25337 -0.63901
C   1.13327  3.10375 -1.66874
H   0.57889  3.94823 -2.08650
C   2.49411  2.94487 -1.89656
H   3.05575  3.66629 -2.49912
C   3.16863  1.82807 -1.33707
C   4.57942  1.61509 -1.52656
H   5.13884  2.34352 -2.12251
C   5.21311  0.53241 -0.96876
H   6.28876  0.38179 -1.10794
C  -0.47463 -2.13394 -0.92422
H   0.59204 -2.24464 -0.70334
C  -1.15604 -3.06185 -1.74177
H  -0.60950 -3.90078 -2.18039
C  -2.51783 -2.89058 -1.95643
H  -3.08743 -3.59713 -2.56905
C  -3.18298 -1.78248 -1.36907
C  -2.40639 -0.87443 -0.57749
C  -3.05714  0.28833  0.00750
C  -2.89882  2.22828  1.26430
H  -2.24967  2.92445  1.80955
C  -4.29142  2.46910  1.18082
H  -4.72598  3.34410  1.67202
C  -5.07623  1.58380  0.45207
H  -6.15480  1.74018  0.34519
```

```
C  -4.46980  0.45794 -0.16674
C  -5.21795 -0.48769 -0.95125
H  -6.29464 -0.33056 -1.07456
C  -4.59473 -1.55992 -1.54008
H  -5.16326 -2.27325 -2.14562
H  -0.36299 -0.74395  3.22630
O   0.69962  1.03867  2.60300
H   1.66080  0.93524  2.46093
```

[Cu(Phen)$_2$(OAc)(OH)]

```
SCF  =      -1645.08306551
H(0 K)=     -1644.686161
H(298 K)=   -1644.654641
G(298 K)=   -1644.750397
SCF(D3BJ) =       -1645.22498607
SCF(BS2) =        -3088.94228295
SCF(BS2+D3BJ) =   -3089.08422671
Low Freq. = 17.4673cm-1,
22.6641cm-1
```

```
Cu  0.00279  0.41372 -0.89534
O  -1.24070  1.98451 -1.29870
O   0.10488  3.73074 -0.67756
N   2.26050  1.11187  0.00986
N   1.17361 -1.37770 -0.42774
N  -0.88271  0.33713  1.01321
N  -2.14869 -0.91307 -1.10854
C   2.79477  2.33103  0.15709
H   2.08579  3.15657  0.01234
C   4.16198  2.54261  0.46298
H   4.54427  3.56156  0.57194
C   4.99341  1.44128  0.62914
H   6.05307  1.56392  0.87723
C   4.46051  0.13341  0.47120
C   3.07042  0.02704  0.14470
C   2.48767 -1.28868 -0.05973
C   0.63642 -2.59274 -0.60815
H  -0.41705 -2.60141 -0.90383
C   1.36229 -3.79275 -0.43851
H   0.86460 -4.75283 -0.59766
C   2.70329 -3.72094 -0.08206
H   3.30280 -4.62806  0.04696
C   3.30691 -2.45107  0.11376
C   4.69483 -2.30142  0.46476
H   5.29954 -3.20545  0.58993
C   5.25302 -1.05700  0.62881
H   6.31229 -0.95094  0.88535
C  -0.25586  0.88144  2.06703
H   0.76315  1.23032  1.87842
C  -0.85242  1.01676  3.33951
H  -0.28399  1.46816  4.15658
C  -2.15983  0.58389  3.51564
H  -2.66456  0.68619  4.48184
C  -2.85431  0.00066  2.42359
C  -2.16317 -0.12178  1.17455
C  -2.84314 -0.74395  0.05014
C  -2.78317 -1.45209 -2.15509
H  -2.18964 -1.56954 -3.06974
C  -4.13627 -1.86761 -2.12100
H  -4.59842 -2.29871 -3.01324
C  -4.84496 -1.72938 -0.93393
H  -5.88817 -2.05313 -0.85718
C  -4.20610 -1.15977  0.20003
C  -4.87193 -1.00133  1.46503
H  -5.91141 -1.33328  1.55559
C  -4.21542 -0.45306  2.53888
H  -4.71896 -0.34218  3.50460
C  -0.95783  3.24313 -1.13158
C  -2.09560  4.18532 -1.57322
H  -1.86444  5.22793 -1.30885
H  -3.04907  3.88854 -1.10557
H  -2.23605  4.11531 -2.66578
O   0.65590  0.41058 -2.68593
H   1.42274 -0.19331 -2.64548
```

[Cu(Phen)(OH)$_2$]

```
SCF  =      -920.783957849
H(0 K)=     -920.591074
H(298 K)=   -920.574400
G(298 K)=   -920.635454
SCF(D3BJ) =       -920.846228438
SCF(BS2) =        -2364.38990348
SCF(BS2+D3BJ) =   -2364.45214931
Low Freq. = 17.9672cm-1,
67.0131cm-1
```

```
Cu  1.92813 -0.00259 -0.00138
N   0.32766 -1.32976 -0.00616
N   0.33165  1.32760 -0.01289
C   0.37999 -2.66838 -0.00637
H   1.38892 -3.09268 -0.01152
C  -0.78922 -3.46454 -0.00061
H  -0.69636 -4.55350 -0.00093
C  -2.04015 -2.85130  0.00587
H  -2.95725 -3.44894  0.01126
C  -2.12344 -1.43277  0.00512
C  -0.89154 -0.71872 -0.00191
C  -0.88931  0.72028 -0.00469
C   0.38824  2.66606 -0.01652
H   1.39873  3.08661 -0.02164
C  -0.77841  3.46597 -0.01157
H  -0.68206  4.55460 -0.01455
C  -2.03122  2.85651 -0.00230
H  -2.94670  3.45666  0.00271
C  -2.11883  1.43828  0.00084
C  -3.35286  0.69334  0.00914
```

```
H  -4.29655  1.24776  0.01347
C  -3.35514 -0.68378  0.01102
H  -4.30085 -1.23474  0.01722
O   3.13076 -1.45485 -0.01644
H   4.03706 -1.09146 -0.02130
O   3.13151  1.44928  0.04269
H   4.03714  1.08473  0.06067
```

[Cu(Phen)(OAc)$_2$]

```
SCF  =      -1226.15321865
H(0 K)=     -1225.886194
H(298 K)=   -1225.862525
G(298 K)=   -1225.942192
SCF(D3BJ) =       -1226.23631849
SCF(BS2) =        -2669.87485309
SCF(BS2+D3BJ) =   -2669.95795682
Low Freq. = 27.0783cm-1,
29.7405cm-1

Cu -1.04026 -0.00163 -0.00123
O  -2.38892  1.46470 -0.09044
O  -1.84227  1.25319  2.08630
N   0.51755  1.26392 -0.42210
N   0.51581 -1.27107  0.41252
C   0.46587  2.53338 -0.84610
H  -0.53757  2.95035 -0.97302
C   1.63619  3.28477 -1.10392
H   1.54317  4.31647 -1.45150
C   2.88507  2.70105 -0.90918
H   3.80289  3.26594 -1.09985
C   2.96712  1.35645 -0.45627
C   1.73611  0.67861 -0.22954
C   1.73511 -0.68608  0.22383
C   0.46231 -2.54124  0.83430
H  -0.54177 -2.95739  0.95875
C   1.63163 -3.29380  1.09336
H   1.53743 -4.32625  1.43835
C   2.88127 -2.71007  0.90362
H   3.79837 -3.27566  1.09574
C   2.96507 -1.36464  0.45361
C   4.19850 -0.65680  0.22016
H   5.14212 -1.18129  0.39874
C   4.19961  0.64859 -0.21737
H   5.14439  1.17249 -0.39187
C  -2.50654  1.79993  1.16894
C  -3.52122  2.90039  1.46344
H  -3.46589  3.20498  2.51813
H  -4.53901  2.53447  1.24586
H  -3.34303  3.77217  0.81326
O  -2.39045 -1.46606  0.10156
O  -1.87703 -1.23694 -2.08111
C  -2.53067 -1.78828 -1.15879
C  -3.56118 -2.87508 -1.44835
H  -3.50008 -3.19612 -2.49785
H  -4.57413 -2.48507 -1.25019
H  -3.40886 -3.73978 -0.78265
```

[Cu(Phen)(OAc)(OH)]

```
SCF  =      -1073.46492824
H(0 K)=     -1073.234808
H(298 K)=   -1073.214723
G(298 K)=   -1073.284917
SCF(D3BJ) =       -1073.53781369
SCF(BS2) =        -2517.12878441
SCF(BS2+D3BJ) =   -2517.20160403
Low Freq. = 22.1573cm-1,
32.0575cm-1

Cu  1.18251 -0.73368 -0.53037
N  -0.72480 -1.41437 -0.08744
N   0.21933  1.06771 -0.38451
C  -1.15186 -2.67313  0.08825
H  -0.39493 -3.45762 -0.00701
C  -2.49941 -2.97860  0.38575
H  -2.79424 -4.02210  0.51969
C  -3.42648 -1.94674  0.50321
H  -4.47569 -2.15999  0.73053
C  -3.00163 -0.60201  0.33152
C  -1.62342 -0.39238  0.03806
C  -1.11617  0.94384 -0.12570
C   0.73648  2.29501 -0.53877
H   1.81258  2.33101 -0.73275
C  -0.05635  3.46205 -0.44664
H   0.41303  4.43940 -0.58265
C  -1.41834  3.34895 -0.18095
H  -2.05014  4.23911 -0.10101
C  -1.99198  2.06042 -0.00856
C  -3.38406  1.82162  0.27653
H  -4.05387  2.68259  0.36318
C  -3.86839  0.54390  0.44134
H  -4.92703  0.37774  0.66295
O   2.99225  0.17466 -0.48899
O   2.52041 -0.40820  1.63971
C   3.28367  0.10818  0.78214
C   4.63719  0.69613  1.17743
H   4.73733  1.72374  0.79160
H   4.75591  0.69700  2.27027
H   5.44782  0.09920  0.72617
O   1.85956 -2.33261 -1.24468
H   1.08161 -2.84106 -1.54663
```

[Cu(Phen)(OAc)]$^+$

```
SCF  =      -997.470798469
H(0 K)=     -997.252592
H(298 K)=   -997.234931
```

G(298 K)=   -997.299425
SCF(D3BJ) =   -997.538140745
SCF(BS2) =   -2441.09576284
SCF(BS2+D3BJ) =   -2441.16310466
Low Freq. = 23.1930cm-1, 35.1897cm-1

```
C  -3.92391  0.44252  0.00048
C  -3.04604 -0.70058  0.00030
C  -1.64287 -0.47590  0.00001
C  -1.12596  0.86041 -0.00009
C  -2.00614  1.97588  0.00008
C  -3.42537  1.72631  0.00038
N  -0.72491 -1.49187 -0.00020
C  -1.15604 -2.76374 -0.00014
C  -2.53376 -3.07836  0.00015
C  -3.47759 -2.05478  0.00036
C  -1.40444  3.26439 -0.00006
C  -0.01711  3.37979 -0.00032
C   0.78761  2.21469 -0.00044
N   0.23271  0.99761 -0.00034
Cu  1.11468 -0.80265 -0.00047
O   2.92770 -0.95370 -0.00046
C   3.92516 -0.07238  0.00020
C   5.30103 -0.70004  0.00111
O   3.72806  1.15190  0.00052
H  -0.39056 -3.54421 -0.00033
H  -2.83663 -4.12763  0.00019
H  -4.54752 -2.28359  0.00057
H   1.88480  2.23284 -0.00058
H   0.46835  4.35831 -0.00042
H  -2.03782  4.15665  0.00004
H  -4.10501  2.58348  0.00052
H  -5.00397  0.26912  0.00072
H   5.42065 -1.34217 -0.88699
H   6.07410  0.08069  0.00125
H   5.41973 -1.34151  0.88981
```

[Cu(Phen)(OH)]⁺

SCF =   -844.799830084
H(0 K)=   -844.618000
H(298 K)=   -844.604007
G(298 K)=   -844.658653
SCF(D3BJ) =   -844.858032304
SCF(BS2) =   -2288.36356986
SCF(BS2+D3BJ) =   -2288.42177209
Low Freq. = 53.0555cm-1, 60.4054cm-1

```
C   1.81974 -2.84965  0.00382
C   1.90647 -1.43104  0.00570
C   0.67876 -0.71526 -0.00258
N  -0.54241 -1.33067 -0.01174
C  -0.60139 -2.67114 -0.01343
C   0.56990 -3.46290 -0.00615
C   0.67540  0.71801 -0.00261
C   1.89975  1.43951  0.00562
C   3.13411  0.69559  0.01466
C   3.13736 -0.68138  0.01470
C   1.80639  2.85772  0.00367
C   0.55368  3.46511 -0.00620
C  -0.61392  2.66787 -0.01332
N  -0.54864  1.32770 -0.01166
Cu -2.02683 -0.00503 -0.00655
O  -3.81555 -0.00591 -0.06450
H  -1.59717 -3.12149 -0.02075
H   0.47381 -4.55081 -0.00829
H   2.73460 -3.44963  0.01002
H  -1.61178  3.11359 -0.02051
H   0.45251  4.55256 -0.00836
H   2.71848  3.46192  0.00980
H   4.07698  1.25028  0.02121
H   4.08284 -1.23163  0.02126
H  -4.18818 -0.00541  0.84220
```

$[Cu(Phen)_2I]^+$

SCF =   -1352.14070052
H(0 K)=   -1351.803168
H(298 K)=   -1351.777803
G(298 K)=   -1351.860974
SCF(D3BJ) =   -1352.27530802
SCF(BS2) =   -3082.24658735
SCF(BS2+D3BJ) =   -3082.38117838
Low Freq. = 11.7448cm-1, 15.9955cm-1

```
C  -3.35995  0.68098 -2.72307
C  -2.08777  0.82785 -2.12877
N  -1.77958  0.25751 -0.95312
C  -2.72606 -0.48357 -0.29986
C  -4.04017 -0.67063 -0.82288
C  -4.33812 -0.06296 -2.07075
C  -4.98287 -1.45916 -0.07242
C  -4.63433 -2.03029  1.12886
C  -3.31345 -1.86029  1.67667
C  -2.35909 -1.08220  0.95740
C  -2.88951 -2.42683  2.90833
C  -1.58977 -2.20595  3.35133
C  -0.71439 -1.41986  2.56688
N  -1.08551 -0.86889  1.40369
Cu  0.00067  0.34098 -0.00642
N   1.77926  0.27801  0.94413
C   2.72900 -0.47076  0.30466
C   4.04248 -0.64574  0.83331
C   4.33536 -0.01929  2.07304
C   3.35353  0.73138  2.71202
```

```
C   2.08269  0.86652  2.11203
C   4.98949 -1.44201  0.09666
C   4.64505 -2.03283 -1.09623
C   3.32446 -1.87640 -1.64882
C   2.36605 -1.09038 -0.94357
C   2.90457 -2.46410 -2.87192
C   1.60455 -2.25546 -3.31995
C   0.72498 -1.46012 -2.54972
N   1.09224 -0.88884 -1.39502
I  -0.00962  3.06003 -0.01848
H  -5.35925 -2.62707  1.69062
H  -5.98812 -1.59606 -0.48207
H   5.37319 -2.63582 -1.64699
H   5.99456 -1.56885  0.50990
H   0.31392 -1.23297  2.89055
H  -1.23185 -2.62886  4.29301
H  -3.58772 -3.03158  3.49516
H  -1.30384  1.42783 -2.59828
H  -3.55730  1.16142 -3.68401
H  -5.33439 -0.18354 -2.50725
H  -0.30329 -1.28254 -2.87855
H   1.24971 -2.69481 -4.25527
H   3.60570 -3.07532 -3.44862
H   1.29590  1.47168  2.56989
H   3.54740  1.22633  3.66628
H   5.33062 -0.13150  2.51382
```

[Cu(Phen)(OAc)I]

```
SCF  =       -1009.15156568
H(0 K)=      -1008.933241
H(298 K)=    -1008.913099
G(298 K)=    -1008.985585
SCF(D3BJ) =        -1009.23010941
SCF(BS2) =         -2739.15950022
SCF(BS2+D3BJ) =    -2739.23802890
Low Freq. = 14.9714cm-1,
28.7121cm-1
```

```
Cu  0.71147  0.36177  0.17266
N  -0.61305 -1.10859  0.58745
N  -1.02945  1.33997 -0.42932
C  -0.36828 -2.31490  1.12048
H   0.68041 -2.55041  1.32155
C  -1.40209 -3.23878  1.39208
H  -1.14955 -4.20644  1.83177
C  -2.71881 -2.90574  1.08845
H  -3.53337 -3.61032  1.28259
C  -3.00637 -1.63599  0.52085
C  -1.90429 -0.76167  0.29490
C  -2.12745  0.54788 -0.25762
C  -1.18745  2.57211 -0.92935
H  -0.27611  3.16901 -1.03069
C  -2.45763  3.07566 -1.29550
H  -2.53584  4.08657 -1.70272
C  -3.58611  2.27811 -1.12711
H  -4.57918  2.64856 -1.39989
C  -3.44651  0.96974 -0.59022
C  -4.54232  0.06260 -0.36344
H  -5.55358  0.38968 -0.62358
C  -4.33055 -1.18731  0.17181
H  -5.17187 -1.86540  0.34458
O   1.81045  2.03367  0.15603
O   1.42491  1.18199  2.17360
C   1.95247  2.08221  1.44990
C   2.78108  3.20388  2.03585
H   2.60505  4.14421  1.49145
H   2.55180  3.33393  3.10314
H   3.85109  2.95308  1.93380
I   2.56904 -1.18520 -0.98810
```

[Cu(Phen)$_2$(OAc)I]

```
SCF  =       -1580.77554049
H(0 K)=      -1580.389986
H(298 K)=    -1580.358411
G(298 K)=    -1580.456817
SCF(D3BJ) =        -1580.92805980
SCF(BS2) =         -3310.97794583
SCF(BS2+D3BJ) =    -3311.13052284
Low Freq. = 17.9166cm-1,
21.1489cm-1
```

```
Cu -0.06264 -0.47047  0.30620
O   0.80096 -1.36577  1.83042
O  -0.87828 -2.21897  3.13604
N  -1.79468  0.44128  1.14492
N  -0.75147  0.57898 -1.33607
N   1.25047  1.56073  0.83361
N   1.89846 -0.71527 -0.60875
C  -2.30068  0.34351  2.38394
H  -1.80646 -0.39097  3.03062
C  -3.42819  1.09099  2.80045
H  -3.79618  0.97440  3.82310
C  -4.05268  1.95231  1.90573
H  -4.92841  2.53803  2.20304
C  -3.54861  2.06450  0.58275
C  -2.40163  1.28212  0.25390
C  -1.84798  1.35018 -1.07287
C  -0.22656  0.60433 -2.56875
H   0.64409 -0.03285 -2.73887
C  -0.75894  1.40361 -3.60480
H  -0.28728  1.38353 -4.59024
C  -1.87115  2.19927 -3.35105
H  -2.30340  2.82917 -4.13482
C  -2.45313  2.18907 -2.05651
C  -3.60697  2.97031 -1.69426
H  -4.06056  3.61285 -2.45515
```

```
C  -4.13213  2.91170 -0.42487
H  -5.01054  3.50802 -0.15906
C   0.94020  2.65839  1.53050
H  -0.09105  2.72361  1.89639
C   1.86295  3.69633  1.80470
H   1.54663  4.56956  2.38145
C   3.16296  3.57413  1.33043
H   3.90965  4.35175  1.52187
C   3.53170  2.42013  0.58853
C   2.52124  1.42958  0.36338
C   2.86038  0.22985 -0.38648
C   2.21954 -1.83050 -1.27863
H   1.40092 -2.54908 -1.41467
C   3.51528 -2.07764 -1.78429
H   3.71620 -3.00610 -2.32475
C   4.50574 -1.12546 -1.58158
H   5.52168 -1.27838 -1.96006
C   4.19920  0.06407 -0.86940
C   5.18230  1.08601 -0.62427
H   6.19587  0.93468 -1.00913
C   4.86100  2.22246  0.07594
H   5.61350  2.99599  0.26038
C   0.32161 -2.06913  2.82675
C   1.42972 -2.75046  3.64522
H   1.00407 -3.22943  4.53884
H   2.19696 -2.01820  3.94551
H   1.93222 -3.51491  3.02856
I  -1.43572 -2.87695 -0.89542
```

[Cu(Phen)$_2$I(OH)]

```
SCF  =      -1428.09962371
H(0 K)=     -1427.751206
H(298 K)=   -1427.723172
G(298 K)=   -1427.812501
SCF(D3BJ) =       -1428.23960171
SCF(BS2) =        -3158.24267929
SCF(BS2+D3BJ) =   -3158.38268968
Low Freq. = 16.5138cm-1,
21.1023cm-1
```

```
C   5.12188 -1.73174  0.01474
C   3.80048 -2.16419 -0.35391
C   2.70988 -1.23522 -0.33070
C   2.95940  0.14202  0.06609
C   4.29074  0.53505  0.42002
C   5.35701 -0.43103  0.38651
N   1.44032 -1.58763 -0.67160
C   1.20891 -2.84997 -1.04486
C   2.21522 -3.84436 -1.10096
C   3.51479 -3.49747 -0.75265
C   4.50644  1.88697  0.79643
C   3.43705  2.77310  0.80802
C   2.15639  2.30082  0.44475
N   1.91885  1.03010  0.08858
Cu -0.01051  0.40675 -0.62299
I  -1.53410  2.99205 -0.28336
O   0.38224  0.86952 -2.43580
N  -1.72949 -0.67920 -1.17375
C  -2.21144 -0.85400 -2.40838
C  -3.38803 -1.60067 -2.65507
C  -4.07167 -2.17284 -1.58490
C  -3.57958 -1.99674 -0.26312
C  -2.38704 -1.22954 -0.11679
C  -1.82623 -0.99646  1.18980
C  -2.46942 -1.54178  2.34015
C  -3.67041 -2.31936  2.16548
C  -4.20486 -2.53784  0.91633
C  -1.87519 -1.27509  3.60201
C  -0.71667 -0.50682  3.66058
C  -0.15348 -0.00611  2.46429
N  -0.68870 -0.24035  1.25835
H  -1.62315 -0.37171 -3.19746
H  -3.74571 -1.71999 -3.68109
H  -4.98431 -2.75475 -1.74910
H   0.75416  0.60385  2.48056
H  -0.23409 -0.28132  4.61479
H  -2.33261 -1.67337  4.51322
H  -4.15522 -2.73354  3.05510
H  -5.11919 -3.12826  0.80029
H   0.17445 -3.09141 -1.31614
H   1.96206 -4.86073 -1.41454
H   4.32352 -4.23506 -0.78226
H   1.28104  2.96253  0.43223
H   3.56595  3.82093  1.09101
H   5.51456  2.21406  1.07135
H   6.36370 -0.10291  0.66458
H   5.93787 -2.46137 -0.00978
H  -0.06092  1.74168 -2.49528
```

[Cu(Phen)I$_2$]

```
SCF  =      -792.145554849
H(0 K)=     -791.976021
H(298 K)=   -791.959331
G(298 K)=   -792.024370
SCF(D3BJ) =       -792.220083994
SCF(BS2) =        -2808.44443891
SCF(BS2+D3BJ) =   -2808.51894117
Low Freq. = 21.7944cm-1,
28.3934cm-1
```

```
Cu -0.47876 -0.00002 -0.00354
N   1.08329 -1.26744 -0.45137
N   1.08115  1.27079  0.44623
C   1.04469 -2.52305 -0.92033
H   0.04980 -2.95966 -1.04866
C   2.21863 -3.25246 -1.21534
```

```
H   2.13107 -4.27102 -1.60068
C   3.46199 -2.66594 -1.00165
H   4.38482 -3.21492 -1.21332
C   3.53294 -1.33858 -0.50070
C   2.29812 -0.67368 -0.24805
C   2.29691  0.67827  0.24489
C   1.04057  2.52577  0.91666
H   0.04500  2.96112  1.04390
C   2.21324  3.25595  1.21500
H   2.12383  4.27384  1.60158
C   3.45753  2.67081  1.00278
H   4.37967  3.22014  1.21653
C   3.53054  1.34397  0.50089
C   4.76436  0.64689  0.24405
H   5.70667  1.16616  0.44291
C   4.76558 -0.64044 -0.24067
H   5.70902 -1.15872 -0.43668
I  -2.20714  1.76387 -0.83200
I  -2.19942 -1.76773  0.83479
```

[Cu(Phen)I(OH)]

```
SCF  =      -856.464823170
H(0 K)=     -856.283233
H(298 K)=   -856.266723
G(298 K)=   -856.329249
SCF(D3BJ) =       -856.532672917
SCF(BS2) =        -2586.41507879
SCF(BS2+D3BJ) =   -2586.48291111
Low Freq. = 20.4451cm-1,
46.0275cm-1

Cu -0.76541 -0.67073  0.59470
N   1.11015 -1.44611  0.09477
N   0.23310  1.07379  0.40222
C   1.50008 -2.71705 -0.07931
H   0.72953 -3.48490  0.03793
C   2.83341 -3.05880 -0.40101
H   3.09888 -4.11031 -0.53347
C   3.78353 -2.05150 -0.54100
H   4.82288 -2.29189 -0.78542
C   3.39758 -0.69540 -0.36742
C   2.03038 -0.44723 -0.05145
C   1.56257  0.90230  0.12145
C  -0.23263  2.32078  0.57864
H  -1.30349  2.40826  0.78313
C   0.60090  3.45744  0.48779
H   0.16895  4.44845  0.64544
C   1.95116  3.29750  0.19172
H   2.61421  4.16408  0.10722
C   2.47264  1.99114 -0.00411
C   3.85133  1.71327 -0.31607
H   4.54260  2.55610 -0.41105
C   4.29572  0.42345 -0.49352
H   5.34457  0.22610 -0.73469
I  -3.07103  0.13255 -0.52998
O  -1.49989 -2.12646  1.47141
H  -0.74035 -2.68748  1.72916
```

[Cu(Phen)I]$^{+}$

```
SCF  =      -780.469079659
H(0 K)=     -780.299547
H(298 K)=   -780.285321
G(298 K)=   -780.343246
SCF(D3BJ) =       -780.531253986
SCF(BS2) =        -2510.38428047
SCF(BS2+D3BJ) =   -2510.44645484
Low Freq. = 20.6521cm-1,
35.0272cm-1

N   0.59922 -1.33837 -0.00004
C   1.81765 -0.71572  0.00018
C   3.04585 -1.43170  0.00039
C   2.96096 -2.85042  0.00038
C   1.71308 -3.46713  0.00014
C   0.53978 -2.67825 -0.00006
C   4.27674 -0.68245  0.00060
C   4.27407  0.69416  0.00061
C   3.04030  1.43864  0.00039
C   1.81487  0.71791  0.00018
N   0.59407  1.33586 -0.00004
C   0.52935  2.67548 -0.00006
C   1.69962  3.46890  0.00015
C   2.94989  2.85703  0.00038
Cu -0.87330 -0.00424 -0.00020
H  -0.46760  3.12286 -0.00025
H   1.60131  4.55659  0.00013
H   3.86366  3.45864  0.00055
H  -0.45544 -3.12950 -0.00025
H   1.61897 -4.55519  0.00011
H   3.87704 -3.44849  0.00054
H   5.22188 -1.23324  0.00076
H   5.21706  1.24860  0.00077
I  -3.31089 -0.00073 -0.00029
```

[Cu(Phen)$_2$Cl]$^{+}$

```
SCF  =      -1355.70609695
H(0 K)=     -1355.368226
H(298 K)=   -1355.343108
G(298 K)=   -1355.424461
SCF(D3BJ) =       -1355.83548286
SCF(BS2) =        -3244.65634343
SCF(BS2+D3BJ) =   -3244.78572935
Low Freq. = 15.5027cm-1,
18.1734cm-1
```

```
C  -3.42670  2.52567  1.44031
C  -2.13878  1.95570  1.54462
N  -1.78802  0.86182  0.85181
C  -2.70188  0.26205  0.03044
C  -4.02579  0.76892 -0.12855
C  -4.36989  1.93478  0.60520
C  -4.93215  0.08340 -1.01305
C  -4.54114 -1.04412 -1.69627
C  -3.21258 -1.57981 -1.54787
C  -2.29220 -0.92340 -0.67852
C  -2.74995 -2.74289 -2.21953
C  -1.44906 -3.18513 -2.00425
C  -0.61107 -2.46791 -1.11877
N  -1.01747 -1.36851 -0.47090
Cu  0.01491 -0.03852  0.90295
N   1.83004 -0.91250  0.79205
C   2.73514 -0.23009  0.02751
C   4.06233 -0.70418 -0.18768
C   4.42080 -1.93308  0.42703
C   3.48641 -2.61125  1.20370
C   2.19370 -2.06657  1.37083
C   4.95393  0.07593 -1.00665
C   4.54413  1.25936 -1.57528
C   3.20937  1.76027 -1.37032
C   2.30574  1.00954 -0.56301
C   2.72314  2.97351 -1.92640
C   1.41537  3.36930 -1.66611
C   0.59311  2.55750 -0.85086
N   1.02398  1.41028 -0.30972
Cl -0.11833 -0.34485  3.23219
H  -5.23854 -1.55735 -2.36529
H  -5.94437  0.48173 -1.13025
H   5.23068  1.84391 -2.19521
H   5.97025 -0.29553 -1.16841
H   0.41607 -2.79448 -0.93005
H  -1.06261 -4.07612 -2.50492
H  -3.42074 -3.27874 -2.89818
H  -1.37618  2.37976  2.20315
H  -3.66228  3.42065  2.02062
H  -5.37538  2.35606  0.51006
H  -0.43924  2.84479 -0.63154
H   1.01039  4.29614 -2.07920
H   3.38023  3.58399 -2.55331
H   1.43746 -2.55669  1.98942
H   3.73275 -3.55693  1.69187
H   5.42917 -2.33425  0.28652
```

[Cu(Phen)(OAc)Cl]

```
SCF   =       -1012.71782192
H(0 K)=       -1012.498929
H(298 K)=     -1012.479184
G(298 K)=     -1012.548661
SCF(D3BJ) =         -1012.79307930
SCF(BS2) =          -2901.57052661
SCF(BS2+D3BJ) =     -2901.64578399
Low Freq. = 26.2647cm-1,
34.0444cm-1

Cu -1.18390 -0.38649  0.32649
N   0.57064 -1.35885 -0.04538
N   0.08828  1.23386  0.39114
C   0.76615 -2.66613 -0.26797
H  -0.12292 -3.29959 -0.20176
C   2.04605 -3.19383 -0.55503
H   2.15202 -4.26660 -0.73274
C   3.14682 -2.34347 -0.60185
H   4.14682 -2.73248 -0.81746
C   2.96783 -0.95404 -0.36557
C   1.64245 -0.51118 -0.09309
C   1.38412  0.88208  0.14638
C  -0.20773  2.52044  0.61670
H  -1.26445  2.74220  0.79227
C   0.78665  3.52639  0.61693
H   0.49821  4.56241  0.80995
C   2.11263  3.18275  0.36833
H   2.89666  3.94637  0.36068
C   2.45032  1.82492  0.11932
C   3.78342  1.35201 -0.15457
H   4.60121  2.07868 -0.17457
C   4.03107  0.01832 -0.38809
H   5.04789 -0.32743 -0.59721
O  -2.78757  0.80081  0.35322
O  -2.35647  0.05022 -1.70912
C  -3.06872  0.73409 -0.92148
C  -4.29687  1.49122 -1.39266
H  -4.32826  2.49728 -0.94586
H  -4.30492  1.56488 -2.48931
H  -5.20362  0.95507 -1.06399
Cl -2.26534 -2.21337  1.25028
```

[Cu(Phen)$_2$(OAc)Cl]

```
SCF   =       -1584.34202908
H(0 K)=       -1583.956261
H(298 K)=     -1583.924842
G(298 K)=     -1584.022145
SCF(D3BJ) =         -1584.48860412
SCF(BS2) =          -3473.38685859
SCF(BS2+D3BJ) =     -3473.53343363
Low Freq. = 17.0515cm-1,
19.3747cm-1

C  -4.32113 -2.00546  1.58086
C  -2.96532 -1.72377  1.96971
C  -2.11298 -0.96725  1.10178
C  -2.63883 -0.49220 -0.16783
C  -3.99531 -0.78915 -0.51831
```

```
C  -4.81642 -1.55439  0.38234
N  -0.82277 -0.66929  1.41883
C  -0.33937 -1.10131  2.58764
C  -1.09791 -1.85536  3.51539
C  -2.41514 -2.16675  3.20237
C  -4.48161 -0.30683 -1.76222
C  -3.64137  0.42576 -2.59061
C  -2.31508  0.67362 -2.17259
N  -1.83042  0.22844 -1.00385
Cu  0.09998  0.77269 -0.35841
N   0.93396 -0.98697 -1.11480
C   2.18759 -1.21770 -0.62593
C   2.94003 -2.38657 -0.94703
C   2.33368 -3.32804 -1.81998
C   1.06044 -3.07442 -2.31888
C   0.38941 -1.88863 -1.94123
C   4.25413 -2.54769 -0.38079
C   4.78966 -1.59122  0.44969
C   4.05761 -0.39496  0.77716
C   2.75073 -0.21310  0.23636
N   1.99262  0.89839  0.48559
C   2.50096  1.86597  1.26446
C   3.78529  1.76311  1.84946
C   4.56345  0.63591  1.61282
O  -0.84280  2.27806  0.53895
C  -0.53082  3.53783  0.71465
C  -1.70493  4.36314  1.26421
O   0.57242  4.07897  0.48593
Cl  0.81156  1.90338 -2.81914
H   1.87419  2.75868  1.37186
H   4.14972  2.58004  2.47744
H   5.56081  0.53620  2.05280
H  -0.61153 -1.66278 -2.31721
H   0.56658 -3.77529 -2.99629
H   2.87337 -4.24005 -2.09349
H   4.82212 -3.44981 -0.62833
H   5.78996 -1.72144  0.87420
H   0.70215 -0.83902  2.80749
H  -0.64437 -2.17934  4.45592
H  -3.03733 -2.74767  3.89103
H  -1.60057  1.24085 -2.78289
H  -3.98349  0.81192 -3.55411
H  -5.51591 -0.51860 -2.05240
H  -5.84884 -1.76831  0.08832
H  -4.95146 -2.58592  2.26228
H  -1.37312  5.38198  1.51116
H  -2.13065  3.88339  2.16072
H  -2.50882  4.41603  0.51041
```

[Cu(Phen)$_2$Cl(OH)]

```
SCF  =        -1431.66543399
H(0 K)=       -1431.316745
H(298 K)=     -1431.288896
G(298 K)=     -1431.376814
SCF(D3BJ) =         -1431.79956896
SCF(BS2) =          -3320.65080154
SCF(BS2+D3BJ) =     -3320.78493651
Low Freq. = 16.4647cm-1,
20.4802cm-1

N   0.89714 -0.50633  1.15030
C   2.16798 -0.00240  1.14271
C   2.92583  0.22329  2.32930
C   2.30417 -0.10058  3.56449
C   1.01168 -0.61545  3.56226
C   0.34033 -0.80429  2.33171
C   4.26006  0.75630  2.21399
C   4.81098  1.04344  0.98586
C   4.07102  0.82137 -0.23012
C   2.74745  0.30076 -0.14082
N   1.97447  0.05910 -1.23511
C   2.46483  0.30952 -2.45366
C   3.76785  0.82797 -2.64338
C   4.57027  1.08564 -1.53455
Cu  0.08347 -0.69452 -0.79159
O  -0.37821 -0.82838 -2.64917
N  -1.03562  1.47162 -0.46638
C  -2.33753  1.24426 -0.13901
C  -3.28620  2.30343  0.03470
C  -2.82117  3.63312 -0.14969
C  -1.49109  3.85278 -0.48658
C  -0.63399  2.73548 -0.63431
C  -2.77300 -0.13249  0.03384
C  -4.13902 -0.39667  0.37252
C  -5.05862  0.69747  0.54221
C  -4.64895  1.99810  0.37983
C  -4.53387 -1.75171  0.52858
C  -3.59870 -2.76316  0.34874
C  -2.27088 -2.41594  0.01455
N  -1.86830 -1.14469 -0.13496
Cl  0.91919 -3.36361 -0.92387
H   1.77500  0.07467 -3.27265
H   4.12885  1.01979 -3.65713
H   5.58160  1.48687 -1.65565
H  -0.67574 -1.20825  2.30088
H   0.50473 -0.87697  4.49442
H   2.84571  0.05587  4.50278
H   4.83268  0.92717  3.13103
H   5.82642  1.44574  0.91465
H   0.42024  2.87604 -0.90061
H  -1.10181  4.86326 -0.63752
H  -3.51760  4.46890 -0.02535
H  -1.49076 -3.17000 -0.15015
H  -3.86908 -3.81642  0.45969
H  -5.57265 -1.98236  0.78692
H  -6.09653  0.46774  0.80349
H  -5.35458  2.82531  0.50844
```

H -0.12849 -1.76729 -2.78404

[Cu(Phen)Cl_2]

SCF = -799.277898076
H(0 K)= -799.107242
H(298 K)= -799.091347
G(298 K)= -799.151148
SCF(D3BJ) = -799.345082578
SCF(BS2) = -3133.26504920
SCF(BS2+D3BJ) = -3133.33223370
Low Freq. = 40.4353cm-1, 48.3183cm-1

Cu	-1.52553	0.00000	0.00003
N	0.02977	-1.33396	-0.08699
N	0.02978	1.33395	0.08704
C	-0.02119	-2.66919	-0.19166
H	-1.02190	-3.11004	-0.19884
C	1.15016	-3.45691	-0.27149
H	1.05630	-4.54164	-0.36123
C	2.39801	-2.84258	-0.22823
H	3.31604	-3.43574	-0.28111
C	2.47868	-1.42885	-0.11279
C	1.24799	-0.71576	-0.05209
C	1.24799	0.71575	0.05210
C	-0.02119	2.66918	0.19172
H	-1.02190	3.11003	0.19894
C	1.15016	3.45690	0.27153
H	1.05631	4.54163	0.36127
C	2.39802	2.84257	0.22822
H	3.31604	3.43574	0.28108
C	2.47869	1.42884	0.11277
C	3.71170	0.68583	0.05579
H	4.65532	1.23771	0.10269
C	3.71170	-0.68584	-0.05585
H	4.65532	-1.23772	-0.10278
Cl	-3.05210	1.54231	-0.69338
Cl	-3.05215	-1.54229	0.69331

[Cu(Phen)Cl(OH)]

SCF = -860.030832194
H(0 K)= -859.848681
H(298 K)= -859.832552
G(298 K)= -859.892405
SCF(D3BJ) = -860.095541464
SCF(BS2) = -2748.82516628
SCF(BS2+D3BJ) = -2748.88987555
Low Freq. = 32.8764cm-1, 57.6426cm-1

Cu	1.62208	-0.52392	-0.17330
N	-0.22640	-1.42460	0.02638
N	0.46645	1.15576	-0.10295
C	-0.52381	-2.72824	0.12940
H	0.32333	-3.42022	0.13601
C	-1.85628	-3.18853	0.22862
H	-2.04563	-4.26129	0.31117
C	-2.90229	-2.27019	0.21762
H	-3.94148	-2.60569	0.28849
C	-2.61513	-0.88228	0.11912
C	-1.24263	-0.51160	0.03026
C	-0.87105	0.87499	-0.04617
C	0.85351	2.43837	-0.17008
H	1.93379	2.60762	-0.19442
C	-0.07456	3.50417	-0.19250
H	0.29419	4.53087	-0.25313
C	-1.43793	3.23126	-0.13195
H	-2.17335	4.04162	-0.14334
C	-1.87707	1.88256	-0.05152
C	-3.25926	1.48286	0.02551
H	-4.02796	2.26162	0.01955
C	-3.61399	0.15586	0.10972
H	-4.66753	-0.13262	0.17374
Cl	3.53191	0.50931	0.69194
O	2.52736	-1.99799	-0.85811
H	1.84549	-2.55625	-1.28271

[Cu(Phen)Cl]$^+$

SCF = -784.031537318
H(0 K)= -783.861696
H(298 K)= -783.847718
G(298 K)= -783.903700
SCF(D3BJ) = -784.091392261
SCF(BS2) = -2672.79138526
SCF(BS2+D3BJ) = -2672.85124021
Low Freq. = 29.1097cm-1, 37.7922cm-1

N	-0.19357	-1.37186	-0.00004
C	0.98080	-0.67068	0.00015
C	2.25348	-1.30103	0.00033
C	2.26160	-2.72252	0.00030
C	1.05641	-3.41992	0.00011
C	-0.16802	-2.71254	-0.00006
C	3.42732	-0.46429	0.00052
C	3.32602	0.90925	0.00053
C	2.04274	1.56533	0.00034
C	0.87614	0.75504	0.00014
N	-0.38683	1.27898	-0.00006
C	-0.55954	2.60807	-0.00007
C	0.54899	3.48662	0.00012
C	1.84315	2.97265	0.00032
Cu	-1.74518	-0.15848	-0.00027
H	-1.58839	2.97666	-0.00023
H	0.36666	4.56335	0.00011

H   2.70790 3.64293 0.00047
H -1.13167 -3.22823 -0.00021
H   1.03376 -4.51172 0.00008
H   3.21432 -3.26042 0.00043
H   4.40970 -0.94510 0.00066
H   4.22663 1.52966 0.00067
Cl -3.87648 -0.09159 -0.00057

8^+
SCF =   -1572.24994018
H(0 K)=   -1571.827106
H(298 K)=   -1571.797821
G(298 K)=   -1571.889353
SCF(D3BJ) =   -1572.40352236
SCF(BS2) =   -3016.05292011
SCF(BS2+D3BJ) =   -3016.20655646
Low Freq. = 13.0240cm-1,
18.6824cm-1

Cu 0.07935 0.45870 -0.29310
N -1.72152 -0.20699 -1.12938
N -0.88801 -0.59194 1.44469
N   1.21962 -1.21662 -1.01968
N   1.96829 0.89331 0.48317
C -2.12288 0.01427 -2.39061
H -1.40764 0.52213 -3.04440
C -3.39484 -0.37448 -2.86407
H -3.66517 -0.17644 -3.90416
C -4.27828 -1.00364 -1.99290
H -5.27224 -1.31553 -2.32891
C -3.88664 -1.24175 -0.64920
C -2.57872 -0.81842 -0.25665
C -2.13504 -1.03131 1.10557
C -0.46879 -0.77835 2.70310
H   0.53616 -0.41098 2.93743
C -1.25605 -1.40979 3.69407
H -0.85726 -1.53471 4.70402
C -2.52926 -1.85806 3.35970
H -3.16706 -2.34826 4.10213
C -3.00930 -1.67455 2.03563
C -4.31664 -2.09696 1.60597
H -4.97166 -2.58787 2.33272
C -4.73931 -1.88624 0.31543
H -5.73602 -2.20695 -0.00321
C   0.82679 -2.25967 -1.76510
H -0.22450 -2.26984 -2.06795
C   1.70255 -3.29964 -2.15237
H   1.32268 -4.12210 -2.76350
C   3.03304 -3.25533 -1.74875
H   3.73447 -4.04573 -2.03387
C   3.48409 -2.16691 -0.95579
C   2.52491 -1.16622 -0.61697
C   2.92462 -0.03652 0.18558
C   2.30687 1.95380 1.23075
H   1.50700 2.67378 1.42843
C   3.61603 2.14323 1.72782
H   3.83594 3.02362 2.33667
C   4.60171 1.20760 1.42948
H   5.62493 1.33239 1.79767
C   4.27389 0.07616 0.63621
C   5.21903 -0.94901 0.27599
H   6.25118 -0.85292 0.62683
C   4.83918 -2.02736 -0.48825
H   5.56528 -2.80140 -0.75555
C -0.49567 2.36005 -0.40176
C -1.25362 2.95546 0.62906
C -0.14162 3.15346 -1.51501
C -1.64230 4.30913 0.55541
C -0.53195 4.50575 -1.59519
C -1.28251 5.08634 -0.55846
H -1.55177 2.36645 1.50601
H   0.44968 2.72744 -2.33612
H -2.22874 4.75236 1.36923
H -0.24555 5.10406 -2.46848
H -1.58526 6.13744 -0.61873

[Cu(Phen)$_2$(OAc)(Ph)]

SCF =   -1800.87457707
H(0 K)=   -1800.403879
H(298 K)=   -1800.368418
G(298 K)=   -1800.474844
SCF(D3BJ) =   -1801.04705795
SCF(BS2) =   -3244.77662719
SCF(BS2+D3BJ) =   -3244.94922026
Low Freq. = 16.0849cm-1,
18.8843cm-1

Cu -0.05175 0.51431 0.16171
O -1.22614 1.67817 1.44795
O   0.07869 1.78339 3.32673
N   1.97460 -0.35792 1.26124
N   0.94874 -0.95589 -1.21232
N -1.26786 -1.12465 0.85191
N -2.08311 0.50635 -1.21034
C   2.49186 -0.00276 2.44590
H   1.81426 0.59138 3.07466
C   3.80523 -0.34816 2.84832
H   4.17321 -0.03485 3.82966
C   4.60224 -1.09285 1.98630
H   5.61873 -1.38868 2.26716
C   4.09010 -1.47285 0.71688
C   2.75565 -1.06320 0.39674
C   2.20235 -1.39993 -0.90335
C   0.43336 -1.27775 -2.40643
H -0.57463 -0.89822 -2.60326
C   1.12362 -2.05013 -3.36829
H   0.64417 -2.28172 -4.32322

```
C    2.40788 -2.49408 -3.07578
H    2.97974 -3.08642 -3.79775
C    2.98720 -2.17227 -1.82010
C    4.31584 -2.58535 -1.45104
H    4.89607 -3.17446 -2.16883
C    4.84998 -2.24220 -0.23266
H    5.86517 -2.55158  0.03766
C   -0.85952 -1.95740  1.81893
H    0.18154 -1.83810  2.13423
C   -1.69873 -2.92586  2.41501
H   -1.30276 -3.57533  3.20008
C   -3.01922 -3.01949  1.99404
H   -3.70522 -3.74649  2.44110
C   -3.48698 -2.15559  0.96884
C   -2.55819 -1.22079  0.40847
C   -2.99948 -0.33586 -0.65597
C   -2.49367  1.35322 -2.16066
H   -1.72463  2.01916 -2.57045
C   -3.82685  1.40836 -2.63396
H   -4.09967  2.12412 -3.41432
C   -4.76254  0.53101 -2.09869
H   -5.80060  0.53040 -2.44732
C   -4.36619 -0.37855 -1.08174
C   -5.27330 -1.32256 -0.48454
H   -6.31195 -1.34187 -0.83074
C   -4.84620 -2.18512  0.49529
H   -5.53697 -2.90710  0.94279
C   -0.94444  2.06745  2.65159
C   -2.00286  3.00717  3.26497
H   -1.85594  3.10909  4.35091
H   -3.02275  2.64365  3.05925
H   -1.91479  4.00731  2.80468
C    0.91540  2.07424 -0.59005
C    1.09273  2.25051 -1.98157
C    1.41828  3.08875  0.25865
C    1.73296  3.39119 -2.51002
C    2.06895  4.22635 -0.25870
C    2.22606  4.38427 -1.64752
H    0.73370  1.48503 -2.68257
H    1.29868  2.98959  1.34488
H    1.84885  3.49895 -3.59597
H    2.45203  4.99425  0.42563
H    2.72829  5.27052 -2.05195
```

[Cu(Phen)$_2$(Ph)(OH)]

```
SCF  =       -1648.18807628
H(0 K)=      -1647.754588
H(298 K)=    -1647.722822
G(298 K)=    -1647.819529
SCF(D3BJ) =        -1648.34717237
SCF(BS2) =         -3092.03199871
SCF(BS2+D3BJ) =    -3092.19108198
```

```
Low Freq. = 15.5369cm-1,
19.9691cm-1

Cu   0.07978  0.24233 -0.72118
O    0.75986  0.23323 -2.52585
N   -1.98614 -1.43639 -0.96939
N   -0.81910 -0.12920  1.17202
N    1.51240 -1.36039 -0.30193
N    2.35940  1.21387  0.29184
C   -2.57411 -2.00699 -2.02753
H   -1.93329 -2.17140 -2.90288
C   -3.93318 -2.40093 -2.05642
H   -4.35121 -2.86140 -2.95598
C   -4.70623 -2.20337 -0.91776
H   -5.75814 -2.50682 -0.88841
C   -4.12206 -1.60019  0.22738
C   -2.74283 -1.21379  0.14320
C   -2.11886 -0.55873  1.27957
C   -0.24091  0.42982  2.25118
H    0.79741  0.74804  2.11028
C   -0.90462  0.61641  3.48161
H   -0.36965  1.06882  4.32108
C   -2.23421  0.22409  3.59550
H   -2.79097  0.36797  4.52719
C   -2.87775 -0.37706  2.48166
C   -4.25287 -0.80027  2.53208
H   -4.81061 -0.64155  3.46099
C   -4.85715 -1.37927  1.44343
H   -5.90709 -1.68797  1.48523
C    1.10681 -2.61810 -0.52661
H    0.03069 -2.73559 -0.69628
C    1.98399 -3.72550 -0.56045
H    1.58735 -4.72720 -0.74645
C    3.34184 -3.50736 -0.36657
H    4.05892 -4.33440 -0.39960
C    3.80884 -2.18887 -0.12262
C    2.84229 -1.13027 -0.08235
C    3.29335  0.22631  0.19637
C    2.78217  2.46372  0.51103
H    1.99937  3.22993  0.57229
C    4.14421  2.81896  0.66289
H    4.42210  3.86205  0.83805
C    5.10249  1.81506  0.59890
H    6.16674  2.03967  0.72686
C    4.69601  0.47431  0.36350
C    5.63448 -0.61394  0.29810
H    6.69890 -0.39682  0.43643
C    5.20472 -1.89833  0.07407
H    5.91902 -2.72724  0.03324
C   -1.00711  1.89673 -0.96188
C   -0.95983  2.94945 -0.01777
C   -1.80131  2.11197 -2.11416
C   -1.65656  4.15900 -0.21202
C   -2.51120  3.31293 -2.31686
```

```
C  -2.43709  4.34529 -1.36628
H  -0.37672  2.82916  0.90610
H  -1.87855  1.32570 -2.87732
H  -1.59184  4.95481  0.54087
H  -3.12234  3.44094 -3.21922
H  -2.98382  5.28238 -1.52084
H   0.31545  1.00546 -2.92560
```

[Cu(Phen)(Ph)(OH)]

```
SCF  =      -1076.57384901
H(0 K)=     -1076.306683
H(298 K)=   -1076.286435
G(298 K)=   -1076.356786
SCF(D3BJ) =       -1076.66028386
SCF(BS2) =        -2520.22286824
SCF(BS2+D3BJ) =   -2520.30933676
Low Freq. = 18.7500cm-1,
25.5573cm-1

Cu  0.75008 -0.89124 -0.26230
N  -0.15007  1.02982 -0.22458
N  -1.28347 -1.38184 -0.02987
C   0.45032  2.22774 -0.29385
H   1.53601  2.20835 -0.43435
C  -0.26258  3.44401 -0.19661
H   0.27958  4.39018 -0.27055
C  -1.64325  3.41787 -0.01552
H  -2.21833  4.34626  0.05912
C  -2.30948  2.16689  0.07124
C  -1.50452  0.99457 -0.03567
C  -2.11797 -0.30585  0.04663
C  -1.81253 -2.61234  0.02075
H  -1.09070 -3.43106 -0.06246
C  -3.20165 -2.83194  0.16639
H  -3.58334 -3.85534  0.20675
C  -4.06133 -1.74008  0.26709
H  -5.13949 -1.88404  0.39064
C  -3.52945 -0.42423  0.20730
C  -4.31961  0.77667  0.30512
H  -5.40261  0.68093  0.43204
C  -3.73265  2.02008  0.24610
H  -4.34357  2.92489  0.32560
C   2.61701 -0.33288  0.04917
C   3.37787  0.28587 -0.97292
C   3.25625 -0.53273  1.29707
C   4.70793  0.70098 -0.75864
C   4.58506 -0.12059  1.52111
C   5.31537  0.49954  0.49243
H   2.93153  0.45454 -1.96273
H   2.71432 -1.01682  2.12060
H   5.26880  1.18014 -1.57052
H   5.05007 -0.28558  2.50091
H   6.34923  0.81969  0.66349
O   1.11858 -2.66481 -0.83054
H   2.09369 -2.70519 -0.86968
```

[Cu(Phen)(OAc)(Ph)]

```
SCF  =      -1229.25862268
H(0 K)=     -1228.954577
H(298 K)=   -1228.930636
G(298 K)=   -1229.010832
SCF(D3BJ) =       -1229.35697159
SCF(BS2) =        -2672.96558856
SCF(BS2+D3BJ) =   -2673.06396371
Low Freq. = 17.6519cm-1,
28.2232cm-1

Cu  0.68161  0.41440 -0.15336
N  -0.46794 -1.27775  0.34455
N  -1.25956  1.14404 -0.48643
C  -0.03561 -2.47788  0.75550
H   1.04915 -2.59056  0.84198
C  -0.91976 -3.54074  1.05213
H  -0.51359 -4.49767  1.38923
C  -2.29108 -3.35047  0.90980
H  -2.99590 -4.15823  1.13108
C  -2.77952 -2.09065  0.47103
C  -1.81210 -1.07876  0.20101
C  -2.23860  0.22397 -0.24343
C  -1.61788  2.36818 -0.89881
H  -0.79222  3.06705 -1.06523
C  -2.96899  2.73599 -1.09730
H  -3.20559  3.74689 -1.43861
C  -3.97480  1.80473 -0.85494
H  -5.02811  2.06469 -1.00085
C  -3.62669  0.50048 -0.41144
C  -4.58305 -0.53958 -0.13007
H  -5.64721 -0.31958 -0.26021
C  -4.17599 -1.78425  0.29247
H  -4.91233 -2.56611  0.50306
C   2.40306 -0.50542 -0.40264
C   3.49470 -0.25946  0.46078
C   2.59479 -1.42977 -1.45620
C   4.73063 -0.91181  0.28368
C   3.83255 -2.07787 -1.64679
C   4.90406 -1.82069 -0.77499
H   3.37851  0.45008  1.28965
H   1.77201 -1.66027 -2.14619
H   5.56002 -0.70693  0.97183
H   3.95592 -2.78577 -2.47558
H   5.86588 -2.32585 -0.91793
O   1.42986  2.32976 -0.32187
O   1.11823  1.85998  1.86108
C   1.48479  2.64193  0.93999
C   2.05187  4.02324  1.26559
H   1.61986  4.78838  0.60113
```

```
H   1.85790  4.28682  2.31528
H   3.14248  4.02062  1.09588
```

[Cu(Phen)(Ph)]$^{+}$

```
SCF  =       -1000.59203936
H(0 K)=      -1000.336513
H(298 K)=    -1000.318532
G(298 K)=    -1000.384827
SCF(D3BJ) =        -1000.67310968
SCF(BS2) =         -2444.20161430
SCF(BS2+D3BJ) =    -2444.28267638
Low Freq. = 9.7235cm-1, 26.0202cm-1
```

```
Cu -0.84558 -0.63240 -0.00670
N   0.97123 -1.48392 -0.01404
N   0.21470  1.10335  0.00480
C   1.31348 -2.78227 -0.02616
H   0.49278 -3.50474 -0.03536
C   2.66210 -3.20423 -0.02691
H   2.88683 -4.27303 -0.03710
C   3.67819 -2.25396 -0.01489
H   4.72930 -2.55829 -0.01508
C   3.34688 -0.87199 -0.00264
C   1.96315 -0.53955 -0.00310
C   1.55684  0.84157  0.00684
C  -0.21318  2.37317  0.01089
H  -1.29559  2.52389  0.00858
C   0.69060  3.46021  0.01911
H   0.29719  4.47927  0.02288
C   2.06023  3.21486  0.02249
H   2.77616  4.04244  0.02879
C   2.53594  1.87552  0.01662
C   3.92933  1.51072  0.01878
H   4.67661  2.30961  0.02764
C   4.31878  0.19115  0.00951
H   5.38029 -0.07349  0.01028
C  -2.69418 -0.20934  0.00188
C  -3.40670 -0.21622  1.22286
C  -3.39612 -0.06011 -1.21572
C  -4.81345 -0.16997  1.21656
C  -4.80279 -0.01367 -1.21553
C  -5.50901 -0.06739 -0.00090
H  -2.87778 -0.27719  2.18121
H  -2.85861  0.00061 -2.16926
H  -5.36171 -0.20280  2.16442
H  -5.34311  0.07483 -2.16438
H  -6.60256 -0.00864 -0.00185
```

[Cu(Phen)$_2$I(Ph)]

```
SCF  =       -1583.88825910
H(0 K)=      -1583.465671
H(298 K)=    -1583.433811
G(298 K)=    -1583.532813
SCF(D3BJ) =        -1584.06049209
SCF(BS2) =         -3314.07461487
SCF(BS2+D3BJ) =    -3314.24694602
Low Freq. = 12.1883cm-1, 20.2376cm-1
```

```
Cu  0.06637  0.53919  0.08813
N   1.82262 -0.02001  1.24776
N   0.88762 -1.06412 -1.06704
N  -1.21623 -1.40101  1.12224
N  -1.86784  0.35692 -0.91854
C   2.27204  0.51828  2.38886
H   1.68862  1.35801  2.78042
C   3.43000  0.05047  3.05264
H   3.74840  0.53001  3.98192
C   4.14153 -1.01364  2.50911
H   5.04142 -1.39908  2.99917
C   3.69296 -1.60620  1.29895
C   2.51297 -1.06447  0.70275
C   2.01218 -1.62313 -0.53132
C   0.41617 -1.56437 -2.21663
H  -0.48055 -1.08421 -2.61846
C   1.02621 -2.64431 -2.89443
H   0.59089 -3.00789 -3.82875
C   2.17273 -3.22124 -2.35897
H   2.67114 -4.05692 -2.86049
C   2.70326 -2.71320 -1.14413
C   3.88730 -3.23980 -0.51619
H   4.40418 -4.07616 -0.99746
C   4.36373 -2.70754  0.65837
H   5.26619 -3.11274  1.12697
C  -0.90104 -2.25423  2.10141
H   0.08928 -2.12445  2.55391
C  -1.76703 -3.27729  2.55753
H  -1.44792 -3.94563  3.36194
C  -3.01812 -3.40259  1.96616
H  -3.72221 -4.17562  2.29144
C  -3.39297 -2.51051  0.92618
C  -2.43731 -1.51720  0.53176
C  -2.78424 -0.58092 -0.52799
C  -2.20475  1.23164 -1.87809
H  -1.42481  1.95488 -2.14729
C  -3.46190  1.22707 -2.52154
H  -3.67358  1.96498 -3.29973
C  -4.40100  0.27282 -2.15070
H  -5.38493  0.23180 -2.62934
C  -4.08103 -0.66439 -1.13357
C  -5.00948 -1.67797 -0.70717
H  -5.99029 -1.72064 -1.19173
C  -4.67739 -2.57069  0.28172
H  -5.38802 -3.34011  0.60090
C  -0.56169  1.98047  1.31511
```

```
C  -1.76574  1.83631  2.04146
C   0.20146  3.14701  1.55523
C  -2.19101  2.80853  2.96934
C  -0.21396  4.12329  2.48382
C  -1.41421  3.95738  3.19517
H  -2.39366  0.94978  1.89263
H   1.13315  3.30644  0.99858
H  -3.13128  2.66449  3.51596
H   0.40227  5.01642  2.64585
H  -1.74189  4.71563  3.91520
I   1.49317  2.27952 -1.92580
```

[Cu(Phen)I(Ph)]

```
SCF  =      -1012.26111640
H(0 K)=     -1012.005640
H(298 K)=   -1011.985213
G(298 K)=   -1012.058161
SCF(D3BJ) =       -1012.35468342
SCF(BS2) =        -2742.25567243
SCF(BS2+D3BJ) =   -2742.34927408
Low Freq. = 17.7784cm-1,
25.9481cm-1
```

```
Cu  0.51300 -0.08554  0.10269
N  -0.89652  1.40430 -0.42612
N  -1.26160 -1.10067  0.49734
C  -0.67652  2.64623 -0.87794
H   0.37260  2.93034 -1.00934
C  -1.72916  3.54619 -1.16199
H  -1.49417  4.54575 -1.53587
C  -3.04535  3.14362 -0.95725
H  -3.87888  3.82184 -1.16530
C  -3.30918  1.83604 -0.46880
C  -2.18348  0.99634 -0.21938
C  -2.37952 -0.34322  0.27652
C  -1.41782 -2.34757  0.96869
H  -0.49747 -2.92224  1.11299
C  -2.68657 -2.90315  1.24810
H  -2.75141 -3.92282  1.63550
C  -3.83006 -2.14387  1.02088
H  -4.82552 -2.55144  1.22344
C  -3.70089 -0.82149  0.51913
C  -4.81958  0.04660  0.25514
H  -5.82856 -0.33272  0.44405
C  -4.63189  1.32381 -0.21943
H  -5.48926  1.97499 -0.41579
C   2.04090  1.07086  0.47532
C   2.75712  1.74637 -0.53615
C   2.38825  1.31537  1.82279
C   3.78078  2.65911 -0.21147
C   3.41426  2.22568  2.14965
C   4.11081  2.90073  1.13306
H   2.52936  1.56013 -1.59342
H   1.86117  0.80138  2.63682
H   4.32310  3.17545 -1.01238
H   3.66619  2.40388  3.20184
H   4.90947  3.60621  1.38682
I   1.94039 -2.19097 -0.74881
```

[Cu(Phen)$_2$Cl(Ph)]

```
SCF  =      -1587.45388067
H(0 K)=     -1587.031241
H(298 K)=   -1586.999479
G(298 K)=   -1587.097332
SCF(D3BJ) =       -1587.61915188
SCF(BS2) =        -3476.48302784
SCF(BS2+D3BJ) =   -3476.64829905
Low Freq. = 14.3738cm-1,
22.2646cm-1
```

```
C  -1.76815  4.87634  0.94993
C  -2.16806  3.68056  1.57017
C  -1.64469  2.44622  1.13410
C  -0.71282  2.37150  0.07399
C  -0.32720  3.58617 -0.53929
C  -0.84470  4.82490 -0.10817
Cu  0.09690  0.63763 -0.49482
N  -1.78638 -0.19396 -1.09692
C  -2.54669 -0.92523 -0.22560
C  -3.86464 -1.37027 -0.56983
C  -4.37009 -1.02232 -1.85007
C  -3.58198 -0.27556 -2.71756
C  -2.29208  0.11958 -2.30049
C  -4.62673 -2.14940  0.37025
C  -4.11160 -2.47711  1.60017
C  -2.79532 -2.04386  1.98600
C  -2.00299 -1.26382  1.08112
N  -0.75785 -0.81310  1.39910
C  -0.25980 -1.11999  2.60102
C  -0.95571 -1.89120  3.56299
C  -2.22915 -2.35229  3.25176
N   2.00697  0.98370  0.43112
C   2.40479  2.06476  1.11486
C   3.68612  2.16732  1.70457
C   4.58022  1.10961  1.57786
C   4.19064 -0.04962  0.85560
C   2.87704 -0.06017  0.29526
C   2.42599 -1.20840 -0.45539
C   3.29899 -2.32510 -0.63064
C   4.61587 -2.28703 -0.04908
C   5.04482 -1.19329  0.66523
C   2.80597 -3.42423 -1.38245
C   1.51991 -3.36654 -1.90840
C   0.73166 -2.21468 -1.68057
N   1.16455 -1.16316 -0.97410
Cl  0.85377  1.40580 -3.07882
```

```
H   1.67411  2.87657  1.18582
H   3.95520  3.07455  2.25165
H   5.57850  1.15927  2.02445
H  -0.28202 -2.13847 -2.08494
H   1.10851 -4.19304 -2.49352
H   3.44153 -4.30136 -1.54116
H   5.27307 -3.15107 -0.18946
H   6.04853 -1.17348  1.10152
H   0.74364 -0.73576  2.81996
H  -0.49124 -2.10977  4.52842
H  -2.80503 -2.94776  3.96783
H  -1.62059  0.70021 -2.94571
H  -3.93809  0.00664 -3.71188
H  -5.37483 -1.34862 -2.13818
H  -5.63019 -2.47716  0.08007
H  -4.69646 -3.07135  2.30995
H  -1.97457  1.53122  1.64071
H   0.38423  3.56141 -1.37363
H  -2.88896  3.70479  2.39691
H  -0.52537  5.75032 -0.60385
H  -2.17316  5.83740  1.28641
```

[Cu(Phen)Cl(Ph)]

```
SCF  =      -1015.82611973
H(0 K)=     -1015.570313
H(298 K)=   -1015.550152
G(298 K)=   -1015.621264
SCF(D3BJ) =        -1015.91541528
SCF(BS2) =         -2904.66485423
SCF(BS2+D3BJ) =   -2904.75414978
Low Freq. = 19.9509cm-1,
20.9621cm-1
```

```
Cu  0.72319 -0.75354 -0.22723
N  -0.21859  1.11794 -0.36103
N  -1.26831 -1.30922  0.11607
C   0.34080  2.31121 -0.60505
H   1.42213  2.30816 -0.77432
C  -0.41339  3.50642 -0.63711
H   0.09100  4.45236 -0.84900
C  -1.78322  3.45783 -0.39617
H  -2.38865  4.36954 -0.41187
C  -2.40204  2.20789 -0.12652
C  -1.56276  1.05527 -0.12457
C  -2.12633 -0.24590  0.13257
C  -1.76142 -2.53409  0.34984
H  -1.03771 -3.35394  0.30647
C  -3.12991 -2.76238  0.62189
H  -3.47632 -3.78133  0.81148
C  -4.01222 -1.68639  0.64136
H  -5.07674 -1.83601  0.84743
C  -3.52235 -0.37696  0.38893
C  -4.34737  0.80387  0.38257
H  -5.41801  0.69291  0.58041
C  -3.80925  2.04561  0.13514
H  -4.44597  2.93565  0.13216
C   2.52008 -0.09817  0.20047
C   3.41899  0.34657 -0.79193
C   2.91920 -0.02270  1.55304
C   4.68261  0.86519 -0.44311
C   4.18525  0.48938  1.90420
C   5.06802  0.93633  0.90647
H   3.14513  0.28749 -1.85302
H   2.24738 -0.36000  2.35293
H   5.36577  1.20847 -1.22912
H   4.47681  0.53971  2.96009
H   6.05094  1.33596  1.17891
Cl  1.34659 -2.78831 -1.19414
```

Int(3^{+}-8^{+})1

```
SCF  =      -2206.03406295
H(0 K)=     -2205.477313
H(298 K)=   -2205.433269
G(298 K)=   -2205.558938
SCF(D3BJ) =        -2206.23138744
SCF(BS2) =         -3650.10734416
SCF(BS2+D3BJ) =   -3650.30473270
Low Freq. = 15.2476cm-1,
18.1722cm-1
```

```
C  -3.43296 -1.37431  0.12997
Cu  0.36841  0.15007  0.17985
N   0.57850  1.54336 -1.43714
C  -0.17605  2.68434 -1.40279
C  -0.19627  3.60948 -2.49440
C   0.62338  3.32401 -3.61786
C   1.40176  2.17313 -3.62354
C   1.34341  1.30307 -2.51259
C  -4.75422 -0.99995 -0.20855
C  -5.76185 -0.85400  0.76211
C  -5.46793 -1.08366  2.11722
C  -4.16268 -1.45858  2.48472
C  -3.16683 -1.60184  1.50227
C  -1.03773  4.77508 -2.42278
C  -1.82180  5.01464 -1.32038
C  -1.80837  4.11773 -0.19536
C  -0.97479  2.95569 -0.22371
N  -0.87829  2.08891  0.82207
C  -1.61313  2.32568  1.91601
C  -2.49068  3.43172  2.03021
C  -2.58440  4.33178  0.97556
B  -2.31928 -1.54968 -1.04347
N   2.02013 -1.40411 -0.80993
C   3.23101 -1.14676 -0.24325
C   4.38648 -1.95604 -0.48757
C   4.22346 -3.08345 -1.33703
C   2.97545 -3.35305 -1.88623
```

C 1.89984 -2.47880 -1.59673
C 5.64559 -1.60910 0.11564
C 5.75482 -0.50906 0.93072
C 4.61250 0.31749 1.22022
C 3.34105 -0.00167 0.64455
N 2.22563 0.73880 0.92319
C 2.32330 1.79604 1.74021
C 3.54382 2.19445 2.32865
C 4.68839 1.45010 2.07312
O 0.21125 -1.18459 1.64470
C 0.23651 -0.86680 2.91481
O 0.20564 0.28750 3.39326
O -2.78952 -1.34695 -2.39697
C 0.29474 -2.09516 3.83624
H -1.46209 1.61275 2.73434
H -3.07120 3.57092 2.94631
H -3.24121 5.20596 1.03468
H 1.91982 0.37407 -2.48820
H 2.04514 1.92250 -4.47050
H 0.63114 4.01340 -4.46828
H -1.04196 5.46571 -3.27194
H -2.46534 5.89926 -1.27692
H 1.39075 2.32937 1.93698
H 3.56621 3.07020 2.98196
H 5.64912 1.72124 2.52282
H 0.90117 -2.65750 -2.01143
H 2.81658 -4.21610 -2.53834
H 5.08350 -3.72779 -1.54703
H 6.51689 -2.23906 -0.09066
H 6.71502 -0.24414 1.38464
H 0.40663 -1.78263 4.88469
H 1.13331 -2.75227 3.55296
H -0.63031 -2.68812 3.73402
H -4.98549 -0.82553 -1.26669
H -6.24765 -0.97377 2.87973
H -3.92413 -1.64144 3.53964
H -2.15180 -1.88764 1.80129
H -6.77670 -0.56421 0.46339
H -3.10265 -2.24094 -2.68179
O -1.16977 -0.56437 -0.88013
H -0.92383 -0.38489 -1.80953
O -1.67034 -2.97269 -0.87690
C -2.14512 -4.03958 -1.49795
O -3.05018 -4.02919 -2.36028
C -1.49128 -5.33829 -1.04936
H -0.39654 -5.22701 -1.00038
H -1.75862 -6.15745 -1.73127
H -1.83829 -5.59098 -0.03249

Int(3^+-8^+)2
SCF = -1977.40384066
H(0 K)= -1976.894456
H(298 K)= -1976.856851
G(298 K)= -1976.966306
SCF(D3BJ) = -1977.58924857
SCF(BS2) = -3421.37735987
SCF(BS2+D3BJ) = -3421.56282446
Low Freq. = 11.4057cm-1, 18.0825cm-1

C 1.09155 -2.83000 0.98110
Cu -0.15587 0.21363 -0.11788
N -1.63200 1.08540 -1.57216
C -2.88557 0.64515 -1.26085
C -4.04621 1.01968 -2.00374
C -3.85392 1.88977 -3.11050
C -2.57252 2.33187 -3.41947
C -1.48554 1.90375 -2.62108
C 0.44183 -3.91703 1.61068
C 0.33462 -4.00971 3.01032
C 0.88399 -3.00428 3.82563
C 1.55072 -1.91864 3.22860
C 1.65262 -1.84205 1.82728
C -5.33363 0.51121 -1.60957
C -5.46120 -0.32893 -0.52925
C -4.31276 -0.72792 0.24163
C -3.02202 -0.24031 -0.12591
N -1.89431 -0.58861 0.56987
C -1.99515 -1.40662 1.63043
C -3.23601 -1.92698 2.06005
C -4.39284 -1.59096 1.36668
B 1.15288 -2.72834 -0.63270
N 1.54615 1.12056 -0.72742
C 1.90592 2.18357 0.05130
C 3.04570 2.99016 -0.22741
C 3.80699 2.65358 -1.37877
C 3.41753 1.57604 -2.16905
C 2.27684 0.82134 -1.81011
C 3.34827 4.08695 0.65604
C 2.55323 4.36247 1.74486
C 1.38870 3.56685 2.03891
C 1.07099 2.47344 1.18312
N -0.01594 1.66567 1.37597
C -0.82382 1.91525 2.41612
C -0.58588 2.97508 3.32104
C 0.51812 3.80125 3.13641
O 0.59172 -3.86380 -1.30261
H -1.06302 -1.65638 2.14519
H -3.26375 -2.58806 2.92910
H -5.36654 -1.98330 1.67647
H -0.46527 2.23467 -2.84106
H -2.39128 3.00076 -4.26441
H -4.71612 2.20199 -3.70808
H -6.21289 0.80827 -2.18947
H -6.44297 -0.71113 -0.23390
H -1.68586 1.25435 2.53783
H -1.27620 3.12885 4.15367
H 0.72147 4.62810 3.82379

H	1.93837	-0.04363	-2.38525
H	3.97914	1.29932	-3.06427
H	4.69013	3.24615	-1.63675
H	4.22677	4.70266	0.44130
H	2.79251	5.19921	2.40794
H	0.01213	-4.70368	0.97984
H	0.80245	-3.06987	4.91611
H	1.99741	-1.13849	3.85580
H	2.19703	-0.99880	1.38296
H	-0.17665	-4.86511	3.46699
H	1.05896	-3.89988	-2.17493
O	0.35219	-1.45892	-1.14475
H	-0.47272	-1.85509	-1.49015
O	2.60383	-2.40211	-1.02265
C	3.14133	-2.79147	-2.17434
O	2.53458	-3.39922	-3.07854
C	4.61597	-2.44509	-2.28719
H	4.79534	-1.40004	-1.98957
H	4.96995	-2.61440	-3.31312
H	5.19450	-3.08323	-1.59714

Int(3$^+$-8$^+$)3

SCF = -1977.38857914
H(0 K)= -1976.880304
H(298 K)= -1976.842786
G(298 K)= -1976.951934
SCF(D3BJ) = -1977.58008726
SCF(BS2) = -3421.35788088
SCF(BS2+D3BJ) = -3421.54935448
Low Freq. = 14.6894cm-1, 22.2015cm-1

C	3.08812	-3.48972	-1.62627
C	3.72252	-2.36107	-1.04560
C	2.93712	-1.18314	-0.79418
N	1.57849	-1.18720	-1.01194
C	1.02332	-2.26887	-1.58830
C	1.73832	-3.43173	-1.93718
C	3.62648	0.01564	-0.32934
C	5.02205	-0.06560	-0.00335
C	5.74493	-1.29297	-0.18762
C	5.12196	-2.39246	-0.71764
C	5.66748	1.10905	0.46834
C	4.95391	2.29459	0.57307
C	3.59670	2.29918	0.18203
N	2.95693	1.20284	-0.25162
Cu	-0.09557	-0.00685	-0.18277
O	0.47081	1.61965	-1.05723
B	-0.16073	2.67285	-0.15636
O	-1.38419	3.14601	-0.94449
C	-0.67587	1.87018	1.20427
C	0.28943	1.49640	2.18918
C	-0.08639	0.96070	3.43235
C	-1.44765	0.77417	3.72905
C	-2.42702	1.12056	2.77665
C	-2.04391	1.65011	1.53690
N	-0.80303	-1.53235	0.90370
C	-2.02550	-1.99056	0.48742
C	-2.70869	-3.05154	1.14966
C	-2.06749	-3.63628	2.27425
C	-0.82371	-3.16332	2.67864
C	-0.21812	-2.10336	1.96764
C	-3.99343	-3.47084	0.65117
C	-4.56293	-2.86808	-0.44653
C	-3.89101	-1.79839	-1.13779
C	-2.61757	-1.36469	-0.66730
N	-1.90654	-0.36442	-1.26502
C	-2.42385	0.24914	-2.33622
C	-3.68080	-0.11265	-2.87650
C	-4.41357	-1.13459	-2.28068
O	0.80874	3.70650	0.08549
H	-1.82521	1.05859	-2.76264
H	-4.05938	0.41593	-3.75470
H	-5.38740	-1.43277	-2.68146
H	0.75761	-1.70842	2.26070
H	-0.30376	-3.59397	3.53736
H	-2.55693	-4.45455	2.81137
H	-4.51055	-4.28503	1.16769
H	-5.53941	-3.19680	-0.81495
H	-0.05447	-2.21833	-1.76979
H	1.21664	-4.26715	-2.40979
H	3.67805	-4.38894	-1.83010
H	2.97923	3.20563	0.21488
H	5.42348	3.21510	0.92879
H	6.72893	1.06282	0.73263
H	6.80742	-1.31974	0.07362
H	5.67567	-3.31836	-0.90134
H	1.35015	1.68097	1.98442
H	-1.74746	0.36542	4.69972
H	-3.48823	0.98381	3.01169
H	-2.81281	1.92435	0.80624
H	0.67736	0.70323	4.17416
H	1.47544	1.52740	-0.81617
H	0.27057	4.51693	0.25889
C	-1.88581	4.36892	-0.78134
O	-1.40204	5.24067	-0.03508
C	-3.13434	4.61186	-1.61004
H	-2.91863	4.43717	-2.67699
H	-3.92599	3.90189	-1.31860
H	-3.49093	5.64065	-1.46745

TS(3$^+$-8$^+$)

SCF = -1977.37813547
H(0 K)= -1976.871713
H(298 K)= -1976.834392
G(298 K)= -1976.943192
SCF(D3BJ) = -1977.56786801
SCF(BS2) = -3421.34739147

SCF(BS2+D3BJ) = -3421.53710563
Low Freq. = -235.6032cm-1,
11.1071cm-1

C 2.97304 -3.72135 -1.20226
C 3.63779 -2.54320 -0.77298
C 2.88826 -1.31831 -0.70415
N 1.53503 -1.30146 -0.95203
C 0.95406 -2.43569 -1.38052
C 1.63094 -3.66175 -1.54349
C 3.61086 -0.09142 -0.38462
C 4.99686 -0.17090 -0.01963
C 5.68157 -1.43347 -0.02556
C 5.02991 -2.57416 -0.41532
C 5.67246 1.03382 0.31334
C 4.99718 2.24478 0.25542
C 3.64849 2.24107 -0.16098
N 2.98224 1.11859 -0.47072
Cu -0.11392 0.07455 -0.23211
O 0.56720 1.52459 -1.36926
B -0.03848 2.61952 -0.62268
O -1.34954 2.94744 -1.24049
C -0.56404 1.74354 1.09812
C 0.41598 1.73743 2.12493
C 0.04792 1.75250 3.48071
C -1.31338 1.77081 3.83694
C -2.30236 1.77760 2.83621
C -1.92844 1.74983 1.48260
N -0.85789 -1.32119 1.03498
C -2.04791 -1.87230 0.64085
C -2.74826 -2.82273 1.44197
C -2.15579 -3.19896 2.67670
C -0.94100 -2.63731 3.05467
C -0.32122 -1.69295 2.20668
C -4.00317 -3.34822 0.96959
C -4.53207 -2.95074 -0.23598
C -3.84329 -2.00079 -1.07020
C -2.59615 -1.46392 -0.63176
N -1.87245 -0.56992 -1.36715
C -2.35832 -0.16748 -2.54816
C -3.58755 -0.63882 -3.06635
C -4.32928 -1.55664 -2.32949
O 0.82959 3.68004 -0.31956
H -1.74965 0.55706 -3.09743
H -3.93643 -0.27912 -4.03752
H -5.28156 -1.94148 -2.70778
H 0.62303 -1.21626 2.48258
H -0.45889 -2.90674 3.99725
H -2.66133 -3.92649 3.31927
H -4.53078 -4.07459 1.59526
H -5.48784 -3.35541 -0.58298
H -0.11684 -2.37281 -1.59343
H 1.08664 -4.53825 -1.90306
H 3.53387 -4.65924 -1.26635
H 3.06450 3.16474 -0.24821
H 5.48989 3.18785 0.50430
H 6.72713 0.98898 0.60369
H 6.73799 -1.45786 0.25926
H 5.55477 -3.53351 -0.45992
H 1.47927 1.75317 1.85931
H -1.60282 1.78405 4.89313
H -3.36240 1.80281 3.11250
H -2.70620 1.74876 0.71021
H 0.81898 1.75958 4.25920
H 1.56991 1.39371 -1.07335
H 0.23884 4.43910 -0.08327
C -1.95195 4.12596 -1.01445
O -1.47638 5.02859 -0.30744
C -3.28689 4.24314 -1.71752
H -3.15623 4.08756 -2.80098
H -3.97294 3.45873 -1.35755
H -3.72557 5.23234 -1.53286

Int(3^+-8^+)4
SCF = -1977.39805956
H(0 K)= -1976.892014
H(298 K)= -1976.853603
G(298 K)= -1976.967830
SCF(D3BJ) = -1977.57825854
SCF(BS2) = -3421.36942422
SCF(BS2+D3BJ) = -3421.54959688
Low Freq. = 11.3941cm-1,
13.4271cm-1

C 2.20578 -4.16603 -0.89902
C 3.07607 -3.04509 -0.85229
C 2.49338 -1.73036 -0.85849
N 1.13555 -1.54360 -0.87629
C 0.35596 -2.63354 -0.94106
C 0.83608 -3.96253 -0.95991
C 3.39328 -0.58083 -0.86863
C 4.81161 -0.80297 -0.82968
C 5.34069 -2.13811 -0.78808
C 4.50154 -3.22108 -0.80677
C 5.67461 0.32464 -0.84674
C 5.14064 1.60332 -0.91189
C 3.73856 1.74026 -0.95901
N 2.89800 0.69379 -0.93731
Cu -0.48513 0.08825 0.29661
O 0.50239 1.54009 -0.86845
B 0.30687 2.88923 -0.80286
O -1.03564 3.28215 -0.47252
C 0.58646 0.43393 1.91198
C 1.38973 -0.56210 2.50829
C 2.10549 -0.30353 3.69533
C 2.02596 0.95890 4.30683
C 1.22907 1.96014 3.72648
C 0.51714 1.69736 2.53884

N -2.01523 -0.97914 1.28618
C -3.10434 -1.21039 0.49366
C -4.27957 -1.86570 0.96550
C -4.28513 -2.28575 2.32256
C -3.16857 -2.04661 3.11701
C -2.04832 -1.38559 2.56284
C -5.37892 -2.06594 0.05661
C -5.30778 -1.64053 -1.24976
C -4.13131 -0.98016 -1.75372
C -3.03030 -0.76376 -0.87445
N -1.87532 -0.14608 -1.26270
C -1.76918 0.27310 -2.53164
C -2.80595 0.10088 -3.47751
C -3.98785 -0.52333 -3.09124
O 1.28697 3.80681 -1.04807
H -0.82825 0.76548 -2.79124
H -2.66472 0.46161 -4.49923
H -4.80603 -0.66739 -3.80397
H -1.15256 -1.17555 3.15487
H -3.14119 -2.35849 4.16391
H -5.16557 -2.79295 2.72927
H -6.27749 -2.56814 0.42805
H -6.14928 -1.79943 -1.93109
H -0.72256 -2.44875 -0.97618
H 0.13200 -4.79693 -1.01331
H 2.63142 -5.17471 -0.89507
H 3.26109 2.72505 -1.01713
H 5.77662 2.49146 -0.93063
H 6.75736 0.16605 -0.81295
H 6.42686 -2.26784 -0.75401
H 4.89999 -4.24058 -0.79116
H 1.46880 -1.55655 2.05178
H 2.58109 1.16114 5.22926
H 1.15856 2.94857 4.19584
H -0.09982 2.49668 2.10899
H 2.72520 -1.09185 4.13902
H 1.52486 1.13460 -0.93390
H 0.86876 4.70711 -0.95192
C -1.41202 4.59070 -0.39656
O -0.63776 5.53137 -0.60067
C -2.86533 4.74796 -0.03995
H -3.49024 4.25181 -0.80081
H -3.06922 4.25055 0.92248
H -3.12487 5.81213 0.02314

Int3(3^+-8^+)5
SCF = -1405.74756311
H(0 K)= -1405.406812
H(298 K)= -1405.380331
G(298 K)= -1405.464721
SCF(D3BJ) = -1405.86194329
SCF(BS2) = -2849.52455538
SCF(BS2+D3BJ) = -2849.63893554
Low Freq. = 13.7433cm-1,
22.8240cm-1

Cu -0.16343 -0.15314 0.31926
O -1.59116 -1.37728 0.75730
B -2.66251 -0.30928 1.04417
O -2.89673 -0.11676 2.43723
C -2.12914 1.04157 0.21704
C -1.84896 1.00185 -1.18847
C -1.65957 2.17917 -1.93578
C -1.76033 3.42722 -1.30214
C -2.03229 3.50041 0.08247
C -2.18978 2.32731 0.82937
N 1.37542 1.11522 0.24447
C 2.56498 0.46300 0.04562
C 3.80846 1.14504 -0.06805
C 3.78143 2.56138 0.03884
C 2.56885 3.20898 0.25088
C 1.37915 2.45327 0.34881
C 5.00650 0.37153 -0.27354
C 4.96561 -1.00198 -0.34878
C 3.72092 -1.71548 -0.22326
C 2.52451 -0.96972 -0.03074
N 1.30145 -1.55919 0.10592
C 1.20449 -2.89414 0.07134
C 2.33986 -3.71516 -0.12039
C 3.59392 -3.13033 -0.27066
O -3.99297 -0.76209 0.46406
H 0.20530 -3.31682 0.20569
H 2.21425 -4.79987 -0.14548
H 4.48400 -3.74904 -0.41960
H 0.41069 2.93217 0.51169
H 2.51463 4.29585 0.34355
H 4.71316 3.12941 -0.04214
H 5.95816 0.90314 -0.36448
H 5.88418 -1.57638 -0.50053
H -1.85032 0.03838 -1.71549
H -1.63160 4.34707 -1.88115
H -2.12525 4.47756 0.56749
H -2.42214 2.38839 1.89800
H -1.45783 2.12037 -3.00973
H -2.11334 0.25361 2.87980
H -1.96996 -1.86612 -0.06405
C -4.11170 -1.62179 -0.53587
O -3.14810 -2.23883 -1.05308
C -5.52914 -1.80294 -1.02356
H -5.88089 -0.86471 -1.48469
H -5.57792 -2.61531 -1.76010
H -6.19603 -2.01791 -0.17362

TS2(3^+-8^+)
SCF = -1405.74099881
H(0 K)= -1405.401356
H(298 K)= -1405.375267

G(298 K)= -1405.457984
SCF(D3BJ) = -1405.85532192
SCF(BS2) = -2849.51772009
SCF(BS2+D3BJ) = -2849.63207508
Low Freq. = -207.3201cm-1, 17.7655cm-1

Cu	-0.13432	-0.29929	0.23978
O	-1.41149	-1.81506	0.44426
B	-2.45301	-0.93655	0.99422
O	-2.57697	-0.87289	2.38412
C	-1.87847	0.86134	0.32921
C	-2.15276	1.22175	-1.01979
C	-2.56875	2.52156	-1.35289
C	-2.71159	3.49082	-0.34336
C	-2.43475	3.16080	0.99718
C	-2.00945	1.86355	1.32585
N	1.25771	1.16588	0.03305
C	2.52138	0.65325	-0.09144
C	3.67316	1.47147	-0.26486
C	3.46589	2.87615	-0.31372
C	2.17584	3.38230	-0.19400
C	1.08914	2.49519	-0.02043
C	4.96258	0.83913	-0.38041
C	5.09357	-0.52975	-0.32645
C	3.94331	-1.37942	-0.15557
C	2.65937	-0.77653	-0.04145
N	1.51318	-1.49950	0.11870
C	1.58459	-2.83593	0.17977
C	2.81665	-3.52221	0.07730
C	3.99362	-2.79856	-0.09143
O	-3.78208	-1.08724	0.36785
H	0.63677	-3.36416	0.31640
H	2.82645	-4.61319	0.13171
H	4.95722	-3.31058	-0.17432
H	0.06491	2.86189	0.08567
H	1.98341	4.45695	-0.22944
H	4.32102	3.54615	-0.44573
H	5.84303	1.47477	-0.51299
H	6.07933	-0.99587	-0.41453
H	-2.06381	0.47569	-1.81916
H	-3.04006	4.50313	-0.60023
H	-2.55608	3.91299	1.78379
H	-1.82604	1.60244	2.37341
H	-2.78618	2.77698	-2.39522
H	-1.73549	-1.08431	2.82613
H	-1.80333	-2.09953	-0.46333
C	-3.95185	-1.67205	-0.82179
O	-3.02308	-2.20187	-1.46992
C	-5.37242	-1.62712	-1.31864
H	-5.65277	-0.57998	-1.52298
H	-5.47347	-2.22260	-2.23477
H	-6.05629	-2.00129	-0.54040

Int(3^+-8^+)6
SCF = -1405.75725704
H(0 K)= -1405.416996
H(298 K)= -1405.389799
G(298 K)= -1405.477990
SCF(D3BJ) = -1405.86479426
SCF(BS2) = -2849.53738437
SCF(BS2+D3BJ) = -2849.64493514
Low Freq. = 13.2903cm-1, 21.4119cm-1

Cu	-0.13064	0.37754	0.14576
O	-1.88126	-0.79662	-0.25981
B	-3.19553	-0.61873	0.14742
O	-3.64748	0.44388	0.85050
C	-1.06067	2.08832	0.28638
C	-1.54497	2.76182	-0.85788
C	-2.18663	4.00960	-0.73808
C	-2.35644	4.59807	0.52723
C	-1.88294	3.93924	1.67273
C	-1.23067	2.69286	1.55426
N	1.72427	1.07262	-0.42310
C	2.69718	0.12806	-0.25131
C	4.06741	0.37415	-0.55465
C	4.39633	1.66015	-1.06171
C	3.39260	2.60802	-1.23412
C	2.05952	2.28012	-0.89757
C	5.02774	-0.67647	-0.33408
C	4.64138	-1.89881	0.16513
C	3.26282	-2.17170	0.48058
C	2.29075	-1.15370	0.26311
N	0.96003	-1.32570	0.52907
C	0.55191	-2.50206	1.02936
C	1.44440	-3.56751	1.28469
C	2.79820	-3.40666	1.00764
O	-4.16695	-1.61008	-0.16844
H	-0.51850	-2.59930	1.22907
H	1.05679	-4.50332	1.69374
H	3.50881	-4.21816	1.19224
H	1.24333	2.99966	-1.00857
H	3.61505	3.60483	-1.62198
H	5.43599	1.89406	-1.31054
H	6.07797	-0.48096	-0.57059
H	5.38026	-2.68801	0.33334
H	-1.42938	2.31712	-1.85395
H	-2.85699	5.56763	0.61917
H	-2.01157	4.39060	2.66284
H	-0.85833	2.20190	2.46198
H	-2.55670	4.51813	-1.63538
H	-2.93631	1.11716	0.96687
H	-1.85667	-1.67154	-0.77758
C	-3.85198	-2.73008	-0.87479
O	-2.70835	-2.96123	-1.29368
C	-5.03042	-3.63377	-1.08705

H -5.82060 -3.08749 -1.62817
H -4.72753 -4.52201 -1.65481
H -5.45041 -3.92918 -0.11155

Int(3⁺-8⁺)9
SCF =   -1977.40326262
H(0 K)=   -1976.894058
H(298 K)=   -1976.856289
G(298 K)=   -1976.967508
SCF(D3BJ) =   -1977.58595002
SCF(BS2) =   -3421.37697729
SCF(BS2+D3BJ) =   -3421.55966473
Low Freq. = 10.1712cm-1,
16.2205cm-1

C   2.11357 3.69575 -2.15851
C   1.29354 3.88335 -1.01341
C   0.65253 2.72810 -0.48321
N   0.80315 1.48552 -1.02869
C   1.58934 1.32686 -2.10276
C   2.26080 2.42063 -2.69691
C -0.18603 2.83131 0.67763
C -0.38035 4.09540 1.30426
C   0.27269 5.25265 0.74724
C   1.07638 5.15137 -0.36527
C -1.21583 4.12452 2.45258
C -1.79582 2.94398 2.90597
C -1.54844 1.73473 2.21665
N -0.76287 1.67701 1.13196
Cu -0.31856 0.07180 -0.11291
C   2.49565 -1.42434 1.01671
C   2.34494 -2.13355 2.23281
C   2.74201 -1.59173 3.46922
C   3.31460 -0.30907 3.52050
C   3.49611 0.41327 2.32732
C   3.09468 -0.14286 1.09930
N -2.10886 0.04661 -1.47292
C -3.05317 -0.79192 -0.95580
C -4.31919 -1.01311 -1.57784
C -4.57821 -0.31191 -2.78617
C -3.60992 0.54088 -3.30389
C -2.38425 0.69157 -2.61305
C -2.73448 -1.48282 0.27340
C -3.68378 -2.38047 0.84991
C -4.95149 -2.57750 0.19685
C -5.25778 -1.91952 -0.97031
C -3.31833 -3.03930 2.05351
C -2.07048 -2.80037 2.61801
C -1.18689 -1.89953 1.98437
N -1.51334 -1.25754 0.85141
O   0.79789 -1.37310 -0.96795
B   2.01923 -2.10943 -0.38201
O   1.63728 -3.49651 -0.32291
O   3.11670 -1.83383 -1.45060
C   4.07896 -2.70984 -1.70744
C   5.12175 -2.18321 -2.67973
O   4.15538 -3.85331 -1.21338
H   1.69453 0.30095 -2.46631
H   2.89146 2.24592 -3.57169
H   2.62599 4.55395 -2.60407
H -1.99375 0.79431 2.55120
H -2.44126 2.93139 3.78725
H -1.39482 5.07197 2.97006
H   0.11704 6.22111 1.23190
H   1.56756 6.03813 -0.77682
H -1.60281 1.35474 -2.99807
H -3.77810 1.09324 -4.23168
H -5.53602 -0.45155 -3.29719
H -0.18896 -1.69941 2.38750
H -1.75770 -3.29574 3.53995
H -4.02300 -3.73243 2.52340
H -5.66837 -3.26735 0.65216
H -6.22362 -2.07734 -1.45996
H   1.91919 -3.14371 2.19117
H   3.62978 0.11866 4.47871
H   3.96099 1.40586 2.35458
H   3.27066 0.42683 0.17919
H   2.61456 -2.17194 4.39070
H   0.22717 -2.09274 -1.30626
H   2.47274 -3.99136 -0.51229
H   4.63509 -1.79649 -3.58978
H   5.66739 -1.34171 -2.22030
H   5.83302 -2.97736 -2.94410

TS3(3⁺-8⁺)
SCF =   -1977.36826582
H(0 K)=   -1976.861053
H(298 K)=   -1976.823225
G(298 K)=   -1976.933152
SCF(D3BJ) =   -1977.56046634
SCF(BS2) =   -3421.33882812
SCF(BS2+D3BJ) =   -3421.53102865
Low Freq. = -271.3757cm-1,
14.3832cm-1

C   3.51243 3.20004 -1.43962
C   2.73747 3.23080 -0.24952
C   1.76910 2.19213 -0.06739
N   1.56887 1.20522 -0.98582
C   2.32558 1.20084 -2.09139
C   3.31129 2.17977 -2.36132
C   0.97162 2.17214 1.14625
C   1.16149 3.19931 2.12532
C   2.13430 4.23462 1.89604
C   2.89628 4.24882 0.75390
C   0.37181 3.14811 3.30384
C -0.54361 2.11695 3.47037
C -0.66429 1.14123 2.45760

```
N   0.06503  1.16431  1.33256
Cu -0.15011 -0.29670 -0.13746
C   1.20626 -1.63587  0.77560
C   0.66362 -2.55808  1.70530
C   1.35567 -2.93430  2.86889
C   2.62491 -2.39129  3.13490
C   3.19813 -1.48943  2.22161
C   2.49207 -1.11373  1.06567
N  -1.69253  0.77090 -1.18918
C  -3.02001  0.49399 -0.99445
C  -4.04381  1.13906 -1.76302
C  -3.64796  2.08398 -2.74487
C  -2.29871  2.34970 -2.92877
C  -1.35662  1.66836 -2.12906
C  -3.40251 -0.47563  0.01755
C  -4.79413 -0.75258  0.22423
C  -5.79019 -0.08872 -0.57173
C  -5.42742  0.82333 -1.53018
C  -5.13789 -1.68898  1.23461
C  -4.13080 -2.28430  1.98118
C  -2.78723 -1.94588  1.69834
N  -2.41759 -1.08101  0.74365
O  -0.14633 -1.67966 -1.70302
B   0.94475 -2.48539 -1.20646
O   0.66916 -3.82143 -0.99221
O   2.19950 -2.06181 -1.81230
C   3.29612 -2.85229 -1.76986
C   4.49160 -2.21557 -2.43531
O   3.31514 -3.97355 -1.24616
H   2.14572  0.37846 -2.79110
H   3.89406  2.12446 -3.28448
H   4.26135  3.97925 -1.61429
H  -1.37315  0.31472  2.55000
H  -1.16712  2.04436  4.36473
H   0.49618  3.92104  4.06894
H   2.25713  5.00974  2.65888
H   3.63972  5.03501  0.58854
H  -0.28738  1.85025 -2.25157
H  -1.95162  3.06851 -3.67495
H  -4.41184  2.58963 -3.34411
H  -1.97798 -2.39331  2.28373
H  -4.35447 -3.00138  2.77517
H  -6.19197 -1.92303  1.41563
H  -6.84411 -0.32490 -0.39419
H  -6.18481  1.33082 -2.13568
H  -0.28931 -3.04656  1.48158
H   3.16965 -2.68050  4.04006
H   4.19615 -1.07708  2.40907
H   2.96036 -0.41307  0.36741
H   0.91143 -3.66115  3.55836
H  -0.97629 -2.19433 -1.63832
H   1.55225 -4.26530 -0.90802
H   4.25719 -1.99157 -3.48908
H   4.72776 -1.25757 -1.94403
H   5.35664 -2.88876 -2.38077
```

Int(3^{+}-8^{+})7_{H2O}

```
SCF  =       -2282.48206588
H(0 K)=      -2281.900176
H(298 K)=    -2281.853955
G(298 K)=    -2281.983689
SCF(D3BJ) =        -2282.68738574
SCF(BS2) =         -3726.59085785
SCF(BS2+D3BJ) =    -3726.79617771
Low Freq. = 16.9884cm-1,
21.8591cm-1

C   3.22561 -1.48027 -0.31463
Cu -0.41982  0.25116 -0.15246
N  -0.36144  1.62122  1.43796
C   0.54614  2.64123  1.36089
C   0.74918  3.55791  2.43988
C  -0.04503  3.39127  3.60468
C  -0.97701  2.36178  3.65545
C  -1.10056  1.49211  2.54897
C   4.52113 -1.02244  0.02162
C   5.49085 -0.74013 -0.95751
C   5.18556 -0.91528 -2.31833
C   3.90672 -1.37298 -2.68385
C   2.94818 -1.64929 -1.69286
C   1.73481  4.59890  2.30899
C   2.47767  4.73003  1.16050
C   2.28335  3.83739  0.04859
C   1.31231  2.79234  0.14192
N   1.05087  1.92932 -0.87950
C   1.74069  2.06383 -2.01953
C   2.73679  3.05388 -2.20258
C   3.00793  3.94235 -1.16896
B   2.13975 -1.80901  0.84969
N  -2.28630 -1.04596  0.95924
C  -3.42513 -0.75695  0.27019
C  -4.63603 -1.50445  0.43136
C  -4.60614 -2.61086  1.32242
C  -3.43410 -2.90583  2.00748
C  -2.29756 -2.08546  1.80399
C  -5.82333 -1.11493 -0.28156
C  -5.81739 -0.02208 -1.11299
C  -4.61819  0.74702 -1.31656
C  -3.40883  0.37083 -0.64782
N  -2.24063  1.04903 -0.85614
C  -2.23030  2.10919 -1.67444
C  -3.38636  2.57175 -2.34078
C  -4.57870  1.87987 -2.17170
O  -0.52409 -1.11921 -1.55914
C  -0.54916 -0.85120 -2.84113
O  -0.38570  0.26892 -3.36957
O   2.72963 -1.92115  2.20330
C  -0.78687 -2.09848 -3.70583
```

H   1.45768 1.36554 -2.81428
H   3.27216 3.11269 -3.15404
H   3.76549 4.72508 -1.28002
H -1.80836 0.65918 2.55139
H -1.60868 2.20654 4.53358
H   0.08435 4.07666 4.44854
H   1.87961 5.28579 3.14873
H   3.22666 5.52336 1.06977
H -1.26118 2.59515 -1.81164
H -3.32130 3.44869 -2.98964
H -5.49080 2.19335 -2.68985
H -1.36958 -2.25411 2.36512
H -3.38110 -3.74553 2.70595
H -5.51079 -3.21139 1.46446
H -6.73630 -1.70196 -0.13792
H -6.72557 0.28239 -1.64304
H -0.97014 -1.80797 -4.75059
H -1.63930 -2.68345 -3.32479
H   0.10081 -2.75334 -3.66889
H   4.76986 -0.89840 1.08273
H   5.93738 -0.70141 -3.08665
H   3.65982 -1.51690 -3.74271
H   1.95453 -2.00395 -1.98863
H   6.48669 -0.38933 -0.66060
H   3.09826 -2.84435 2.21405
O   1.05816 -0.78527 0.93332
H   0.63859 -0.90585 1.82835
O   1.43491 -3.17090 0.48913
C   1.96959 -4.33591 0.80959
O   2.98652 -4.48962 1.52277
C   1.23354 -5.52495 0.21176
H   0.15007 -5.43771 0.39153
H   1.61150 -6.46440 0.63808
H   1.38161 -5.53857 -0.88160
O   0.51293 -1.54466 3.55616
H   1.44297 -1.78238 3.22326
H   0.64845 -0.75357 4.10993

Int(3^+-8^+)9_{H2O}

SCF =   -2053.85072609
H(0 K)=   -2053.316588
H(298 K)=   -2053.276698
G(298 K)=   -2053.392514
SCF(D3BJ) =   -2054.04036734
SCF(BS2) =   -3497.85993957
SCF(BS2+D3BJ) =   -3498.04958081
Low Freq. = 7.9496cm-1, 14.4490cm-1

C -4.59547 -0.70329 -2.59916
C -4.25030 -1.31597 -1.36446
C -2.98443 -0.97350 -0.79946
N -2.11776 -0.10371 -1.39454
C -2.47222 0.45412 -2.55837
C -3.70553 0.18209 -3.19664
C -2.58039 -1.56992 0.45422
C -3.44531 -2.49832 1.10978
C -4.71491 -2.81940 0.51184
C -5.10308 -2.24996 -0.67736
C -2.99770 -3.06178 2.33404
C -1.75422 -2.70424 2.84268
C -0.95725 -1.77971 2.13254
N -1.36215 -1.22733 0.97824
Cu -0.30411 0.13762 -0.08871
O   0.95132 -1.21121 -0.87214
B   2.17467 -1.81965 -0.22515
O   3.27846 -1.62171 -1.31106
C   2.62267 -1.00221 1.10837
C   2.58451 -1.62445 2.37939
C   2.96784 -0.95000 3.55354
C   3.41088 0.38180 3.48412
C   3.47841 1.02184 2.23338
C   3.09361 0.33377 1.06892
N -0.91169 1.76784 1.06517
C -0.45840 2.94682 0.53964
C -0.77847 4.21924 1.09395
C -1.61094 4.23057 2.24474
C -2.06658 3.02557 2.77001
C -1.69679 1.80955 2.15092
C -0.24678 5.40238 0.46654
C   0.55695 5.31925 -0.64745
C   0.89570 4.04460 -1.22671
C   0.37922 2.86283 -0.62351
N   0.65210 1.61308 -1.09923
C   1.44061 1.47298 -2.17364
C   1.99102 2.59251 -2.83980
C   1.72010 3.87511 -2.37152
O   1.91205 -3.24891 -0.00950
H   1.64496 0.44233 -2.47722
H   2.62754 2.43243 -3.71317
H   2.13848 4.75352 -2.87248
H -2.04269 0.85025 2.54426
H -2.70647 2.99884 3.65504
H -1.88612 5.18343 2.70723
H -0.49495 6.37648 0.89846
H   0.95468 6.22613 -1.11269
H -1.75019 1.14463 -3.00630
H -3.94028 0.66815 -4.14681
H -5.55711 -0.93611 -3.06719
H   0.03342 -1.48389 2.49247
H -1.37929 -3.12321 3.77929
H -3.63674 -3.77493 2.86404
H -5.36599 -3.53171 1.02749
H -6.06965 -2.50144 -1.12466
H   2.25765 -2.66998 2.43567
H   3.71348 0.91269 4.39349
H   3.84217 2.05385 2.16608
H   3.18003 0.84301 0.10196

H   2.92884 -1.46486 4.52061
H   0.44632 -1.95289 -1.33637
H   2.80665 -3.67265 -0.10508
C   4.31048 -2.44615 -1.41881
O   4.45252 -3.50227 -0.76626
C   5.34647 -1.98299 -2.42863
H   4.86385 -1.74870 -3.39119
H   5.82493 -1.05575 -2.07039
H   6.11342 -2.75619 -2.57153
O   0.06464 -3.52722 -1.85086
H   0.78428 -3.70142 -1.16067
H -0.75894 -3.82220 -1.41946

TS3(3^{+}-8^{+})$_{H2O}$
SCF =   -2053.81740347
H(0 K)=   -2053.285686
H(298 K)=   -2053.245656
G(298 K)=   -2053.360031
SCF(D3BJ) =   -2054.01536477
SCF(BS2) =   -3497.82375554
SCF(BS2+D3BJ) =   -3498.02171684
Low Freq. = -220.4830cm-1,
17.3448cm-1

C -3.63912 1.94295 -2.87663
C -4.01415 1.15041 -1.76070
C -2.97190 0.56340 -0.97114
N -1.65147 0.75660 -1.26852
C -1.33277 1.50500 -2.33475
C -2.29346 2.11854 -3.16742
C -3.32799 -0.26220 0.17034
C -4.71416 -0.45686 0.48044
C -5.73033 0.15257 -0.33387
C -5.39210 0.92672 -1.41508
C -5.03137 -1.26267 1.60561
C -4.00439 -1.81992 2.35492
C -2.66803 -1.56906 1.96603
N -2.32473 -0.82221 0.90723
Cu -0.07781 -0.18715 -0.10436
O   0.04785 -1.67014 -1.40528
B   1.03826 -2.49713 -0.71975
O   2.33859 -2.39743 -1.43477
C   1.40475 -1.55382 1.00668
C   0.79874 -2.15525 2.13778
C   1.34381 -2.02140 3.42544
C   2.52828 -1.28481 3.60860
C   3.17065 -0.70728 2.49809
C   2.61035 -0.83755 1.21666
N   0.15740 1.38788 1.17076
C   1.05722 2.36377 0.82834
C   1.26266 3.51709 1.64857
C   0.50106 3.62714 2.84140
C -0.40004 2.62342 3.17261
C -0.54231 1.51693 2.30815
C   2.22605 4.51008 1.25091
C   2.96514 4.36173 0.10257
C   2.79294 3.20832 -0.73948
C   1.83101 2.21060 -0.38699
N   1.61493 1.09671 -1.14056
C   2.34934 0.91898 -2.24569
C   3.32869 1.84557 -2.67734
C   3.54523 2.99491 -1.92591
O   0.56548 -3.80139 -0.41737
H   2.15585 -0.00137 -2.80609
H   3.89642 1.64912 -3.59053
H   4.29067 3.73605 -2.23180
H -1.24409 0.71101 2.53211
H -0.99952 2.67098 4.08471
H   0.63604 4.49958 3.48829
H   2.36171 5.38641 1.89238
H   3.70089 5.11798 -0.18839
H -0.26542 1.62010 -2.53577
H -1.96415 2.71682 -4.02041
H -4.41646 2.40285 -3.49517
H -1.84088 -1.98627 2.54831
H -4.20736 -2.44102 3.23111
H -6.08065 -1.43074 1.86874
H -6.77989 -0.01594 -0.07268
H -6.16517 1.38902 -2.03673
H -0.07622 -2.79681 1.98996
H   2.95934 -1.17753 4.60981
H   4.11070 -0.16023 2.63225
H   3.12801 -0.39222 0.36126
H   0.86069 -2.50686 4.28094
H -0.81473 -2.20696 -1.49811
H   1.37655 -4.37034 -0.34078
C   3.27799 -3.34745 -1.31939
O   3.12505 -4.40738 -0.68859
C   4.56015 -2.99507 -2.04004
H   4.34839 -2.75625 -3.09487
H   5.00578 -2.09463 -1.58542
H   5.27039 -3.82988 -1.97942
O -1.84070 -3.48666 -1.52504
H -1.09068 -3.97454 -1.08607
H -2.50593 -3.38300 -0.81864

Int(3^{+}-8^{+})7$_{MeOH}$
SCF =   -2321.77693184
H(0 K)=   -2321.167683
H(298 K)=   -2321.119780
G(298 K)=   -2321.254367
SCF(D3BJ) =   -2321.98975683
SCF(BS2) =   -3765.89556025
SCF(BS2+D3BJ) =   -3766.10838525
Low Freq. = 12.1472cm-1,
16.9653cm-1

C   3.27135 -0.97242 -0.67149

```
Cu -0.41309  0.13736 -0.23954
N  -0.78826  1.47099  1.38604
C  -0.18108  2.69667  1.38357
C  -0.31173  3.60782  2.47950
C  -1.12555  3.21340  3.57368
C  -1.75087  1.97351  3.54827
C  -1.54595  1.12683  2.43664
C   4.14367  0.14059 -0.74019
C   4.90146  0.42735 -1.88952
C   4.81530 -0.41187 -3.01430
C   3.96596 -1.53144 -2.97273
C   3.20768 -1.79799 -1.81822
C   0.37508  4.87208  2.43944
C   1.15278  5.22113  1.36207
C   1.28726  4.33984  0.23283
C   0.61137  3.07892  0.23149
N   0.65781  2.21575 -0.82053
C   1.38602  2.55513 -1.89140
C   2.11259  3.76824 -1.97640
C   2.05938  4.66375 -0.91522
B   2.45349 -1.26810  0.70267
N  -2.00272 -1.54277  0.66982
C  -3.19541 -1.38007  0.03405
C  -4.28848 -2.29078  0.19713
C  -4.07905 -3.41695  1.03785
C  -2.84741 -3.58906  1.65794
C  -1.83867 -2.61835  1.44728
C  -5.53580 -2.04281 -0.47500
C  -5.69253 -0.94149 -1.28024
C  -4.61100 -0.01533 -1.49085
C  -3.35169 -0.23358 -0.84499
N  -2.28956  0.60352 -1.04931
C  -2.43300  1.66088 -1.85941
C  -3.64762  1.96348 -2.51362
C  -4.73622  1.12004 -2.33409
O  -0.15992 -1.19113 -1.69102
C  -0.10156 -0.85840 -2.95379
O  -0.08472  0.30306 -3.41820
O   3.23146 -0.92383  1.91627
C  -0.01127 -2.06645 -3.89810
H   1.35302  1.83374 -2.71535
H   2.69364  3.99039 -2.87565
H   2.59692  5.61712 -0.95181
H  -1.99909  0.13328  2.38953
H  -2.38455  1.63630  4.37226
H  -1.24897  3.89051  4.42511
H   0.26515  5.55001  3.29179
H   1.67677  6.18225  1.34177
H  -1.54115  2.27627 -1.99592
H  -3.70774  2.84445 -3.15750
H  -5.68941  1.31429 -2.83661
H  -0.85791 -2.71715  1.92617
H  -2.65189 -4.44787  2.30570
H  -4.89074 -4.13691  1.18523
H  -6.35932 -2.74941 -0.32939
H  -6.64405 -0.75172 -1.78702
H  -0.41437 -1.80390 -4.88799
H  -0.53957 -2.94385 -3.49477
H   1.05171 -2.33784 -4.02069
H   4.24241  0.78573  0.14138
H   5.40971 -0.20027 -3.91041
H   3.90001 -2.19996 -3.83971
H   2.55322 -2.67640 -1.80103
H   5.56836  1.29776 -1.90547
H   3.81717 -1.71547  2.04651
O   1.15986 -0.52513  0.80302
H   0.92058 -0.48658  1.77094
O   2.07277 -2.78903  0.74585
C   2.92953 -3.70540  1.16890
O   4.03444 -3.46293  1.70156
C   2.45758 -5.13097  0.93264
H   1.39515 -5.23975  1.20085
H   3.06962 -5.83800  1.50955
H   2.55016 -5.37269 -0.14038
O   1.26913 -0.48453  3.55456
H   2.16378 -0.66529  3.10472
C   0.90815 -1.66214  4.27958
H  -0.14793 -1.57242  4.58410
H   1.01730 -2.58239  3.67128
H   1.51750 -1.78618  5.19764
```

Int(3^+-8^+)9_{MeOH}

```
SCF  =      -2093.14654397
H(0 K)=     -2092.585176
H(298 K)=   -2092.543496
G(298 K)=   -2092.663398
SCF(D3BJ) =        -2093.34220374
SCF(BS2) =         -3537.16577117
SCF(BS2+D3BJ) =    -3537.36143093
Low Freq. = 13.0530cm-1,
16.2753cm-1
```

```
C   2.56095  3.38062 -2.41538
C   1.76235  3.77026 -1.30657
C   1.00324  2.74893 -0.66812
N   1.02520  1.44719 -1.07984
C   1.79364  1.09690 -2.12070
C   2.57591  2.04805 -2.81644
C   0.17664  3.05925  0.46405
C   0.11384  4.39559  0.95334
C   0.88577  5.41443  0.28863
C   1.67699  5.11475 -0.79667
C  -0.71764  4.63162  2.08040
C  -1.42021  3.57189  2.64489
C  -1.29876  2.27818  2.08809
N  -0.51963  2.02485  1.02702
Cu -0.24657  0.25540 -0.04506
C   2.36860 -1.43618  1.26433
```

C 2.13834 -2.09064 2.49858
C 2.54127 -1.53291 3.72603
C 3.19898 -0.29084 3.75000
C 3.45799 0.37567 2.53902
C 3.04998 -0.19522 1.31994
N -2.04222 0.35383 -1.39485
C -3.09235 -0.27082 -0.78749
C -4.39376 -0.34507 -1.37042
C -4.57163 0.27077 -2.63848
C -3.49520 0.90493 -3.24843
C -2.24309 0.92322 -2.58944
C -2.85341 -0.88582 0.49908
C -3.91554 -1.56652 1.16873
C -5.21613 -1.61749 0.55366
C -5.44690 -1.02873 -0.66665
C -3.62415 -2.16559 2.42279
C -2.33865 -2.07904 2.94555
C -1.34365 -1.38598 2.22161
N -1.59816 -0.80209 1.04001
O 0.69049 -1.38028 -0.71500
B 1.87215 -2.11393 -0.12842
O 1.50877 -3.53368 -0.02295
O 2.99115 -1.91479 -1.19990
C 3.96909 -2.79597 -1.35847
C 5.03918 -2.33965 -2.33472
O 4.03554 -3.89888 -0.77537
O -0.38387 -3.56502 -1.80901
H 1.79757 0.03253 -2.37060
H 3.18733 1.71895 -3.65984
H 3.16130 4.12986 -2.94063
H -1.84235 1.43042 2.51257
H -2.06658 3.71863 3.51329
H -0.79773 5.64122 2.49483
H 0.83111 6.43932 0.66766
H 2.25835 5.89846 -1.29160
H -1.37731 1.41355 -3.04636
H -3.59868 1.38596 -4.22417
H -5.55293 0.23756 -3.12229
H -0.31678 -1.30779 2.59311
H -2.08145 -2.53533 3.90416
H -4.41517 -2.69374 2.96418
H -6.01999 -2.13966 1.08133
H -6.43895 -1.07361 -1.12625
H 1.64444 -3.06980 2.48331
H 3.51905 0.14762 4.70165
H 3.98877 1.33493 2.54525
H 3.28687 0.32724 0.38569
H 2.35112 -2.07017 4.66253
H 0.08710 -2.04319 -1.17519
H 2.37445 -4.00859 -0.14333
H 4.58020 -2.04342 -3.29192
H 5.55560 -1.45167 -1.93295
H 5.76981 -3.14222 -2.50317
H 0.37076 -3.82368 -1.18396
C -1.58918 -4.15549 -1.30579
H -1.59399 -5.25280 -1.45367
H -1.74222 -3.95109 -0.22825
H -2.43822 -3.73033 -1.86473

TS3(3^{+}-8^{+})$_{MeOH}$
SCF = -2093.11261438
H(0 K)= -2092.553358
H(298 K)= -2092.511614
G(298 K)= -2092.630559
SCF(D3BJ) = -2093.31613091
SCF(BS2) = -3537.12955190
SCF(BS2+D3BJ) = -3537.33306842
Low Freq. = -219.4521cm-1, 17.6085cm-1

C -3.98677 -2.33634 -2.18680
C -3.32256 -2.75464 -1.00236
C -2.22531 -1.95607 -0.55181
N -1.80521 -0.84140 -1.21210
C -2.45982 -0.46720 -2.31820
C -3.55960 -1.18774 -2.84314
C -1.52880 -2.32136 0.66472
C -1.94606 -3.48206 1.38844
C -3.04359 -4.27172 0.89436
C -3.70881 -3.92168 -0.25541
C -1.25265 -3.80293 2.58473
C -0.21143 -2.98839 3.01057
C 0.13751 -1.86024 2.23763
N -0.49423 -1.53399 1.09991
Cu 0.03376 0.07291 -0.04600
C -1.25076 1.59456 1.13037
C -0.58503 2.03328 2.30191
C -1.16944 1.90659 3.57305
C -2.45175 1.34157 3.69825
C -3.14953 0.92962 2.54809
C -2.55178 1.05199 1.28288
N 1.47758 -1.01282 -1.25184
C 2.80384 -1.01988 -0.92121
C 3.78101 -1.66983 -1.74361
C 3.33340 -2.31246 -2.92725
C 1.98303 -2.28984 -3.24763
C 1.08947 -1.62586 -2.37951
C 3.23272 -0.34951 0.29454
C 4.62362 -0.36456 0.64106
C 5.57394 -1.02712 -0.21062
C 5.16734 -1.65610 -1.36047
C 5.01111 0.28816 1.84085
C 4.04344 0.90134 2.62500
C 2.69643 0.86145 2.19518
N 2.28991 0.26553 1.06554
O 0.16304 1.63986 -1.24555
B -0.71003 2.56781 -0.53249
O -0.05885 3.77391 -0.15588

```
O   -1.99523  2.70069 -1.27281
C   -2.78781  3.77103 -1.11940
C   -4.09355  3.64743 -1.87236
O   -2.49442  4.76418 -0.43165
O    2.14104  3.32363 -1.58161
H   -2.09958  0.44722 -2.80026
H   -4.05425 -0.83648 -3.75242
H   -4.83078 -2.92140 -2.56599
H    0.95425 -1.20088  2.53759
H    0.34247 -3.20069  3.92805
H   -1.54882 -4.68608  3.15945
H   -3.34110 -5.15791  1.46342
H   -4.54684 -4.52392 -0.62013
H    0.02105 -1.58451 -2.60301
H    1.59996 -2.77022 -4.15123
H    4.05936 -2.81610 -3.57340
H    1.91325  1.32525  2.80273
H    4.30037  1.40769  3.55896
H    6.06604  0.29499  2.13334
H    6.62934 -1.01907  0.07960
H    5.89103 -2.16057 -2.00832
H    0.37807  2.54468  2.20207
H   -2.91332  1.24038  4.68640
H   -4.16130  0.51855  2.63903
H   -3.11159  0.73861  0.39600
H   -0.63820  2.26590  4.46169
H    1.07668  2.06906 -1.37168
H   -0.78446  4.44820 -0.06605
H   -3.89961  3.42116 -2.93328
H   -4.67936  2.80806 -1.46199
H   -4.67051  4.57728 -1.78557
H    1.47994  3.85120 -1.05393
C    3.40407  3.35901 -0.89433
H    3.83349  4.37744 -0.90913
H    3.31984  3.02383  0.15598
H    4.08939  2.68278 -1.42735
```

TS4(3^{+}-8^{+})

```
SCF  =      -1977.35579017
H(0 K)=     -1976.849767
H(298 K)=   -1976.811510
G(298 K)=   -1976.920537
SCF(D3BJ) =        -1977.55234701
SCF(BS2) =         -3421.32760644
SCF(BS2+D3BJ) =    -3421.52419044
Low Freq. = -262.2718cm-1,
15.2165cm-1

C   -2.98037 -3.24033  2.05063
C   -3.50875 -2.33365  1.09514
C   -2.65898 -1.27691  0.63401
N   -1.37434 -1.13441  1.07914
C   -0.91176 -2.00089  1.99098
C   -1.68010 -3.06847  2.50555
C   -3.17779 -0.31284 -0.31671
C   -4.52711 -0.44526 -0.77905
C   -5.34453 -1.53109 -0.30858
C   -4.85351 -2.44034  0.59516
C   -5.00486  0.52849 -1.69513
C   -4.16486  1.56262 -2.08659
C   -2.85059  1.60837 -1.56591
N   -2.35468  0.69897 -0.71698
Cu  -0.12803  0.45275  0.27483
B    0.22288  3.56272  0.31020
O    0.90630  4.67705 -0.22647
C    1.07481  2.00755 -0.61461
C    0.82112  2.07725 -2.01463
C    1.85464  2.08756 -2.96125
C    3.19390  2.02116 -2.53339
C    3.48789  1.94365 -1.15960
C    2.44278  1.91963 -0.22321
N    0.55454 -0.93743 -1.16789
C    1.68600 -1.67560 -0.94871
C    2.18909 -2.58217 -1.93841
C    1.46048 -2.72209 -3.14790
C    0.30239 -1.97988 -3.33836
C   -0.10855 -1.09017 -2.32371
C    3.40203 -3.31562 -1.69019
C    4.09220 -3.16432 -0.51348
C    3.60883 -2.28014  0.51251
C    2.39856 -1.54018  0.31122
N    1.87924 -0.71467  1.26076
C    2.54064 -0.57987  2.41648
C    3.75316 -1.24898  2.70564
C    4.28470 -2.10587  1.74967
O   -1.17300  3.57587  0.09691
H    2.08467  0.08593  3.15806
H    4.24626 -1.09367  3.66884
H    5.21499 -2.65218  1.93598
H   -1.00171 -0.47243 -2.44734
H   -0.28705 -2.06161 -4.25478
H    1.82169 -3.41288 -3.91641
H    3.76427 -3.99563 -2.46758
H    5.01840 -3.71944 -0.33363
H    0.11618 -1.83897  2.32144
H   -1.24009 -3.73925  3.24765
H   -3.60587 -4.05890  2.42105
H   -2.17850  2.42852 -1.82913
H   -4.49913  2.33686 -2.78206
H   -6.02937  0.45648 -2.07434
H   -6.37035 -1.61295 -0.68177
H   -5.47962 -3.26122  0.95863
H   -0.21167  2.15525 -2.37134
H    4.00594  2.03501 -3.26831
H    4.52969  1.90249 -0.82280
H    2.69326  1.87595  0.84213
H    1.62256  2.15590 -4.02983
H    1.86825  4.59815 -0.10254
```

H -1.36550 4.26723 -0.56244
O 0.57270 3.37114 1.76718
C 0.11278 2.40552 2.52388
O -0.39806 1.33627 2.10298
C 0.22387 2.66574 4.00835
H 1.22284 3.06099 4.24960
H -0.51063 3.43930 4.29055
H 0.02322 1.75067 4.58044

Int(3⁺-8⁺)10
SCF = -1405.76016274
H(0 K)= -1405.418698
H(298 K)= -1405.392104
G(298 K)= -1405.476275
SCF(D3BJ) = -1405.87384304
SCF(BS2) = -2849.53727499
SCF(BS2+D3BJ) = -2849.65098795
Low Freq. = 16.2884cm-1,
22.3668cm-1

C -3.90078 -2.48864 0.09755
C -3.72170 -1.07943 0.08141
C -2.41943 -0.59820 -0.22997
N -1.36325 -1.42698 -0.49658
C -1.56314 -2.75312 -0.48846
C -2.82391 -3.31979 -0.19527
C -2.17123 0.81289 -0.29357
C -3.21708 1.74115 -0.03520
C -4.52335 1.23110 0.29471
C -4.76532 -0.12276 0.34902
C -2.88865 3.12072 -0.12966
C -1.59298 3.49835 -0.47055
C -0.61599 2.50670 -0.71697
N -0.90429 1.20134 -0.62432
Cu 0.33031 -0.35710 -0.84527
B 2.86217 -1.20171 0.03327
O 3.72972 -2.29769 0.31652
C 2.34861 -0.32836 1.30225
C 1.39537 -0.87346 2.20289
C 0.96912 -0.17545 3.34635
C 1.49405 1.09750 3.62610
C 2.44827 1.65846 2.75951
C 2.86590 0.95203 1.61908
O 1.68144 -1.77188 -0.79615
H -0.70721 -3.38873 -0.72732
H -2.93095 -4.40668 -0.20208
H -4.88335 -2.90871 0.33295
H 0.41008 2.75618 -0.99870
H -1.31397 4.55099 -0.55487
H -3.65751 3.87518 0.06221
H -5.32636 1.94561 0.49774
H -5.76341 -0.49710 0.59419
H 0.98325 -1.87410 2.01830
H 1.16656 1.64596 4.51569
H 2.87019 2.64630 2.97538
H 3.61901 1.40498 0.96370
H 0.23269 -0.62685 4.02024
H 4.02265 -2.26027 1.24243
H 1.42629 -2.61822 -0.37969
O 3.57017 -0.27908 -1.00463
C 2.95063 0.59513 -1.73348
O 1.69017 0.79122 -1.73315
C 3.80307 1.41632 -2.66676
H 4.81492 1.54022 -2.25631
H 3.88394 0.87995 -3.62843
H 3.33519 2.39187 -2.85759

TS5(3⁺-8⁺)
SCF = -1405.74444435
H(0 K)= -1405.404736
H(298 K)= -1405.378373
G(298 K)= -1405.460739
SCF(D3BJ) = -1405.86102457
SCF(BS2) = -2849.52044774
SCF(BS2+D3BJ) = -2849.63707733
Low Freq. = -220.7611cm-1,
21.9975cm-1

C 4.11471 -2.21971 -0.62861
C 3.86562 -0.87034 -0.25956
C 2.50330 -0.46261 -0.18879
N 1.46412 -1.30491 -0.46030
C 1.72277 -2.57403 -0.80318
C 3.04423 -3.06763 -0.89807
C 2.16684 0.88457 0.18239
C 3.20086 1.81815 0.47766
C 4.57233 1.38405 0.39582
C 4.89252 0.09320 0.04270
C 2.80091 3.13221 0.83978
C 1.44651 3.44526 0.88843
C 0.48728 2.45438 0.57733
N 0.83697 1.20630 0.23446
Cu -0.34565 -0.40138 -0.22739
B -2.64630 -1.32870 -0.67725
O -3.69956 -1.39204 -1.59214
C -2.18875 0.60542 -0.45490
C -2.10983 1.33518 -1.67424
C -2.59436 2.64848 -1.77077
C -3.17975 3.26315 -0.64807
C -3.26644 2.56592 0.57081
C -2.75592 1.26022 0.66932
O -1.40584 -1.92576 -1.18122
H 0.85407 -3.20663 -1.00642
H 3.20557 -4.10993 -1.18276
H 5.14551 -2.58087 -0.69680
H -0.58459 2.66844 0.60777
H 1.10620 4.44646 1.16280
H 3.55969 3.88425 1.07691

```
H   5.36047  2.10810  0.62305
H   5.93832 -0.22305 -0.01410
H  -1.68678  0.86352 -2.56964
H  -3.56557  4.28504 -0.72251
H  -3.72604  3.04058  1.44410
H  -2.82351  0.74127  1.63145
H  -2.52563  3.18961 -2.72029
H  -4.53151 -1.06339 -1.20743
H  -1.38068 -1.85586 -2.15684
O  -2.98579 -1.82165  0.67207
C  -2.11570 -1.88596  1.68015
O  -0.94838 -1.44981  1.61883
C  -2.67457 -2.53894  2.91873
H  -3.61276 -2.04265  3.21454
H  -2.91877 -3.59158  2.69862
H  -1.94506 -2.49129  3.73701
```

Int(3⁺-8⁺)11

```
SCF  =      -1405.75412401
H(0 K)=     -1405.413729
H(298 K)=   -1405.386192
G(298 K)=   -1405.473767
SCF(D3BJ) =       -1405.86526118
SCF(BS2) =        -2849.53259258
SCF(BS2+D3BJ) =   -2849.64377253
Low Freq. = 15.8265cm-1,
25.7489cm-1

C  -3.91294 -2.52031 -0.47097
C  -3.83167 -1.12455 -0.21915
C  -2.53014 -0.54435 -0.19570
N  -1.38857 -1.26694 -0.40245
C  -1.49760 -2.58247 -0.63946
C  -2.74518 -3.24596 -0.68155
C  -2.37868  0.86738  0.04798
C  -3.52988  1.68083  0.26169
C  -4.83244  1.06696  0.23570
C  -4.97739 -0.28134  0.00542
C  -3.31095  3.06562  0.49100
C  -2.01179  3.56234  0.49395
C  -0.92953  2.68096  0.26931
N  -1.10748  1.36982  0.05770
Cu  0.33028 -0.10376 -0.29319
B   2.26514 -1.15895  1.99752
O   2.99500 -0.63381  3.01859
C   1.87733  1.06872 -0.58174
C   2.43705  1.86204  0.44339
C   3.53728  2.70696  0.18817
C   4.08877  2.77395 -1.10137
C   3.54113  1.99109 -2.13235
C   2.44619  1.14314 -1.87264
O   0.89863 -1.01631  1.93832
H  -0.55981 -3.12005 -0.80306
H  -2.77373 -4.31997 -0.88004
H  -4.89143 -3.00981 -0.49845
H   0.10586  3.03398  0.25793
H  -1.81053  4.62251  0.66515
H  -4.16549  3.72793  0.66089
H  -5.70977  1.69910  0.40334
H  -5.97198 -0.73717 -0.01344
H   2.02487  1.83091  1.45986
H   4.94106  3.43181 -1.30270
H   3.96473  2.03725 -3.14244
H   2.04202  0.53826 -2.69403
H   3.95872  3.31071  1.00027
H   3.94894 -0.81930  2.93684
H   0.55648 -0.49070  2.68699
O   2.93836 -1.93750  1.02165
C   2.48630 -2.32543 -0.18774
O   1.47894 -1.85927 -0.74260
C   3.35370 -3.37516 -0.82120
H   4.37881 -2.98350 -0.93103
H   3.41206 -4.25531 -0.16021
H   2.95301 -3.66036 -1.80147
```

TS6(3⁺-8⁺)

```
SCF  =      -1977.35419346
H(0 K)=     -1976.848379
H(298 K)=   -1976.810129
G(298 K)=   -1976.919435
SCF(D3BJ) =       -1977.55198530
SCF(BS2) =        -3421.32654367
SCF(BS2+D3BJ) =   -3421.52434869
Low Freq. = -212.5821cm-1,
16.8868cm-1

C   2.31869 -3.70291 -1.71665
C   2.95299 -2.85218 -0.77467
C   2.30086 -1.62296 -0.43630
N   1.09601 -1.26964 -0.98009
C   0.53346 -2.08900 -1.88021
C   1.11152 -3.31299 -2.28062
C   2.94993 -0.70522  0.47926
C   4.22530 -1.05226  1.02997
C   4.83076 -2.31406  0.69891
C   4.21803 -3.17990 -0.17269
C   4.85222 -0.10201  1.87872
C   4.23116  1.11877  2.10746
C   2.97215  1.36938  1.51192
N   2.32900  0.48055  0.74182
Cu  0.07280  0.42827 -0.17568
B   0.17931  3.15153 -0.51827
O  -0.68818  4.11235 -1.08816
C  -0.90263  2.13840  0.82434
C  -0.46098  2.25189  2.16940
C  -1.35930  2.40374  3.23796
C  -2.74245  2.44669  2.99041
C  -3.21596  2.34257  1.66935
```

```
C  -2.30899  2.17912  0.61225
N  -0.89571 -0.86622  1.11010
C  -2.09102 -1.39746  0.71139
C  -2.81907 -2.30477  1.54455
C  -2.24961 -2.65724  2.79631
C  -1.02868 -2.11170  3.17419
C  -0.38433 -1.20950  2.30072
C  -4.08746 -2.81866  1.09887
C  -4.61577 -2.44715 -0.11325
C  -3.90797 -1.54266 -0.97966
C  -2.63657 -1.02222 -0.58068
N  -1.90706 -0.18145 -1.36708
C  -2.41389  0.18768 -2.55020
C  -3.66910 -0.25861 -3.02781
C  -4.41368 -1.13039 -2.24181
O   1.30434  3.67853  0.15681
H  -1.79538  0.86507 -3.14936
H  -4.03152  0.07812 -4.00250
H  -5.38476 -1.50531 -2.58062
H   0.56840 -0.74447  2.56956
H  -0.56335 -2.35948  4.13115
H  -2.78172 -3.35332  3.45237
H  -4.62608 -3.51031  1.75395
H  -5.58465 -2.83615 -0.44190
H  -0.41801 -1.75834 -2.30033
H   0.59854 -3.93208 -3.02061
H   2.79302 -4.64983 -1.99355
H   2.47789  2.33967  1.62420
H   4.69614  1.88589  2.73230
H   5.82433 -0.33600  2.32463
H   5.79920 -2.56241  1.14483
H   4.68718 -4.13272 -0.43699
H   0.61052  2.24410  2.38815
H  -3.44753  2.56582  3.82000
H  -4.29143  2.39036  1.46560
H  -2.70866  2.10203 -0.40479
H  -0.98077  2.49838  4.26181
H  -1.42697  3.69251 -1.56238
H   1.07097  4.54213  0.54237
O   0.57419  2.00456 -1.42920
C   1.39606  2.07864 -2.53183
O   1.69767  1.05560 -3.13008
C   1.84679  3.46838 -2.92210
H   0.97132  4.12315 -3.05721
H   2.44202  3.90309 -2.10310
H   2.43912  3.41322 -3.84458
```

TS7(3⁺-8⁺)

```
SCF  =       -1405.72064640
H(0 K)=      -1405.382282
H(298 K)=    -1405.355343
G(298 K)=    -1405.439658
SCF(D3BJ) =        -1405.83813567
SCF(BS2) =         -2849.49890456
SCF(BS2+D3BJ) =    -2849.61639029
Low Freq. = -240.7545cm-1,
16.4709cm-1

Cu -0.36500 -0.27038  0.07637
B  -2.70067 -1.08600  0.84653
O  -2.55331 -1.19176  2.23699
C  -2.14764  0.75970  0.31067
C  -2.58174  1.20250 -0.97084
C  -3.12223  2.48528 -1.15278
C  -3.22144  3.36544 -0.05852
C  -2.77760  2.96067  1.21429
C  -2.23310  1.67878  1.39141
N   0.90956  1.28626 -0.14398
C   2.21684  0.88512 -0.08445
C   3.30655  1.78909 -0.23205
C   2.98606  3.15515 -0.45566
C   1.65231  3.54545 -0.51988
C   0.63236  2.58070 -0.35675
C   4.64992  1.27503 -0.14963
C   4.88911 -0.06233  0.06924
C   3.80344 -0.99656  0.22046
C   2.46741 -0.51304  0.13720
N   1.37580 -1.32414  0.26208
C   1.55874 -2.63332  0.48283
C   2.84871 -3.20234  0.58595
C   3.96996 -2.38887  0.45196
O  -3.97763 -1.27199  0.30634
H   0.65547 -3.24189  0.58218
H   2.94589 -4.27509  0.76764
H   4.97782 -2.80904  0.52404
H  -0.42533  2.85394 -0.39730
H   1.37500  4.58774 -0.69279
H   3.78987  3.88795 -0.57566
H   5.48259  1.97513 -0.26500
H   5.91431 -0.43887  0.13185
H  -2.51886  0.52785 -1.83223
H  -3.64118  4.36690 -0.19856
H  -2.85961  3.64280  2.06709
H  -1.91774  1.36529  2.39236
H  -3.46550  2.79975 -2.14392
H  -1.63294 -1.14402  2.54977
H  -4.65782 -0.98190  0.94007
O  -1.63063 -1.87361  0.06968
C  -1.75217 -2.31717 -1.24620
O  -1.02367 -1.81989 -2.09558
C  -2.73342 -3.43501 -1.44418
H  -2.42885 -4.30116 -0.83312
H  -3.72267 -3.11024 -1.08378
H  -2.77012 -3.71663 -2.50448
```

Int3(3⁺-8⁺)13

```
SCF  =       -1405.74996590
H(0 K)=      -1405.409820
```

```
H(298 K)=   -1405.382064
G(298 K)=   -1405.472092
SCF(D3BJ) =        -1405.85619045
SCF(BS2) =         -2849.53117356
SCF(BS2+D3BJ) =   -2849.63740380
Low Freq. = 11.8502cm-1,
20.3149cm-1

Cu -0.27127  0.16312 -0.63756
B  -2.68250 -2.12933  2.07947
O  -2.02473 -2.24499  3.26708
C  -1.73018  1.46108 -0.73679
C  -2.04344  2.04638 -1.98286
C  -3.08600  2.98872 -2.09271
C  -3.82889  3.35548 -0.95776
C  -3.52772  2.77769  0.28670
C  -2.48371  1.83626  0.39549
N   1.12802  1.32665  0.36630
C   2.37599  0.77027  0.34156
C   3.50753  1.39089  0.94599
C   3.29206  2.63658  1.59465
C   2.01574  3.19004  1.61036
C   0.95219  2.50528  0.97899
C   4.78634  0.73265  0.86913
C   4.92671 -0.47406  0.22363
C   3.79967 -1.12084 -0.39771
C   2.52210 -0.49365 -0.33353
N   1.39820 -1.03337 -0.89453
C   1.50167 -2.20551 -1.53911
C   2.72731 -2.89972 -1.65713
C   3.87615 -2.36132 -1.08634
O  -4.03395 -2.24016  1.95723
H   0.57838 -2.60098 -1.97138
H   2.75441 -3.85019 -2.19513
H   4.83667 -2.88040 -1.16253
H  -0.06468  2.90858  0.96252
H   1.81901  4.14639  2.10066
H   4.13141  3.14965  2.07428
H   5.64977  1.21534  1.33678
H   5.90389 -0.96336  0.16974
H  -1.47999  1.77609 -2.88503
H  -4.63892  4.08781 -1.04326
H  -4.10375  3.05573  1.17699
H  -2.26812  1.39796  1.37801
H  -3.31410  3.43440 -3.06798
H  -1.05808 -2.15701  3.17648
H  -4.46364 -2.44198  2.80911
O  -1.90925 -1.81034  0.92200
C  -2.16624 -2.07073 -0.37174
O  -1.51544 -1.44771 -1.23132
C  -3.17366 -3.12039 -0.74403
H  -2.94122 -4.06851 -0.23211
H  -4.17417 -2.80610 -0.40697
H  -3.16598 -3.26528 -1.83126
```

Int(3⁺-8⁺)14

```
SCF  =      -2053.36724760
H(0 K)=     -2052.846757
H(298 K)=   -2052.806257
G(298 K)=   -2052.924007
SCF(D3BJ) =       -2053.55207247
SCF(BS2) =        -3497.38259410
SCF(BS2+D3BJ) =   -3497.56737322
Low Freq. = 10.4932cm-1,
18.2207cm-1

C  -3.83413 -1.90193  0.15248
Cu  0.33050 -0.10994  0.40343
N  -0.12272  0.91954 -1.38241
C  -0.60954  2.19641 -1.31142
C  -0.92448  2.94847 -2.48770
C  -0.70235  2.33184 -3.74691
C  -0.19709  1.03845 -3.79746
C   0.07795  0.36484 -2.58778
C  -4.19033 -3.26730  0.04510
C  -5.21091 -3.70790 -0.81602
C  -5.91351 -2.78082 -1.60685
C  -5.58589 -1.41621 -1.52106
C  -4.56188 -0.99294 -0.65410
C  -1.45301  4.28102 -2.36000
C  -1.66393  4.84244 -1.12425
C  -1.35227  4.11806  0.07910
C  -0.81485  2.79447 -0.00585
N  -0.48260  2.05901  1.09169
C  -0.69021  2.59542  2.30115
C  -1.22144  3.89395  2.49146
C  -1.54814  4.65867  1.37805
B  -2.63012 -1.40267  1.14150
N   1.59099 -1.85010 -0.71528
C   2.91934 -1.55780 -0.71559
C   3.91474 -2.48541 -1.15956
C   3.46723 -3.76524 -1.58550
C   2.10923 -4.06118 -1.55652
C   1.20375 -3.06551 -1.11624
C   5.30082 -2.10005 -1.16201
C   5.68371 -0.85116 -0.73720
C   4.71480  0.10179 -0.26220
C   3.32619 -0.24735 -0.23904
N   2.37300  0.61277  0.23226
C   2.75301  1.82107  0.66757
C   4.09717  2.25635  0.66510
C   5.08116  1.39215  0.20264
O   0.96581 -0.91559  2.12190
C   0.40364 -0.86793  3.28827
O  -0.71911 -0.36225  3.56731
O  -2.23354 -2.44333  2.07981
O  -2.92502 -0.10806  1.73439
C   1.21232 -1.54817  4.40013
```

H -0.44320 1.95096 3.15192
H -1.36517 4.27791 3.50502
H -1.95595 5.66911 1.48559
H 0.47430 -0.65431 -2.58055
H -0.01368 0.53326 -4.74885
H -0.93289 2.88296 -4.66422
H -1.68831 4.83636 -3.27337
H -2.07156 5.85448 -1.03462
H 1.95431 2.46655 1.04306
H 4.34130 3.25614 1.03285
H 6.13501 1.68870 0.19506
H 0.12393 -3.25036 -1.08572
H 1.73430 -5.03837 -1.87262
H 4.19838 -4.50402 -1.93007
H 6.04549 -2.82252 -1.51145
H 6.73862 -0.55878 -0.74384
H 0.87538 -1.20201 5.38815
H 2.29078 -1.36190 4.28049
H 1.05540 -2.63958 4.34203
H -3.65631 -3.99641 0.66641
H -6.71004 -3.11798 -2.28002
H -6.13213 -0.68309 -2.12713
H -4.32897 0.07812 -0.58947
H -5.46292 -4.77413 -0.87065
H -2.14572 0.08207 2.30742
H -1.80755 -1.97239 2.82425
O -1.36261 -1.21211 0.12740
H -1.74810 -0.76834 -0.65366

Int(3^+-8^+)15

SCF = -1824.73029687
H(0 K)= -1824.259015
H(298 K)= -1824.224192
G(298 K)= -1824.327869
SCF(D3BJ) = -1824.89987502
SCF(BS2) = -3268.64538348
SCF(BS2+D3BJ) = -3268.81491443
Low Freq. = 12.5729cm-1, 14.7413cm-1

C 3.23849 -2.31765 -0.34968
Cu -0.13082 -0.05187 -0.20312
N 1.30330 1.20416 0.57513
C 1.12559 2.50333 0.18473
C 1.97576 3.56301 0.61678
C 3.04438 3.22359 1.48828
C 3.21882 1.89834 1.87047
C 2.32710 0.91310 1.38937
C 3.52650 -3.33733 0.59382
C 4.82210 -3.53927 1.10234
C 5.87920 -2.71789 0.67328
C 5.62607 -1.70000 -0.26318
C 4.32460 -1.50826 -0.75944
C 1.71278 4.90088 0.15337
C 0.66449 5.16525 -0.69665
C -0.20599 4.11258 -1.15249
C 0.03066 2.77975 -0.70540
N -0.74401 1.72080 -1.09006
C -1.77076 1.94246 -1.92252
C -2.07976 3.22964 -2.41799
C -1.30064 4.31577 -2.03431
B 1.73849 -2.10670 -0.95993
N -1.70145 -1.14229 -0.93887
C -2.77967 -1.31387 -0.11591
C -3.91737 -2.07697 -0.52430
C -3.88799 -2.66231 -1.81801
C -2.77250 -2.48718 -2.62859
C -1.68918 -1.71898 -2.14898
C -5.03135 -2.22125 0.37575
C -5.01490 -1.64200 1.62166
C -3.88016 -0.87706 2.06712
C -2.75494 -0.71029 1.20119
N -1.64228 -0.00861 1.56108
C -1.60894 0.55122 2.77576
C -2.67223 0.44842 3.70422
C -3.80912 -0.26788 3.34877
O 1.23195 -3.24919 -1.70702
O 1.56448 -0.88572 -1.78699
H -2.36736 1.07200 -2.20654
H -2.92762 3.35039 -3.09626
H -1.52037 5.32235 -2.40333
H 2.45882 -0.13616 1.66149
H 4.03402 1.60032 2.53371
H 3.72146 4.00499 1.84664
H 2.36942 5.70784 0.49225
H 0.47586 6.18527 -1.04463
H -0.69918 1.10606 3.02973
H -2.58588 0.92786 4.68263
H -4.64988 -0.37036 4.04223
H -0.78016 -1.57127 -2.73636
H -2.71585 -2.93126 -3.62516
H -4.74447 -3.25084 -2.16190
H -5.89490 -2.80592 0.04412
H -5.86624 -1.75787 2.29978
H 2.71939 -3.99773 0.94168
H 6.89110 -2.87049 1.06473
H 6.44526 -1.05637 -0.60529
H 4.13297 -0.71199 -1.48907
H 5.00830 -4.33639 1.83157
H 1.55303 -1.15804 -2.72198
H 1.69164 -4.05683 -1.41906
O 0.77059 -1.80703 0.25667
H 1.26054 -1.80579 1.10000

Int(3^+-8^+)16

SCF = -1824.71458634
H(0 K)= -1824.243225
H(298 K)= -1824.208889

G(298 K)=   -1824.309227
SCF(D3BJ) =   -1824.89506865
SCF(BS2) =   -3268.62727407
SCF(BS2+D3BJ) =   -3268.80771070
Low Freq. = 15.4317cm-1,
22.3291cm-1

```
C   2.30214 -3.96058 -0.62085
C   3.14092 -2.85246 -0.33505
C   2.58568 -1.52780 -0.41403
N   1.24519 -1.34237 -0.66690
C   0.49451 -2.42052 -0.95768
C   0.97836 -3.74379 -0.96976
C   3.49074 -0.39494 -0.24229
C   4.85724 -0.64186  0.12348
C   5.34363 -1.98514  0.26933
C   4.51917 -3.05144  0.02300
C   5.71394  0.47773  0.30208
C   5.22532  1.76090  0.10021
C   3.87926  1.91038 -0.30107
N   3.04445  0.87439 -0.46680
Cu -0.20549  0.31777 -0.31762
O   0.66933  1.54426 -1.51072
B   0.20030  2.93971 -0.90160
O  -0.79769  3.50379 -1.78979
C  -0.35218  2.55180  0.61201
C   0.53481  2.11548  1.64340
C   0.10604  1.95484  2.97388
C  -1.23548  2.19980  3.30977
C  -2.14359  2.61213  2.31275
C  -1.70466  2.78431  0.99383
N  -1.30613 -0.71307  1.01215
C  -2.58301 -0.97422  0.58741
C  -3.54081 -1.64111  1.40478
C  -3.12076 -2.04687  2.69981
C  -1.81756 -1.78834  3.10994
C  -0.93407 -1.11426  2.23707
C  -4.86230 -1.87472  0.88102
C  -5.20486 -1.48212 -0.39224
C  -4.25037 -0.82536 -1.24698
C  -2.93980 -0.56871 -0.74822
N  -1.97040  0.04021 -1.49129
C  -2.25075  0.40819 -2.74850
C  -3.52266  0.19544 -3.33029
C  -4.52275 -0.41664 -2.58051
O   1.35451  3.81859 -0.79599
H  -1.43790  0.88930 -3.30204
H  -3.70243  0.51538 -4.35942
H  -5.51520 -0.59097 -3.00753
H   0.09445 -0.89358  2.53162
H  -1.46113 -2.09442  4.09611
H  -3.82417 -2.56370  3.35995
H  -5.59229 -2.37983  1.52057
H  -6.21069 -1.67107 -0.77926
H  -0.55879 -2.22778 -1.18107
H   0.30401 -4.56544 -1.22208
H   2.71651 -4.97252 -0.57092
H   3.42871  2.89303 -0.49233
H   5.85990  2.64154  0.22808
H   6.75694  0.30856  0.58904
H   6.39004 -2.13649  0.55225
H   4.89201 -4.07793  0.09408
H   1.59107  1.95274  1.40205
H  -1.57455  2.07909  4.34401
H  -3.18722  2.81537  2.57700
H  -2.40748  3.14595  0.23386
H   0.81846  1.64862  3.74779
H  -1.54842  2.89415 -1.89535
H   1.62160  1.34203 -1.19049
H   1.48632  4.22604 -1.67300
```

TS8(3^{+}-8^{+})
SCF =   -1824.71046573
H(0 K)=   -1824.241333
H(298 K)=   -1824.206967
G(298 K)=   -1824.309076
SCF(D3BJ) =   -1824.88194910
SCF(BS2) =   -3268.62386338
SCF(BS2+D3BJ) =   -3268.79530548
Low Freq. = -201.4275cm-1,
9.9943cm-1

```
C  -2.00498 -3.07014 -2.65827
C  -2.96937 -2.44117 -1.82714
C  -2.52661 -1.38176 -0.96312
N  -1.22420 -0.96394 -0.92989
C  -0.35433 -1.57946 -1.74151
C  -0.68782 -2.63755 -2.61927
C  -3.51489 -0.72389 -0.11445
C  -4.88860 -1.13244 -0.19123
C  -5.27954 -2.20238 -1.06813
C  -4.35145 -2.83727 -1.85309
C  -5.83624 -0.45043  0.61760
C  -5.41938  0.58209  1.44677
C  -4.04625  0.90807  1.47336
N  -3.12634  0.27908  0.72789
Cu  0.74931  0.70056  0.56092
O  -0.81936  0.98656  1.71935
B  -0.60921  2.43772  1.71793
O   0.29355  2.92441  2.70072
C   0.33554  2.64732 -0.03371
C  -0.44334  2.56513 -1.21929
C  -0.24708  3.46027 -2.28477
C   0.74402  4.45503 -2.19201
C   1.53858  4.54655 -1.03432
C   1.34700  3.64082  0.02307
N   2.41952  0.36870 -0.56265
C   3.11696 -0.73594 -0.15184
```

C	4.33487	-1.15236	-0.76092
C	4.81491	-0.36876	-1.84466
C	4.09141	0.74551	-2.25823
C	2.89364	1.08880	-1.59090
C	4.99681	-2.32862	-0.25637
C	4.46992	-3.04969	0.79092
C	3.23609	-2.65356	1.41968
C	2.56459	-1.49352	0.94015
N	1.39071	-1.04252	1.46830
C	0.83671	-1.71015	2.48822
C	1.43048	-2.87251	3.03358
C	2.62713	-3.34520	2.50203
O	-1.79622	3.18739	1.54637
H	-0.10033	-1.29971	2.87638
H	0.94145	-3.38362	3.86627
H	3.10415	-4.24243	2.90856
H	2.30471	1.96245	-1.88331
H	4.43278	1.36441	-3.09123
H	5.74647	-0.64911	-2.34600
H	5.93384	-2.64054	-0.72736
H	4.98318	-3.94149	1.16282
H	0.67794	-1.21456	-1.70509
H	0.08345	-3.09023	-3.24817
H	-2.31694	-3.88289	-3.32254
H	-3.66200	1.71765	2.10389
H	-6.12442	1.13221	2.07496
H	-6.88909	-0.74779	0.57161
H	-6.33371	-2.49613	-1.09580
H	-4.64730	-3.65152	-2.52261
H	-1.22362	1.79858	-1.29988
H	0.89680	5.15605	-3.01942
H	2.30758	5.32327	-0.95654
H	1.95263	3.73409	0.93091
H	-0.86682	3.38527	-3.18530
H	0.94480	2.24697	2.95547
H	-1.68523	0.69008	1.19444
H	-1.63863	4.11121	1.81185

Int(3^{+}-8^{+})17

SCF = -1824.73419448
H(0 K)= -1824.263836
H(298 K)= -1824.228698
G(298 K)= -1824.335060
SCF(D3BJ) = -1824.90217044
SCF(BS2) = -3268.64870585
SCF(BS2+D3BJ) = -3268.81670196
Low Freq. = 7.2469cm-1, 13.8614cm-1

C	-1.74150	-2.88920	-2.93390
C	-2.73025	-2.43825	-2.02013
C	-2.36633	-1.41471	-1.07781
N	-1.11033	-0.87208	-1.04665
C	-0.21570	-1.32166	-1.93777
C	-0.47489	-2.32676	-2.89801
C	-3.38749	-0.93778	-0.14859
C	-4.71066	-1.49169	-0.22229
C	-5.01961	-2.52007	-1.17740
C	-4.06142	-2.97972	-2.04278
C	-5.70008	-0.99694	0.66793
C	-5.37328	-0.00040	1.57599
C	-4.04718	0.48015	1.59360
N	-3.08716	0.03207	0.76943
Cu	0.76348	0.79039	0.29618
O	-0.82727	0.94282	1.65742
B	-0.88574	2.11194	2.40813
O	0.21720	2.56907	3.10137
C	0.33740	2.49111	-0.59325
C	-0.83297	2.62437	-1.37128
C	-1.15454	3.84829	-1.99071
C	-0.31428	4.96364	-1.83134
C	0.84981	4.84906	-1.05356
C	1.17327	3.62024	-0.44238
N	2.61912	0.45427	-0.64109
C	3.28741	-0.62094	-0.12487
C	4.57298	-1.02645	-0.58851
C	5.15630	-0.25913	-1.63225
C	4.46398	0.83065	-2.14988
C	3.19154	1.15845	-1.62821
C	5.20024	-2.17545	0.01203
C	4.57712	-2.88234	1.01433
C	3.27693	-2.49607	1.49863
C	2.63600	-1.35907	0.92641
N	1.40707	-0.91626	1.32474
C	0.76997	-1.58356	2.29743
C	1.32259	-2.72586	2.92132
C	2.57531	-3.18322	2.52542
O	-2.07394	2.81004	2.48000
H	-0.20568	-1.18244	2.58504
H	0.75804	-3.23212	3.70804
H	3.02607	-4.06367	2.99397
H	2.61848	2.01099	-2.00348
H	4.88457	1.43923	-2.95401
H	6.14257	-0.53325	-2.01955
H	6.18770	-2.47806	-0.34947
H	5.06251	-3.75512	1.46172
H	0.77576	-0.85640	-1.89854
H	0.31264	-2.63953	-3.58878
H	-1.99717	-3.66983	-3.65799
H	-3.73808	1.26678	2.29078
H	-6.11107	0.40774	2.27110
H	-6.71307	-1.40988	0.62137
H	-6.03694	-2.92359	-1.19821
H	-4.29351	-3.76137	-2.77340
H	-1.50451	1.76680	-1.49665
H	-0.56565	5.91639	-2.31004
H	1.51093	5.71348	-0.92041
H	2.09219	3.55625	0.15444

H -2.06564 3.92868 -2.59558
H 0.97570 1.96420 3.01352
H -1.72076 0.60066 1.18796
H -2.00001 3.57162 3.08387

Int(3⁺-8⁺)18
SCF = -1253.07106785
H(0 K)= -1252.766966
H(298 K)= -1252.743647
G(298 K)= -1252.819234
SCF(D3BJ) = -1253.17427955
SCF(BS2) = -2696.79322408
SCF(BS2+D3BJ) = -2696.89652256
Low Freq. = 16.7078cm-1,
29.6810cm-1

Cu 0.52514 -0.57484 -0.08300
O 1.50925 -2.23826 -0.17297
B 2.96519 -1.58802 -0.42522
O 3.30628 -1.75926 -1.80936
C 2.72351 -0.00093 0.05798
C 2.44385 0.32555 1.42532
C 2.62335 1.62718 1.92633
C 3.10248 2.63752 1.07651
C 3.37370 2.35462 -0.28137
C 3.16215 1.06535 -0.78290
N -0.56277 1.09341 -0.28898
C -1.90556 0.84930 -0.15226
C -2.88726 1.87767 -0.21527
C -2.42379 3.20256 -0.43445
C -1.06058 3.43412 -0.58214
C -0.15407 2.35331 -0.50376
C -4.27533 1.52195 -0.06373
C -4.66107 0.21446 0.12376
C -3.68795 -0.84560 0.17610
C -2.31019 -0.51377 0.04507
N -1.31891 -1.45022 0.08081
C -1.63800 -2.74217 0.22923
C -2.98034 -3.16454 0.36785
C -4.00327 -2.22102 0.34589
O 3.89815 -2.21345 0.47006
H -0.80771 -3.45344 0.22976
H -3.19405 -4.22884 0.48906
H -5.04816 -2.52817 0.45219
H 0.92215 2.50359 -0.61494
H -0.67137 4.43926 -0.75843
H -3.14150 4.02659 -0.48996
H -5.02313 2.31933 -0.10600
H -5.71902 -0.04153 0.23231
H 2.14557 -0.46928 2.12136
H 3.26618 3.64753 1.46555
H 3.75616 3.14500 -0.93563
H 3.39823 0.83754 -1.82804
H 2.41634 1.84544 2.97867
H 2.59432 -1.46320 -2.40259
H 1.56350 -2.66933 0.70642
H 4.29983 -2.96795 -0.00019

TS9(3⁺-8⁺)
SCF = -1253.06912279
H(0 K)= -1252.766409
H(298 K)= -1252.743250
G(298 K)= -1252.818247
SCF(D3BJ) = -1253.17220025
SCF(BS2) = -2696.79079975
SCF(BS2+D3BJ) = -2696.89390456
Low Freq. = -156.4905cm-1,
19.3072cm-1

Cu -0.46947 -0.64453 -0.06626
O -1.34636 -2.41699 -0.22835
B -2.65914 -1.88897 0.32067
O -2.79980 -2.09091 1.71711
C -2.47546 -0.01535 0.00335
C -2.71590 0.46351 -1.31833
C -3.38096 1.67789 -1.54795
C -3.82032 2.45233 -0.45787
C -3.58091 2.01460 0.85876
C -2.89707 0.80902 1.08297
N 0.51263 1.12165 0.07607
C 1.87172 0.95329 0.05463
C 2.78603 2.04202 0.12159
C 2.23069 3.34714 0.20416
C 0.84862 3.50357 0.21488
C 0.01425 2.36450 0.15039
C 4.19953 1.76320 0.10102
C 4.67150 0.47309 0.01783
C 3.76669 -0.64494 -0.05632
C 2.36644 -0.39240 -0.04141
N 1.43387 -1.38565 -0.11451
C 1.83849 -2.66026 -0.19394
C 3.20919 -3.00726 -0.21142
C 4.17225 -2.00444 -0.14527
O -3.72384 -2.33899 -0.49353
H 1.04945 -3.41622 -0.24054
H 3.49264 -4.06015 -0.27769
H 5.23816 -2.25183 -0.15985
H -1.07492 2.45345 0.16695
H 0.39089 4.49352 0.27518
H 2.89359 4.21616 0.25738
H 4.89597 2.60533 0.15388
H 5.74749 0.27620 0.00522
H -2.41174 -0.13952 -2.18282
H -4.34701 3.39590 -0.63376
H -3.92921 2.61248 1.70764
H -2.73821 0.46585 2.11062
H -3.56620 2.01681 -2.57260
H -1.98429 -1.90624 2.21524

```
H  -1.46439 -2.66573 -1.16920
H  -4.57590 -2.17384 -0.05279
```

Int(3⁺-8⁺)19

```
SCF  =      -1253.09224301
H(0 K)=     -1252.787837
H(298 K)=   -1252.763796
G(298 K)=   -1252.843740
SCF(D3BJ) =       -1253.19005628
SCF(BS2) =        -2696.81516096
SCF(BS2+D3BJ) =   -2696.91303100
Low Freq. = 18.6601cm-1,
22.0466cm-1

Cu -0.54142 -0.29025 -0.23232
O  -1.55138 -2.26287 -0.21149
B  -2.33072 -2.53175  0.93897
O  -1.74482 -2.54458  2.17647
C  -2.23300  0.65983 -0.38275
C  -2.79387  0.87053 -1.66103
C  -4.02395  1.54470 -1.80001
C  -4.70132  2.01852 -0.66405
C  -4.14883  1.81735  0.61171
C  -2.91843  1.14348  0.75175
N   0.69454  1.31134  0.20061
C   2.01502  0.96675  0.13218
C   3.06547  1.90368  0.35302
C   2.68647  3.23869  0.65865
C   1.33732  3.57137  0.72686
C   0.36146  2.57706  0.48691
C   4.43019  1.45328  0.25694
C   4.72644  0.14365 -0.04294
C   3.68244 -0.82238 -0.27020
C   2.32408 -0.40442 -0.17941
N   1.26874 -1.25178 -0.37788
C   1.52194 -2.53619 -0.67340
C   2.83747 -3.04027 -0.78816
C   3.91792 -2.18754 -0.58678
O  -3.66427 -2.77233  0.75194
H   0.64705 -3.17642 -0.81434
H   2.98626 -4.09461 -1.03242
H   4.94549 -2.55498 -0.66921
H  -0.70919  2.79739  0.52450
H   1.01580  4.58904  0.96035
H   3.45894  3.99312  0.83684
H   5.23163  2.17824  0.42807
H   5.76695 -0.18712 -0.11425
H  -2.28161  0.51416 -2.56361
H  -5.65551  2.54502 -0.77308
H  -4.67148  2.18357  1.50274
H  -2.50539  0.99905  1.75783
H  -4.44728  1.69982 -2.79904
H  -0.77719 -2.43775  2.15185
H  -2.11180 -2.28158 -1.01371
H  -4.11808 -2.97827  1.58957
```

Int(3⁺-8⁺)20

```
SCF  =      -2059.37887879
H(0 K)=     -2058.763992
H(298 K)=   -2058.722816
G(298 K)=   -2058.836647
SCF(D3BJ) =       -2059.60507708
SCF(BS2) =        -3503.36110578
SCF(BS2+D3BJ) =   -3503.58730404
Low Freq. = 17.7431cm-1,
19.8170cm-1

C   3.06153 -3.30436 -2.31491
C   3.44786 -2.61167 -1.13621
C   2.50568 -1.69927 -0.57902
N   1.27369 -1.47843 -1.13443
C   0.92522 -2.15919 -2.23625
C   1.80258 -3.07825 -2.85787
C   2.82882 -0.97754  0.62044
C   4.09367 -1.17121  1.25042
C   5.03205 -2.09047  0.66166
C   4.72055 -2.78308 -0.48518
C   4.34351 -0.43550  2.43916
C   3.36411  0.42060  2.93066
C   2.13892  0.54725  2.23653
N   1.87522 -0.13130  1.11197
Cu  0.19380 -0.02672 -0.20095
C  -2.02898 -2.17066  1.26438
C  -3.12328 -2.03036  2.15278
C  -2.97210 -2.11522  3.54855
C  -1.70304 -2.34910  4.10775
C  -0.59747 -2.51324  3.25610
C  -0.76860 -2.43074  1.86141
N   1.05573  1.75721 -1.27147
C   0.71402  2.88736 -0.58608
C   1.17857  4.18696 -0.95137
C   2.04223  4.27357 -2.07638
C   2.38870  3.11601 -2.76410
C   1.87024  1.87377 -2.32737
C  -0.16779  2.74135  0.54965
C  -0.57691  3.89480  1.28545
C  -0.09016  5.19009  0.88812
C   0.75701  5.33039 -0.18525
C  -1.45369  3.69425  2.38475
C  -1.87870  2.40877  2.70201
C  -1.42533  1.31801  1.92664
N  -0.59329  1.48468  0.88677
O  -1.61722 -0.68269 -0.86348
B  -2.22752 -2.07629 -0.35731
O  -3.61531 -1.94755 -0.81512
O  -1.58805 -3.19373 -1.04941
H  -0.08180 -1.97994 -2.60878
H   1.47097 -3.60215 -3.75742
```

H 3.75588 -4.01134 -2.77949
H 1.35443 1.21221 2.60601
H 3.52110 0.99761 3.84502
H 5.30021 -0.55062 2.95795
H 6.00104 -2.23120 1.14979
H 5.43853 -3.48292 -0.92327
H 2.12140 0.94669 -2.85299
H 3.04863 3.14924 -3.63452
H 2.42373 5.25018 -2.39066
H -1.73601 0.28972 2.13966
H -2.55431 2.22307 3.54008
H -1.78674 4.55530 2.97249
H -0.41087 6.06450 1.46241
H 1.12230 6.31936 -0.47876
H -4.11756 -1.85786 1.72627
H -1.57932 -2.41478 5.19430
H 0.39465 -2.71380 3.67691
H 0.11205 -2.58559 1.22261
H -3.84485 -2.00380 4.20286
C -3.92466 -0.64731 -1.32826
C -2.53650 -0.10150 -1.84821
C -4.97819 -0.80654 -2.43936
H -4.65106 -1.52812 -3.20336
H -5.91791 -1.18162 -1.99904
H -5.19765 0.15683 -2.93175
C -4.51892 0.22993 -0.20354
H -4.87077 1.20647 -0.57811
H -5.38368 -0.29674 0.23411
H -3.78404 0.40344 0.59849
C -2.43986 1.42719 -1.86930
H -1.45195 1.74603 -2.24021
H -3.19996 1.83743 -2.55662
H -2.60415 1.86808 -0.87622
C -2.17550 -0.64579 -3.24450
H -2.24492 -1.74487 -3.27104
H -2.83994 -0.23190 -4.02149
H -1.14573 -0.34347 -3.50097
H -0.99283 -3.65817 -0.43576

TS10(3-8$^+$)
SCF = -2059.35437006
H(0 K)= -2058.740732
H(298 K)= -2058.699510
G(298 K)= -2058.813647
SCF(D3BJ) = -2059.58013489
SCF(BS2) = -3503.33457425
SCF(BS2+D3BJ) = -3503.56033934
Low Freq. = -190.8524cm-1,
14.6476cm-1

C 3.57500 -2.26058 -2.66546
C 3.93777 -1.34837 -1.64006
C 2.88754 -0.75566 -0.86710
N 1.57007 -1.06196 -1.08620
C 1.25980 -1.92580 -2.06766
C 2.23371 -2.54375 -2.88331
C 3.22991 0.19403 0.17687
C 4.60761 0.50139 0.42054
C 5.63368 -0.12095 -0.37163
C 5.30920 -1.00975 -1.36635
C 4.89781 1.43034 1.45504
C 3.85474 1.99815 2.17557
C 2.52658 1.63346 1.85123
N 2.21516 0.76129 0.88642
Cu 0.07915 -0.09093 0.13630
C -0.25454 -1.64231 1.47172
C -1.15698 -1.53823 2.56214
C -0.92073 -2.16366 3.80232
C 0.25988 -2.89193 4.00948
C 1.19125 -2.99988 2.96001
C 0.93638 -2.38326 1.72523
N -0.15154 1.41128 -1.35804
C -0.72535 2.56199 -0.89660
C -0.98985 3.68662 -1.73628
C -0.65545 3.56740 -3.11080
C -0.08611 2.38636 -3.57187
C 0.15426 1.33310 -2.66044
C -1.04741 2.62494 0.50709
C -1.57878 3.83300 1.05418
C -1.83836 4.94831 0.18196
C -1.56256 4.87553 -1.16229
C -1.81288 3.87072 2.45375
C -1.50741 2.75459 3.22268
C -1.00752 1.59353 2.58984
N -0.80276 1.51233 1.26642
O -1.61461 -1.49468 -1.13382
B -1.52734 -2.47046 -0.08158
O -2.80162 -2.64680 0.51517
O -0.70469 -3.58355 -0.33243
H 0.19300 -2.12370 -2.19584
H 1.91597 -3.23575 -3.66728
H 4.35769 -2.72682 -3.27260
H 1.68467 2.06944 2.40038
H 4.04139 2.71612 2.97852
H 5.93988 1.68720 1.67148
H 6.67912 0.13108 -0.16697
H 6.09023 -1.47993 -1.97210
H 0.61152 0.39991 -2.99754
H 0.18529 2.25785 -4.62250
H -0.84514 4.40513 -3.78919
H -0.77668 0.69456 3.16792
H -1.65407 2.75123 4.30549
H -2.21868 4.77841 2.91134
H -2.25826 5.86097 0.61580
H -1.76296 5.72752 -1.81910
H -2.10255 -1.00599 2.43252
H 0.45230 -3.37482 4.97363
H 2.11889 -3.56593 3.10346

H 1.67427 -2.50729 0.92744
H -1.66609 -2.07929 4.60148
C -3.76485 -1.78524 -0.15967
C -3.03008 -1.43829 -1.51895
C -5.06629 -2.58213 -0.32851
H -4.89022 -3.54461 -0.83178
H -5.50490 -2.78865 0.66214
H -5.80628 -2.00860 -0.91249
C -4.03770 -0.54421 0.70928
H -4.83787 0.07867 0.27540
H -4.36744 -0.86896 1.71025
H -3.13777 0.08098 0.82183
C -3.34730 -0.05533 -2.09180
H -2.77315 0.10858 -3.01865
H -4.41933 0.01904 -2.34196
H -3.09876 0.74937 -1.38467
C -3.23628 -2.51367 -2.60397
H -3.00501 -3.52048 -2.22056
H -4.27161 -2.51114 -2.98345
H -2.56106 -2.30390 -3.45001
H -0.70652 -4.18429 0.43455

Int(3^+-8^+)21

SCF = -2059.39179419
H(0 K)= -2058.777486
H(298 K)= -2058.735952
G(298 K)= -2058.853477
SCF(D3BJ) = -2059.60019276
SCF(BS2) = -3503.37234844
SCF(BS2+D3BJ) = -3503.58074701
Low Freq. = 9.8344cm-1, 12.4854cm-1

C -0.33036 4.22283 -2.35157
C -1.36452 3.94361 -1.41863
C -1.31272 2.68651 -0.75104
N -0.32169 1.76985 -0.97288
C 0.65677 2.06304 -1.84384
C 0.67627 3.28561 -2.55593
C -2.32086 2.34222 0.21323
C -3.37327 3.25843 0.50356
C -3.40721 4.52029 -0.18971
C -2.44236 4.84890 -1.11361
C -4.33032 2.85774 1.47340
C -4.20355 1.61542 2.08616
C -3.12769 0.77021 1.73148
N -2.20853 1.12223 0.82114
Cu -0.63077 0.02358 0.02988
C 2.36304 0.53003 1.61711
C 2.37996 -0.16472 2.85170
C 2.14712 0.48600 4.07737
C 1.89234 1.86895 4.10119
C 1.88468 2.58878 2.89338
C 2.11927 1.92317 1.67604
N -1.74573 -1.18173 -1.51893
C -2.14114 -2.36767 -0.97142
C -2.87484 -3.35142 -1.70254
C -3.19767 -3.04954 -3.05284
C -2.79396 -1.83654 -3.59907
C -2.06617 -0.92896 -2.79393
C -1.78488 -2.62664 0.40757
C -2.15561 -3.86605 1.01415
C -2.89642 -4.83293 0.24685
C -3.24506 -4.58453 -1.05903
C -1.76568 -4.08000 2.36276
C -1.04396 -3.09993 3.03451
C -0.71977 -1.90063 2.36235
N -1.08852 -1.67006 1.09354
O 3.19351 0.56742 -0.89264
B 2.62600 -0.23837 0.20774
O 3.50624 -1.40753 0.34930
O 1.21092 -0.73585 -0.26588
H 1.46657 1.33036 -1.93548
H 1.49360 3.47776 -3.25514
H -0.33113 5.17449 -2.89196
H -3.00727 -0.21191 2.19521
H -4.92046 1.27769 2.83813
H -5.15654 3.52786 1.73016
H -4.21952 5.21748 0.03634
H -2.47647 5.81123 -1.63334
H -1.73132 0.03257 -3.19711
H -3.02547 -1.57513 -4.63464
H -3.75917 -3.77595 -3.64881
H -0.13631 -1.11394 2.85146
H -0.72293 -3.23724 4.06967
H -2.03392 -5.01807 2.85871
H -3.17624 -5.77468 0.72883
H -3.80833 -5.32547 -1.63474
H 2.60024 -1.23933 2.85072
H 1.71454 2.38243 5.05266
H 1.70521 3.67051 2.90242
H 2.13379 2.50143 0.74443
H 2.17368 -0.08171 5.01510
C 4.45136 -1.42192 -0.74068
C 4.54009 0.10612 -1.13888
C 5.76744 -2.03390 -0.23491
H 6.13032 -1.51573 0.66585
H 5.61367 -3.09637 0.02094
H 6.55167 -1.98333 -1.01017
C 3.89173 -2.30236 -1.88166
H 4.63557 -2.46170 -2.68132
H 3.61058 -3.28690 -1.47210
H 2.99617 -1.85041 -2.34111
C 4.89532 0.36825 -2.60914
H 4.92417 1.45476 -2.80042
H 5.88980 -0.04265 -2.85547
H 4.15481 -0.07815 -3.29099
C 5.50453 0.89411 -0.22594

H   5.28556 0.70210 0.83700
H   6.56040 0.63874 -0.41984
H   5.37275 1.97308 -0.41341
H   1.28885 -1.06500 -1.18385

Int(3^+-8^+)22
SCF =   -2059.37947272
H(0 K)=   -2058.764816
H(298 K)=   -2058.723904
G(298 K)=   -2058.837859
SCF(D3BJ) =   -2059.59710614
SCF(BS2) =   -3503.35548253
SCF(BS2+D3BJ) =   -3503.57311596
Low Freq. = 16.8100cm-1,
21.4374cm-1

C   0.22341 4.84901 -1.84162
C -0.95380 4.46286 -1.14979
C -1.12240 3.07915 -0.79684
N -0.11810 2.16775 -1.02983
C   0.96873 2.57213 -1.71255
C   1.17475 3.89217 -2.16052
C -2.39648 2.67030 -0.21390
C -3.36896 3.67450 0.11308
C -3.11531 5.05805 -0.17766
C -1.95981 5.43320 -0.81185
C -4.59458 3.25212 0.69501
C -4.83062 1.90049 0.90486
C -3.83810 0.97672 0.50873
N -2.66669 1.34600 -0.02882
Cu 0.42840 0.21011 -0.14129
O -1.12863 -0.67805 -0.85201
B -1.27678 -1.89308 0.12807
C -0.36665 -1.48786 1.44118
C -0.72316 -0.40066 2.29602
C -0.02301 -0.13088 3.48741
C   1.07329 -0.92961 3.84895
C   1.46302 -2.00092 3.01809
C   0.75362 -2.27073 1.84058
N   2.07007 0.92992 0.76649
C   3.23701 0.38353 0.30076
C   4.51368 0.74779 0.81845
C   4.54145 1.72148 1.85211
C   3.34814 2.26907 2.31163
C   2.12505 1.84664 1.74459
C   5.69032 0.11930 0.27483
C   5.59855 -0.81879 -0.72752
C   4.32097 -1.20383 -1.26955
C   3.14159 -0.59383 -0.75292
N   1.89049 -0.89664 -1.20575
C   1.74946 -1.81742 -2.16694
C   2.86317 -2.47300 -2.74422
C   4.14612 -2.16591 -2.30117
H   0.72141 -2.05327 -2.45567
H   2.70010 -3.21504 -3.52963
H   5.02140 -2.66021 -2.73430
H   1.17075 2.25628 2.08441
H   3.33649 3.02052 3.10444
H   5.50100 2.03265 2.27647
H   6.66550 0.40466 0.68077
H   6.50080 -1.28954 -1.12976
H   1.73019 1.80955 -1.90250
H   2.08206 4.14085 -2.71589
H   0.35864 5.89786 -2.12411
H -3.96949 -0.10695 0.62405
H -5.76465 1.54501 1.34726
H -5.34452 4.00447 0.96013
H -3.87617 5.79819 0.08898
H -1.77789 6.47991 -1.07467
H -1.59667 0.21212 2.04609
H   1.62049 -0.72672 4.77571
H   2.31362 -2.62961 3.30474
H   1.03848 -3.11914 1.20880
H -0.33944 0.69381 4.13560
H -1.79691 0.05660 -0.59340
O -0.83098 -3.09056 -0.57885
O -2.69536 -2.08418 0.46352
C -1.88449 -4.07739 -0.51010
C -3.18403 -3.19244 -0.33370
C -1.85743 -4.92090 -1.79302
H -1.92169 -4.28950 -2.69261
H -0.91557 -5.49392 -1.84396
H -2.69188 -5.64355 -1.81131
C -1.62408 -4.98806 0.70969
H -0.61703 -5.42883 0.61758
H -1.66041 -4.41084 1.64802
H -2.35370 -5.81347 0.77605
C -3.71581 -2.64577 -1.67615
H -4.17449 -3.43952 -2.29048
H -4.48765 -1.88375 -1.47414
H -2.90677 -2.17143 -2.25413
C -4.32716 -3.87246 0.43334
H -4.01793 -4.15303 1.45184
H -5.18767 -3.18607 0.51343
H -4.66931 -4.78078 -0.09245

TS11(3^+-8^+)
SCF =   -2059.36941353
H(0 K)=   -2058.756507
H(298 K)=   -2058.715771
G(298 K)=   -2058.830455
SCF(D3BJ) =   -2059.57941590
SCF(BS2) =   -3503.34636400
SCF(BS2+D3BJ) =   -3503.55636646
Low Freq. = -209.8848cm-1,
13.1942cm-1

C   1.16497 5.13810 0.18525

C -0.21573 4.82267 0.08488
C -0.58676 3.43888 -0.03266
N 0.34258 2.43339 -0.02867
C 1.63217 2.78227 0.06814
C 2.09866 4.11340 0.17222
C -2.00435 3.11310 -0.15015
C -2.97300 4.17132 -0.10033
C -2.55017 5.53902 0.02689
C -1.21806 5.85314 0.10824
C -4.34840 3.82623 -0.18108
C -4.71879 2.49403 -0.30060
C -3.70140 1.51738 -0.36191
N -2.39374 1.81072 -0.29718
Cu 0.62555 -0.46173 0.02704
O -1.04529 -0.34728 -0.99366
B -1.57762 -1.55412 -0.33467
C -0.65880 -1.35078 1.43145
C -1.17244 -0.34820 2.29623
C -1.24226 -0.55220 3.68605
C -0.77800 -1.75854 4.24070
C -0.23964 -2.75665 3.40525
C -0.16937 -2.54704 2.01911
N 2.35777 -0.52913 1.07277
C 3.46319 -0.56506 0.26529
C 4.78824 -0.67422 0.77498
C 4.93384 -0.72902 2.18714
C 3.80296 -0.67307 2.99623
C 2.52354 -0.57859 2.40323
C 5.88831 -0.71355 -0.15460
C 5.67787 -0.63643 -1.51267
C 4.34869 -0.50809 -2.05284
C 3.24446 -0.47289 -1.15488
N 1.95116 -0.34247 -1.56986
C 1.69537 -0.24850 -2.88057
C 2.72598 -0.27903 -3.84945
C 4.04994 -0.40806 -3.43902
H 0.64145 -0.14897 -3.15756
H 2.46835 -0.20002 -4.90830
H 4.86296 -0.43179 -4.17133
H 1.61265 -0.54781 3.00771
H 3.88400 -0.70616 4.08513
H 5.93327 -0.81106 2.62539
H 6.90282 -0.80403 0.24501
H 6.52326 -0.66701 -2.20663
H 2.35482 1.96011 0.06618
H 3.17110 4.31247 0.24568
H 1.47104 6.18574 0.27264
H -3.93172 0.44901 -0.45149
H -5.76828 2.19406 -0.35582
H -5.10041 4.62134 -0.14396
H -3.31535 6.32135 0.05528
H -0.89206 6.89433 0.20046
H -1.54212 0.59384 1.87421
H -0.82884 -1.91938 5.32272
H 0.12515 -3.69501 3.83742
H 0.24236 -3.32985 1.37349
H -1.65758 0.22660 4.33504
H -1.50801 0.53589 -0.66526
O -1.14281 -2.76723 -0.94392
O -2.97919 -1.60770 -0.05978
C -2.26721 -3.69759 -0.95699
C -3.51623 -2.72901 -0.83042
C -2.20677 -4.49210 -2.26868
H -2.16735 -3.82796 -3.14524
H -1.30367 -5.12552 -2.27709
H -3.08381 -5.15451 -2.36761
C -2.13520 -4.66527 0.23309
H -1.15819 -5.17409 0.17794
H -2.19347 -4.13630 1.19678
H -2.92234 -5.43749 0.20828
C -3.98277 -2.17783 -2.19245
H -4.44469 -2.96314 -2.81403
H -4.73743 -1.39128 -2.02545
H -3.14373 -1.73580 -2.75421
C -4.71074 -3.31290 -0.06795
H -4.43737 -3.59711 0.95934
H -5.52025 -2.56562 -0.01401
H -5.10795 -4.20315 -0.58494

Int(3^{+}-8^{+})23
SCF = -2059.39652351
H(0 K)= -2058.783692
H(298 K)= -2058.741722
G(298 K)= -2058.863212
SCF(D3BJ) = -2059.60014718
SCF(BS2) = -3503.37665096
SCF(BS2+D3BJ) = -3503.58027462
Low Freq. = 7.1604cm-1, 11.5959cm-1

C 0.59663 -4.92192 -1.34183
C 1.78166 -4.15628 -1.18006
C 1.65371 -2.73210 -1.03017
N 0.43266 -2.11215 -1.02329
C -0.65570 -2.87497 -1.20112
C -0.63161 -4.27910 -1.36549
C 2.87466 -1.94052 -0.90921
C 4.14577 -2.60818 -0.92603
C 4.21654 -4.03803 -1.05103
C 3.07420 -4.78480 -1.17650
C 5.32360 -1.82120 -0.82598
C 5.22628 -0.44089 -0.72322
C 3.94154 0.14007 -0.71386
N 2.81071 -0.57756 -0.80120
Cu -0.71094 -0.16304 0.40829
O 0.80429 0.96879 -0.48034
B 1.04244 2.30635 -0.30112
C 0.27886 -0.51186 2.07201

```
C   0.58113 -1.82126  2.50507
C   1.24931 -2.04534  3.72642
C   1.62210 -0.95968  4.53674
C   1.32561  0.34923  4.12012
C   0.66081  0.56827  2.89691
N  -2.60435 -0.70617  1.16787
C  -3.62409 -0.45261  0.29422
C  -4.99317 -0.69178  0.61207
C  -5.27541 -1.21712  1.90141
C  -4.22770 -1.47210  2.78023
C  -2.89952 -1.20158  2.37764
C  -6.00006 -0.39585 -0.37446
C  -5.65975  0.10517 -1.60973
C  -4.28438  0.35250 -1.95830
C  -3.26782  0.07545 -0.99823
N  -1.93891  0.28020 -1.23804
C  -1.57088  0.75893 -2.43480
C  -2.50545  1.05976 -3.45229
C  -3.86193  0.86000 -3.21620
H  -0.49728  0.90898 -2.57537
H  -2.14904  1.44692 -4.40996
H  -4.60567  1.08724 -3.98631
H  -2.04808 -1.38806  3.03874
H  -4.41166 -1.87722  3.77826
H  -6.31221 -1.41658  2.19001
H  -7.04839 -0.58028 -0.12040
H  -6.43391  0.32405 -2.35158
H  -1.61471 -2.34568 -1.21360
H  -1.56596 -4.82964 -1.50303
H   0.67210 -6.00835 -1.45523
H   3.80595  1.22498 -0.63380
H   6.11289  0.19334 -0.64960
H   6.29861 -2.31912 -0.83547
H   5.20263 -4.51304 -1.05314
H   3.12438 -5.87317 -1.28318
H   0.29945 -2.68654  1.89213
H   2.14012 -1.13249  5.48640
H   1.61103  1.20351  4.74550
H   0.44112  1.59908  2.59236
H   1.47657 -3.07071  4.04168
H   1.62573  0.28094 -0.65905
O   0.03833  3.19017  0.06647
O   2.28014  2.90644 -0.49810
C   0.69349  4.48567  0.32107
C   2.03439  4.36111 -0.51720
C  -0.24980  5.60038 -0.13759
H  -0.55164  5.47280 -1.18790
H  -1.15910  5.59743  0.48589
H   0.23225  6.58629 -0.02778
C   0.93565  4.57601  1.83620
H  -0.02620  4.46076  2.36212
H   1.61746  3.78164  2.18228
H   1.36559  5.55114  2.11777
C   1.86667  4.76028 -1.99243
H   1.74182  5.84977 -2.10270
H   2.76723  4.45890 -2.55168
H   0.99521  4.26133 -2.44783
C   3.24754  5.06393  0.09517
H   3.47452  4.68356  1.10225
H   4.13222  4.90287 -0.54269
H   3.07210  6.15074  0.16118
```

Int(3^+-8^+)24

```
SCF  =       -2212.04755686
H(0 K)=      -2211.395766
H(298 K)=    -2211.351048
G(298 K)=    -2211.473929
SCF(D3BJ) =        -2212.28658902
SCF(BS2) =         -3656.08800538
SCF(BS2+D3BJ) =    -3656.32703756
Low Freq. = 11.8652cm-1,
22.3894cm-1

C   0.99484  4.20163 -2.26571
C   0.16984  4.18982 -1.10958
C  -0.14261  2.91938 -0.54433
N   0.32488  1.74412 -1.06995
C   1.11421  1.78288 -2.15261
C   1.46386  2.99952 -2.78271
C  -0.96834  2.83856  0.62807
C  -1.47206  4.03003  1.22825
C  -1.14874  5.30072  0.63387
C  -0.35998  5.37682 -0.49020
C  -2.27079  3.87995  2.39285
C  -2.52187  2.60544  2.88882
C  -1.98224  1.48111  2.22264
N  -1.22636  1.59127  1.12230
Cu -0.40530  0.09105 -0.15644
C   2.42100 -0.50337  1.56680
C   2.56107 -1.44042  2.61935
C   2.40785 -1.07535  3.96903
C   2.10778  0.25630  4.30798
C   1.97740  1.21323  3.28692
C   2.13648  0.83242  1.94163
N  -2.21269 -0.10670 -1.43754
C  -3.08376 -0.97556 -0.84509
C  -4.38127 -1.25256 -1.37053
C  -4.75654 -0.57244 -2.56028
C  -3.86349  0.31311 -3.15323
C  -2.59647  0.51811 -2.55761
C  -2.64699 -1.63917  0.35972
C  -3.50857 -2.57277  1.01056
C  -4.81183 -2.82860  0.45481
C  -5.23270 -2.19196 -0.68864
C  -3.02282 -3.20250  2.18719
C  -1.75050 -2.89605  2.65732
C  -0.95938 -1.95759  1.95721
N  -1.39920 -1.34991  0.84408
```

O   1.19816 -1.13619 -0.64694
B   2.57838 -0.96189 0.01606
O   3.22500 -2.24625 -0.20079
O   3.25905 0.14870 -0.80506
C   4.52358 0.51840 -0.57537
C   4.98329 1.61167 -1.53500
O   5.26129 0.04139 0.29339
H   1.49050 0.82407 -2.50731
H   2.10391 2.97341 -3.66755
H   1.25623 5.15501 -2.73523
H -2.16560 0.47046 2.59596
H -3.12823 2.45375 3.78487
H -2.67834 4.76611 2.88889
H -1.54377 6.20935 1.09803
H -0.11922 6.34697 -0.93501
H -1.87201 1.20592 -3.00511
H -4.12066 0.85016 -4.06945
H -5.74300 -0.75382 -2.99822
H   0.04806 -1.68935 2.29425
H -1.34815 -3.36388 3.55873
H -3.65587 -3.92406 2.71273
H -5.46299 -3.54417 0.96582
H -6.22532 -2.39265 -1.10310
H   2.81353 -2.47606 2.36465
H   1.99104 0.54754 5.35770
H   1.76373 2.25829 3.53971
H   2.06635 1.60055 1.16194
H   2.53056 -1.82628 4.75836
H   5.00008 1.22326 -2.56768
H   4.28116 2.46132 -1.52093
H   5.99052 1.95646 -1.26295
C   2.36555 -3.17716 -0.87773
C   1.28960 -2.26612 -1.59421
C   3.21978 -4.00605 -1.85256
H   3.78992 -3.36021 -2.53741
H   3.94068 -4.61259 -1.27860
H   2.59856 -4.69601 -2.44971
C   1.74085 -4.12353 0.17036
H   1.15140 -4.93313 -0.29281
H   2.55563 -4.58505 0.75289
H   1.09276 -3.57250 0.87058
C -0.07430 -2.93669 -1.77411
H -0.78191 -2.24527 -2.26010
H   0.03225 -3.81690 -2.43122
H -0.50475 -3.27349 -0.82118
C   1.76798 -1.73247 -2.95786
H   2.73437 -1.21360 -2.86858
H   1.86920 -2.55056 -3.69006
H   1.01861 -1.02916 -3.35966

TS12(3⁺-8⁺)

SCF =   -2212.01229633
H(0 K)=   -2211.362373
H(298 K)=   -2211.317570
G(298 K)=   -2211.440445
SCF(D3BJ) =   -2212.25114279
SCF(BS2) =   -3656.05157594
SCF(BS2+D3BJ) =   -3656.29042243
Low Freq. = -247.1671cm-1, 16.2627cm-1

C   0.69693 -3.96282 -2.75123
C   1.62311 -3.73445 -1.70003
C   1.45234 -2.55949 -0.89987
N   0.41962 -1.68426 -1.11642
C -0.43492 -1.92294 -2.12587
C -0.32698 -3.05094 -2.96928
C   2.39844 -2.28617 0.16520
C   3.46767 -3.20700 0.40730
C   3.59881 -4.38392 -0.40865
C   2.71177 -4.63477 -1.42625
C   4.36775 -2.90027 1.46245
C   4.18312 -1.73869 2.20176
C   3.09921 -0.88919 1.87686
N   2.23009 -1.14722 0.89355
Cu 0.30542 0.02846 0.13566
C -1.21283 -0.67892 1.42874
C -1.62893 0.07636 2.55749
C -1.86726 -0.51272 3.81241
C -1.66906 -1.89152 3.99015
C -1.26519 -2.67538 2.89493
C -1.04462 -2.07652 1.64460
N   1.33790 1.16942 -1.35113
C   1.84245 2.34406 -0.87026
C   2.54183 3.28073 -1.69074
C   2.68188 2.96397 -3.06739
C   2.15371 1.77167 -3.54789
C   1.49339 0.89809 -2.65407
C   1.67001 2.61140 0.53495
C   2.25063 3.78586 1.10415
C   2.94402 4.71493 0.25087
C   3.07706 4.47669 -1.09592
C   2.12280 3.96733 2.50595
C   1.46351 3.00239 3.25691
C   0.89686 1.88427 2.60405
N   0.96857 1.69777 1.27688
O -1.87671 0.60418 -1.12852
B -2.62117 -0.09222 -0.11754
O -3.58691 0.75985 0.44844
O -2.99577 -1.43826 -0.51634
C -3.90915 -2.22517 0.11044
C -4.14016 -3.50733 -0.66915
O -4.47043 -1.94586 1.16356
H -1.23015 -1.18450 -2.25165
H -1.05272 -3.18857 -3.77453
H   0.80430 -4.85311 -3.37900
H   2.93453 0.03629 2.43935
H   4.85669 -1.47128 3.02009

H 5.19662 -3.58185 1.67921
H 4.42364 -5.07389 -0.20443
H 2.81619 -5.52699 -2.05163
H 1.08155 -0.05017 -3.00748
H 2.24415 1.49256 -4.60045
H 3.20678 3.65704 -3.73210
H 0.35470 1.12133 3.16867
H 1.36359 3.09346 4.34112
H 2.55467 4.85480 2.97893
H 3.36949 5.61637 0.70240
H 3.60608 5.18735 -1.73827
H -1.82567 1.14639 2.44796
H -1.84177 -2.35384 4.96795
H -1.12146 -3.75528 3.01436
H -0.75238 -2.72161 0.81051
H -2.21321 0.10754 4.64708
H -4.49434 -3.27548 -1.68725
H -3.19226 -4.06052 -0.77481
H -4.88008 -4.13322 -0.15291
C -3.47429 2.07683 -0.17693
C -2.69964 1.75815 -1.51764
C -4.89662 2.61740 -0.37510
H -5.52754 1.90656 -0.92942
H -5.36478 2.79761 0.60694
H -4.87909 3.57527 -0.92241
C -2.69383 3.01422 0.76069
H -2.68156 4.04648 0.37278
H -3.18428 3.02889 1.74812
H -1.65120 2.68309 0.89066
C -1.79077 2.88053 -2.02240
H -1.27245 2.56042 -2.94125
H -2.38788 3.77554 -2.26679
H -1.03219 3.16171 -1.27758
C -3.62891 1.28589 -2.65274
H -4.29583 0.47545 -2.31764
H -4.24876 2.11306 -3.03675
H -3.01425 0.90432 -3.48470

Int(3^+-8^+)25
SCF = -2212.05336632
H(0 K)= -2211.402232
H(298 K)= -2211.357235
G(298 K)= -2211.482423
SCF(D3BJ) = -2212.28096029
SCF(BS2) = -3656.09196700
SCF(BS2+D3BJ) = -3656.31956098
Low Freq. = 7.7655cm-1, 19.4728cm-1

C -5.79501 -0.25868 -1.25538
C -4.98212 -1.30735 -0.74717
C -3.60053 -1.01504 -0.53678
N -3.04966 0.20353 -0.80871
C -3.84193 1.16866 -1.28969
C -5.22431 0.97986 -1.52639
C -2.72598 -2.04192 -0.02252
C -3.24288 -3.34269 0.25534
C -4.64116 -3.60374 0.03290
C -5.47862 -2.62465 -0.44651
C -2.33311 -4.31992 0.73958
C -0.99529 -3.98672 0.91732
C -0.55907 -2.67439 0.62467
N -1.40657 -1.73440 0.17796
Cu -0.87165 0.15389 -0.26820
B 2.79382 -0.95164 -0.60208
O 4.04386 -1.68790 -0.62782
C 2.99043 0.56024 -1.15172
C 2.92486 1.67544 -0.28497
C 3.22953 2.97737 -0.72246
C 3.60937 3.20019 -2.05682
C 3.67896 2.11080 -2.94372
C 3.37388 0.81580 -2.49098
N -0.91845 0.89958 1.68809
C -0.76862 2.25872 1.68997
C -0.82798 3.04497 2.87640
C -1.04919 2.35349 4.09729
C -1.19967 0.97028 4.08270
C -1.13149 0.27413 2.85384
C -0.66749 4.47292 2.77090
C -0.46397 5.07888 1.55224
C -0.39905 4.30646 0.33800
C -0.54765 2.89299 0.42007
N -0.49407 2.08309 -0.67819
C -0.29345 2.62339 -1.88689
C -0.14662 4.01832 -2.06215
C -0.19976 4.85991 -0.95510
O 2.20668 -1.13939 0.72994
H -0.24149 1.92138 -2.72322
H 0.01021 4.41502 -3.06765
H -0.08654 5.94240 -1.06883
H -1.25062 -0.81188 2.81280
H -1.37130 0.40923 5.00428
H -1.10094 2.91274 5.03649
H -0.71348 5.07120 3.68582
H -0.34765 6.16475 1.48576
H -3.36479 2.13148 -1.49916
H -5.82170 1.80605 -1.91965
H -6.86075 -0.43681 -1.42997
H 0.48634 -2.35032 0.73925
H -0.27301 -4.72166 1.28067
H -2.69508 -5.32829 0.96385
H -5.02383 -4.60471 0.25392
H -6.53981 -2.83408 -0.61266
H 2.63716 1.51343 0.76006
H 3.85161 4.21129 -2.40268
H 3.97677 2.27176 -3.98662
H 3.44412 -0.02068 -3.19803
H 3.17274 3.81856 -0.02173

O	1.83329	-1.73039	-1.69660
C	0.79202	-1.35463	-2.35351
O	-0.04693	-0.45060	-2.02403
C	0.52419	-2.08966	-3.65710
H	1.41295	-2.64063	-3.99298
H	-0.30445	-2.80201	-3.50175
H	0.20103	-1.37600	-4.43045
C	4.08713	-2.55971	0.52222
C	3.21851	-1.76326	1.57356
C	5.55582	-2.75807	0.92543
H	6.08850	-3.31218	0.13364
H	6.06755	-1.79401	1.06844
H	5.63734	-3.34233	1.85851
C	3.47105	-3.92490	0.14540
H	4.00348	-4.32216	-0.73515
H	3.56486	-4.66104	0.96238
H	2.40713	-3.82436	-0.12162
C	2.53711	-2.63333	2.63752
H	1.91328	-3.42474	2.19482
H	3.29221	-3.11868	3.27971
H	1.89737	-2.00935	3.28493
C	4.02068	-0.64661	2.27299
H	4.76668	-1.05677	2.97479
H	4.54005	-0.01378	1.53595
H	3.32726	-0.00908	2.84733

TS13(3^+-8^+)

SCF = -2212.01264307
H(0 K)= -2211.363182
H(298 K)= -2211.318456
G(298 K)= -2211.440893
SCF(D3BJ) = -2212.24786406
SCF(BS2) = -3656.04844521
SCF(BS2+D3BJ) = -3656.28366608
Low Freq. = -223.0121cm-1, 15.5418cm-1

C	-3.99325	3.21539	1.74241
C	-2.93301	3.66867	0.91467
C	-1.88750	2.74115	0.60227
N	-1.89716	1.45395	1.06047
C	-2.90471	1.06451	1.85258
C	-3.97268	1.91276	2.22238
C	-0.76336	3.18337	-0.19920
C	-0.72822	4.53501	-0.66983
C	-1.81246	5.42913	-0.36318
C	-2.87362	5.01168	0.40192
C	0.40847	4.93764	-1.42016
C	1.43514	4.02860	-1.63942
C	1.30841	2.71566	-1.12672
N	0.23732	2.28898	-0.44464
Cu	-0.32104	0.07276	0.33821
B	2.79985	-0.71233	0.52898
O	3.76747	-1.69492	0.17012
C	1.17485	-1.22522	-0.51788
C	1.28741	-0.89832	-1.90119
C	1.09521	-1.85250	-2.90868
C	0.78218	-3.18102	-2.56046
C	0.66771	-3.54635	-1.20698
C	0.85539	-2.57983	-0.20783
N	-1.68732	-0.41118	-1.16879
C	-2.56690	-1.44546	-0.99686
C	-3.49534	-1.81291	-2.02443
C	-3.50440	-1.04803	-3.21956
C	-2.61986	0.01362	-3.36009
C	-1.71839	0.29075	-2.31135
C	-4.38041	-2.92977	-1.82337
C	-4.35445	-3.65254	-0.65661
C	-3.45244	-3.29961	0.40672
C	-2.56262	-2.18832	0.25177
N	-1.71167	-1.78750	1.23648
C	-1.69782	-2.47642	2.38393
C	-2.52066	-3.60118	2.62841
C	-3.40405	-4.00938	1.63639
O	3.28580	0.59510	0.23321
H	-1.00610	-2.11769	3.15437
H	-2.45955	-4.12350	3.58674
H	-4.06649	-4.86758	1.78907
H	-0.99110	1.10262	-2.39580
H	-2.59967	0.62727	-4.26385
H	-4.20855	-1.30464	-4.01736
H	-5.07481	-3.19183	-2.62773
H	-5.02614	-4.50484	-0.51324
H	-2.86278	0.03169	2.20503
H	-4.76326	1.53044	2.87289
H	-4.80829	3.89987	1.99882
H	2.11574	1.98574	-1.23563
H	2.33426	4.30856	-2.19469
H	0.46713	5.96172	-1.80295
H	-1.76776	6.45484	-0.74309
H	-3.69097	5.69785	0.64516
H	1.54597	0.12553	-2.19118
H	0.63481	-3.93152	-3.34460
H	0.44135	-4.58339	-0.93602
H	0.78886	-2.88537	0.84160
H	1.19466	-1.57052	-3.96257
O	2.42570	-0.89557	1.99052
C	1.49852	-0.26571	2.66045
O	0.51668	0.34228	2.15796
C	1.67878	-0.29223	4.16143
H	1.98383	-1.29786	4.48875
H	2.49182	0.40284	4.43304
H	0.75635	0.01662	4.67013
C	5.04952	-1.00780	0.11121
C	4.63211	0.45523	-0.33371
C	5.94911	-1.75314	-0.88268
H	6.16985	-2.76219	-0.49568
H	5.46589	-1.86266	-1.86523

H 6.90932 -1.22665 -1.01840
C 5.68820 -1.05029 1.51540
H 5.77834 -2.10236 1.83326
H 6.69688 -0.60374 1.51785
H 5.06847 -0.52273 2.25750
C 5.50340 1.57721 0.24542
H 5.49372 1.57299 1.34576
H 6.54786 1.47734 -0.09682
H 5.12863 2.55632 -0.09752
C 4.54653 0.60446 -1.86330
H 5.54754 0.55626 -2.32363
H 3.91948 -0.18198 -2.31137
H 4.11345 1.58631 -2.11819

TS14(3^{+}-8^{+})
SCF = -2212.01120271
H(0 K)= -2211.362107
H(298 K)= -2211.317239
G(298 K)= -2211.440244
SCF(D3BJ) = -2212.24960403
SCF(BS2) = -3656.04710246
SCF(BS2+D3BJ) = -3656.28550386
Low Freq. = -220.8087cm-1, 14.7350cm-1

C -3.68718 3.22075 1.94726
C -2.76844 3.64591 0.95300
C -1.75191 2.72472 0.54016
N -1.66317 1.46436 1.06717
C -2.53637 1.10252 2.01861
C -3.56295 1.94906 2.48947
C -0.77033 3.14750 -0.44077
C -0.84920 4.47180 -0.98118
C -1.90286 5.35747 -0.56545
C -2.82611 4.95939 0.36901
C 0.14951 4.86023 -1.91285
C 1.16305 3.96764 -2.23476
C 1.15909 2.68395 -1.63882
N 0.21438 2.26778 -0.78392
Cu -0.30263 0.05622 0.20977
B 2.41588 -0.29164 0.50106
O 3.31914 -1.22819 1.06015
C 1.18293 -1.24383 -0.75305
C 1.30823 -0.95196 -2.13753
C 1.25150 -1.95966 -3.11285
C 1.05790 -3.29911 -2.72553
C 0.92898 -3.62120 -1.36210
C 0.98012 -2.60468 -0.39613
N -1.75709 -0.50348 -1.15616
C -2.55690 -1.57088 -0.85350
C -3.55287 -2.04121 -1.76765
C -3.70757 -1.34610 -2.99534
C -2.89437 -0.25416 -3.27309
C -1.92126 0.13028 -2.32640
C -4.35111 -3.18820 -1.42316
C -4.17230 -3.84188 -0.22877
C -3.19154 -3.38760 0.72063
C -2.38615 -2.24322 0.42152
N -1.46197 -1.74701 1.29041
C -1.29307 -2.36750 2.46557
C -2.02448 -3.51770 2.84559
C -2.97847 -4.02576 1.97177
O 3.10703 0.68624 -0.25845
H -0.55675 -1.91783 3.14039
H -1.83922 -3.98283 3.81733
H -3.57173 -4.90763 2.23433
H -1.24696 0.96929 -2.51795
H -2.98542 0.30387 -4.20801
H -4.46501 -1.68060 -3.71123
H -5.10300 -3.53141 -2.14054
H -4.77784 -4.71818 0.02327
H -2.41181 0.09646 2.42021
H -4.24159 1.58894 3.26655
H -4.47627 3.90312 2.27902
H 1.97183 1.97581 -1.82617
H 1.96065 4.23863 -2.93156
H 0.11538 5.86245 -2.35228
H -1.94506 6.36069 -1.00150
H -3.61986 5.63828 0.69616
H 1.47793 0.07988 -2.45955
H 1.01238 -4.08860 -3.48339
H 0.79381 -4.66417 -1.05481
H 0.89848 -2.87688 0.66080
H 1.36422 -1.70504 -4.17253
O 1.35068 0.20766 1.45120
C 1.36696 0.40181 2.81425
O 0.30592 0.44150 3.42328
C 2.72174 0.59486 3.45001
H 3.39012 -0.23363 3.17101
H 3.17197 1.52845 3.07294
H 2.60353 0.65950 4.53952
C 4.57158 -1.13357 0.31444
C 4.52895 0.34874 -0.27780
C 5.72383 -1.39475 1.29436
H 5.70903 -0.69963 2.14741
H 5.64712 -2.42231 1.68723
H 6.69790 -1.29988 0.78524
C 4.57347 -2.23194 -0.76622
H 4.45453 -3.21218 -0.27587
H 3.74859 -2.10732 -1.48386
H 5.52552 -2.24209 -1.32259
C 5.25268 1.38736 0.59849
H 6.34599 1.24344 0.57787
H 5.03301 2.39633 0.21196
H 4.91759 1.34372 1.64618
C 5.03413 0.46671 -1.72313
H 4.44863 -0.16231 -2.41019
H 4.95121 1.51397 -2.05941

H 6.09537 0.17375 -1.79496

$11\text{-}1_{I}$
SCF = -1240.75073287
H(0 K)= -1240.445223
H(298 K)= -1240.419369
G(298 K)= -1240.503467
SCF(D3BJ) = -1240.86610209
SCF(BS2) = -2970.84156409
SCF(BS2+D3BJ) = -2970.95694014
Low Freq. = 18.6977cm-1,
21.0633cm-1

Cu 0.55693 -0.23980 0.18258
N -0.93398 1.57514 0.45754
N -1.28731 -1.11143 0.03408
C -0.76614 2.87688 0.71862
H 0.26670 3.21611 0.84966
C -1.83722 3.79454 0.83320
H -1.62826 4.84636 1.04450
C -3.13751 3.33285 0.67225
H -3.99219 4.01268 0.74836
C -3.36004 1.95402 0.41516
C -2.20976 1.10849 0.32200
C -2.39310 -0.30965 0.09504
C -1.43988 -2.43281 -0.14936
H -0.51765 -3.01822 -0.16360
C -2.70792 -3.03562 -0.29294
H -2.77422 -4.11638 -0.43938
C -3.84584 -2.23871 -0.24591
H -4.84376 -2.67454 -0.35794
C -3.71390 -0.84008 -0.04314
C -4.84498 0.04624 0.03642
H -5.84714 -0.37876 -0.07654
C -4.67504 1.39108 0.25805
H -5.54009 2.05836 0.32526
C 2.36855 0.47662 0.23949
C 3.40641 -0.35425 0.68103
C 2.57375 1.83722 -0.02086
C 4.67295 0.21668 0.91957
C 3.84505 2.39445 0.23230
C 4.89258 1.58716 0.70175
H 3.23214 -1.41787 0.86462
H 1.78289 2.46374 -0.44035
H 5.48452 -0.42400 1.28192
H 4.00731 3.45992 0.03622
H 5.88071 2.02134 0.88500
I 0.98870 -0.40687 -2.34714
O 0.63928 -0.54027 2.12978
O 1.23551 -2.63162 1.48183
C 0.97175 -1.78698 2.36761
C 1.06195 -2.13789 3.85239
H 0.41293 -1.49515 4.46501
H 0.80086 -3.19586 4.00342

H 2.10381 -1.99151 4.18701

$11\text{-}3_{I}$
SCF = -1240.75044609
H(0 K)= -1240.445296
H(298 K)= -1240.419358
G(298 K)= -1240.503520
SCF(D3BJ) = -1240.86359676
SCF(BS2) = -2970.83730500
SCF(BS2+D3BJ) = -2970.95345893
Low Freq. = 22.8923cm-1,
28.9895cm-1

Cu -0.54799 -0.11536 0.41644
N 1.24250 -1.09159 0.50781
N 0.90810 1.53136 -0.23547
C 1.37220 -2.36889 0.88901
H 0.44407 -2.89199 1.13332
C 2.63472 -2.99327 0.98810
H 2.69270 -4.03732 1.30473
C 3.77770 -2.26399 0.67923
H 4.76974 -2.72207 0.74297
C 3.66065 -0.90678 0.27919
C 2.34642 -0.34875 0.20637
C 2.17467 1.03656 -0.18517
C 0.73162 2.81023 -0.58364
H -0.30634 3.16209 -0.60948
C 1.80649 3.67339 -0.90507
H 1.60415 4.71113 -1.18235
C 3.10513 3.17837 -0.86152
H 3.95984 3.81753 -1.10532
C 3.32693 1.82402 -0.49343
C 4.63571 1.23045 -0.41719
H 5.50528 1.84936 -0.65946
C 4.79551 -0.08286 -0.04540
H 5.79350 -0.52783 0.01419
C -0.88412 -0.97875 -1.29240
C -0.42530 -0.46676 -2.50993
C -1.47886 -2.24241 -1.18966
C -0.54668 -1.26876 -3.66431
C -1.59096 -3.03116 -2.35403
C -1.12664 -2.54561 -3.58736
H 0.02031 0.52914 -2.57737
H -1.84982 -2.61700 -0.23009
H -0.18440 -0.88049 -4.62258
H -2.05125 -4.02283 -2.28348
H -1.22188 -3.15937 -4.48886
I -2.98383 1.09571 -0.08224
O -1.36592 -1.57833 2.27687
O -0.42634 0.46012 2.31495
C -0.95694 -0.56903 2.91713
C -1.09491 -0.47598 4.42847
H -1.16002 -1.48057 4.87140
H -0.25426 0.08002 4.87100

```
H  -2.02299  0.07026  4.67207
```

11-4$_{I}$

```
SCF  =      -1240.75110197
H(0 K)=     -1240.445039
H(298 K)=   -1240.419297
G(298 K)=   -1240.503691
SCF(D3BJ) =       -1240.86646184
SCF(BS2) =        -2970.83957030
SCF(BS2+D3BJ) =   -2970.95490925
Low Freq. = 24.0893cm-1,
29.1828cm-1

Cu  0.56690 -0.29976 -0.35456
N  -1.29437 -1.07278 -0.55810
N  -0.39054  1.43603 -0.41974
C  -1.68873 -2.35021 -0.60681
H  -0.90300 -3.10349 -0.50134
C  -3.04719 -2.70449 -0.77428
H  -3.32173 -3.76124 -0.81053
C  -4.01135 -1.70586 -0.88593
H  -5.06808 -1.96012 -1.01283
C  -3.61674 -0.34215 -0.82888
C  -2.22710 -0.08305 -0.66657
C  -1.74251  1.26236 -0.58578
C   0.09671  2.68212 -0.31124
H   1.17617  2.77349 -0.17513
C  -0.73962  3.81822 -0.36993
H  -0.28957  4.80915 -0.27675
C  -2.11044  3.66087 -0.54792
H  -2.77254  4.53045 -0.59969
C  -2.65352  2.35378 -0.65897
C  -4.05588  2.07024 -0.83263
H  -4.75101  2.91279 -0.89303
C  -4.51913  0.77702 -0.91590
H  -5.58734  0.57849 -1.04369
C   2.32220  0.51878 -0.42572
C   2.80916  0.89327 -1.68165
C   3.00057  0.76323  0.76905
C   4.03510  1.59156 -1.72692
C   4.22512  1.46290  0.69550
C   4.73739  1.87548 -0.54465
H   2.27213  0.64628 -2.60052
H   2.59063  0.43903  1.73143
H   4.43166  1.90287 -2.69926
H   4.76926  1.67522  1.62209
H   5.68961  2.41343 -0.59085
I   0.02253 -0.61579  2.78329
O   1.07884 -1.54247 -2.76450
O   1.50281 -1.93467 -0.56855
C   1.55234 -2.24961 -1.85843
C   2.27638 -3.56411 -2.12400
H   3.35892 -3.36185 -2.19635
H   2.12092 -4.28435 -1.30756
```

```
H   1.93421 -3.98773 -3.07883
```

11-5$_{I}$

```
SCF  =      -1240.74564310
H(0 K)=     -1240.440282
H(298 K)=   -1240.414313
G(298 K)=   -1240.499286
SCF(D3BJ) =       -1240.86172460
SCF(BS2) =        -2970.83555905
SCF(BS2+D3BJ) =   -2970.95164664
Low Freq. = 20.4308cm-1,
26.5883cm-1

Cu  0.80134 -0.07689  0.04370
N  -1.00631 -0.15166  1.51154
N  -0.66824  0.38260 -1.16179
C  -1.16346 -0.43347  2.81021
H  -0.25075 -0.66199  3.37089
C  -2.42604 -0.44288  3.45015
H  -2.49175 -0.68037  4.51502
C  -3.56217 -0.14573  2.70584
H  -4.55419 -0.14184  3.16851
C  -3.43333  0.15439  1.32325
C  -2.11434  0.13495  0.77327
C  -1.93076  0.41987 -0.63348
C  -0.47592  0.63241 -2.46712
H   0.55439  0.59105 -2.82812
C  -1.54423  0.93032 -3.33594
H  -1.33553  1.12082 -4.39099
C  -2.83863  0.97606 -2.82929
H  -3.68759  1.20729 -3.47991
C  -3.06397  0.71811 -1.45245
C  -4.37510  0.73798 -0.85857
H  -5.23018  0.97110 -1.50015
C  -4.55387  0.46596  0.47619
H  -5.55557  0.47869  0.91697
C   1.25473  1.83870  0.11125
C   2.08327  2.36829 -0.88118
C   0.71375  2.61004  1.14148
C   2.36602  3.75085 -0.84003
C   1.01060  3.99007  1.16443
C   1.83089  4.55761  0.17668
H   2.52978  1.73631 -1.65260
H   0.07655  2.17150  1.91377
H   3.01507  4.18255 -1.60968
H   0.59119  4.61015  1.96417
H   2.05736  5.62831  0.20211
I   0.49346 -2.72817 -0.56288
O   3.57064 -0.42309 -0.71738
O   2.29355 -0.32970  1.16487
C   3.43792 -0.48316  0.51545
C   4.61485 -0.72546  1.45965
H   4.90221  0.22804  1.93483
H   4.34183 -1.42834  2.26164
```

H   5.47190 -1.11411 0.89219

11-1$_{I}$_T
SCF =   -1240.72437535
H(0 K)=   -1240.419460
H(298 K)=   -1240.393475
G(298 K)=   -1240.478952
SCF(D3BJ) =   -1240.83946658
SCF(BS2) =   -2970.81025398
SCF(BS2+D3BJ) =   -2970.92531862
Low Freq. = 23.5157cm-1,
26.2709cm-1

Cu -0.56001 -0.37537 -0.23241
N   0.56360 1.39085 -0.37108
N   1.47844 -1.15423 -0.25537
C   0.08516 2.64209 -0.37825
H -1.00005 2.74263 -0.28953
C   0.92642 3.77054 -0.50174
H   0.48314 4.76914 -0.50653
C   2.30036 3.58774 -0.61441
H   2.97480 4.44411 -0.71314
C   2.83544 2.27273 -0.59233
C   1.91262 1.19131 -0.46281
C   2.40174 -0.16435 -0.40481
C   1.89938 -2.42199 -0.17966
H   1.11868 -3.17958 -0.05956
C   3.26748 -2.77447 -0.25197
H   3.55664 -3.82623 -0.18500
C   4.22077 -1.77302 -0.40647
H   5.28652 -2.01571 -0.46418
C   3.80386 -0.41731 -0.48592
C   4.70953 0.69185 -0.63517
H   5.78217 0.48563 -0.70292
C   4.24341 1.98457 -0.68526
H   4.93896 2.82260 -0.79246
C -2.44773 0.43493 -0.42830
C -3.49470 0.16307 0.46919
C -2.70309 1.20692 -1.57719
C -4.78527 0.67750 0.22931
C -3.99336 1.72718 -1.81231
C -5.03519 1.46334 -0.90840
H -3.31401 -0.44248 1.36384
H -1.90926 1.40499 -2.30654
H -5.59317 0.46117 0.93831
H -4.17809 2.33129 -2.70835
H -6.03794 1.86431 -1.09160
I -0.44684 -0.32329 2.55193
O -0.67522 -1.10315 -2.29466
O -1.28990 -2.30469 -0.54219
C -1.15528 -2.19078 -1.82252
C -1.59231 -3.31523 -2.72801
H -1.09035 -3.24342 -3.70370
H -1.38226 -4.28957 -2.26157

H -2.68205 -3.24252 -2.88736

11-2$_{I}$_T
SCF =   -1240.72706138
H(0 K)=   -1240.422376
H(298 K)=   -1240.396270
G(298 K)=   -1240.481999
SCF(D3BJ) =   -1240.84226076
SCF(BS2) =   -2970.81398806
SCF(BS2+D3BJ) =   -2970.92918859
Low Freq. = 24.3776cm-1,
29.9191cm-1

Cu 0.50436 -0.23067 -0.34381
N -1.37405 -1.20474 -0.43148
N -0.77068 1.41944 -0.18667
C -1.61950 -2.51473 -0.53983
H -0.74212 -3.15923 -0.64950
C -2.93523 -3.03332 -0.52402
H -3.08779 -4.11118 -0.61833
C -4.01145 -2.16073 -0.39256
H -5.03923 -2.53673 -0.37912
C -3.77339 -0.76522 -0.27467
C -2.41351 -0.33486 -0.29678
C -2.09305 1.06309 -0.17453
C -0.44999 2.71683 -0.07772
H   0.62055 2.94614 -0.09075
C -1.42696 3.73029 0.04625
H -1.10629 4.77165 0.12721
C -2.77370 3.38468 0.06523
H -3.54927 4.15048 0.16451
C -3.14477 2.01850 -0.04485
C -4.50984 1.55857 -0.03125
H -5.30698 2.30152 0.06876
C -4.81290 0.22146 -0.13934
H -5.85410 -0.11483 -0.12629
C   0.75603 -0.69593 1.66498
C   1.31605 -1.94822 1.95998
C   0.31078 0.14676 2.69468
C   1.42058 -2.36575 3.30348
C   0.41686 -0.27660 4.03639
C   0.97087 -1.53127 4.34072
H   1.67157 -2.60238 1.15638
H -0.11492 1.13250 2.47699
H   1.85689 -3.34520 3.53220
H   0.06715 0.38269 4.83946
H   1.05533 -1.85603 5.38329
I   2.90043 1.15703 -0.33659
O   0.57858 -0.41237 -2.64396
O   1.41427 -1.85996 -1.17700
C   1.18812 -1.49678 -2.40840
C   1.70312 -2.39486 -3.51289
H   2.79343 -2.25570 -3.61160
H   1.52340 -3.45330 -3.26887

H 1.22476 -2.13944 -4.46907

11-4$_{I}$_T

SCF = -1240.72439720
H(0 K)= -1240.419413
H(298 K)= -1240.393452
G(298 K)= -1240.478728
SCF(D3BJ) = -1240.83946025
SCF(BS2) = -2970.81024710
SCF(BS2+D3BJ) = -2970.92530514
Low Freq. = 24.5199cm-1,
28.7478cm-1

Cu 0.56176 -0.37475 -0.23481
N -1.47716 -1.15405 -0.26547
N -0.56112 1.39139 -0.37740
C -1.89878 -2.42174 -0.19179
H -1.11842 -3.18013 -0.07453
C -3.26724 -2.77322 -0.26184
H -3.55710 -3.82488 -0.19656
C -4.22023 -1.77077 -0.41155
H -5.28631 -2.01251 -0.46762
C -3.80262 -0.41513 -0.48839
C -2.40013 -0.16313 -0.41018
C -1.91049 1.19247 -0.46592
C -0.08222 2.64250 -0.38614
H 1.00338 2.74290 -0.30200
C -0.92333 3.77149 -0.50504
H -0.47954 4.76988 -0.51070
C -2.29782 3.58945 -0.61203
H -2.97228 4.44637 -0.70564
C -2.83324 2.27458 -0.59088
C -4.24158 1.98737 -0.68084
H -4.93695 2.82588 -0.78544
C -4.70818 0.69479 -0.63219
H -5.78113 0.48918 -0.69693
C 2.45044 0.43671 -0.41969
C 2.71056 1.20568 -1.56960
C 3.49337 0.16901 0.48385
C 4.00125 1.72692 -1.80010
C 4.78434 0.68474 0.24870
C 5.03900 1.46708 -0.89040
H 1.92008 1.40051 -2.30343
H 3.30922 -0.43372 1.37974
H 4.18954 2.32831 -2.69724
H 5.58902 0.47196 0.96242
H 6.04215 1.86869 -1.06996
I 0.43444 -0.32988 2.55031
O 1.29394 -2.30241 -0.54309
O 0.68656 -1.10058 -2.29771
C 1.16483 -2.18826 -1.82394
C 1.60638 -3.31250 -2.72746
H 1.40381 -4.28677 -2.25763
H 1.10136 -3.24656 -3.70201

H 2.69517 -3.23311 -2.89030

11-5$_{I}$_T

SCF = -1240.72071905
H(0 K)= -1240.415932
H(298 K)= -1240.389783
G(298 K)= -1240.476956
SCF(D3BJ) = -1240.83482158
SCF(BS2) = -2970.80597355
SCF(BS2+D3BJ) = -2970.92007354
Low Freq. = 15.1139cm-1,
26.8425cm-1

Cu -0.83095 -0.19112 0.00836
N 0.62867 0.26991 -1.34279
N 0.64437 0.24859 1.34517
C 0.57911 0.24628 -2.68065
H -0.34507 -0.12675 -3.13109
C 1.66983 0.67119 -3.47318
H 1.58412 0.63149 -4.56158
C 2.83121 1.12855 -2.85641
H 3.68663 1.46176 -3.45195
C 2.90672 1.15841 -1.43830
C 1.76061 0.71237 -0.71965
C 1.76898 0.70095 0.71636
C 0.60980 0.20159 2.68292
H -0.30957 -0.17960 3.13641
C 1.70930 0.61371 3.47006
H 1.63595 0.55592 4.55854
C 2.86335 1.08205 2.84764
H 3.72510 1.40660 3.43890
C 2.92316 1.13492 1.42939
C 4.06676 1.59030 0.68074
H 4.94993 1.92607 1.23247
C 4.05884 1.60169 -0.69530
H 4.93566 1.94690 -1.25132
C -1.61464 1.80915 0.01056
C -1.83859 2.46828 1.22582
C -1.95106 2.40947 -1.20942
C -2.40770 3.75990 1.21732
C -2.51927 3.70130 -1.21129
C -2.74614 4.37457 0.00053
H -1.58354 2.00233 2.18406
H -1.78514 1.89595 -2.16285
H -2.58379 4.27787 2.16716
H -2.78281 4.17331 -2.16484
H -3.18707 5.37700 -0.00340
I 0.32396 -3.05605 -0.02410
O -2.42274 -0.88117 1.12468
O -2.45050 -0.87704 -1.07401
C -3.04994 -1.15130 0.03236
C -4.40732 -1.79439 0.04836
H -4.95228 -1.52056 0.96358
H -4.98197 -1.50182 -0.84266

H -4.28233 -2.89103 0.03444

11-1$_{C1}$

SCF =   -1244.31432974
H(0 K)=   -1244.008374
H(298 K)=   -1243.982830
G(298 K)=   -1244.065378
SCF(D3BJ) =   -1244.42345674
SCF(BS2) =   -3133.24975602
SCF(BS2+D3BJ) =   -3133.35888301
Low Freq. = 15.5102cm-1, 21.1830cm-1

```
Cu -0.72782 -0.28988  0.31593
N   0.76373  1.50023 -0.29400
N   1.14110 -1.13286  0.40729
C   0.58500  2.76819 -0.68208
H  -0.45265  3.10674 -0.77390
C   1.64876  3.65477 -0.97405
H   1.43050  4.68006 -1.28373
C   2.95461  3.19604 -0.85713
H   3.80516  3.85208 -1.06844
C   3.18817  1.85128 -0.46450
C   2.04327  1.03513 -0.19735
C   2.23876 -0.35074  0.17893
C   1.30537 -2.42037  0.74905
H   0.38722 -2.99264  0.90091
C   2.57990 -3.01155  0.88475
H   2.65614 -4.06541  1.16300
C   3.71108 -2.23688  0.65777
H   4.71426 -2.66429  0.75374
C   3.56585 -0.87283  0.29256
C   4.69114 -0.01455  0.03144
H   5.69771 -0.43362  0.12653
C   4.50950  1.29641 -0.33521
H   5.36937  1.94241 -0.53899
C  -2.54972  0.38320  0.29435
C  -3.60753 -0.51031  0.10567
C  -2.74259  1.75927  0.43884
C  -4.91502  0.01413  0.02612
C  -4.05698  2.26527  0.34639
C  -5.13929  1.39570  0.14028
H  -3.42859 -1.58343 -0.00063
H  -1.91235  2.43703  0.65143
H  -5.75296 -0.67335 -0.13333
H  -4.21993  3.34302  0.45503
H  -6.15718  1.79346  0.07657
Cl -0.83164 -0.09151  2.54509
O  -0.95435 -0.73188 -1.55660
O  -1.46189 -2.77652 -0.71256
C  -1.27765 -2.00293 -1.67565
C  -1.45517 -2.45914 -3.12172
H  -0.84992 -1.86020 -3.81753
H  -1.19410 -3.52396 -3.20919
```

H -2.51668 -2.34074 -3.40048

11-3$_{C1}$

SCF =   -1244.31381606
H(0 K)=   -1244.008090
H(298 K)=   -1243.982547
G(298 K)=   -1244.064569
SCF(D3BJ) =   -1244.42516711
SCF(BS2) =   -3133.24959324
SCF(BS2+D3BJ) =   -3133.36094429
Low Freq. = 23.6317cm-1, 33.0000cm-1

```
Cu -0.98097 -0.41550  0.47657
N   0.23161 -0.34708 -1.09867
N   1.07706  0.08798  1.48411
C  -0.20571 -0.58359 -2.34427
H  -1.27191 -0.79871 -2.44346
C   0.66761 -0.58284 -3.45191
H   0.26562 -0.78221 -4.44788
C   2.01905 -0.32676 -3.25064
H   2.71897 -0.31435 -4.09194
C   2.50144 -0.08243 -1.93839
C   1.55637 -0.10242 -0.86524
C   2.00759  0.12630  0.49268
C   1.47843  0.28361  2.74425
H   0.69713  0.24206  3.51151
C   2.82792  0.53291  3.09302
H   3.09852  0.68515  4.14112
C   3.78619  0.57902  2.08674
H   4.83860  0.77049  2.31965
C   3.39470  0.37165  0.73657
C   4.31925  0.39400 -0.36557
H   5.37683  0.58545 -0.15859
C   3.88845  0.17509 -1.65173
H   4.59567  0.18828 -2.48666
C  -1.57185  1.37370 -0.00918
C  -0.92859  2.52389  0.45088
C  -2.64418  1.41207 -0.90526
C  -1.37477  3.77297 -0.03185
C  -3.07562  2.67005 -1.37678
C  -2.44208  3.84558 -0.94159
H  -0.10179  2.47071  1.16387
H  -3.14651  0.49610 -1.23384
H  -0.87662  4.68536  0.31382
H  -3.91350  2.71656 -2.08088
H  -2.78319  4.81894 -1.30843
Cl -2.42228 -0.25484  2.17869
O  -2.29894 -2.27382 -0.80268
O  -0.62581 -2.36155  0.70712
C  -1.50193 -2.92850 -0.07991
C  -1.53188 -4.45233 -0.04555
H  -1.95542 -4.84826 -0.98023
H  -0.52674 -4.86878  0.12160
```

```
H  -2.17385 -4.78025  0.79080
```

11-4$_{Cl}$

```
SCF  =      -1244.31704348
H(0 K)=     -1244.010695
H(298 K)=   -1243.985252
G(298 K)=   -1244.066761
SCF(D3BJ) =       -1244.42777632
SCF(BS2) =        -3133.24895975
SCF(BS2+D3BJ) =   -3133.35969259
Low Freq. = 28.4029cm-1,
33.3739cm-1

Cu -0.56112  0.38467  0.25494
N   1.30764  1.16972  0.16276
N   0.39276 -1.32469 -0.13038
C   1.70784  2.43406  0.33463
H   0.92282  3.16331  0.55440
C   3.06964  2.80443  0.24253
H   3.34887  3.85069  0.38706
C   4.03008  1.83367 -0.02903
H   5.08908  2.09945 -0.10326
C   3.62896  0.48232 -0.20724
C   2.23672  0.20660 -0.10082
C   1.74612 -1.13133 -0.25427
C  -0.09793 -2.56851 -0.24204
H  -1.17869 -2.67735 -0.13007
C   0.73463 -3.68168 -0.49290
H   0.28076 -4.67171 -0.57660
C   2.10695 -3.50182 -0.63184
H   2.76722 -4.35168 -0.82947
C   2.65459 -2.19755 -0.51012
C   4.05923 -1.89532 -0.62339
H   4.75146 -2.71810 -0.82539
C   4.52796 -0.60955 -0.47903
H   5.59770 -0.39703 -0.56468
C  -2.31597 -0.40335  0.02052
C  -2.80419 -0.55571 -1.28045
C  -2.99145 -0.85218  1.15640
C  -4.03026 -1.23608 -1.44387
C  -4.21646 -1.52826  0.96472
C  -4.73093 -1.72027 -0.32741
H  -2.26775 -0.15183 -2.14241
H  -2.57187 -0.69727  2.15538
H  -4.42881 -1.37462 -2.45469
H  -4.75934 -1.89880  1.84111
H  -5.68310 -2.24271 -0.46457
Cl -0.23024  0.19957  2.91192
O  -1.01407  1.96846 -2.00883
O  -1.48255  2.04934  0.21446
C  -1.50112  2.54039 -1.01845
C  -2.21352  3.88614 -1.11051
H  -3.29539  3.70472 -1.23192
H  -2.07154  4.48327 -0.19784
```

```
H  -1.85012  4.43771 -1.98919
```

11-5$_{Cl}$

```
SCF  =      -1244.31351271
H(0 K)=     -1244.007623
H(298 K)=   -1243.982053
G(298 K)=   -1244.064459
SCF(D3BJ) =       -1244.42416674
SCF(BS2) =        -3133.24758114
SCF(BS2+D3BJ) =   -3133.35823517
Low Freq. = 25.2503cm-1,
26.3285cm-1

Cu  0.83620 -0.42435 -0.47349
N  -0.97328 -1.34207  0.72193
N  -0.60146  0.82239 -0.94149
C  -1.15154 -2.40899  1.50892
H  -0.25614 -2.99642  1.73945
C  -2.41350 -2.78147  2.03172
H  -2.49660 -3.66423  2.67088
C  -3.52605 -2.00830  1.72083
H  -4.51694 -2.26416  2.10955
C  -3.37511 -0.87068  0.88305
C  -2.05888 -0.58412  0.40443
C  -1.85704  0.55517 -0.46686
C  -0.39569  1.86394 -1.76221
H   0.62972  2.02603 -2.10317
C  -1.44415  2.71321 -2.16909
H  -1.22580  3.54584 -2.84138
C  -2.73088  2.47155 -1.70140
H  -3.56475  3.11587 -1.99624
C  -2.96984  1.37561 -0.83235
C  -4.27705  1.05986 -0.31930
H  -5.11521  1.70134 -0.60798
C  -4.47311 -0.02122  0.50522
H  -5.47198 -0.25612  0.88590
C   1.46637  0.90308  0.81939
C   2.33482  1.90194  0.37206
C   1.00875  0.82566  2.13591
C   2.74770  2.88364  1.29872
C   1.43574  1.81683  3.04574
C   2.29871  2.84230  2.62812
H   2.71399  1.91480 -0.65254
H   0.33727  0.02840  2.46389
H   3.42951  3.67355  0.96549
H   1.08231  1.77368  4.08164
H   2.62553  3.60668  3.34029
Cl  0.32677 -1.75424 -2.35029
O   3.53957 -0.36991 -1.47767
O   2.30880 -1.45536  0.10110
C   3.41745 -1.22584 -0.58672
C   4.57450 -2.12020 -0.14225
H   5.02580 -1.69747  0.77188
H   4.22752 -3.13731  0.09465
```

H   5.33978 -2.15447 -0.93042

11-1$_{Cl}$_T
SCF =   -1244.28873807
H(0 K)=   -1243.983430
H(298 K)=   -1243.957783
G(298 K)=   -1244.040953
SCF(D3BJ) =   -1244.39768799
SCF(BS2) =   -3133.21864140
SCF(BS2+D3BJ) =   -3133.32759132
Low Freq. = 24.6383cm-1,
27.7886cm-1

Cu	-0.61565	-0.45525	0.28942
N	0.43906	1.31261	-0.09445
N	1.47221	-1.17895	0.22729
C	-0.09644	2.53299	-0.22659
H	-1.18497	2.59208	-0.14117
C	0.69250	3.67954	-0.47082
H	0.20475	4.65168	-0.57484
C	2.07274	3.54683	-0.57259
H	2.70845	4.41781	-0.76036
C	2.66640	2.26534	-0.42577
C	1.79452	1.16078	-0.18259
C	2.34603	-0.16253	-0.01148
C	1.95355	-2.41373	0.40528
H	1.21148	-3.19545	0.59684
C	3.33667	-2.70808	0.35008
H	3.67576	-3.73597	0.50141
C	4.23982	-1.67954	0.10291
H	5.31569	-1.87526	0.05405
C	3.75836	-0.35625	-0.08597
C	4.61139	0.77500	-0.34087
H	5.69203	0.61198	-0.39938
C	4.08577	2.03489	-0.50448
H	4.74074	2.89054	-0.69558
C	-2.55124	0.31768	0.11197
C	-3.45262	0.30118	1.18805
C	-2.95207	0.82544	-1.13615
C	-4.75891	0.80452	1.01610
C	-4.25744	1.33614	-1.30096
C	-5.16083	1.32472	-0.22573
H	-3.14376	-0.09377	2.16133
H	-2.26457	0.82356	-1.98894
H	-5.45864	0.78920	1.86017
H	-4.56220	1.73592	-2.27528
H	-6.17520	1.71717	-0.35561
Cl	-0.42716	-0.25894	2.65819
O	-0.77097	-1.26788	-1.68698
O	-1.31569	-2.42246	0.11092
C	-1.21172	-2.35790	-1.17254
C	-1.62941	-3.51676	-2.03838
H	-1.07175	-3.51107	-2.98640
H	-1.47545	-4.46885	-1.50933

H -2.70474 -3.41976 -2.26776

11-2$_{Cl}$_T
SCF =   -1244.29216999
H(0 K)=   -1243.986882
H(298 K)=   -1243.961176
G(298 K)=   -1244.044385
SCF(D3BJ) =   -1244.40203928
SCF(BS2) =   -3133.22349007
SCF(BS2+D3BJ) =   -3133.33335936
Low Freq. = 25.9296cm-1,
29.5253cm-1

Cu	-0.89592	0.22622	-0.59215
N	0.39164	0.98533	0.87526
N	0.92427	-0.50723	-1.29863
C	0.07018	1.71547	1.94882
H	-0.98645	1.97989	2.04935
C	1.04378	2.12732	2.88814
H	0.73632	2.72683	3.74828
C	2.37476	1.76569	2.69951
H	3.14522	2.07455	3.41273
C	2.73661	0.98699	1.56789
C	1.68849	0.61685	0.67528
C	1.97484	-0.17601	-0.48890
C	1.15281	-1.24581	-2.39302
H	0.27176	-1.47806	-2.99932
C	2.44859	-1.69340	-2.73881
H	2.58182	-2.29157	-3.64344
C	3.52833	-1.36791	-1.92379
H	4.53942	-1.70695	-2.16996
C	3.31312	-0.58703	-0.75701
C	4.35626	-0.19540	0.15628
H	5.38116	-0.51322	-0.05788
C	4.07986	0.55874	1.27341
H	4.88182	0.84780	1.95946
C	-1.45971	-1.36433	0.67228
C	-2.52076	-1.11710	1.55266
C	-0.79600	-2.59771	0.66256
C	-2.91880	-2.12893	2.45256
C	-1.19955	-3.60367	1.56690
C	-2.25841	-3.36904	2.45982
H	-3.03827	-0.15188	1.54889
H	0.02602	-2.79866	-0.03304
H	-3.74706	-1.93929	3.14533
H	-0.68137	-4.56971	1.56469
H	-2.56944	-4.15229	3.15928
Cl	-2.15201	-0.58841	-2.41909
O	-0.89345	2.41067	-1.50326
O	-2.33815	1.55198	-0.03760
C	-1.92679	2.53813	-0.78848
C	-2.75577	3.80735	-0.77788
H	-3.66205	3.65116	-1.38790
H	-3.08115	4.05220	0.24502

H -2.18145 4.64256 -1.20337

11-4$_{Cl}$_T
SCF = -1244.28873807
H(0 K)= -1243.983430
H(298 K)= -1243.957783
G(298 K)= -1244.040953
SCF(D3BJ) = -1244.39768800
SCF(BS2) = -3133.21864143
SCF(BS2+D3BJ) = -3133.32759136
Low Freq. = 24.6408cm-1, 27.7895cm-1

Cu 0.61565 -0.45525 0.28942
N -1.47222 -1.17895 0.22728
N -0.43905 1.31260 -0.09446
C -1.95357 -2.41372 0.40528
H -1.21151 -3.19545 0.59683
C -3.33670 -2.70806 0.35009
H -3.67579 -3.73594 0.50142
C -4.23984 -1.67951 0.10292
H -5.31570 -1.87523 0.05406
C -3.75837 -0.35623 -0.08597
C -2.34603 -0.16252 -0.01148
C -1.79451 1.16079 -0.18260
C 0.09646 2.53297 -0.22660
H 1.18500 2.59205 -0.14119
C -0.69247 3.67954 -0.47084
H -0.20470 4.65166 -0.57487
C -2.07271 3.54683 -0.57261
H -2.70841 4.41782 -0.76038
C -2.66638 2.26535 -0.42578
C -4.08575 2.03491 -0.50449
H -4.74072 2.89057 -0.69559
C -4.61138 0.77503 -0.34087
H -5.69202 0.61202 -0.39938
C 2.55123 0.31769 0.11198
C 2.95211 0.82535 -1.13616
C 3.45257 0.30128 1.18809
C 4.25749 1.33604 -1.30097
C 4.75888 0.80460 1.01615
C 5.16084 1.32469 -0.22571
H 2.26463 0.82342 -1.98898
H 3.14368 -0.09359 2.16139
H 4.56228 1.73574 -2.27531
H 5.45858 0.78935 1.86024
H 6.17521 1.71713 -0.35559
Cl 0.42713 -0.25898 2.65819
O 1.31569 -2.42246 0.11092
O 0.77097 -1.26788 -1.68698
C 1.21172 -2.35790 -1.17254
C 1.62941 -3.51676 -2.03837
H 1.47539 -4.46886 -1.50935
H 1.07180 -3.51103 -2.98643

H 2.70476 -3.41980 -2.26770

11-5$_{Cl}$_T
SCF = -1244.28658407
H(0 K)= -1243.981431
H(298 K)= -1243.955594
G(298 K)= -1244.040535
SCF(D3BJ) = -1244.39618390
SCF(BS2) = -3133.21498105
SCF(BS2+D3BJ) = -3133.32458086
Low Freq. = 11.4510cm-1, 28.0219cm-1

Cu 0.83738 -0.68547 0.02238
N -0.65370 -0.26617 1.36964
N -0.69749 -0.43802 -1.31894
C -0.58769 -0.21192 2.70497
H 0.37871 -0.45067 3.15840
C -1.71139 0.12863 3.49308
H -1.61005 0.16103 4.58037
C -2.92389 0.41350 2.87167
H -3.80584 0.67991 3.46218
C -3.01821 0.35238 1.45557
C -1.83527 0.00351 0.74196
C -1.85902 -0.09004 -0.69219
C -0.67812 -0.56197 -2.65125
H 0.27355 -0.85689 -3.10282
C -1.83032 -0.33116 -3.43806
H -1.76641 -0.44409 -4.52282
C -3.02223 0.03340 -2.81855
H -3.92545 0.21810 -3.40815
C -3.06713 0.16185 -1.40454
C -4.24775 0.52050 -0.66091
H -5.17150 0.71622 -1.21372
C -4.22427 0.61255 0.71177
H -5.12924 0.88240 1.26449
C 1.38893 1.39221 -0.10353
C 1.49539 2.01585 -1.35344
C 1.69896 2.08570 1.07381
C 1.91491 3.36143 -1.42451
C 2.11700 3.43130 0.99740
C 2.22412 4.06775 -0.25035
H 1.26167 1.47969 -2.27985
H 1.62738 1.60216 2.05420
H 1.99833 3.84983 -2.40236
H 2.35883 3.97472 1.91821
H 2.54890 5.11207 -0.30765
Cl 0.17061 -3.17792 0.15577
O 2.53780 -1.18862 -1.05936
O 2.53927 -1.06953 1.13826
C 3.17869 -1.31983 0.04892
C 4.60947 -1.78080 0.07502
H 5.11352 -1.52787 -0.86908
H 5.14178 -1.33021 0.92589

H   4.62996 -2.87738 0.19856

TS(11-12)
SCF =   -1240.74130609
H(0 K)=   -1240.437718
H(298 K)=   -1240.411796
G(298 K)=   -1240.497950
SCF(D3BJ) =   -1240.85706207
SCF(BS2) =   -2970.83755743
SCF(BS2+D3BJ) =   -
2970.95331512
Low Freq. = -47.2616cm-1,
17.8134cm-1

Cu 0.57173 -0.10527 -0.49760
N -1.20359 -1.21282 -0.14941
N -0.96078 1.48604 -0.49094
C -1.30340 -2.54191 -0.01495
H -0.36017 -3.09389 -0.07682
C -2.54006 -3.19933 0.17996
H -2.56326 -4.28691 0.28757
C -3.70704 -2.44412 0.23094
H -4.68064 -2.92149 0.38264
C -3.63539 -1.03371 0.07747
C -2.34109 -0.45880 -0.11516
C -2.21249 0.97483 -0.29399
C -0.83492 2.80807 -0.66913
H   0.18541 3.17687 -0.82391
C -1.93374 3.69820 -0.65853
H -1.76559 4.76768 -0.80989
C -3.21184 3.18936 -0.45524
H -4.08566 3.84879 -0.44060
C -3.38524 1.79225 -0.26723
C -4.67248 1.18106 -0.06044
H -5.55800 1.82449 -0.04135
C -4.79308 -0.17814 0.10421
H -5.77619 -0.63502 0.25637
C   1.39122 -0.13733 1.43696
C   0.59518 0.49537 2.42049
C   1.79320 -1.48563 1.57760
C   0.12763 -0.26285 3.50334
C   1.30641 -2.22522 2.67042
C   0.48041 -1.62085 3.63284
H   0.32953 1.55139 2.32466
H   2.42672 -1.95384 0.82027
H -0.50946 0.21549 4.25480
H   1.59117 -3.27810 2.76958
H   0.12313 -2.19927 4.49043
I   2.78590 1.21640 0.22586
O   1.73716 -2.40343 -1.65643
O   0.90346 -0.45365 -2.46683
C   1.43426 -1.63412 -2.60598
C   1.66294 -2.07506 -4.05789
H   2.29758 -2.97223 -4.10426
H   0.69203 -2.30537 -4.53079
H   2.12339 -1.26332 -4.64429

Int(11-12)
SCF =   -1240.74790203
H(0 K)=   -1240.443633
H(298 K)=   -1240.417426
G(298 K)=   -1240.503137
SCF(D3BJ) =   -1240.86104265
SCF(BS2) =   -2970.84389387
SCF(BS2+D3BJ) =   -
2970.95706384
Low Freq. = 15.8177cm-1,
19.1411cm-1

Cu -0.19464 -0.42282 0.23856
N   1.75773 -1.23602 -0.01648
N   0.92423 1.34462 0.18660
C   2.15498 -2.51317 -0.11912
H   1.35903 -3.26415 -0.14080
C   3.51333 -2.89425 -0.20178
H   3.77265 -3.95299 -0.28263
C   4.49593 -1.90985 -0.17852
H   5.55713 -2.17123 -0.24091
C   4.11379 -0.54640 -0.07275
C   2.71599 -0.26000 0.00590
C   2.27232 1.11320 0.11530
C   0.50276 2.61433 0.29988
H -0.58177 2.75696 0.35535
C   1.38358 3.71815 0.33951
H   0.97627 4.72839 0.42975
C   2.75435 3.49571 0.26100
H   3.46372 4.32910 0.28757
C   3.23806 2.16578 0.14664
C   4.63873 1.84201 0.06616
H   5.36466 2.66083 0.09347
C   5.06013 0.53812 -0.04023
H   6.12732 0.30164 -0.10000
C -1.72901 -0.29982 -1.23491
C -1.18355 0.05120 -2.50199
C -1.69581 -1.64771 -0.76702
C -0.59642 -0.94277 -3.29087
C -1.09905 -2.63053 -1.60812
C -0.56422 -2.28702 -2.85137
H -1.23595 1.08391 -2.85713
H -2.19998 -1.93741 0.16747
H -0.17427 -0.67123 -4.26373
H -1.09620 -3.67300 -1.27202
H -0.13035 -3.05906 -3.49446
I -3.18052 1.08647 -0.36908
O -2.28740 -2.13870 2.23279
O -0.58888 -0.60704 2.22225
C -1.48568 -1.35328 2.79908
C -1.52173 -1.21256 4.33141

H -2.26155 -1.89552 4.77442
H -0.52651 -1.42319 4.75818
H -1.77646 -0.17495 4.60807

12

SCF = -997.650271021
H(0 K)= -997.433417
H(298 K)= -997.415226
G(298 K)= -997.481023
SCF(D3BJ) = -997.716916964
SCF(BS2) = -2441.28069941
SCF(BS2+D3BJ) = -2441.34735106
Low Freq. = 25.2583cm-1, 34.8748cm-1

C -3.96420 0.19895 -0.00752
C -2.98804 -0.86093 -0.00617
C -1.60108 -0.52819 -0.00032
C -1.19865 0.86063 0.00374
C -2.18901 1.88737 0.00363
C -3.58127 1.51993 -0.00245
N -0.60730 -1.47626 0.00149
C -0.96973 -2.77378 -0.00255
C -2.31792 -3.19291 -0.00859
C -3.33079 -2.23920 -0.01026
C -1.72431 3.23082 0.00901
C -0.35551 3.48195 0.01245
C 0.54980 2.39309 0.00984
N 0.14036 1.11735 0.00627
Cu 1.23774 -0.75757 0.00946
O 3.10763 -1.03328 0.02801
C 3.91595 0.00468 -0.01578
C 5.39816 -0.38525 0.01406
O 3.57246 1.20382 -0.07589
H -0.15524 -3.50322 -0.00088
H -2.54514 -4.26183 -0.01157
H -4.38429 -2.53611 -0.01461
H 1.63780 2.52712 0.00789
H 0.02987 4.50515 0.01606
H -2.44780 4.05258 0.00979
H -4.33307 2.31570 -0.00324
H -5.02479 -0.07183 -0.01262
H 5.63266 -1.05181 -0.83298
H 6.03957 0.50624 -0.03437
H 5.62332 -0.94735 0.93622

TS(11-1$_I$-12)

SCF = -1240.73911388
H(0 K)= -1240.435420
H(298 K)= -1240.409451
G(298 K)= -1240.496111
SCF(D3BJ) = -1240.85290588
SCF(BS2) = -2970.83571531
SCF(BS2+D3BJ) = -2970.94955682
Low Freq. = -96.5286cm-1, 12.2026cm-1

Cu 0.35363 -0.19241 0.42930
N -1.04427 1.39685 0.58691
N -1.46131 -1.23623 0.05400
C -0.84316 2.68509 0.90908
H 0.19763 2.98082 1.07564
C -1.88881 3.62766 1.02624
H -1.65373 4.66180 1.29104
C -3.19853 3.21988 0.79636
H -4.03160 3.92629 0.87064
C -3.45538 1.86272 0.46782
C -2.33448 0.98026 0.38278
C -2.55481 -0.42016 0.08791
C -1.64021 -2.54409 -0.18265
H -0.72929 -3.15201 -0.16138
C -2.91436 -3.10866 -0.42225
H -3.00246 -4.18086 -0.61678
C -4.03678 -2.28602 -0.40111
H -5.03713 -2.69290 -0.58088
C -3.88247 -0.89993 -0.13184
C -4.98592 0.02206 -0.06248
H -5.99734 -0.35837 -0.23823
C -4.77977 1.34959 0.22845
H -5.62459 2.04314 0.28887
C 2.33030 0.67270 -0.49947
C 2.28073 2.08075 -0.41375
C 3.34798 -0.06658 0.14101
C 3.20819 2.74178 0.41042
C 4.26431 0.61729 0.95434
C 4.19807 2.01594 1.09507
H 1.53597 2.64789 -0.97762
H 3.38410 -1.15428 0.04522
H 3.16260 3.83250 0.49570
H 5.04027 0.04733 1.47593
H 4.92683 2.53925 1.72148
I 1.27850 -0.27617 -2.21450
O 1.10331 -0.89020 2.15898
O 1.49477 -2.82662 1.03061
C 1.50004 -2.12715 2.07660
C 2.04695 -2.71606 3.38536
H 1.44433 -2.39202 4.24901
H 2.07586 -3.81507 3.34100
H 3.07585 -2.34904 3.54884

TS1(11-4$_I$-12)

SCF = -1240.74471329
H(0 K)= -1240.439933
H(298 K)= -1240.414613

G(298 K)=   -1240.497648
SCF(D3BJ) =   -1240.85897980
SCF(BS2) =   -2970.83737942
SCF(BS2+D3BJ) =   -2970.95172900
Low Freq. = -26.2937cm-1, 21.4350cm-1

Cu	0.60270	0.31357	-0.24706
N	-1.23750	0.72445	-1.01837
N	-0.91164	-0.51443	1.39300
C	-1.36578	1.35569	-2.19493
H	-0.43647	1.62380	-2.70488
C	-2.62360	1.66408	-2.75498
H	-2.67167	2.17609	-3.71890
C	-3.77663	1.30466	-2.06535
H	-4.76773	1.52584	-2.47389
C	-3.66858	0.64369	-0.81403
C	-2.35526	0.37322	-0.31823
C	-2.18549	-0.29068	0.95531
C	-0.75962	-1.15296	2.55889
H	0.26842	-1.33638	2.88731
C	-1.84033	-1.59038	3.36061
H	-1.63919	-2.10388	4.30426
C	-3.13889	-1.35165	2.92837
H	-4.00106	-1.67033	3.52289
C	-3.34619	-0.68609	1.69160
C	-4.65582	-0.40275	1.16559
H	-5.52925	-0.71140	1.74861
C	-4.81162	0.24011	-0.03841
H	-5.80992	0.45465	-0.43207
C	2.46595	0.04986	0.24724
C	2.77617	-0.34274	1.55135
C	3.46050	0.39713	-0.67629
C	4.13161	-0.37767	1.94614
C	4.80811	0.35410	-0.26937
C	5.14461	-0.03389	1.03907
H	2.00033	-0.60386	2.27358
H	3.20744	0.69741	-1.69701
H	4.37816	-0.67637	2.97080
H	5.59042	0.62411	-0.98713
H	6.19400	-0.06895	1.34890
I	0.78686	-2.02241	-1.39082
O	0.54910	2.48870	1.86585
O	1.03361	2.20702	-0.34559
C	0.89524	2.92174	0.75480
C	1.24354	4.39923	0.53480
H	2.33867	4.50646	0.45446
H	0.80391	4.77603	-0.40168
H	0.88684	4.99681	1.38561

Int(11-4_I-12)
SCF =   -1240.74734389
H(0 K)=   -1240.441743
H(298 K)=   -1240.415879

G(298 K)=   -1240.500431
SCF(D3BJ) =   -1240.86296754
SCF(BS2) =   -2970.83811243
SCF(BS2+D3BJ) =   -2970.95378004
Low Freq. = 17.6523cm-1, 28.0702cm-1

Cu	-0.46439	-0.18433	0.25611
N	1.37698	-1.01615	0.22136
N	0.70961	1.56872	-0.09316
C	1.66268	-2.31016	0.41905
H	0.81349	-2.96230	0.63813
C	2.98424	-2.80389	0.35079
H	3.16596	-3.86820	0.51773
C	4.02955	-1.92808	0.07123
H	5.06131	-2.28791	0.01017
C	3.75338	-0.54979	-0.12736
C	2.39023	-0.14035	-0.03975
C	2.03544	1.24313	-0.19901
C	0.36673	2.86138	-0.16631
H	-0.69813	3.08691	-0.05719
C	1.31085	3.89206	-0.37951
H	0.96636	4.92773	-0.43093
C	2.65575	3.56969	-0.52219
H	3.40616	4.34698	-0.69682
C	3.05771	2.21140	-0.42635
C	4.42558	1.77166	-0.53184
H	5.19947	2.52224	-0.71923
C	4.76041	0.44530	-0.39036
H	5.80408	0.12480	-0.46372
C	-2.23260	0.64389	0.17502
C	-2.48561	1.58439	-0.83723
C	-3.17959	0.37279	1.16991
C	-3.68241	2.32873	-0.79572
C	-4.37377	1.12359	1.19403
C	-4.62445	2.10255	0.22015
H	-1.78542	1.73552	-1.66278
H	-2.99156	-0.39567	1.92241
H	-3.87550	3.06662	-1.58195
H	-5.10360	0.93057	1.98804
H	-5.55753	2.67466	0.24185
I	-1.29617	-1.60639	-1.88140
O	0.22492	0.36744	3.03353
O	-0.91159	-1.24501	1.90091
C	-0.39628	-0.71332	2.98635
C	-0.65202	-1.54219	4.25045
H	-1.72101	-1.48252	4.51747
H	-0.41578	-2.60389	4.07682
H	-0.05174	-1.15549	5.08611

TS2(11-4_I-12)
SCF =   -1240.73989376
H(0 K)=   -1240.435882
H(298 K)=   -1240.410166

G(298 K)= -1240.494641
SCF(D3BJ) = -1240.85313548
SCF(BS2) = -2970.83642261
SCF(BS2+D3BJ) = -2970.94966433
Low Freq. = -68.3364cm-1, 16.9263cm-1

Cu	-0.35454	-0.18641	0.24391
N	1.42955	-1.28774	-0.14776
N	0.98300	1.38018	-0.08604
C	1.62216	-2.61347	-0.18336
H	0.72537	-3.23371	-0.07508
C	2.89747	-3.19763	-0.35698
H	2.99385	-4.28612	-0.37939
C	4.00899	-2.37161	-0.49627
H	5.01030	-2.79328	-0.63030
C	3.84010	-0.96205	-0.46377
C	2.51221	-0.46743	-0.28725
C	2.27460	0.95742	-0.24796
C	0.74969	2.70059	-0.02820
H	-0.29550	2.99897	0.09538
C	1.77509	3.66682	-0.13032
H	1.52032	4.72811	-0.07257
C	3.09049	3.24864	-0.30538
H	3.90611	3.97363	-0.39175
C	3.37489	1.85922	-0.37055
C	4.70254	1.32930	-0.54740
H	5.53525	2.03344	-0.64200
C	4.92671	-0.02641	-0.59413
H	5.94001	-0.41829	-0.72749
C	-2.19774	0.62065	-0.50335
C	-2.01968	1.52045	-1.57764
C	-3.00715	0.95631	0.60429
C	-2.59222	2.80198	-1.49265
C	-3.56441	2.24357	0.66806
C	-3.36689	3.16544	-0.37735
H	-1.43932	1.23063	-2.45754
H	-3.14808	0.23765	1.41459
H	-2.43957	3.50834	-2.31531
H	-4.16676	2.51911	1.54019
H	-3.82434	4.15818	-0.32697
I	-2.14509	-1.62310	-1.16181
O	0.46541	0.38724	3.19144
O	-1.03786	-0.87742	2.03436
C	-0.47128	-0.44372	3.12661
C	-1.05745	-1.04497	4.41551
H	-2.13455	-0.81579	4.48462
H	-0.96421	-2.14428	4.39977
H	-0.54453	-0.65037	5.30497

6_I
SCF = -1561.33182231
H(0 K)= -1560.995101
H(298 K)= -1560.964451
G(298 K)= -1561.063730
SCF(D3BJ) = -1561.48024312
SCF(BS2) = -5021.16755210
SCF(BS2+D3BJ) = -5021.31597292
Low Freq. = 4.8449cm-1, 12.3519cm-1

I	2.32202	-0.00000	-0.41196
Cu	0.00000	1.29273	-0.12826
I	-2.32202	0.00000	-0.41196
Cu	-0.00000	-1.29273	-0.12826
N	0.00000	-2.71481	1.42393
N	0.00000	-3.06316	-1.27795
C	0.00000	-3.22263	-2.61092
H	0.00000	-2.30373	-3.20622
H	0.00000	-4.55245	-4.32774
C	0.00000	-4.48970	-3.23643
C	0.00000	-5.63502	-2.44670
H	0.00000	-6.63224	-2.89841
C	0.00000	-5.50618	-1.03261
C	0.00000	-6.63576	-0.13987
C	0.00000	-4.18355	-0.49150
C	0.00000	-3.99851	0.94511
H	0.00000	-7.64153	-0.57206
C	0.00000	-6.46052	1.22321
C	0.00000	-5.14210	1.80212
C	0.00000	-4.91146	3.20315
H	0.00000	-5.76276	3.89140
H	0.00000	-7.32439	1.89563
C	0.00000	-3.60428	3.67889
H	0.00000	-3.38987	4.75076
C	0.00000	-2.53543	2.75499
H	0.00000	-1.49727	3.10106
N	0.00000	2.71481	1.42393
N	0.00000	3.06316	-1.27795
C	0.00000	3.22263	-2.61092
H	0.00000	2.30373	-3.20622
C	0.00000	4.48970	-3.23643
H	0.00000	4.55245	-4.32774
C	0.00000	5.63502	-2.44670
H	0.00000	6.63224	-2.89841
C	0.00000	5.50618	-1.03261
C	0.00000	3.99851	0.94511
C	0.00000	5.14210	1.80212
C	0.00000	6.46052	1.22321
H	0.00000	7.32439	1.89563
C	0.00000	6.63576	-0.13987
H	0.00000	7.64153	-0.57206
C	0.00000	4.91146	3.20315
H	0.00000	5.76276	3.89140
C	0.00000	3.60428	3.67889
H	0.00000	3.38987	4.75076
C	0.00000	2.53543	2.75499
H	0.00000	1.49727	3.10106

C   0.00000 4.18355 -0.49150

TS(11-3$_{Cl}$-12)
SCF =   -1244.30078282
H(0 K)=   -1243.996685
H(298 K)=   -1243.971140
G(298 K)=   -1244.055198
SCF(D3BJ) =   -1244.41015681
SCF(BS2) =   -3133.24099122
SCF(BS2+D3BJ) =   -3133.35036522
Low Freq. = -155.6125cm-1, 13.2903cm-1

Cu	0.97781	-0.55775	-0.29683
N	-0.28279	0.16613	1.17929
N	-1.06140	-0.84922	-1.23863
C	0.12135	0.63282	2.36852
H	1.20272	0.61680	2.53712
C	-0.78398	1.09811	3.34894
H	-0.40203	1.47160	4.30248
C	-2.14797	1.07009	3.07812
H	-2.87529	1.42501	3.81537
C	-2.60532	0.56861	1.83034
C	-1.61845	0.11883	0.89964
C	-2.03257	-0.41671	-0.38354
C	-1.43383	-1.35273	-2.42192
H	-0.62462	-1.68996	-3.07930
C	-2.78549	-1.45535	-2.82775
H	-3.02714	-1.87660	-3.80713
C	-3.78314	-1.01613	-1.96442
H	-4.83953	-1.08043	-2.24478
C	-3.42574	-0.47866	-0.69893
C	-4.39312	-0.00808	0.25795
H	-5.45477	-0.06229	-0.00356
C	-3.99857	0.49432	1.47487
H	-4.74046	0.84649	2.19866
C	1.63441	1.24151	-1.03063
C	0.70227	2.03440	-1.72584
C	2.54198	1.78101	-0.09967
C	0.60047	3.39033	-1.37543
C	2.41394	3.14172	0.23265
C	1.44988	3.94675	-0.39934
H	0.05575	1.59835	-2.49107
H	3.28610	1.14156	0.38183
H	-0.14479	4.01467	-1.87963
H	3.08670	3.57000	0.98328
H	1.37159	5.00858	-0.14725
Cl	2.39368	-0.36429	-2.12502
O	2.77508	-1.20520	1.55332
O	1.30488	-2.45933	0.37817
C	2.24682	-2.31482	1.26183
C	2.70066	-3.59806	1.96347
H	3.53185	-3.40130	2.65593
H	1.85810	-4.03744	2.52447
H	3.01482	-4.34710	1.21729

TS(11-1$_{Cl}$-12)
SCF =   -1244.30020523
H(0 K)=   -1243.995594
H(298 K)=   -1243.970312
G(298 K)=   -1244.052334
SCF(D3BJ) =   -1244.40776106
SCF(BS2) =   -3133.24032015
SCF(BS2+D3BJ) =   -3133.34787598
Low Freq. = -165.1242cm-1, 22.6597cm-1

Cu	-0.62325	-0.27526	0.18435
N	0.76159	1.39827	-0.31783
N	1.26601	-1.19573	0.34031
C	0.50911	2.65251	-0.71561
H	-0.54773	2.92793	-0.79794
C	1.52357	3.59179	-1.01321
H	1.24869	4.60068	-1.33145
C	2.85437	3.21080	-0.88628
H	3.66555	3.91509	-1.09714
C	3.16350	1.88397	-0.48515
C	2.06769	1.00485	-0.22113
C	2.33559	-0.37037	0.14642
C	1.48626	-2.48130	0.65163
H	0.58773	-3.09757	0.75781
C	2.78661	-3.01501	0.80479
H	2.90992	-4.06991	1.06309
C	3.88684	-2.18554	0.61475
H	4.90649	-2.56977	0.72079
C	3.68511	-0.82315	0.26655
C	4.76365	0.09836	0.02139
H	5.79220	-0.26239	0.12266
C	4.51212	1.39913	-0.34452
H	5.33736	2.09085	-0.54131
C	-2.42698	0.52097	0.80934
C	-2.49310	1.92735	0.80041
C	-3.52006	-0.28205	0.42725
C	-3.63448	2.53808	0.24953
C	-4.64674	0.35434	-0.11578
C	-4.70911	1.75848	-0.21074
H	-1.67935	2.52840	1.21291
H	-3.45435	-1.37088	0.49216
H	-3.68350	3.63124	0.20764
H	-5.48595	-0.25908	-0.46070
H	-5.60187	2.24124	-0.61934
Cl	-1.30475	-0.20880	2.40879
O	-1.20507	-0.98965	-1.61599
O	-1.58893	-2.86936	-0.40081
C	-1.57461	-2.23169	-1.48662
C	-2.06353	-2.90429	-2.77472
H	-1.55027	-2.50063	-3.66110
H	-1.92205	-3.99434	-2.72136

H -3.14381 -2.70775 -2.89519

TS(11-4$_{C1}$-12)
SCF =   -1244.29865614
H(0 K)=   -1243.993981
H(298 K)=   -1243.968731
G(298 K)=   -1244.050756
SCF(D3BJ) =   -1244.40614898
SCF(BS2) =   -3133.23893243
SCF(BS2+D3BJ) =   -3133.34642527
Low Freq. = -172.4568cm-1,
20.2299cm-1

Cu -0.57487 -0.48364 -0.30194
N   1.41166 -1.12805 -0.58279
N   0.44401 1.28062 0.15318
C   1.85473 -2.33449 -0.95975
H   1.08785 -3.08406 -1.17956
C   3.23117 -2.63839 -1.07057
H   3.53845 -3.63876 -1.38569
C   4.17094 -1.65641 -0.77093
H   5.24323 -1.86478 -0.84246
C   3.73165 -0.36872 -0.36308
C   2.32254 -0.15579 -0.29121
C   1.80480 1.12800 0.11653
C -0.05031 2.45685 0.56574
H -1.14134 2.54183 0.57698
C   0.76881 3.54178 0.95052
H   0.30272 4.47386 1.27952
C   2.15192 3.40493 0.89754
H   2.81177 4.23079 1.18154
C   2.71135 2.17166 0.47119
C   4.12891 1.92884 0.38719
H   4.81151 2.74077 0.65680
C   4.62045 0.70977 -0.01692
H   5.69964 0.53736 -0.07518
C -2.46246 0.18756 -0.78282
C -2.53866 1.49624 -1.29528
C -3.47925 -0.36169 0.01945
C -3.60126 2.31757 -0.87377
C -4.52731 0.48061 0.42612
C -4.59438 1.81564 -0.01575
H -1.78693 1.86971 -1.99541
H -3.41074 -1.39745 0.35822
H -3.65314 3.34737 -1.24262
H -5.30092 0.07888 1.08927
H -5.42832 2.45402 0.29106
Cl -1.67297 -1.14262 -2.29497
O -0.47738 -0.91841 2.65936
O -1.18287 -2.06988 0.82708
C -0.97173 -1.92994 2.10740
C -1.40525 -3.13840 2.95169
H -2.49772 -3.27491 2.87473
H -0.93797 -4.06200 2.57124
H -1.13406 -2.99798 4.00821

6$_{C1}$
SCF =   -1568.45615626
H(0 K)=   -1568.118997
H(298 K)=   -1568.088839
G(298 K)=   -1568.184850
SCF(D3BJ) =   -1568.59013034
SCF(BS2) =   -5345.98085499
SCF(BS2+D3BJ) =   -5346.11482907
Low Freq. = 12.0335cm-1,
12.4169cm-1

Cl -1.82210 -0.00674 -0.63367
Cu 0.00681 -1.48793 -0.28676
Cl 1.82210 0.00674 -0.63367
Cu -0.00681 1.48793 -0.28676
N -0.02593 2.81436 1.28587
N   0.00940 3.26318 -1.39381
C   0.02908 3.47224 -2.72123
H   0.03101 2.57610 -3.35027
H   0.06155 4.86312 -4.38769
C   0.04612 4.76112 -3.29939
C   0.04216 5.87819 -2.46922
H   0.05441 6.89079 -2.88495
C   0.02161 5.69812 -1.06091
C   0.01522 6.79313 -0.12601
C   0.00624 4.35735 -0.56829
C -0.01436 4.11885 0.85863
H   0.02745 7.81460 -0.51963
C -0.00624 6.56665 1.22951
C -0.02197 5.22755 1.75919
C -0.04458 4.94360 3.15018
H -0.05118 5.76719 3.87127
H -0.01152 7.40483 1.93366
C -0.05828 3.61800 3.57386
H -0.07609 3.36226 4.63654
C -0.04812 2.58488 2.61145
H -0.05822 1.53458 2.91825
N   0.02593 -2.81436 1.28587
N -0.00940 -3.26318 -1.39381
C -0.02908 -3.47224 -2.72123
H -0.03101 -2.57610 -3.35027
C -0.04612 -4.76112 -3.29939
H -0.06155 -4.86312 -4.38769
C -0.04216 -5.87819 -2.46922
H -0.05441 -6.89079 -2.88495
C -0.02161 -5.69812 -1.06091
C   0.01436 -4.11885 0.85863
C   0.02197 -5.22755 1.75919
C   0.00624 -6.56665 1.22951
H   0.01152 -7.40483 1.93366
C -0.01522 -6.79313 -0.12601
H -0.02745 -7.81460 -0.51963

C   0.04458 -4.94360 3.15018
H   0.05118 -5.76719 3.87127
C   0.05828 -3.61800 3.57386
H   0.07609 -3.36226 4.63654
C   0.04812 -2.58488 2.61145
H   0.05822 -1.53458 2.91825
C -0.00624 -4.35735 -0.56829

TS1(CuIII-2^{+}-10^{+})
SCF =   -1583.72461611
H(0 K)=   -1583.300208
H(298 K)=   -1583.269902
G(298 K)=   -1583.361636
SCF(D3BJ) =   -1583.90265001
SCF(BS2) =   -3313.90961070
SCF(BS2+D3BJ) =   -3314.08764460
Low Freq. = -48.3129cm-1,
21.9243cm-1

Cu 0.00993 0.39258 -0.01291
N   1.89577 0.23996 1.18098
N   0.99941 -1.03801 -1.05436
N -1.00057 -1.27031 1.13941
N -1.89495 0.09955 -1.05354
C   2.36763 0.95315 2.21129
H   1.69571 1.71099 2.62542
C   3.65666 0.75445 2.75790
H   3.98122 1.36765 3.60205
C   4.47917 -0.22573 2.21568
H   5.47679 -0.41454 2.62423
C   4.02080 -0.98419 1.10618
C   2.71193 -0.69722 0.61037
C   2.22492 -1.39207 -0.55392
C   0.54157 -1.65587 -2.15381
H -0.43324 -1.33301 -2.52451
C   1.27092 -2.66597 -2.81651
H   0.84412 -3.13398 -3.70648
C   2.51807 -3.03634 -2.32799
H   3.11020 -3.81071 -2.82518
C   3.03466 -2.39227 -1.17374
C   4.33127 -2.68817 -0.62344
H   4.93606 -3.46131 -1.10684
C   4.80931 -2.00527 0.46872
H   5.80346 -2.22285 0.87081
C -0.52485 -1.96367 2.17963
H   0.47185 -1.68549 2.53793
C -1.25593 -3.00030 2.80450
H -0.81809 -3.53342 3.65190
C -2.52384 -3.31330 2.32755
H -3.12026 -4.10435 2.79281
C -3.05094 -2.60011 1.21828
C -2.23018 -1.57787 0.64804
C -2.70902 -0.84237 -0.50496
C -2.30434 0.78138 -2.13019
H -1.59790 1.52102 -2.52698
C -3.56448 0.56021 -2.73018
H -3.85067 1.14053 -3.61061
C -4.41331 -0.39690 -2.18450
H -5.39667 -0.59094 -2.62417
C -3.99937 -1.13675 -1.04507
C -4.80691 -2.15736 -0.43089
H -5.79429 -2.36481 -0.85452
C -4.34917 -2.86356 0.65575
H -4.96630 -3.64349 1.11230
C -0.89726 1.76028 1.07580
C -0.85078 1.54662 2.45897
C -1.61619 2.82933 0.52619
C -1.51982 2.45028 3.31419
C -2.28274 3.71781 1.39416
C -2.23203 3.53452 2.78477
H -0.33223 0.69232 2.89798
H -1.65567 3.00120 -0.55041
H -1.48339 2.27648 4.39493
H -2.83281 4.56084 0.96232
H -2.75056 4.23078 3.45140
I   1.19646 2.34788 -1.60007

Int(CuIII-2^{+}-10^{+})
SCF =   -1583.72757905
H(0 K)=   -1583.303487
H(298 K)=   -1583.272070
G(298 K)=   -1583.367643
SCF(D3BJ) =   -1583.90364126
SCF(BS2) =   -3313.91431735
SCF(BS2+D3BJ) =   -3314.09037954
Low Freq. = 14.8308cm-1,
23.2869cm-1

Cu 0.01782 0.40557 -0.14170
N   2.18240 0.47418 1.11634
N   1.07727 -1.30913 -0.69640
N -0.97255 -0.51572 1.44923
N -2.09608 -0.53328 -1.08268
C   2.72325 1.31998 2.00401
H   2.06939 2.11636 2.37530
C   4.05255 1.22284 2.47605
H   4.42630 1.95278 3.19848
C   4.85066 0.18780 2.00891
H   5.88494 0.07233 2.34839
C   4.31181 -0.74348 1.08259
C   2.95536 -0.55386 0.65995
C   2.37021 -1.49320 -0.27697
C   0.54572 -2.19585 -1.55403
H -0.48040 -1.99798 -1.86630
C   1.24784 -3.31447 -2.04762
H   0.75012 -3.99690 -2.74055
C   2.56130 -3.51216 -1.64607
H   3.14406 -4.36227 -2.01429

```
C   3.15887 -2.59477 -0.74393
C   4.51767 -2.74682 -0.29742
H   5.09560 -3.59591 -0.67518
C   5.07622 -1.85193  0.57967
H   6.11067 -1.96951  0.91715
C  -0.43852 -0.50079  2.67942
H   0.51473  0.02150  2.77952
C  -1.04818 -1.12301  3.78904
H  -0.55971 -1.07643  4.76513
C  -2.25868 -1.77848  3.60966
H  -2.76327 -2.27108  4.44672
C  -2.85423 -1.80676  2.32191
C  -2.16816 -1.15403  1.24707
C  -2.76417 -1.16161 -0.07662
C  -2.65448 -0.50210 -2.29907
H  -2.08229  0.02150 -3.07446
C  -3.90095 -1.10239 -2.59619
H  -4.30515 -1.04520 -3.61011
C  -4.58437 -1.76080 -1.58106
H  -5.54894 -2.24250 -1.77065
C  -4.02721 -1.80598 -0.27476
C  -4.68088 -2.45849  0.82683
H  -5.64209 -2.95103  0.64958
C  -4.11262 -2.46003  2.07659
H  -4.61055 -2.95497  2.91607
C  -0.73689  2.18857  0.28939
C   0.10109  3.16383  0.84848
C  -2.12848  2.34896  0.26340
C  -0.47960  4.26940  1.50270
C  -2.69278  3.46274  0.91758
C  -1.87438  4.41731  1.54287
H   1.18632  3.09624  0.75987
H  -2.77678  1.62359 -0.22963
H   0.17536  5.02423  1.95060
H  -3.78246  3.57286  0.92688
H  -2.32182  5.28541  2.03687
I   0.62744  1.72341 -2.25434
```

TS2(CuIII-2⁺-10⁺)

```
SCF  =      -1583.72632243
H(0 K)=     -1583.303046
H(298 K)=   -1583.272108
G(298 K)=   -1583.366813
SCF(D3BJ) =       -1583.90014013
SCF(BS2) =        -3313.91490982
SCF(BS2+D3BJ) =   -3314.08872752
Low Freq. = -57.2872cm-1,
12.7837cm-1

Cu  0.06486  0.31995 -0.15910
N   2.16428  0.50647  1.08905
N   1.23764 -1.37549 -0.70144
N  -1.01109 -0.47354  1.50623
N  -1.98857 -0.73604 -1.06749
C   2.61209  1.39275  1.98911
H   1.90052  2.16378  2.30361
C   3.91591  1.37053  2.53569
H   4.21099  2.12841  3.26587
C   4.79357  0.37601  2.12550
H   5.81375  0.32308  2.51909
C   4.35566 -0.59392  1.18589
C   3.01331 -0.48685  0.69549
C   2.52500 -1.47640 -0.24699
C   0.79142 -2.30279 -1.56347
H  -0.24062 -2.18199 -1.89875
C   1.58041 -3.37648 -2.02921
H   1.15095 -4.09555 -2.73109
C   2.89005 -3.48958 -1.58261
H   3.53606 -4.30548 -1.92217
C   3.39812 -2.53058 -0.66825
C   4.74320 -2.59458 -0.16235
H   5.38894 -3.40917 -0.50492
C   5.20546 -1.66120  0.73129
H   6.22823 -1.71484  1.11751
C  -0.54720 -0.35063  2.75893
H   0.40863  0.16781  2.86492
C  -1.22324 -0.85506  3.89104
H  -0.78806 -0.71911  4.88417
C  -2.43131 -1.51496  3.71035
H  -2.98730 -1.92000  4.56183
C  -2.95473 -1.66503  2.39953
C  -2.20072 -1.12234  1.30924
C  -2.71835 -1.25745 -0.04196
C  -2.47629 -0.83580 -2.31113
H  -1.86038 -0.39397 -3.10388
C  -3.70560 -1.46440 -2.61897
H  -4.04713 -1.51468 -3.65614
C  -4.45264 -2.01071 -1.58264
H  -5.40738 -2.50992 -1.77722
C  -3.97288 -1.91768 -0.24894
C  -4.69727 -2.45313  0.87200
H  -5.65125 -2.95702  0.68675
C  -4.20435 -2.33269  2.14757
H  -4.75671 -2.73946  3.00060
C  -0.67447  2.26034 -0.11903
C   0.07537  3.17206  0.65157
C  -2.08294  2.32498 -0.14750
C  -0.59857  4.05879  1.51038
C  -2.73746  3.21804  0.71688
C  -2.00247  4.07876  1.55245
H   1.16338  3.20446  0.57177
H  -2.65949  1.67552 -0.80794
H  -0.01431  4.75102  2.12559
H  -3.83222  3.24436  0.72182
H  -2.52277  4.78263  2.20904
I   0.43311  1.80248 -2.27095
```

10^+

```
SCF   =       -1340.65892643
H(0 K)=      -1340.323056
H(298 K)=    -1340.299813
G(298 K)=    -1340.376237
SCF(D3BJ) =        -1340.77522377
SCF(BS2) =         -2784.38586531
SCF(BS2+D3BJ) =    -2784.50216271
Low Freq. = 14.1956cm-1,
19.3534cm-1

C    5.22511  0.48632  0.48658
C    3.99155  1.01210  1.01228
C    2.75358  0.51030  0.51028
C    2.75376 -0.51002 -0.51020
C    3.99191 -1.01175 -1.01182
C    5.22528 -0.48590 -0.48574
N    1.53359  0.95430  0.95425
C    1.51449  1.90550  1.90555
C    2.68896  2.45965  2.45987
C    3.93101  2.01503  2.01533
C    3.93173 -2.01469 -2.01490
C    2.68984 -2.45936 -2.45981
C    1.51517 -1.90528 -1.90587
N    1.53392 -0.95408 -0.95454
Cu  -0.00000 -0.00019 -0.00050
N   -1.53388  0.95444 -0.95420
C   -2.75374  0.51026 -0.51003
C   -3.99186  1.01222 -1.01148
C   -3.93164  2.01551 -2.01419
C   -2.68973  2.46031 -2.45893
C   -1.51509  1.90599 -1.90517
C   -5.22525  0.48620 -0.48562
C   -5.22513 -0.48638  0.48634
C   -3.99159 -1.01239  1.01186
C   -2.75360 -0.51043  0.51008
N   -1.53363 -0.95462  0.95391
C   -1.51457 -1.90618  1.90486
C   -2.68906 -2.46052  2.45893
C   -3.93110 -2.01571  2.01453
H   -0.52648 -2.23866  2.23709
H   -2.60578 -3.23135  3.22924
H   -4.85776 -2.42771  2.42635
H   -0.52709  2.23848 -2.23765
H   -2.60665  3.23112 -3.22928
H   -4.85841  2.42750 -2.42577
H   -6.16844  0.87905 -0.87808
H   -6.16821 -0.87923  0.87905
H    0.52638  2.23785  2.23786
H    2.60564  3.23017  3.23047
H    4.85765  2.42688  2.42733
H    0.52718 -2.23768 -2.23849
H    2.60680 -3.22989 -3.23044
H    4.85852 -2.42651 -2.42662
H    6.16849 -0.87857 -0.87833
H    6.16817  0.87904  0.87946
```

CuI-2$_{I}$

```
SCF   =       -1352.29271225
H(0 K)=      -1351.958015
H(298 K)=    -1351.931684
G(298 K)=    -1352.018189
SCF(D3BJ) =        -1352.42367079
SCF(BS2) =         -3082.40304118
SCF(BS2+D3BJ) =    -3082.53402793
Low Freq. = 11.6434cm-1,
21.6073cm-1

C    3.81057 -2.86991  0.13416
C    2.48463 -2.41007  0.31079
N    2.07596 -1.17939 -0.02564
C    2.99573 -0.31960 -0.54939
C    4.36030 -0.69024 -0.77933
C    4.75007 -2.00742 -0.42059
C    5.27128  0.26316 -1.35496
C    4.85055  1.52866 -1.68349
C    3.49187  1.94458 -1.45023
C    2.55893  1.02534 -0.87298
C    3.03124  3.24951 -1.76439
C    1.70622  3.58182 -1.50237
C    0.85833  2.61608 -0.92188
N    1.25793  1.37022 -0.60198
Cu   0.00989 -0.02713  0.26759
N   -2.06577  1.19081  0.18392
C   -2.98314  0.44380 -0.49403
C   -4.34920  0.84806 -0.64801
C   -4.74289  2.07002 -0.04185
C   -3.80581  2.81301  0.66782
C   -2.47796  2.33248  0.75048
C   -5.25886  0.02200 -1.39632
C   -4.83606 -1.15567 -1.96232
C   -3.47668 -1.60625 -1.81313
C   -2.54414 -0.81364 -1.07042
C   -3.01599 -2.82662 -2.37179
C   -1.69090 -3.20277 -2.17939
C   -0.84317 -2.36573 -1.42518
N   -1.24288 -1.20440 -0.87187
I   -0.02676 -0.32102  2.91897
H    5.54596  2.25049 -2.12411
H    6.30886 -0.04176 -1.52635
H   -5.53032 -1.78041 -2.53368
H   -6.29703  0.35250 -1.50572
H   -0.18801  2.84800 -0.70424
H    1.31178  4.57499 -1.73287
H    3.72116  3.97518 -2.20688
H    1.72321 -3.06877  0.74542
H    4.07706 -3.88914  0.42705
H    5.78498 -2.32684 -0.58183
H    0.20317 -2.63552 -1.25608
```

```
H  -1.29644 -4.13335 -2.59595
H  -3.70632 -3.45452 -2.94414
H  -1.71789  2.89938  1.30153
H  -4.07545  3.75576  1.15171
H  -5.77915  2.41054 -0.13776
```

CuI-2$_{Cl}$

```
SCF  =      -1355.85738069
H(0 K)=     -1355.522342
H(298 K)=   -1355.496252
G(298 K)=   -1355.580728
SCF(D3BJ) =        -1355.98311442
SCF(BS2) =         -3244.81054590
SCF(BS2+D3BJ) =    -3244.93627961
Low Freq. = 13.4883cm-1,
24.5265cm-1

C  -3.79437  2.89502  0.86493
C  -2.46697  2.41740  0.97126
N  -2.07261  1.21634  0.52805
C  -3.00835  0.40529 -0.04344
C  -4.37615  0.79870 -0.20960
C  -4.75073  2.08326  0.26509
C  -5.30710 -0.10041 -0.83785
C  -4.90214 -1.33629 -1.27893
C  -3.54004 -1.77418 -1.11726
C  -2.58700 -0.90929 -0.49080
C  -3.09682 -3.04966 -1.55355
C  -1.76686 -3.40783 -1.35897
C  -0.89670 -2.49744 -0.72576
N  -1.27929 -1.28035 -0.29034
Cu  0.00048 -0.00044  0.65056
N   2.07265 -1.21561  0.52820
C   3.00848 -0.40455 -0.04317
C   4.37610 -0.79823 -0.20997
C   4.75044 -2.08317  0.26390
C   3.79402 -2.89494  0.86364
C   2.46679 -2.41701  0.97071
C   5.30704  0.10101 -0.83806
C   4.90221  1.33723 -1.27834
C   3.54028  1.77537 -1.11594
C   2.58730  0.91034 -0.48964
C   3.09713  3.05119 -1.55132
C   1.76730  3.40947 -1.35602
C   0.89717  2.49887 -0.72304
N   1.27971  1.28146 -0.28847
Cl -0.00138 -0.00396  3.07246
H  -5.61242 -2.01763 -1.75853
H  -6.34691  0.22143 -0.95699
H   5.61250  2.01865 -1.75782
H   6.34673 -0.22102 -0.95776
H   0.15448 -2.74996 -0.56058
H  -1.38492 -4.37924 -1.68390
H  -3.80373 -3.73233 -2.03580
H  -1.69256  3.03844  1.43752
H  -4.04860  3.88772  1.24649
H  -5.78768  2.41760  0.15566
H  -0.15391  2.75144 -0.55740
H   1.38541  4.38115 -1.68024
H   3.80396  3.73402 -2.03346
H   1.69236 -3.03806  1.43691
H   4.04808 -3.88793  1.24457
H   5.78724 -2.41779  0.15394
```

CuI-3

```
SCF  =      -1569.28000668
H(0 K)=     -1568.897118
H(298 K)=   -1568.867211
G(298 K)=   -1568.961338
SCF(D3BJ) =        -1569.41265937
SCF(BS2) =         -3013.10703887
SCF(BS2+D3BJ) =    -3013.23977885
Low Freq. = 12.8022cm-1,
16.5569cm-1

Cu -0.01358 -0.04860  0.44959
O   0.27619  0.04095  2.44629
O  -0.63047 -2.01333  2.85758
N  -2.09592  1.19299  0.50626
N  -1.25025 -1.09402 -0.75444
N   1.25776  1.36100 -0.37419
N   2.10319 -1.22593  0.04528
C  -2.52185  2.26722  1.18497
H  -1.75833  2.81147  1.75396
C  -3.86373  2.71385  1.18524
H  -4.14345  3.60135  1.75964
C  -4.80074  2.01492  0.43095
H  -5.84695  2.33583  0.38998
C  -4.39297  0.86715 -0.29768
C  -3.01472  0.48315 -0.21118
C  -2.56108 -0.70364 -0.91009
C  -0.83862 -2.18743 -1.43191
H   0.21164 -2.45842 -1.29206
C  -1.67827 -2.95177 -2.26475
H  -1.27208 -3.82769 -2.77778
C  -3.01330 -2.58312 -2.40674
H  -3.69905 -3.16129 -3.03416
C  -3.48723 -1.43604 -1.71988
C  -4.85851 -1.00349 -1.80125
H  -5.54861 -1.58340 -2.42293
C  -5.29854  0.09770 -1.10864
H  -6.34631  0.41100 -1.16520
C   0.85130  2.62160 -0.62077
H  -0.20752  2.82102 -0.42951
C   1.70550  3.63868 -1.09410
H   1.30461  4.64020 -1.27165
C   3.04619  3.34402 -1.31910
H   3.74281  4.10846 -1.67807
```

C   3.51583 2.02721 -1.07513
C   2.57541 1.05304 -0.60814
C   3.02546 -0.30594 -0.36098
C   2.52967 -2.46693 0.31514
H   1.76384 -3.17744 0.64903
C   3.87648 -2.88049 0.18636
H   4.15611 -3.91073 0.42337
C   4.81799 -1.95811 -0.25607
H   5.86889 -2.23876 -0.38288
C   4.40997 -0.62921 -0.54507
C   5.32615 0.37916 -1.00713
H   6.37779 0.10696 -1.14517
C   4.89295 1.65542 -1.26991
H   5.59190 2.42000 -1.62465
C -0.08902 -0.93917 3.21891
C   0.19703 -0.69773 4.71387
H -0.11397 -1.55996 5.32242
H -0.33786 0.20242 5.06266
H   1.27308 -0.51112 4.87189

CuI-4$_{I}$

SCF =   -780.660645832
H(0 K)=   -780.492324
H(298 K)=   -780.477694
G(298 K)=   -780.536380
SCF(D3BJ) =   -780.722008809
SCF(BS2) =   -2510.57718792
SCF(BS2+D3BJ) =   -2510.63854839
Low Freq. = 21.6629cm-1, 27.5938cm-1

N -0.63913 -1.35838 0.00044
C -1.85218 -0.72266 0.00011
C -3.09331 -1.42637 -0.00021
C -3.03781 -2.84609 -0.00069
C -1.80089 -3.48187 -0.00020
C -0.62261 -2.70113 0.00046
C -4.32589 -0.68177 -0.00013
C -4.32353 0.69300 -0.00023
C -3.08824 1.43314 -0.00026
C -1.84966 0.72493 0.00007
N -0.63436 1.35614 0.00056
C -0.61274 2.69875 0.00037
C -1.78813 3.48384 -0.00024
C -3.02738 2.85265 -0.00054
Cu 0.91487 -0.00504 0.00087
H   0.37638 3.16673 0.00079
H -1.70654 4.57364 -0.00055
H -3.95499 3.43369 -0.00100
H   0.36471 -3.17286 0.00096
H -1.72318 -4.57195 -0.00039
H -3.96777 -3.42345 -0.00143
H -5.26957 -1.23622 0.00001
H -5.26540 1.25059 -0.00030
I   3.39743 -0.00032 -0.00040

CuI-4$_{Cl}$

SCF =   -784.225100687
H(0 K)=   -784.056397
H(298 K)=   -784.042025
G(298 K)=   -784.098345
SCF(D3BJ) =   -784.284241985
SCF(BS2) =   -2672.98545744
SCF(BS2+D3BJ) =   -2673.04459874
Low Freq. = 36.1981cm-1, 43.6291cm-1

N -0.20195 -1.37942 -0.00005
C   0.97628 -0.67560 0.00014
C   2.25249 -1.31336 0.00034
C   2.27617 -2.73358 0.00034
C   1.07611 -3.43635 0.00015
C -0.14309 -2.72423 -0.00004
C   3.44432 -0.50476 0.00053
C   3.37115 0.86795 0.00052
C   2.09889 1.54179 0.00032
C   0.89801 0.77006 0.00013
N -0.34668 1.33297 -0.00006
C -0.44045 2.66990 -0.00007
C   0.69028 3.52003 0.00010
C   1.96165 2.95614 0.00030
Cu -1.82751 -0.24197 -0.00029
H -1.45433 3.08403 -0.00023
H   0.55099 4.60412 0.00008
H   2.85686 3.58611 0.00044
H -1.10150 -3.25042 -0.00020
H   1.05763 -4.52891 0.00015
H   3.23656 -3.25871 0.00049
H   4.41523 -1.00992 0.00069
H   4.28330 1.47298 0.00067
Cl -3.98691 0.05972 -0.00057

CuIII-2

SCF =   -1800.72682958
H(0 K)=   -1800.253505
H(298 K)=   -1800.218970
G(298 K)=   -1800.320279
SCF(D3BJ) =   -1800.90518237
SCF(BS2) =   -3244.62391880
SCF(BS2+D3BJ) =   -3244.80227159
Low Freq. = 20.2443cm-1, 27.6237cm-1

Cu -0.03634 0.41292 0.24067
O -1.04747 1.43746 1.51272
O -0.02568 0.48811 3.32565
N   2.05977 -0.62339 1.15022
N   0.90432 -0.39932 -1.34241

```
N  -1.21389 -1.23966  0.54159
N  -2.21597  0.84578 -1.01999
C   2.62786 -0.69260  2.36057
H   1.99979 -0.34025  3.18539
C   3.93809 -1.18745  2.56988
H   4.35080 -1.21785  3.58181
C   4.67154 -1.63930  1.47946
H   5.68250 -2.04031  1.60446
C   4.09625 -1.58634  0.18150
C   2.77557 -1.04617  0.07545
C   2.14780 -0.96133 -1.22681
C   0.29624 -0.37195 -2.53980
H  -0.69961  0.07804 -2.55617
C   0.89594 -0.88542 -3.70711
H   0.35012 -0.83791 -4.65205
C   2.17383 -1.42376 -3.62744
H   2.67823 -1.81130 -4.51800
C   2.83470 -1.47527 -2.37340
C   4.15854 -2.01875 -2.22301
H   4.66666 -2.39588 -3.11569
C   4.77209 -2.06175 -0.99514
H   5.78160 -2.47174 -0.89109
C  -0.72555 -2.27731  1.24036
H   0.32870 -2.20839  1.51593
C  -1.51118 -3.38618  1.61667
H  -1.05336 -4.20160  2.18141
C  -2.85602 -3.40053  1.27414
H  -3.50373 -4.23383  1.56453
C  -3.40442 -2.31677  0.54007
C  -2.53400 -1.24185  0.16584
C  -3.06903 -0.13627 -0.60924
C  -2.72525  1.88873 -1.68556
H  -2.01220  2.66356 -1.98984
C  -4.09720  2.02082 -2.00578
H  -4.44704  2.90114 -2.55114
C  -4.96809  1.00702 -1.62969
H  -6.03395  1.05724 -1.87448
C  -4.46899 -0.11416 -0.91409
C  -5.31449 -1.20294 -0.50591
H  -6.37815 -1.16458 -0.76134
C  -4.79703 -2.27117  0.18244
H  -5.43648 -3.10611  0.48490
C  -0.86494  1.24178  2.80592
C  -1.81194  2.09907  3.65120
H  -1.91518  1.65536  4.65185
H  -2.79889  2.20288  3.17655
H  -1.37934  3.10895  3.75597
C   0.97892  2.06315 -0.02244
C   0.87746  2.72201 -1.24941
C   1.73636  2.56590  1.03970
C   1.57843  3.93619 -1.42020
C   2.43438  3.77576  0.84801
C   2.35541  4.45933 -0.37641
H   0.29457  2.31840 -2.08156
H   1.77723  2.05006  2.00172
H   1.51200  4.45559 -2.38218
H   3.03003  4.18051  1.67342
H   2.89772  5.39992 -0.51543
```

CuIII-3$_I$

```
SCF  =       -1583.72811198
H(0 K)=      -1583.303455
H(298 K)=    -1583.272354
G(298 K)=    -1583.367260
SCF(D3BJ) =        -1583.90713998
SCF(BS2) =         -3313.91307266
SCF(BS2+D3BJ) =    -3314.09210068
Low Freq. = 13.8349cm-1,
21.7228cm-1
```

```
Cu -0.03176 -0.41483  0.09098
N  -1.64778 -0.08616  1.24514
N  -0.80914  1.08776 -1.02578
N   1.24721  1.49077  1.05447
N   1.74352 -0.52521 -0.80525
C  -2.06179 -0.72123  2.35156
H  -1.42980 -1.53525  2.71211
C  -3.25077 -0.35985  3.02292
H  -3.53922 -0.91271  3.91978
C  -4.02581  0.68770  2.53738
H  -4.94836  0.98621  3.04436
C  -3.61443  1.37387  1.36461
C  -2.40684  0.94198  0.74330
C  -1.95392  1.57560 -0.45966
C  -0.35394  1.64128 -2.15684
H   0.55899  1.21532 -2.58053
C  -1.02016  2.71705 -2.78577
H  -0.60583  3.13307 -3.70691
C  -2.19076  3.22134 -2.22754
H  -2.72635  4.04930 -2.70179
C  -2.69646  2.64917 -1.03005
C  -3.90334  3.08071 -0.37401
H  -4.46862  3.90771 -0.81357
C  -4.34513  2.46578  0.77420
H  -5.26745  2.79553  1.26145
C   0.99950  2.47524  1.92543
H   0.01960  2.45596  2.41533
C   1.93144  3.49714  2.22737
H   1.67082  4.27499  2.94959
C   3.16862  3.48218  1.59571
H   3.91897  4.25102  1.80537
C   3.46600  2.45403  0.66122
C   2.45178  1.47243  0.42184
C   2.70789  0.41328 -0.53601
C   1.97366 -1.50333 -1.69599
H   1.14539 -2.20231 -1.86059
C   3.19097 -1.60713 -2.39672
H   3.32500 -2.41874 -3.11518
```

```
C   4.19031 -0.67406 -2.15194
H   5.14994 -0.72960 -2.67510
C   3.96930  0.36750 -1.21374
C   4.96328  1.36935 -0.93505
H   5.91820  1.31088 -1.46570
C   4.72160  2.37625 -0.03378
H   5.48096  3.13780  0.16861
C   0.67425 -1.80709  1.28771
C   1.30227 -1.36380  2.45599
C   0.50973 -3.15957  0.98117
C   1.75333 -2.33441  3.37688
C   0.97061 -4.11059  1.91770
C   1.58896 -3.70148  3.10850
H   1.43647 -0.30234  2.67158
H   0.01645 -3.48277  0.06037
H   2.23713 -1.99917  4.30035
H   0.83432 -5.17433  1.69533
H   1.94595 -4.44738  3.82555
I  -1.69338 -2.10508 -1.78051
```

CuIII-3$_{Cl}$

```
SCF  =       -1587.29599236
H(0 K)=      -1586.870839
H(298 K)=    -1586.840100
G(298 K)=    -1586.932044
SCF(D3BJ) =        -1587.46786364
SCF(BS2) =         -3476.32476501
SCF(BS2+D3BJ) =    -3476.49663629
Low Freq. = 21.9504cm-1,
28.8611cm-1

C  -1.76537  4.63216  1.05854
C  -1.77142  3.51543  1.90797
C  -1.25398  2.27690  1.46855
C  -0.72651  2.21574  0.17677
C  -0.71542  3.29878 -0.70273
C  -1.23995  4.52495 -0.23828
Cu  0.11834  0.54452 -0.41292
Cl  1.06786  1.37460 -2.80026
N   1.85526  0.99550  0.45864
C   2.21994  2.09903  1.12840
C   3.51680  2.25528  1.66561
C   4.45706  1.24405  1.50380
C   4.10023  0.06726  0.79427
C   2.77247 -0.01182  0.28308
C   2.35587 -1.16947 -0.45240
C   3.26352 -2.24419 -0.67567
C   4.59727 -2.14579 -0.14229
C   4.99831 -1.03522  0.56307
C   2.78239 -3.35119 -1.42442
C   1.47578 -3.33856 -1.90179
C   0.64566 -2.22588 -1.63374
N   1.07456 -1.17407 -0.92657
N  -0.75294 -0.87392  1.43111
C  -0.29431 -1.34027  2.59786
C  -1.03518 -2.21469  3.42896
C  -2.30386 -2.60945  3.02320
C  -2.82405 -2.13572  1.78880
C  -1.99121 -1.26134  1.02004
C  -2.47666 -0.76716 -0.25450
C  -3.77486 -1.15944 -0.71469
C  -4.58251 -2.03113  0.09599
C  -4.12555 -2.50296  1.30160
C  -4.21624 -0.67282 -1.97256
C  -3.38868  0.15137 -2.72477
C  -2.12332  0.50118 -2.21441
N  -1.69007  0.05212 -1.02429
H   1.46112  2.87565  1.24053
H   3.75766  3.17477  2.20379
H   5.46640  1.34533  1.91386
H  -0.38212 -2.18599 -2.00394
H   1.07614 -4.17274 -2.48294
H   3.44305 -4.20081 -1.62171
H   5.29026 -2.97491 -0.31312
H   6.01509 -0.96794  0.96115
H   0.70521 -1.00495  2.89637
H  -0.60600 -2.56238  4.37211
H  -2.90950 -3.28160  3.63921
H  -1.41488  1.13234 -2.76322
H  -3.69458  0.53670 -3.69994
H  -5.20792 -0.95666 -2.33826
H  -5.57130 -2.31385 -0.27733
H  -4.74457 -3.16917  1.91037
H  -1.26035  1.41154  2.13360
H  -0.29212  3.20594 -1.70775
H  -2.18020  3.58555  2.92149
H  -1.22824  5.39026 -0.90966
H  -2.17358  5.58615  1.40676
```

CuIII-4

```
SCF  =       -1229.09973476
H(0 K)=      -1228.793591
H(298 K)=    -1228.770451
G(298 K)=    -1228.846275
SCF(D3BJ) =        -1229.20117226
SCF(BS2) =         -2672.80414812
SCF(BS2+D3BJ) =    -2672.90558561
Low Freq. = 26.5810cm-1,
35.7031cm-1

Cu  0.57078  0.40780 -0.19512
N  -0.40895 -1.27259  0.08290
N  -1.27440  1.21728 -0.32275
C   0.06783 -2.51084  0.29026
H   1.15334 -2.62536  0.28243
C  -0.79052 -3.61077  0.50717
H  -0.35003 -4.59661  0.67056
C  -2.16914 -3.42595  0.50658
```

H -2.84691 -4.26902 0.67033
C -2.69991 -2.12651 0.28995
C -1.76824 -1.07150 0.08440
C -2.23392 0.26411 -0.13762
C -1.64434 2.48245 -0.55568
H -0.83701 3.20376 -0.71048
C -3.00641 2.86034 -0.60359
H -3.26267 3.90471 -0.79490
C -3.99570 1.90108 -0.40537
H -5.05428 2.17642 -0.43541
C -3.62607 0.54950 -0.16529
C -4.55305 -0.53250 0.04506
H -5.62470 -0.31445 0.02573
C -4.10708 -1.81602 0.26511
H -4.81988 -2.63027 0.42435
C 2.30231 -0.43500 -0.25591
C 3.02089 -0.62718 0.92445
C 2.72795 -0.87153 -1.51193
C 4.23300 -1.34657 0.83406
C 3.94325 -1.58755 -1.57238
C 4.68812 -1.82269 -0.40561
H 2.67116 -0.23440 1.88219
H 2.15216 -0.68574 -2.42355
H 4.81429 -1.51996 1.74565
H 4.29482 -1.95272 -2.54289
H 5.63172 -2.37391 -0.46439
O 1.52045 2.01066 -0.51207
O 1.28189 1.97479 1.73163
C 1.68516 2.53864 0.69667
C 2.42718 3.86387 0.69525
H 2.20507 4.44957 -0.20850
H 2.16560 4.43431 1.59726
H 3.51140 3.65855 0.70920

CuIII-5$_{I}$
SCF = -1012.09804729
H(0 K)= -1011.841128
H(298 K)= -1011.821225
G(298 K)= -1011.890815
SCF(D3BJ) = -1012.19549524
SCF(BS2) = -2742.09411850
SCF(BS2+D3BJ) = -2742.19157160
Low Freq. = 17.9011cm-1,
34.8257cm-1

Cu 0.39795 -0.20911 -0.01033
N -0.84802 1.37580 -0.01903
N -1.34631 -1.25753 0.00021
C -0.54937 2.68188 -0.02941
H 0.51306 2.93955 -0.03837
C -1.55324 3.67623 -0.02905
H -1.25850 4.72789 -0.03746
C -2.89304 3.30145 -0.01716
H -3.68492 4.05664 -0.01615
C -3.23558 1.92249 -0.00618
C -2.16097 0.98906 -0.00799
C -2.42902 -0.42111 0.00225
C -1.55179 -2.58165 0.00808
H -0.65531 -3.21039 0.00617
C -2.84987 -3.14034 0.01851
H -2.96165 -4.22688 0.02406
C -3.95932 -2.30090 0.02145
H -4.97308 -2.71262 0.02933
C -3.77172 -0.89223 0.01385
C -4.84372 0.06989 0.01705
H -5.87413 -0.29753 0.02760
C -4.58601 1.42158 0.00731
H -5.40844 2.14272 0.00997
C 2.05324 0.82522 0.00716
C 2.50853 1.33337 -1.21718
C 2.48042 1.32089 1.24664
C 3.37397 2.44587 -1.18769
C 3.34599 2.43359 1.24830
C 3.79181 2.99063 0.03806
H 2.19943 0.89882 -2.17168
H 2.14890 0.87752 2.18946
H 3.72433 2.87052 -2.13394
H 3.67403 2.84874 2.20669
H 4.47777 3.84301 0.05026
I 2.12974 -1.99957 -0.00979

CuIII-5$_{Cl}$
SCF = -1015.65910693
H(0 K)= -1015.401504
H(298 K)= -1015.382033
G(298 K)= -1015.449345
SCF(D3BJ) = -1015.75181016
SCF(BS2) = -2904.50071372
SCF(BS2+D3BJ) = -2904.59341695
Low Freq. = 21.4574cm-1,
41.5143cm-1

Cu 0.60993 -0.79971 -0.00400
N -0.09714 1.04750 -0.00449
N -1.35245 -1.30986 -0.00103
C 0.57392 2.20946 -0.00684
H 1.66395 2.14698 -0.00907
C -0.09485 3.45309 -0.00637
H 0.49903 4.36951 -0.00840
C -1.48530 3.49186 -0.00319
H -2.01766 4.44778 -0.00255
C -2.21914 2.27572 -0.00079
C -1.46978 1.06621 -0.00171
C -2.14415 -0.19706 0.00031
C -1.92487 -2.51997 0.00080
H -1.24311 -3.37606 -0.00044
C -3.33024 -2.67695 0.00419
H -3.75330 -3.68391 0.00565

C -4.14992 -1.55202 0.00557
H -5.23915 -1.65480 0.00816
C -3.56461 -0.25651 0.00357
C -4.30482 0.97890 0.00462
H -5.39777 0.93400 0.00715
C -3.65777 2.19370 0.00246
H -4.23012 3.12570 0.00317
C 2.46920 -0.24332 0.00095
C 3.06792 0.01916 -1.23148
C 3.05906 0.01966 1.23757
C 4.32651 0.65904 -1.21097
C 4.31778 0.65950 1.22577
C 4.94472 0.97483 0.00957
H 2.59278 -0.24023 -2.18156
H 2.57695 -0.23901 2.18435
H 4.81337 0.89615 -2.16241
H 4.79774 0.89714 2.18057
H 5.92570 1.45955 0.01300
Cl 1.46993 -2.78746 -0.00513

CuIII-6
SCF = -1457.74214671
H(0 K)= -1457.388207
H(298 K)= -1457.358764
G(298 K)= -1457.450894
SCF(D3BJ) = -1457.86152103
SCF(BS2) = -2901.54746562
SCF(BS2+D3BJ) = -2901.66683994
Low Freq. = 18.4379cm-1, 25.9983cm-1

Cu -0.60629 -0.43852 0.11072
O -1.38605 -2.08642 -0.69149
O -0.67526 -1.23061 -2.67726
N 1.28035 -1.10925 0.00113
N 0.31403 1.36366 -0.33929
C 1.70399 -2.36770 0.16482
H 0.92750 -3.11802 0.33802
C 3.07516 -2.70671 0.10480
H 3.37482 -3.74807 0.24465
C 4.01882 -1.71058 -0.13174
H 5.08572 -1.95000 -0.18035
C 3.59031 -0.36883 -0.31463
C 2.18989 -0.12139 -0.23765
C 1.67270 1.20480 -0.41649
C -0.19590 2.59280 -0.49639
H -1.28393 2.67733 -0.43273
C 0.61596 3.72445 -0.73986
H 0.14243 4.70188 -0.85972
C 1.99621 3.57593 -0.82525
H 2.64446 4.43698 -1.01530
C 2.56702 2.28582 -0.66299
C 3.98101 2.01486 -0.73308
H 4.66084 2.85107 -0.92319
C 4.47331 0.74088 -0.56706
H 5.54925 0.54994 -0.62351
C -1.20632 -2.12912 -1.99219
C -1.75249 -3.40538 -2.63942
H -1.26241 -3.57591 -3.60888
H -2.83598 -3.28281 -2.81119
H -1.61420 -4.27883 -1.98453
O -0.78228 -1.01755 2.09652
O 0.76178 0.54041 2.72402
C -0.00875 -0.41383 2.96147
C -0.14057 -0.97278 4.38943
H 0.65348 -0.57035 5.03527
H -0.09788 -2.07381 4.38565
H -1.12070 -0.68584 4.80826
C -2.39187 0.29433 0.04244
C -2.86328 0.68878 -1.21657
C -3.13944 0.45918 1.21121
C -4.12459 1.31675 -1.29104
C -4.39869 1.08986 1.11340
C -4.88787 1.52042 -0.12980
H -2.27701 0.50084 -2.12077
H -2.75708 0.10389 2.17077
H -4.50386 1.63320 -2.26899
H -4.98980 1.23736 2.02411
H -5.86720 2.00548 -0.19585

CuIII-7$_{I}$
SCF = -1023.75081110
H(0 K)= -1023.494186
H(298 K)= -1023.471620
G(298 K)= -1023.549532
SCF(D3BJ) = -1023.86352305
SCF(BS2) = -3040.13039879
SCF(BS2+D3BJ) = -3040.24311076
Low Freq. = 20.5417cm-1, 24.5737cm-1

Cu 0.38116 -0.10184 -0.27109
N -0.89558 1.50084 -0.24252
N -1.37487 -1.11528 -0.62792
C -0.61724 2.79073 -0.01317
H 0.43822 3.04185 0.12530
C -1.62655 3.77782 0.04515
H -1.34581 4.81647 0.23474
C -2.95578 3.41188 -0.14390
H -3.75388 4.15984 -0.10766
C -3.27900 2.04976 -0.38269
C -2.19790 1.12162 -0.41818
C -2.45430 -0.27597 -0.63162
C -1.57682 -2.42616 -0.81584
H -0.68202 -3.05740 -0.80841
C -2.86508 -2.97098 -1.01646
H -2.97165 -4.04802 -1.16576

C   -3.97305 -2.12893 -1.01889
H   -4.98115 -2.52761 -1.16897
C   -3.79013 -0.73455 -0.82098
C   -4.86440 0.22448 -0.79520
H   -5.88717 -0.13483 -0.94356
C   -4.61915 1.56134 -0.58284
H   -5.44402 2.27980 -0.55914
C   2.01134 0.98916 -0.47061
C   2.14588 1.69055 -1.67923
C   2.75122 1.31166 0.67325
C   2.99297 2.81611 -1.70620
C   3.59515 2.44048 0.62184
C   3.71647 3.18880 -0.56063
H   1.60183 1.38981 -2.57884
H   2.65055 0.72543 1.59099
H   3.08926 3.38661 -2.63588
H   4.16038 2.72020 1.51719
H   4.38542 4.05436 -0.59413
I   2.09588 -1.75728 -1.13732
I   0.15351 -0.68760 2.63442

CuIII-7$_{Cl}$
SCF =   -1030.87879222
H(0 K)=   -1030.621322
H(298 K)=   -1030.599434
G(298 K)=   -1030.672924
SCF(D3BJ) =   -1030.98083204
SCF(BS2) =   -3364.94659870
SCF(BS2+D3BJ) =   -3365.04863853
Low Freq. = 24.7774cm-1,
32.3728cm-1

Cu   0.59962 -0.73089 -0.01426
N   -0.15656 1.15164 -0.00813
N   -1.36826 -1.22897 -0.23032
C   0.48170 2.32090 0.14092
H   1.57161 2.27681 0.20125
C   -0.21184 3.54980 0.21060
H   0.35903 4.47353 0.32941
C   -1.59993 3.56404 0.12180
H   -2.15510 4.50592 0.16749
C   -2.30203 2.33884 -0.02664
C   -1.52541 1.14559 -0.08109
C   -2.17416 -0.12711 -0.20911
C   -1.92349 -2.44088 -0.34634
H   -1.22986 -3.28722 -0.36392
C   -3.32315 -2.61587 -0.44566
H   -3.72932 -3.62571 -0.53952
C   -4.15886 -1.50304 -0.42044
H   -5.24491 -1.61606 -0.49257
C   -3.59312 -0.20564 -0.29796
C   -4.35668 1.01464 -0.25305
H   -5.44676 0.95164 -0.32232
C   -3.73611 2.23542 -0.11986
H   -4.32623 3.15579 -0.08034
C   2.43404 -0.10112 -0.22267
C   2.78111 0.39448 -1.48266
C   3.26679 -0.03777 0.89337
C   4.02093 1.05700 -1.60442
C   4.50428 0.62665 0.74222
C   4.87761 1.17042 -0.49692
H   2.12389 0.28771 -2.35010
H   2.95824 -0.46228 1.85230
H   4.30756 1.46983 -2.57739
H   5.16831 0.70797 1.60949
H   5.84259 1.67583 -0.60335
Cl   1.45731 -2.61434 -0.83017
Cl   0.44362 -1.09711 2.51484

References

1. CrystalClear-SM Expert v2.1. Rigaku Americas, The Woodlands, Texas, USA, and Rigaku Corporation, Tokyo, Japan, **2015**.
2. CrysAlisPro v1.171.38.46. Rigaku Oxford Diffraction, Rigaku Corporation, Oxford, U.K., **2015**.
3. Sheldrick, G. M. SHELXT – Integrated space-group and crystal-structure determination. *Acta Cryst. Sect. A* **2015**, *71*, 3–8.
4. Sheldrick, G. M. Crystal structure refinement with SHELXL. *Acta Cryst. Sect. C* **2015**, *71*, 3–8.
5. CrystalStructure v4.3.0. Rigaku Americas, The Woodlands, Texas, USA, and Rigaku Corporation, Tokyo, Japan, **2018**.
6. Dolomanov, O. V.; Bourhis, L. J.; Gildea, R. J.; Howard, J. A. K.; Puschmann, H. OLEX2: a complete structure solution, refinement and analysis program. *J. Appl. Cryst.* **2009**, *42*, 339–341.
7. Jing, B.; Li, L.; Dong J.; Xu T. (Acetato-κ*O*)bis(1,10-phenanthroline-κ^2*N,N'*)copper(II) acetate heptahydrate. *Acta Cryst.* **2011**, *E67*, m464.
8. Barquín, M.; González Garmendia, M. J.; Larrínaga, L.; Pinilla, E.; Torres, M. R. Complexes of $Cu_2(\mu\text{-}OAc)_4(H_2O)_2$ with 1,10-phenanthroline and comparison with those of 2,2'bipyridine. Crystal structure of the dimer $[Cu(OAc)_2(phen)](\mu\text{-}H_2O)$. *Z. Anorg. Allg. Chem.* **2005**, *631*, 2151–2155.
9. Latham, K.; Mensforth, E. J.; Rix, C. J.; White, J. M. Crystal disassembly and reassembly of heterometallic Ni^{II} – Ta^{V} oxalate compounds. *Cryst. Eng. Comm.* **2009**, *11*, 1343–1351.
10. Healy, P. C.; Pakawatchai, C.; White, A. H. Lewis-base adducts of group 1B metal(I) compounds. Part 18. Stereo-chemistries and strucutres of the 1:1 neutral complex of $Cu^{I}X$ with 1,10-phenanthroline (X = I) or 2,9-dimethyl-1,10-phenanthroline (X = I, Br, or Cl). *J. Chem. Soc., Dalton Trans.* **1985**, 2531–2539.
11. Wen Tan, P.; Haughey, M.; Dixon, D. J. Palladium(II)-catalysed *ortho*-arylation of *N*-benzylpiperidines. *Chem. Commun.*, **2015**, *51*, 4406–4409.
12. Frisch, M. J.; Trucks, G. W.; Schlegel, H. B.; Scuseria, G. E.; Robb, M. A.; Cheeseman, J. R.; Scalmani, G.; Barone, V.; Petersson, G. A.; Nakatsuji, H.; Li, X.; Caricato, M.; Marenich, A. V.; Bloino, J.; Janesko, B. G.; Gomperts, R.; Mennucci, B.; Hratchian, H. P.; Ortiz, J. V.; Izmaylov, A. F.; Sonnenberg, J. L.; Williams-Young, D.; Ding, F.; Lipparini, F.; Egidi, F.; Goings, J.; Peng, B.; Petrone, A.; Henderson, T.; Ranasinghe, D.; Zakrzewski, V. G.; Gao, J.; Rega, N.; Zheng, G.; Liang, W.; Hada, M.; Ehara, M.; Toyota, K.; Fukuda, R.; Hasegawa, J.; Ishida, M.; Nakajima, T.; Honda, Y.; Kitao, O.; Nakai, H.; Vreven, T.; Throssell, K.; Montgomery, J. A.; Peralta, J. E.; Ogliaro, F.; Bearpark, M. J.; Heyd, J. J.; Brothers, E. N.; Kudin, K. N.; Staroverov, V. N.; Keith, T. A.; Kobayashi, R.; Normand, J.; Raghavachari, K.; Rendell, A. P.; Burant, J. C.; Iyengar, S. S.; Tomasi, J.; Cossi, M.; Millam, J. M.; Klene, M.; Adamo, C.; Cammi, R.; Ochterski, J. W.; Martin, R. L.; Morokuma, K.; Farkas, O.; Foresman, J. B.; Fox, D. J. Gaussian 16, Revision A.03, Gaussian, Inc: Wallingford CT, **2016**.
13. Becke, A. D. Density-functional exchange-energy approximation with correct asymptotic behavior. *Phys. Rev. A* **1988**, *38*, 3098–3100.
14. Perdew, J. P. Density-functional approximation for the correlation energy of the inhomogeneous electron gas. *Phys. Rev. B* **1986**, *33*, 8822–8824.

15. Andrae, D.; Häußermann, U.; Dolg, M.; Stoll, H.; Preuß, H. Energy-adjusted *ab initio* pseudopotentials for the second and third row transition elements. *Theor. Chim. Acta* **1990**, *77*, 123–141.
16. Höllwarth, A.; Böhme, M.; Dapprich, S.; Ehlers, A. W.; Gobbi, A.; Jonas, V.; Köhler, K. F.; Stegmann, R.; Veldkamp, A.; Frenking, G. A set of d-polarization functions for pseudo-potential basis sets of the main group elements Al Bi and f-type polarization functions for Zn, Cd, Hg. *Chem. Phys. Lett.* **1993**, *208*, 237–240.
17. Hariharan, P. C.; Pople, J. A. The influence of polarization functions on molecular orbital hydrogenation energies. *Theor. Chim. Acta* **1973**, *28*, 213–222.
18. Tomasi, J.; Mennucci, B; Cammi, R. Quantum mechanical continuum solvation models. *Chem. Rev.* **2005**, *105*, 2999–3094.
19. Weigend, F.; Ahlrichs, R. Balanced basis sets of split valence, triple zeta valence and quadruple zeta valence quality for H to Rn: Design and assessment of accuracy. *Phys. Chem. Chem. Phys.* **2005**, *7*, 3297-3305.
20. Grimme, S.; Ehrlich, S; Goerigk, L. Effect of the damping function in dispersion corrected density functional theory. *J. Comput. Chem.* **2011**, *32*, 1456–1465.
21. Cordero, B.; Gómez, V.; Platero-Prats, A. E.; Revés, M.; Echeverría, J.; Cremades, E.; Barragán, F.; Alvarez, S. Covalent radii revisited. *Dalton Trans.* **2008**, 2832–2838.
22. Addison, A. W.; Rao, T. N.; Reedijk, J.; van Rijn, J.; Verschoor, G. C. Synthesis, structure, and spectroscopic properties of copper(II) compounds containing nitrogen–sulphur donor ligands; the crystal and molecular structure of aqua[1,7-bis(N-methylbenzimidazol-2′-yl)-2,6-dithiaheptane]copper(II) perchlorate. *J. Chem. Soc., Dalton Trans.* **1984**, 1349–1356.
