# Supplementary material for: Mechanism of Cu-Catalyzed Iododeboronation: A Description of Ligand-Enabled Transmetalation, Disproportionation, and Turnover in Cu-Mediated Oxidative Coupling Reactions
Source: ACS Catal. 2023 Aug 7;13(16):11117–26. doi: 10.1021/acscatal.3c02839 (PMC10442916; doi:10.1021/acscatal.3c02839)

checkCIF/PLATON report

Structure factors have been supplied for datablock(s) 3Cl, 3OAc, 4, 5, 6

THIS REPORT IS FOR GUIDANCE ONLY. IF USED AS PART OF A REVIEW PROCEDURE FOR PUBLICATION, IT SHOULD NOT REPLACE THE EXPERTISE OF AN EXPERIENCED CRYSTALLOGRAPHIC REFEREE.

No syntax errors found. CIF dictionary Interpreting this report

Datablock: 3OAc

Bond precision: C-C = 0.0035 A Wavelength=0.71075

Cell:	a=8.7325(6)	b=12.2149(8)	c=15.6708(11)
	alpha=103.318(5)	beta=102.145(5)	gamma=97.555(5)
Temperature:	173 K		

	Calculated	Reported
Volume	1561.49(19)	1561.49(19)
Space group	P -1	P -1
Hall group	-P 1	-P 1
Moiety formula	C26 H19 Cu N4 O2, C2 H3 O2, 7(H2 O)	C26 H19 Cu N4 O2, C2 H3 O2, 7(H2 O)
Sum formula	C28 H36 Cu N4 O11	C28 H36 Cu N4 O11
Mr	668.16	668.16
Dx,g cm-3	1.421	1.421
Z	2	2
Mu (mm-1)	0.763	0.763
F000	698.0	698.0
F000’	698.98	
h,k,lmax	11,15,20	11,15,20
Nref	7200	7138
Tmin,Tmax	0.845,0.878	0.635,0.878
Tmin’	0.845	

Correction method= # Reported T Limits: Tmin=0.635 Tmax=0.878
AbsCorr = MULTI-SCAN

Data completeness= 0.991 Theta(max)= 27.523

R(reflections)= 0.0390(5315) wR2(reflections)= 0.1024(7138)

S = 0.989 Npar= 441

The following ALERTS were generated. Each ALERT has the format
test-name_ALERT_alert-type_alert-level.
Click on the hyperlinks for more details of the test.

Alert level C

```
PLAT911_ALERT_3_C Missing FCF Refl Between Thmin & STh/L=    0.600          27 Report
PLAT913_ALERT_3_C Missing # of Very Strong Reflections in FCF ....           8 Note
```

Alert level G

```
PLAT002_ALERT_2_G Number of Distance or Angle Restraints on AtSite          21 Note
PLAT154_ALERT_1_G The s.u.'s on the Cell Angles are Equal ..(Note)       0.005 Degree
PLAT172_ALERT_4_G The CIF-Embedded .res File Contains DFIX Records           1 Report
PLAT794_ALERT_5_G Tentative Bond Valency for Cu1       (II)      .        2.27 Info
PLAT860_ALERT_3_G Number of Least-Squares Restraints ............           14 Note
PLAT910_ALERT_3_G Missing # of FCF Reflection(s) Below Theta(Min).           2 Note
PLAT912_ALERT_4_G Missing # of FCF Reflections Above STh/L=  0.600          34 Note
PLAT933_ALERT_2_G Number of HKL-OMIT Records in Embedded .res File           1 Note
PLAT941_ALERT_3_G Average HKL Measurement Multiplicity ..........          2.3 Low
PLAT978_ALERT_2_G Number C-C Bonds with Positive Residual Density.           4 Info
```

0 **ALERT level A** = Most likely a serious problem - resolve or explain
0 **ALERT level B** = A potentially serious problem, consider carefully
2 **ALERT level C** = Check. Ensure it is not caused by an omission or oversight
10 **ALERT level G** = General information/check it is not something unexpected

1 ALERT type 1 CIF construction/syntax error, inconsistent or missing data
3 ALERT type 2 Indicator that the structure model may be wrong or deficient
5 ALERT type 3 Indicator that the structure quality may be low
2 ALERT type 4 Improvement, methodology, query or suggestion
1 ALERT type 5 Informative message, check

Datablock: 3Cl

Bond precision: C-C = 0.0015 A Wavelength=0.71073

Cell:	a=22.0731(3)	b=12.05397(14)	c=21.9959(3)
	alpha=90	beta=93.9717(10)	gamma=90
Temperature:	93 K		

	Calculated	Reported
Volume	5838.36(13)	5838.36(12)
Space group	C 2/c	C 1 2/c 1
Hall group	-C 2yc	-C 2yc
Moiety formula	C26 H19 Cu N4 O2, 2(C H2 Cl2), Cl, 2(H2 O)	C26 H19 Cu N4 O2, 2(C H2 Cl2), Cl, 2(H2 O)
Sum formula	C28 H27 Cl5 Cu N4 O4	C28 H27 Cl5 Cu N4 O4
Mr	724.34	724.36
Dx,g cm-3	1.648	1.648
Z	8	8
Mu (mm-1)	1.249	1.249
F000	2952.0	2952.0
F000'	2961.35	
h,k,lmax	37,20,37	36,19,36
Nref	14625	13953
Tmin,Tmax	0.883,0.883	0.497,0.883
Tmin'	0.883	

Correction method= # Reported T Limits: Tmin=0.497 Tmax=0.883
AbsCorr = MULTI-SCAN

Data completeness= 0.954 Theta(max)= 36.776

R(reflections)= 0.0321(13025) wR2(reflections)= 0.1053(13953)

S = 1.057 Npar= 393

The following ALERTS were generated. Each ALERT has the format
test-name_ALERT_alert-type_alert-level.
Click on the hyperlinks for more details of the test.

Alert level B

```
PLAT417_ALERT_2_B Short Inter D-H..H-D       H34A     ..H34B     .       2.06 Ang.
                                                1-x,2-y,1-z  =      5_676 Check
PLAT934_ALERT_3_B Number of (Iobs-Icalc)/Sigma(W) > 10 Outliers ..          2 Check
```

Alert level C

```
PLAT244_ALERT_4_C Low    'Solvent' Ueq as Compared to Neighbors of        C32 Check
PLAT911_ALERT_3_C Missing FCF Refl Between Thmin & STh/L=    0.600          2 Report
PLAT972_ALERT_2_C Check Calcd Resid. Dens.  0.37Ang From O34          -1.72 eA-3
PLAT972_ALERT_2_C Check Calcd Resid. Dens.  0.38Ang From O34          -1.62 eA-3
PLAT977_ALERT_2_C Check Negative Difference Density on H30A      .     -0.65 eA-3
PLAT977_ALERT_2_C Check Negative Difference Density on H30B      .     -0.59 eA-3
PLAT977_ALERT_2_C Check Negative Difference Density on H30C      .     -0.70 eA-3
```

Alert level G

```
PLAT002_ALERT_2_G Number of Distance or Angle Restraints on AtSite          6 Note
PLAT083_ALERT_2_G SHELXL Second Parameter in WGHT  Unusually Large       5.40 Why ?
PLAT172_ALERT_4_G The CIF-Embedded .res File Contains DFIX Records          2 Report
PLAT232_ALERT_2_G Hirshfeld Test Diff (M-X)  Cu1      --N1       .        6.3 s.u.
PLAT232_ALERT_2_G Hirshfeld Test Diff (M-X)  Cu1      --N8       .        6.7 s.u.
PLAT232_ALERT_2_G Hirshfeld Test Diff (M-X)  Cu1      --N15      .        8.7 s.u.
PLAT380_ALERT_4_G Incorrectly? Oriented X(sp2)-Methyl Moiety .....        C30 Check
PLAT431_ALERT_2_G Short Inter HL..A Contact  Cl3      ..O30      .       2.98 Ang.
                                                      x,y,z  =      1_555 Check
PLAT432_ALERT_2_G Short Inter X...Y Contact  O34      ..C32      .       2.99 Ang.
                                                      x,y,z  =      1_555 Check
PLAT434_ALERT_2_G Short Inter HL..HL Contact Cl4      ..Cl4      .       3.24 Ang.
                                                1-x,1-y,1-z  =      5_666 Check
PLAT794_ALERT_5_G Tentative Bond Valency for Cu1       (II)      .       2.31 Info
PLAT860_ALERT_3_G Number of Least-Squares Restraints ............           5 Note
PLAT910_ALERT_3_G Missing # of FCF Reflection(s) Below Theta(Min).          3 Note
PLAT912_ALERT_4_G Missing # of FCF Reflections Above STh/L=  0.600        628 Note
PLAT913_ALERT_3_G Missing # of Very Strong Reflections in FCF ....          1 Note
PLAT933_ALERT_2_G Number of HKL-OMIT Records in Embedded .res File          4 Note
PLAT941_ALERT_3_G Average HKL Measurement Multiplicity ..........         4.5 Low
PLAT978_ALERT_2_G Number C-C Bonds with Positive Residual Density.         14 Info
```

```
   0 ALERT level A = Most likely a serious problem - resolve or explain
   2 ALERT level B = A potentially serious problem, consider carefully
   7 ALERT level C = Check. Ensure it is not caused by an omission or oversight
  18 ALERT level G = General information/check it is not something unexpected

   0 ALERT type 1 CIF construction/syntax error, inconsistent or missing data
  16 ALERT type 2 Indicator that the structure model may be wrong or deficient
   6 ALERT type 3 Indicator that the structure quality may be low
   4 ALERT type 4 Improvement, methodology, query or suggestion
   1 ALERT type 5 Informative message, check
```

Datablock: 4

```
Bond precision:   C-C = 0.0044 A          Wavelength=0.71075

Cell:             a=17.9655(10)     b=9.431(4)         c=18.835(11)
                  alpha=90          beta=100.04(2)     gamma=90
Temperature:      93 K
```

	Calculated	Reported
Volume	3142(2)	3142(2)
Space group	C 2/c	C 1 2/c 1
Hall group	-C 2yc	-C 2yc
Moiety formula	C32 H30 Cu2 N4 O9	C32 H30 Cu2 N4 O9
Sum formula	C32 H30 Cu2 N4 O9	C32 H30 Cu2 N4 O9
Mr	741.70	741.70
Dx,g cm-3	1.568	1.568
Z	4	4
Mu (mm-1)	1.415	1.415
F000	1520.0	1520.0
F000'	1523.17	
h,k,lmax	21,11,22	21,11,22
Nref	2896	2882
Tmin,Tmax	0.844,0.844	0.552,0.844
Tmin'	0.844	

Correction method= # Reported T Limits: Tmin=0.552 Tmax=0.844
AbsCorr = MULTI-SCAN

Data completeness= 0.995 Theta(max)= 25.376

R(reflections)= 0.0393(2676) wR2(reflections)= 0.1192(2882)

S = 1.109 Npar= 219

The following ALERTS were generated. Each ALERT has the format
test-name_ALERT_alert-type_alert-level.
Click on the hyperlinks for more details of the test.

Alert level B

PLAT094_ALERT_2_B Ratio of Maximum / Minimum Residual Density .... 4.08 Report

---

Alert level C

PLAT220_ALERT_2_C NonSolvent Resd 1 C Ueq(max)/Ueq(min) Range 3.7 Ratio
PLAT230_ALERT_2_C Hirshfeld Test Diff for O17 --C17 . 6.3 s.u.
PLAT241_ALERT_2_C High 'MainMol' Ueq as Compared to Neighbors of O18 Check
PLAT250_ALERT_2_C Large U3/U1 Ratio for Average U(i,j) Tensor .... 2.6 Note
PLAT911_ALERT_3_C Missing FCF Refl Between Thmin & STh/L= 0.600 8 Report
PLAT971_ALERT_2_C Check Calcd Resid. Dens. 2.28Ang From O18 1.93 eA-3
PLAT976_ALERT_2_C Check Calcd Resid. Dens. 0.88Ang From O18 . -0.44 eA-3

Alert level G

PLAT002_ALERT_2_G Number of Distance or Angle Restraints on AtSite 2 Note
PLAT083_ALERT_2_G SHELXL Second Parameter in WGHT Unusually Large 5.37 Why ?

```
PLAT172_ALERT_4_G The CIF-Embedded .res File Contains DFIX Records          1 Report
PLAT794_ALERT_5_G Tentative Bond Valency for Cu1       (II)      .       2.18 Info
PLAT860_ALERT_3_G Number of Least-Squares Restraints ............           1 Note
PLAT912_ALERT_4_G Missing # of FCF Reflections Above STh/L=  0.600          6 Note
PLAT933_ALERT_2_G Number of HKL-OMIT Records in Embedded .res File          5 Note
PLAT978_ALERT_2_G Number C-C Bonds with Positive Residual Density.          5 Info
```

```
   0 ALERT level A = Most likely a serious problem - resolve or explain
   1 ALERT level B = A potentially serious problem, consider carefully
   7 ALERT level C = Check. Ensure it is not caused by an omission or oversight
   8 ALERT level G = General information/check it is not something unexpected

   0 ALERT type 1 CIF construction/syntax error, inconsistent or missing data
  11 ALERT type 2 Indicator that the structure model may be wrong or deficient
   2 ALERT type 3 Indicator that the structure quality may be low
   2 ALERT type 4 Improvement, methodology, query or suggestion
   1 ALERT type 5 Informative message, check
```

Datablock: 5

```
Bond precision:   C-C = 0.0039 A          Wavelength=0.71073

Cell:           a=10.1076(3)      b=11.5890(3)      c=12.0092(3)
                alpha=66.113(2)   beta=65.541(3)    gamma=72.032(2)
Temperature:    173 K

                Calculated                    Reported
Volume          1153.92(6)                    1153.92(6)
Space group     P -1                          P -1
Hall group      -P 1                          -P 1
Moiety formula  C24 H16 Cu I N4, I, H2 O      C24 H16 Cu I N4, I, H2 O
Sum formula     C24 H18 Cu I2 N4 O            C24 H18 Cu I2 N4 O
Mr              695.77                        695.76
Dx,g cm-3       2.003                         2.002
Z               2                             2
Mu (mm-1)       3.646                         3.646
F000            666.0                         666.0
F000'           664.94
h,k,lmax        13,15,16                      13,15,15
Nref            6234                          5085
Tmin,Tmax                                     0.016,0.038
Tmin'           0.844

Correction method= # Reported T Limits: Tmin=0.016 Tmax=0.038
AbsCorr = SPHERE
```

Data completeness= 0.816	Theta(max)= 29.144
R(reflections)= 0.0254(4506)	wR2(reflections)= 0.0670(5085)
S = 1.076	Npar= 297

The following ALERTS were generated. Each ALERT has the format
test-name_ALERT_alert-type_alert-level.
Click on the hyperlinks for more details of the test.

Alert level C

```
PLAT094_ALERT_2_C Ratio of Maximum / Minimum Residual Density ....       3.20 Report
PLAT911_ALERT_3_C Missing FCF Refl Between Thmin & STh/L=    0.600         45 Report
```

Alert level G

```
PLAT002_ALERT_2_G Number of Distance or Angle Restraints on AtSite          3 Note
PLAT172_ALERT_4_G The CIF-Embedded .res File Contains DFIX Records          1 Report
PLAT232_ALERT_2_G Hirshfeld Test Diff (M-X)  I1       --Cu1      .       19.8 s.u.
PLAT794_ALERT_5_G Tentative Bond Valency for Cu1       (II)      .       2.14 Info
PLAT860_ALERT_3_G Number of Least-Squares Restraints .............          2 Note
PLAT912_ALERT_4_G Missing # of FCF Reflections Above STh/L=  0.600       1073 Note
PLAT941_ALERT_3_G Average HKL Measurement Multiplicity ...........        3.0 Low
PLAT978_ALERT_2_G Number C-C Bonds with Positive Residual Density.          5 Info
```

---

0 **ALERT level A** = Most likely a serious problem - resolve or explain
0 **ALERT level B** = A potentially serious problem, consider carefully
2 **ALERT level C** = Check. Ensure it is not caused by an omission or oversight
8 **ALERT level G** = General information/check it is not something unexpected

0 ALERT type 1 CIF construction/syntax error, inconsistent or missing data
4 ALERT type 2 Indicator that the structure model may be wrong or deficient
3 ALERT type 3 Indicator that the structure quality may be low
2 ALERT type 4 Improvement, methodology, query or suggestion
1 ALERT type 5 Informative message, check

Datablock: 6

Bond precision:	C-C = 0.0046 A		Wavelength=0.71075
Cell:	a=14.387(3)	b=17.196(3)	c=9.9809(19)
	alpha=90	beta=90	gamma=90
Temperature:	93 K		

	Calculated	Reported
Volume	2469.3(8)	2469.3(8)
Space group	P n m a	P n m a
Hall group	-P 2ac 2n	-P 2ac 2n
Moiety formula	C24 H16 Cu2 I2 N4, C H4 O	C24 H16 Cu2 I2 N4, C H4 O
Sum formula	C25 H20 Cu2 I2 N4 O	C25 H20 Cu2 I2 N4 O
Mr	773.35	773.36
Dx,g cm-3	2.080	2.080
Z	4	4
Mu (mm-1)	4.249	4.250
F000	1480.0	1480.0
F000'	1479.17	
h,k,lmax	17,20,12	17,20,12
Nref	2342	2325
Tmin,Tmax	0.880,0.919	0.703,0.919
Tmin'	0.880	

Correction method= # Reported T Limits: Tmin=0.703 Tmax=0.919
AbsCorr = MULTI-SCAN

Data completeness= 0.993 Theta(max)= 25.381

R(reflections)= 0.0239(2228) wR2(reflections)= 0.0621(2325)

S = 1.063 Npar= 162

The following ALERTS were generated. Each ALERT has the format
test-name_ALERT_alert-type_alert-level.
Click on the hyperlinks for more details of the test.

Alert level B

PLAT420_ALERT_2_B D-H Bond Without Acceptor O16 --H16 . Please Check

---

Alert level C

PLAT910_ALERT_3_C Missing # of FCF Reflection(s) Below Theta(Min). 6 Note
PLAT911_ALERT_3_C Missing FCF Refl Between Thmin & STh/L= 0.600 9 Report
PLAT971_ALERT_2_C Check Calcd Resid. Dens. 1.97Ang From I2 2.05 eA-3
PLAT971_ALERT_2_C Check Calcd Resid. Dens. 0.83Ang From I2 1.99 eA-3
PLAT971_ALERT_2_C Check Calcd Resid. Dens. 1.49Ang From C15 1.82 eA-3
PLAT975_ALERT_2_C Check Calcd Resid. Dens. 1.09Ang From O16 . 0.53 eA-3
PLAT977_ALERT_2_C Check Negative Difference Density on H15A . -0.61 eA-3
PLAT977_ALERT_2_C Check Negative Difference Density on H15C . -0.45 eA-3

Alert level G

PLAT007_ALERT_5_G Number of Unrefined Donor-H Atoms .... 1 Report

```
PLAT083_ALERT_2_G SHELXL Second Parameter in WGHT  Unusually Large       8.83 Why ?
PLAT232_ALERT_2_G Hirshfeld Test Diff (M-X)  I1       --Cu1      .       10.3 s.u.
PLAT232_ALERT_2_G Hirshfeld Test Diff (M-X)  I2       --Cu1      .       11.3 s.u.
PLAT300_ALERT_4_G Atom Site Occupancy of H15A       Constrained at        0.5 Check
PLAT300_ALERT_4_G Atom Site Occupancy of H15B       Constrained at        0.5 Check
PLAT300_ALERT_4_G Atom Site Occupancy of H15C       Constrained at        0.5 Check
PLAT300_ALERT_4_G Atom Site Occupancy of H16        Constrained at        0.5 Check
PLAT413_ALERT_2_G Short Inter XH3 .. XHn     H9       ..H15A     .       1.96 Ang.
                                      3/2-x,1-y,1/2+z  =      4_665 Check
PLAT413_ALERT_2_G Short Inter XH3 .. XHn     H14      ..H15C     .       1.99 Ang.
                                            x,1/2-y,z  =      7_565 Check
PLAT779_ALERT_4_G Suspect or Irrelevant (Bond) Angle(s) in CIF ...      25.60 Deg.
              H15C -C15  -H15A    1_555   1_555   7_565 ......     #   67 Check
PLAT779_ALERT_4_G Suspect or Irrelevant (Bond) Angle(s) in CIF ...      25.60 Deg.
              H15B -C15  -H15B    1_555   1_555   7_565 ......     #   70 Check
PLAT779_ALERT_4_G Suspect or Irrelevant (Bond) Angle(s) in CIF ...      25.60 Deg.
              H15A -C15  -H15C    1_555   1_555   7_565 ......     #   74 Check
PLAT912_ALERT_4_G Missing # of FCF Reflections Above STh/L=  0.600          2 Note
PLAT933_ALERT_2_G Number of HKL-OMIT Records in Embedded .res File          9 Note
PLAT978_ALERT_2_G Number C-C Bonds with Positive Residual Density.          7 Info
```

---

0 **ALERT level A** = Most likely a serious problem - resolve or explain
1 **ALERT level B** = A potentially serious problem, consider carefully
8 **ALERT level C** = Check. Ensure it is not caused by an omission or oversight
16 **ALERT level G** = General information/check it is not something unexpected

0 ALERT type 1 CIF construction/syntax error, inconsistent or missing data
14 ALERT type 2 Indicator that the structure model may be wrong or deficient
2 ALERT type 3 Indicator that the structure quality may be low
8 ALERT type 4 Improvement, methodology, query or suggestion
1 ALERT type 5 Informative message, check

It is advisable to attempt to resolve as many as possible of the alerts in all categories. Often the minor alerts point to easily fixed oversights, errors and omissions in your CIF or refinement strategy, so attention to these fine details can be worthwhile. In order to resolve some of the more serious problems it may be necessary to carry out additional measurements or structure refinements. However, the purpose of your study may justify the reported deviations and the more serious of these should normally be commented upon in the discussion or experimental section of a paper or in the "special_details" fields of the CIF. checkCIF was carefully designed to identify outliers and unusual parameters, but every test has its limitations and alerts that are not important in a particular case may appear. Conversely, the absence of alerts does not guarantee there are no aspects of the results needing attention. It is up to the individual to critically assess their own results and, if necessary, seek expert advice.

Publication of your CIF in IUCr journals

A basic structural check has been run on your CIF. These basic checks will be run on all CIFs submitted for publication in IUCr journals (*Acta Crystallographica*, *Journal of Applied Crystallography*, *Journal of Synchrotron Radiation*); however, if you intend to submit to *Acta Crystallographica Section C* or *E* or *IUCrData*, you should make sure that full publication checks are run on the final version of your CIF prior to submission.

Publication of your CIF in other journals

Please refer to the *Notes for Authors* of the relevant journal for any special instructions relating to CIF submission.

PLATON version of 10/05/2023; check.def file version of 10/05/2023

Datablock 3OAc - ellipsoid plot

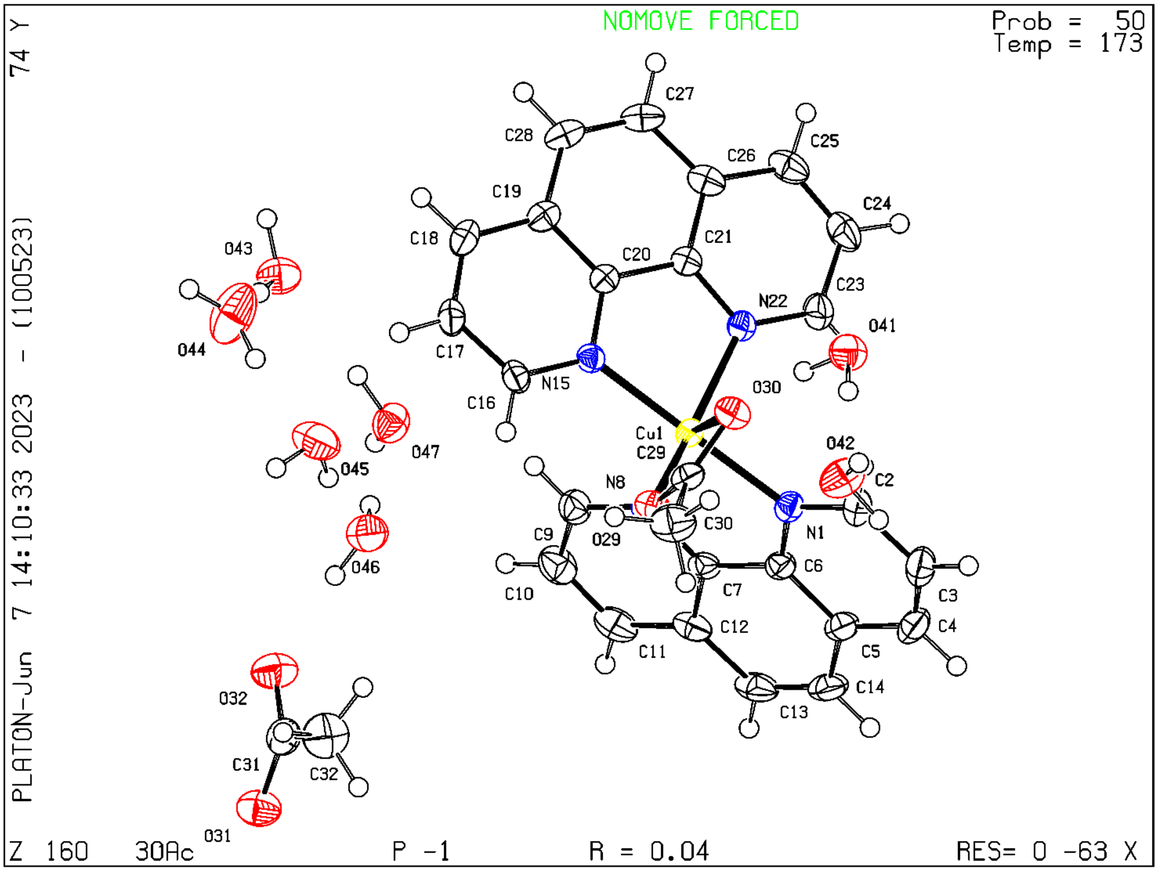

Datablock 3Cl - ellipsoid plot

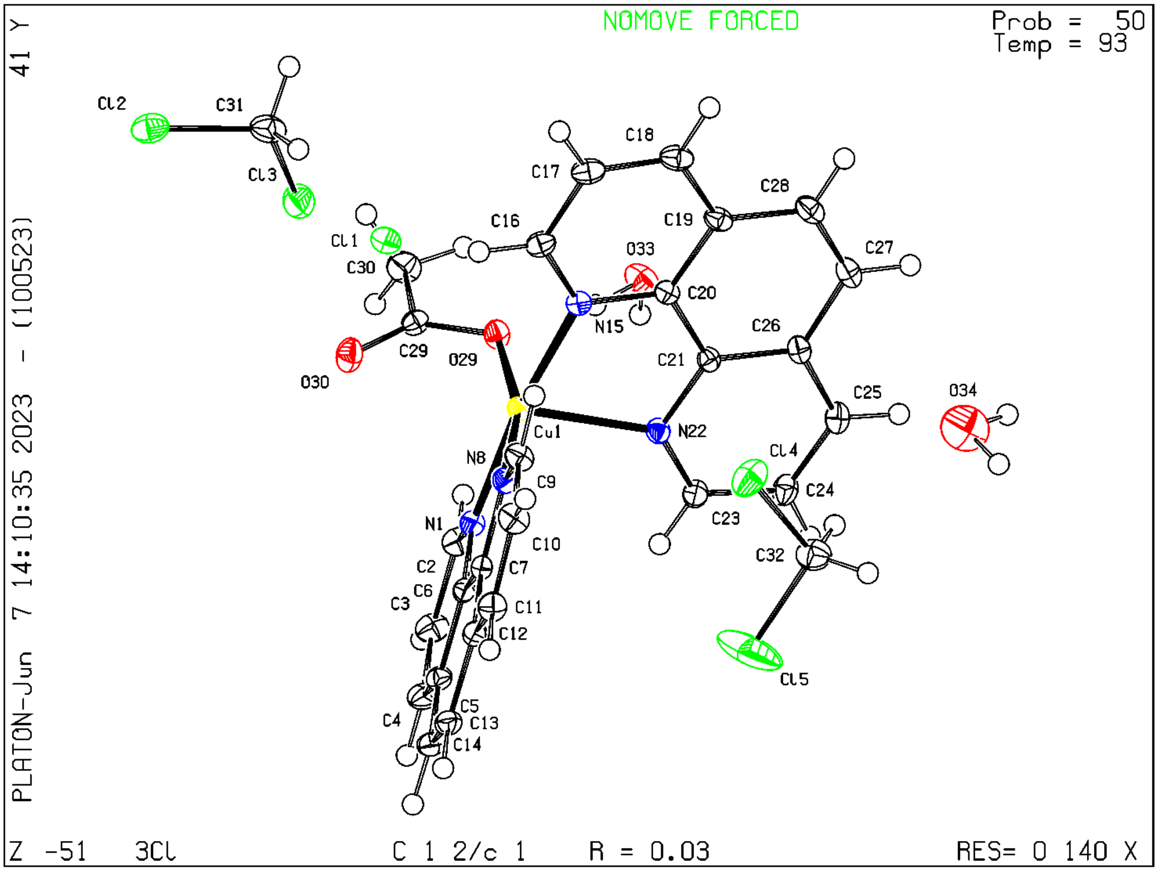

Datablock 4 - ellipsoid plot

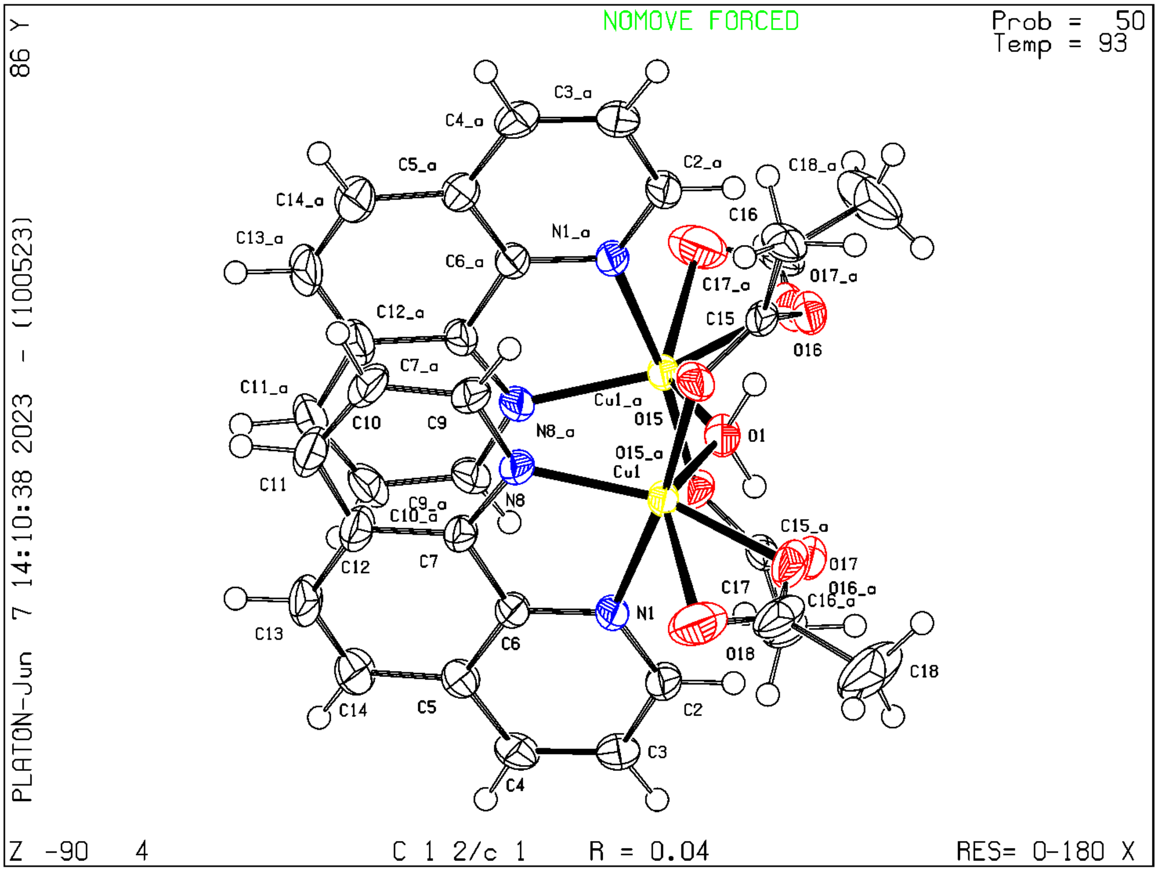

Datablock 5 - ellipsoid plot

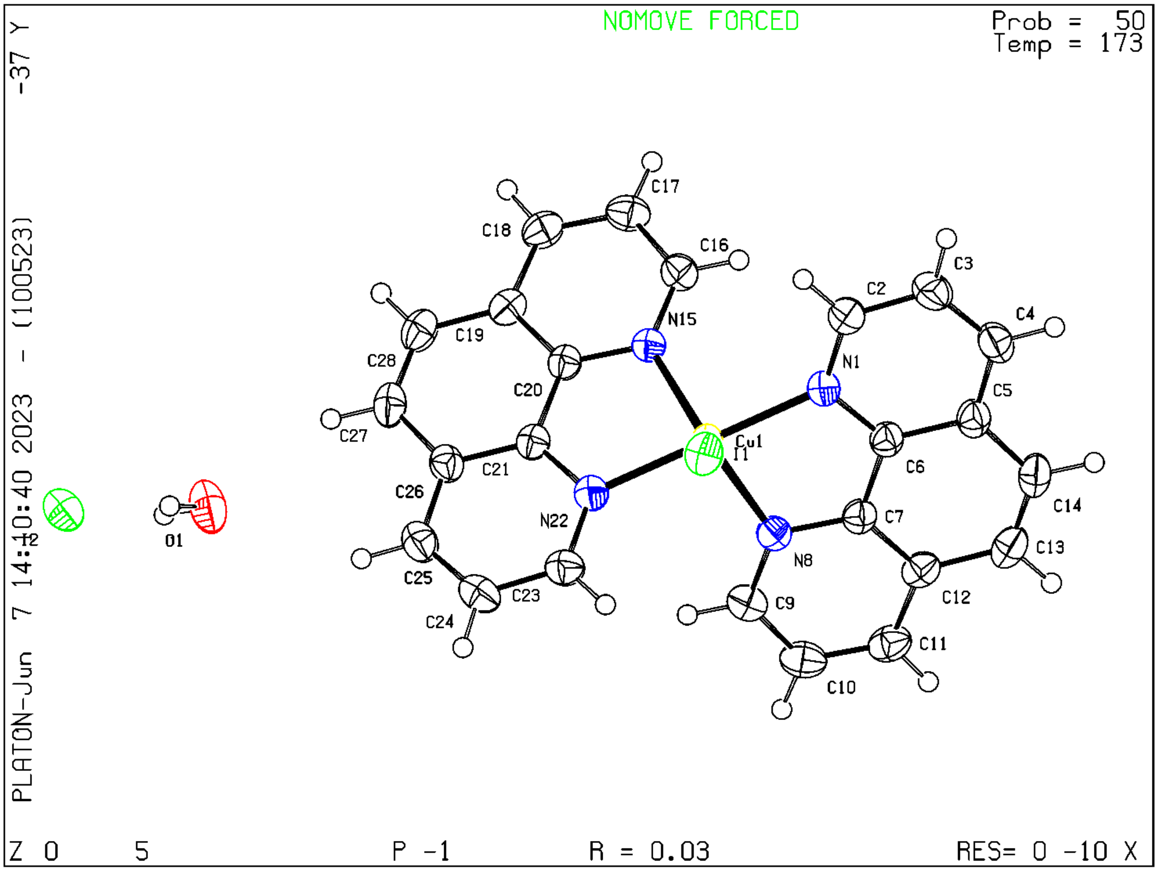

Datablock 6 - ellipsoid plot

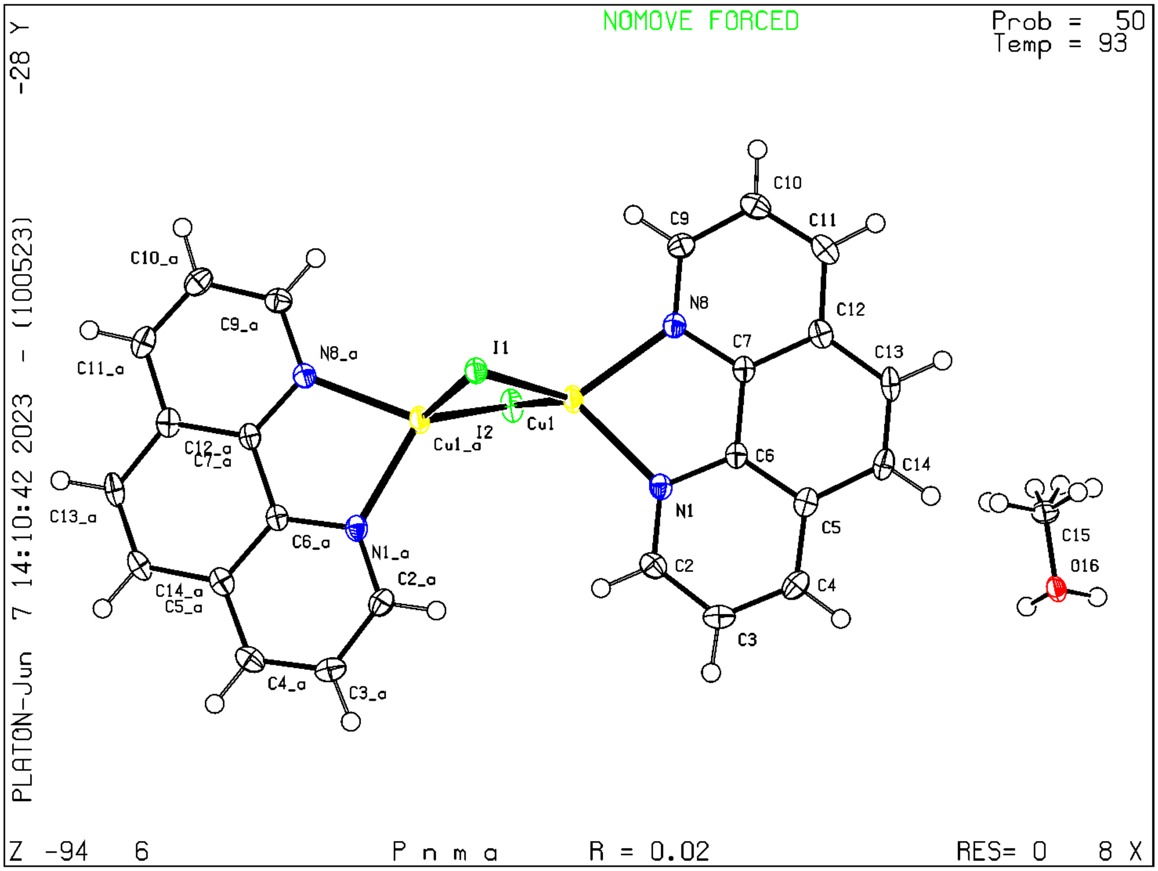

Supplement: Supplementary file 3 — cs3c02839_si_003.pdf [file cs3c02839_si_003.pdf]
